# Benzo[*d*]oxazoles from Anilides by *N*-Deprotonation–*O*-S_N_Ar Cyclization

**DOI:** 10.3390/molecules29184322

**Published:** 2024-09-12

**Authors:** Nash E. Nevels, Luke Subera, Richard A. Bunce

**Affiliations:** Department of Chemistry, Oklahoma State University, Stillwater, OK 74078-3071, USA; nnevels@okstate.edu (N.E.N.); luke.subera@okstate.edu (L.S.)

**Keywords:** benzo[*d*]oxazoles, *N*-deprotonation–*O*-S_N_Ar cyclization, ring formation, annulation

## Abstract

A synthesis of benzo[*d*]oxazoles by an *N*-deprotonation–*O*-S_N_Ar cyclization sequence from anilide precursors is reported. Anilides derived from 2-fluorobenzaldehydes, activated toward S_N_Ar ring closure by C5 electron-withdrawing groups, were prepared and subjected to deprotonation–cyclization using 2 equiv. of K_2_CO_3_ in anhydrous DMF. Following deprotonation at nitrogen, the delocalized anion cyclized from the amide oxygen to give high yields of benzo[*d*]oxazoles. The temperature required for the cyclization of benzanilides correlated with the potency of the C5 activating group on the S_N_Ar acceptor ring with nitro (most potent) reacting at 90 °C (1 h), cyano reacting at 115 °C (1 h), methoxycarbonyl reacting at 120 °C (2 h), and trifluoromethyl (least potent) reacting at 130 °C (3 h). Acetanilides were more difficult to cyclize but generally required 4–6 h at these same temperatures for completion. Product purification was accomplished by recrystallization or chromatography.

## 1. Introduction

Recent work in our laboratory has sought to develop novel methods to fuse 5-membered heterocycles to aromatic scaffolds. One previous project described the intermolecular reaction of 2-fluoro-5-nitrobenzaldehydes and similarly substituted acetophenones with arylhydrazine hydrochlorides in the presence of K_2_CO_3_ in DMF at 90 °C to generate 1-aryl-5-nitro-1*H*-indazoles [1]. This transformation involved a sequential reaction for the benzaldehydes while a domino process was achieved with the acetophenones. A copper-catalyzed variant was also advanced for systems lacking a C5-activating group in the precursor molecules. These reactions led to high yields of indazoles, which constitute an important scaffold in drug research. The current work describes a two-step synthesis of benzo[*d*]oxazoles from a series of commercial C5-substituted 2-fluoroanilines. The acylation of the aniline to give the acetanilide or benzanilide, followed by intramolecular-base-induced cyclization via a *N*-deprotonation-*O*-S_N_Ar sequence, gives the benzo[*d*]oxazoles, which are high-value targets as prospective anticancer and antibiotic drugs.

There are numerous synthetic approaches to the benzo[*d*]oxazole ring system. One of the earliest methods involved the cyclocondensation of 2-aminophenols with carboxylic acids using heat [2] as well as Lawesson’s reagent under microwave irradiation [3]. In a related process, Brønsted’s [4] and Lewis’s [5] acid-catalyzed reactions of 2-aminophenols with orthoesters also proved successful in generating these targets. Further condensative ring closures of 2-aminophenols with (a) dimethylformamide derivatives promoted by catalytic imidazolium chloride [6], (b) aldehydes in the presence Sm(OTf)_3_ [7], or (c) β-diketones with catalytic *p*-TsOH·H_2_O and CuI [8] provided alternative routes to these structures. Several other copper-promoted reactions were utilized to close benzo[*d*]oxazole rings [9,10,11,12,13] including one report that paralleled the current reaction. This involved the treatment of unactivated *N*-(2-bromophenyl)benzamides with base in the presence of 5 mol% of copper(II) oxide nanoparticles under aerobic conditions to induce the ring closure process [13]. Furthermore, a similar iridium-catalyzed transformation involving this class of substrates has been recently reported [14]. Several oxidative ring closures of 2-hydroxyarylimines with hypochlorite and Dess–Martin periodinane were also advanced as routes to these heterocyclic systems [15,16,17]. Finally, C–H bond arylation with aryl chlorides [18] and aryltrimethylammonium triflates [19] to introduce aromatic groups at C2 of unsubstituted benzo[*d*]oxazoles has been reported using various palladium catalysts.

**Figure 1:**
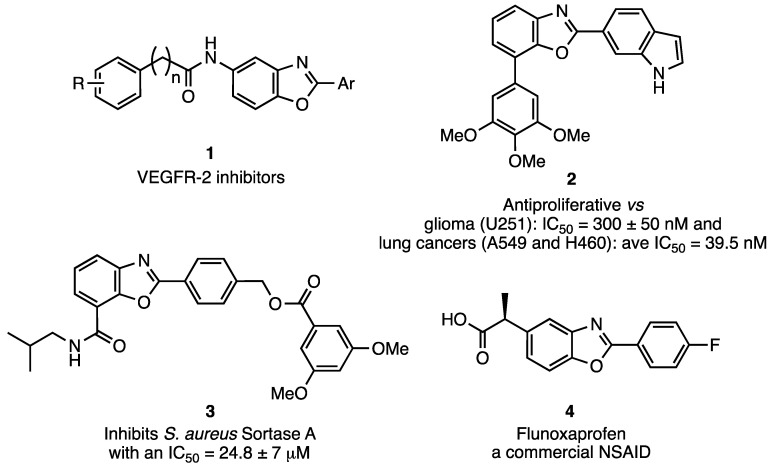
Experimental and commercial drugs based on the benzo[*d*]oxazole system.

Benzo[*d*]oxazoles have a broad spectrum of pharmaceutical activities in drug research (see Figure 1). The anticancer properties of this class of compounds have recently been reviewed [20]. In this survey, a series of benzo[*d*]oxazoles (generalized as **1**) were claimed to be effective inhibitors of vascular epithelial growth factor receptor-2 (VEGFR-2). VEGF-VEGFR-2 is an important signaling pathway for angiogenesis that can promote the abnormal growth and migration of tumors. Since angiogenesis appears to be crucial in the transition of a tumor from benign to malignant, it is an important therapeutic target. Compounds such as **1** could be easily prepared using the current method followed by nitro reduction and *N*-acylation. A further exploration of benzo[*d*]oxazoles has revealed that derivatives such as **2** demonstrate antiproliferative activity against other cancers [21] while **3** lowers the potency of *S. aureus* by preventing the enzyme Sortase A from linking virulence factors to the cell wall at the site of infection [22]. Finally, simple derivatives, such as Flunoxaprofen (**4**), is a commercial drug expressing non-steroidal anti-inflammatory activity [23].

## 2. Results and Discussion

The current project sought to access benzo[*d*]oxazoles from suitably substituted acetanilide and benzanilide derivatives. Starting from 2-fluoroanilines (1 equiv.), activated a C5 by NO_2_, CN, CO_2_Me, and CF_3_, the amino groups were converted to amides **5**–**38** by reaction with various acid chlorides (0.99 equiv.) in the presence of triethylamine (2 equiv.). Yields were generally in the 50–86% range. Though the yields were not optimized for this reaction, it was necessary to have a slight excess of the 2-fluoroaniline derivative to prevent double acylation, which was observed in several cases when excess acid chloride was present.

**Scheme 1:**
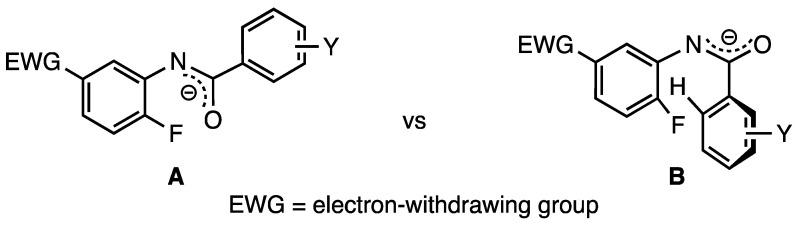
Possible conformations for the delocalized amide anion.

Relatively strong precedent exists for the formation of benzo[*d*]oxazoles from benzanilides as a similar cyclization has been disclosed using a copper catalyst on aromatic systems lacking S_N_Ar activation [13]. The current reaction requires the addition of the oxygen atom of a deprotonated amide to a C5 activated acceptor ring by a 5-exo-trig cyclization–elimination [24]. The presence of activating groups at C5 obviates the need for a copper catalyst and simplifies the purification. The current reaction, however, complements this earlier report by making available numerous derivatives with substituent groups that allow for the further elaboration of the structure. Although the closure of a five-membered ring containing two double bonds significantly increases strain in the system, the aromaticity gained in forming the benzo[*d*]oxazole framework could potentially offset this deterrent to reaction. Conformationally, rotamer A (Scheme 1) is sterically more favorable and would allow the reactive centers to readily align for ring closure, perhaps further promoted by the potential formation of aromatic-based intramolecular interactions [25]. The alternative rotamer B has a high-energy aryl–aryl steric repulsion and would, therefore, be less prevalent. Finally, for acetanilides, it was correctly anticipated that the enolizability of the side chain would be a problem. The pivalamide substrates proved better than acetamides and hexanamides due to the lack of enolizable protons and possibly a Thorpe–Ingold effect [26] on ring closure but still suffered some degradation upon prolonged exposure to base and heat.

**Scheme 2:**
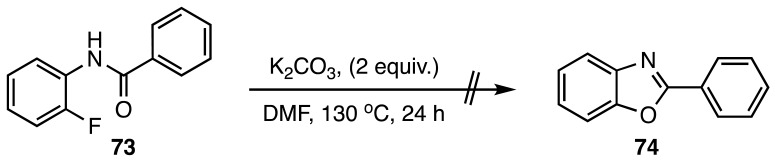
Control reaction of *N*-(2-fluorophenyl)benzamide (**73**).

Our results are summarized in Table 1. As expected, the conversion proceeded in high yield for benzanilides and lower yields for acetanilides. The reaction required only base and heat to initiate the cyclization. No other additives or catalysts were required, and thus the removal of trace metals was not required in the product purification scheme. Despite the high temperature conditions the reaction was remarkably clean. In the case of benzanilides, no major side reactions were noted. Additionally, while the sensitivity of the acetanilide side chains to the basic conditions led to lower yields, no isolable side products were generated. Since the substrate loses only a proton and a fluoride ion (20 amu total), the transformation possesses high atom economy [27,28]. The required temperature for benzanilide cyclizations correlated with the potency of the activating group at C5 of the S_N_Ar acceptor ring. Nitro was the most powerful activator, and reactions of benzanilides 8–14 were complete in 1 h at 90 °C. Cyano- and methoxycarbonyl-activated substrates, 18–24 and 25–31, respectively, showed intermediate reactivity requiring 1 h at 115 °C and 2 h at 120 °C. Finally, the trifluoromethyl group was the least potent activator, and precursors 32–38 incorporating this moiety required 3 h at 130 °C for complete conversion. Acetanilides 5–7 required 4–6 h at 90 °C while 15–17 needed 4–6 h at 115 °C, and reaction progress proved more difficult to monitor by thin layer chromatography (TLC). The extended heating of these substrates in the presence of K_2_CO_3_ at >115 °C led to decreased yields, and, as a result, acetanilide derivatives with CO_2_Me and CF_3_ ring-activating groups were not studied. A control reaction using *N*-(2-fluorophenyl)benzamide (**73**) lacking a C5 activating group failed to give 2-phenylbenzo[*d*]oxazole (**74**) even after 24 h at 130 °C in DMF (Scheme 2).

The purification of the products was accomplished using two different protocols. For highly crystalline benzo[*d*]oxazoles, products were isolated by recrystallization from 1–5% ethyl acetate in petroleum ether (Method A). Oils and lower-melting solids were best purified by concentrating the crude product onto 1–2 grams of silica gel, adding this to the top of a 25 cm × 1 cm silica gel column, and eluting with 5–25% ether in petroleum ether (Method B). In general, this chromatographic procedure consistently yielded cleaner products in higher yields with less loss due to multiple crystallizations and static repulsion during transfer to storage vials. Method B was also superior in allowing for the recovery of unreacted material, which was common in the acetanilide series. For these reactions, unreacted material generally eluted with 30–50% ether in petroleum ether. Reactions to prepare 39, 40, 68, and 71 (Table 1) illustrate the yield enhancements achievable using this purification strategy.

The benzo[*d*]oxazoles were characterized by spectroscopic methods. Copies of the ^1^H, ^13^C, and some ^19^F NMR spectra of the amide precursors as well as the benzo[*d*]oxazole products are given in the Supplemental Materials. The starting anilides all showed a downfield N–H singlet in the ^1^H NMR run in DMSO-*d_6_* as well as a prominent N–H stretch in the IR spectrum. The IR also showed a moderate C=O absorbance in the 1680–1650 cm^−1^ range. These were absent in the final products. The ^1^H NMR spectra were generally simpler for the benzo[*d*]oxazoles as there was one less fluorine in the structure to couple with neighboring carbon and hydrogen nuclei. The benzo[*d*]oxazoles were chromatographically less polar than the starting amides and several of them (especially those substituted at C2 by halogenated aromatics) fluoresced a bright blue color when viewed with UV light on a TLC plate. 

**Scheme 3:**
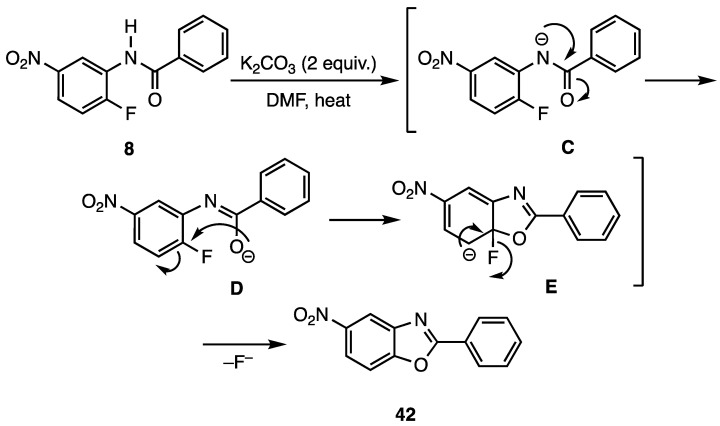
Probable mechanism for cyclization of *N*-(2-fluoro-5-nitrophenyl)benzamide (**8**) to 2-phenyl-5-nitrobenzo[*d*]oxazole (**42**).

Scheme 3 illustrates the probable mechanism for the reaction of benzanilide *N*-(2-fluoro-5-nitrophenyl)benzamide (**8**) to give 2-phenyl-5-nitrobenzo[*d*]oxazole (**42**). The cyclization process would require an initial deprotonation of the relatively acidic amide nitrogen (pKa = *ca*. 18.8 for benzanilides and pKa = *ca*. 21.5 for acetanilides [29,30]) to give delocalized amide anion C. Once intermediate C is produced, the oxygen of the delocalized anion contributor D would add to the fluorine-bearing aromatic carbon to generate the Meisenheimer complex E. Rearomatization by the loss of fluoride ion would then afford the desired benzo[*d*]oxazoles.

## 3. Materials and Methods

### 3.1. General Methods

Unless otherwise indicated, all reactions were performed under dry N_2_ in dry glassware. All commercial reagents and solvents were used as received. Reactions were monitored by TLC on Analtech No 21521 silica gel GF plates (Newark, DE, USA). Preparative separations were performed on Davisil^®^, grade 62, 60–200 mesh silica gel containing 0.5% of UV-05 phosphor (both from Sorbent Technologies, Norcross, GA, USA) slurry packed into quartz columns. Band elution for all chromatographic separations was detected using a hand-held UV light source (Fisher Scientific, Pittsburgh, PA, USA). Melting points (uncorrected) were obtained using a MEL-TEMP apparatus (Cambridge, MA, USA). FT-IR spectra were run using an Agilent Cary 630 spectrometer (Santa Clara, CA, USA) fitted with an ATR sampling module. ^1^H- and ^13^C-NMR spectra were measured using a Bruker Avance 400 system (Billerica, MA, USA) at 400 MHz and 101 MHz, respectively, in DMSO-*d_6_* or CDCl_3_ containing 0.05% *v*/*v* tetramethylsilane as the internal standard. Chemical shifts are given in δ (ppm) units relative to the standard and coupling constants (*J*) are given in Hz. ^19^F-NMR spectra were collected on the same instrument and are referenced to internal C_6_H_5_F at δ –113.15. Low-resolution mass spectra were obtained using a Hewlett-Packard Model 1800A GCD GC–MS system (Palo Alto, CA, USA). Elemental analyses (±0.4%) on all new compounds were determined by Atlantic Microlabs (Norcross, GA, USA).

### 3.2. Representative Procedure for Preparing Acetanilide and Benzanilide Derivatives 

In a 50 mL round-bottomed flask equipped with a magnetic stirrer, an addition funnel, and a drying tube, was added 2-fluoro-5-nitroaniline (1.00 g, 6.41 mmol, 1 equiv.) and dichloromethane (20 mL). Acetyl chloride (0.50 g, 0.45 mL, 6.35 mmol, 0.99 equiv.) was added dropwise at 23 °C and followed by slow addition of triethylamine (1.29 g, 1.78 mL, 12.82 mmol, 2 equiv.). The reaction was heated at reflux for 12–18 h and then cooled and added to saturated aqueous NaCl (25 mL). The layers were separated, and the organic phase was washed with 1 M HCl (1 × 25 mL) and saturated aqueous NaCl (3 × 25 mL), dried (Na_2_SO_4_) and concentrated under vacuum to give the crude amide as a dark solid. Recrystallization of the product from absolute ethanol gave the acetanilide as a tan, off-white, or gray solid. Benzanilides were prepared using the same procedure. All amide forming reactions were performed using 1.00 g of the corresponding aniline with other reagents scaled accordingly.

#### 3.2.1. *N*-(2-Fluoro-5-nitrophenyl)acetamide (**5**)

Yield: 0.79 g (62%) as a tan solid, m.p. 175–177 °C (lit. [31] m.p. 177–178 °C); IR: 3262, 1670, 1542, 1351 cm^−1^; ^1^H NMR (400 MHz, DMSO-*d_6_*): δ 10.18 (s, 1H), 9.00 (dd, *J* = 6.7, 2.9 Hz, 1H), 8.02 (ddd, *J* = 9.1, 4.8, 2.9 Hz, 1H), 7.55 (dd, *J* = 10.3, 9.1 Hz, 1H), 2.17 (s, 3H); ^13^C {^1^H} NMR (101 MHz, DMSO-*d_6_*): δ 170.0, 156.6 (d, *J* = 255.6 Hz), 144.2 (d, *J* = 2.6 Hz), 128.0 (d, *J* = 13.3 Hz), 120.5 (d, *J* = 9.5 Hz), 118.3 (d, *J* = 4.1 Hz), 117.0 (d, *J* = 22.5 Hz), 24.2; MS (*m*/*z*): 198 (M^+·^); Anal. Calcd for C_8_H_7_FN_2_O_3_: C, 48.49; H, 3.56; N, 14.14; Found: C, 48.40; H, 3.61; N, 14.06.

#### 3.2.2. *N*-(2-Fluoro-5-nitrophenyl)hexanamide (**6**)

Yield: 1.04 g (64%) as a tan solid, m.p. 85–86 °C; IR: 3272, 1666, 1538, 1351 cm^−1^; ^1^H NMR (400 MHz, CDCl_3_): δ 9.33 (dd, *J* = 6.8, 2.8 Hz, 1H), 7.97 (ddd, *J* = 12.7, 8.3, 5.4 Hz, 1H), 7.54 (br s, 1H), 7.23 (dd, *J* = 9.9, 8.9 Hz, 1H), 2.46 (t, *J* = 7.4 Hz, 2H), 1.73 (pentet, *J* = 7.4 Hz, 2H), 1.38 (m, 4H), 0.92 (t, *J* = 7.3 Hz, 3H); ^13^C {^1^H} NMR (101 MHz, CDCl_3_): δ 171.7, 155.3 (d, *J* = 253.2 Hz), 144.5, 127.4 (d, *J* = 11.7 Hz), 119.5 (d, J = 9.3 Hz), 117.3 (d, *J* = 3.3 Hz), 115.3 (d, *J* = 22.3 Hz), 37.6, 31.3, 25.0, 22.4, 13.9; MS (*m*/*z*): 254 (M^+^); Anal. Calcd for C_12_H_15_FN_2_O_3_: C, 56.69; H, 5.95; N, 11.02; Found: C, 56.63; H, 5.99; N, 10.95.

#### 3.2.3. *N*-(2-Fluoro-5-nitrophenyl)pivalamide (**7**)

Yield: 1.20 g (78%) as a tan solid, m.p. 75–77 °C; IR: 3295, 1672, 1521, 1328 cm^−1^; ^1^H NMR (400 MHz, CDCl_3_): δ 9.34 (dd, *J* = 6.9, 2.9 Hz, 1H), 7.97 (ddd, *J* = 9.1, 4.5, 2.9 Hz, 1H), 7.73 (br s, 1H), 7.24 (dd, *J* = 1.0, 9.1 Hz, 1H), 1.36 (s, 9H); ^13^C {^1^H} (NMR 101 MHz, CDCl_3_): δ 176.9, 155.5 (d, *J* = 252.7 Hz), 144.5, 127.5 (d, *J* = 11.4 Hz), 119.6 (d, *J* = 9.3 Hz), 117.3 (d, *J* = 3.3 Hz), 115.2 (d, *J* = 22.2 Hz), 40.2, 27.4; MS (*m*/*z*): 240 (M^+·^); Anal. Calcd for C_11_H_13_FN_2_O_3_: C, 55.00 H, 5.45; N, 11.66; Found: C, 54.89; H, 5.48; N, 11.58.

#### 3.2.4. *N*-(2-Fluoro-5-nitrophenyl)benzamide (**8**)

Yield: 1.07 g (64%) as an off-white solid, m.p. 170–171 °C; (lit. [32] m.p. 157–159 °C); IR: 3287, 1660, 1529, 1351 cm^−1^; ^1^H NMR (400 MHz, DMSO-*d_6_*): δ 10.48 (s, 1H), 8.68 (dd, *J* = 6.6, 2.9 Hz, 1H), 8.17 (m, 1H), 8.01 (d, *J* = 7.9 Hz, 2H), 7.67–7.54 (complex, 4H); ^13^C {^1^H} NMR (101 MHz, DMSO-*d_6_*): δ 166.3, 159.2 (d, *J* = 258.0 Hz), 144.1 (d, *J* = 2.7 Hz), 133.9, 132.7, 129.0, 128.5, 127.4 (d, *J* = 14.0 Hz), 121.6 (d, *J* = 9.9 Hz), 121.8 (d, *J* = 3.8 Hz), 117.6 (d, *J* = 22.9 Hz); MS (*m*/*z*): 260 (M^+·^); Anal. Calcd for C_13_H_9_FN_2_O_3_: C, 60.00; H, 3.49; N, 11.02; Found: C, 59.91; H, 3.57; N, 10.96.

#### 3.2.5. *N*-(2-Fluoro-5-nitrophenyl)-3-methylbenzamide (**9**)

Yield: 1.14 g (65%) as a light tan solid, m.p. 110–112 °C; IR: 3396, 1665, 1534, 1349 cm^−1^; ^1^H NMR (400 MHz, DMSO-*d_6_*): δ 10.43 (s, 1H), 8.67 (dd, *J* = 6.6, 2.9 Hz, 1H), 8.16 (ddd, *J* = 9.1, 4.1, 2.9 Hz, 1H), 7.82 (br s, 1H), 7.79 (m, 1H), 7.62 (t, *J* = 9.6 Hz, 1H), 7.45 (m, 2H), 2.41 (s, 3H); ^13^C {^1^H} NMR (101 MHz, DMSO-*d_6_*): δ 166.3, 159.2 (d, *J* = 258.0 Hz), 144.1 (d, *J* = 2.9 Hz), 138.4, 133.8, 133.3, 128.9, 127.6 (d, *J* = 13.9 Hz), 125.6, 122.5 (d, *J* = 9.7 Hz), 121.8 (d, *J* = 4.1 Hz), 117.4 (d, *J* = 23.0 Hz), 21.4; MS (*m*/*z*): 274 (M^+·^); Anal. Calcd for C_14_H_11_FN_2_O_3_: C, 61.31; H, 4.04: N, 10.21; Found: C, 61.22; H, 4.08; N, 10.09.

#### 3.2.6. *N*-(2-Fluoro-5-nitrophenyl)-4-methylbenzamide (**10**)

Yield: 1.21 g (69%) as a light tan solid, m.p. 201–202 °C; IR: 3298, 1651, 1536, 1349 cm^−1^; ^1^H NMR (400 MHz, DMSO-*d_6_*): δ 10.38 (s, 1H), 8.66 (dd, *J* = 6.6, 2.9 Hz, 1H), 8.16 (ddd, *J* = 9.1, 4.1, 2.9 Hz, 1H), 7.90 (d, *J* = 8.1 Hz, 1H), 7.62 (t, *J* = 9.6 Hz, 1H), 7.37 (d, *J* = 8.1 Hz, 2H), 2.40 (s, 3H); ^13^C {^1^H} NMR (101 MHz, DMSO-*d_6_*): δ 166.0, 159.2 (d, *J* = 257.9 Hz), 144.1 (d, *J* = 4.1 Hz), 142.9, 131.0, 129.5, 128.5, 127.6 (d, *J* = 13.9 Hz), 122.4 (d, *J* = 9.7 Hz), 121.8 (d, *J* = 4.0 Hz), 117.6 (d, *J* = 22.9 Hz), 21.5; MS (*m*/*z*): 274 (M^+·^); Anal. Calcd for C_14_H_11_FN_2_O_3_: C, 61.31; H, 4.04; N, 10.21; Found: C, 61.25; H, 4.10; N, 10.13.

#### 3.2.7. *N*-(2-Fluoro-5-nitrophenyl)-4-methoxybenzamide (**11**)

Yield: 1.24 g (67%) as a light tan solid, m.p. 174–175 °C; IR: 3300, 2840, 1657, 1532, 1351 cm^−1^; ^1^H NMR (400 MHz, DMSO-*d_6_*): δ 10.30 (s, 1H), 8.66 (dd, *J* = 6.6, 2.9 Hz, 1H), 8.14 (ddd, *J* = 9.1, 4.1,2.9 Hz, 1H), 8.00 (d, *J* = 8.9 Hz, 2H), 7.61 (t, *J* = 9.6 Hz, 1H), 7.10 (d, *J* = 8.9 Hz, 2H), 3.86 (s, 3H); ^13^C {^1^H} NMR (101 MHz, DMSO-*d_6_*): δ 165.6, 162.9, 159.2 (d, *J* = 257.8 Hz), 144.1 (d, *J* = 3.0 Hz), 130.5, 127.8 (d, *J* = 13.9 Hz), 125.9, 122.3 (d, *J* = 9.6 Hz), 121.7 (d, *J* = 4.0 Hz), 117.5 (d, *J* = 23.0 Hz), 114.3, 56.0; MS (*m*/*z*): 290 (M^+·^); Anal. Calcd for C_14_H_11_FN_2_O_4_: C, 57.93; H, 3.82; N, 9.65; Found: C, 57.87; H, 3.88; N, 9.54.

#### 3.2.8. 2-Fluoro-*N*-(2-fluoro-5-nitrophenyl)benzamide (**12**)

Yield: 1.57 g (86%) as a tan solid, m.p. 189–190 °C; IR: 3457, 1670, 1538, 1340 cm^−1^; ^1^H NMR (400 MHz, DMSO-*d_6_*): δ 10.58 (s, 1H), 8.90 (dd, *J* = 6.7, 2.9 Hz, 1H), 8.15 (ddd, *J* = 9.1, 4.1, 2.9 Hz, 1H), 7.77 (td, *J* = 7.4, 1.8 Hz, 1H), 7.68–7.58 (complex, 2H), 7.43–7.34 (complex, 2H); ^13^C {^1^H} NMR (101 MHz, DMSO-*d_6_*): δ 163.8, 159.7 (d, *J* = 249.8 Hz), 158.0 (d, *J* = 257.5 Hz), 144.2 (d, *J* = 2.8 Hz), 133.9 (d, *J* = 8.6 Hz), 130.9 (d, *J* = 2.4 Hz), 127.3 (d, *J* = 13.7 Hz), 125.1 (d, *J* = 3.4 Hz), 123.7 (d, *J* = 14.2 Hz), 122.1 (d, *J* = 9.7 Hz), 120.1 (d, *J* = 3.8 Hz), 117.5 (d, *J* = 22.5 Hz), 116.7 (d, *J* = 22.0 Hz); ^19^F {^1^H} NMR (376 Hz, DMSO-*d_6_* referenced to C_6_H_5_F): δ –112.39, –113.79; MS (*m*/*z*): 278 (M^+·^); Anal. Calcd for C_13_H_8_F_2_N_2_O_3_: C, 56.12; H, 2.90; N, 10.07; Found: C, 56.07; H, 2.94; N, 9.98.

#### 3.2.9. 3-Chloro-*N*-(2-fluoro-5-nitrophenyl)benzamide (**13**)

Yield: 1.07 g (57%) as a tan solid, m.p. 163–165 °C; IR: 3350, 1650, 1532, 1340 cm^−1^; ^1^H NMR (400 MHz, DMSO-*d_6_*): δ 10.59 (s, 1H), 8.68 (dd, *J* = 6.5, 2.9 Hz, 1H), 8.17 (m, 1H), 8.04 (t, *J* = 1.9 Hz, 1H), 7.95 (dt, *J* = 7.8, 1.4 Hz, 1H), 7.70 (m, 1H), 7.65–7.57 (complex, 2H); ^13^C {^1^H} NMR (101 MHz, DMSO-*d_6_*): δ 164.9, 159.1 (d, *J* = 257.7 Hz), 144.1 (d, *J* = 2.8 Hz), 135.8, 133.8, 132.4, 130.9 (d, *J* = 3.5 Hz), 128.3, 127.3, 127.2, 122.7 (d, *J* = 10.2 Hz), 121.9 117.6 (d, *J* = 22.6 Hz); MS (*m*/*z*): 294, 296 (*ca* 3:1, M^+·^); Anal. Calcd for C_13_H_8_ClFN_2_O_3_: C, 59.22; H, 2.75; N, 9.51; Found: C, 59.17; H, 2.82; N, 9.45.

#### 3.2.10. 4-Chloro-*N*-(2-fluoro-5-nitrophenyl)benzamide (**14**)

Yield: 1.13 g (60%) as a tan solid, m.p. 167–169 °C; IR: 3298, 1660, 1525, 1340 cm^−1^; ^1^H NMR (400 MHz, DMSO-*d_6_*): δ 10.57 (s, 1H), 8.67 (dd, *J* = 6.6, 2.9 Hz, 1H), 8.17 (ddd, *J* = 9.1, 4.2, 2.9 Hz, 1H), 8.01 (d, *J* = 8.5 Hz, 2H), 7.65 (d, *J* = 8.5 Hz, 2H), 7.64 (t, *J* = 9.1 Hz, 1H); ^13^C {^1^H} NMR (101 MHz, DMSO-*d_6_*): δ 165.3, 159.2 (d, *J* = 257.7 Hz), 144.2 (d, *J* = 2.7 Hz), 137.6, 132.6, 130.4, 129.1, 127.3 (d, *J* = 13.9 Hz), 122.7 (d, *J* = 9.6 Hz), 121.9, 117.6 (d, *J* = 22.9 Hz); MS (*m*/*z*): 294, 296 (*ca* 3:1, M^+·^); Anal. Calcd for C_13_H_8_ClFN_2_O_3_: C, 59.22; H, 2.75; N, 9.51; Found: C, 59.12; H, 2.80; N, 9.43.

#### 3.2.11. *N*-(5-Cyano-2-fluorophenyl)acetamide (**15**)

Yield: 0.65 g (50%) as a tan solid, m.p. 181–182 °C; IR: 3274, 2232, 1668 cm^−1^; ^1^H NMR (400 MHz, DMSO-*d_6_*): δ 10.07 (s, 1H), 8.43 (dd, *J* = 7.3, 2.1 Hz, 1H), 7.66 (ddd, *J* = 8.5, 4.6, 2.1 Hz, 1H), 7.51 (dd, *J* = 10.8, 8.5 Hz, 1H), 2.13 (s, 3H); ^13^C {^1^H} NMR (101 MHz, DMSO-*d_6_*): δ 169.8, 156.0 (d, *J* = 254.5 Hz), 129.7 (d, *J* = 8.9 Hz), 128.2 (d, *J* = 12.7 Hz), 127.4, 118.6, 117.7 (d, *J* = 21.4 Hz), 108.0 (d, *J* = 3.7 Hz), 24.0; MS (*m*/*z*): 178 (M^+·^); Anal. Calcd for C_9_H_7_FN_2_O: C, 60.67; H, 3.96; N, 15.72; Found: C, 60.59; H, 3.99; N, 15.66.

#### 3.2.12. *N*-(5-Cyano-2-fluorophenyl)hexanamide (**16**)

Yield: 0.85 g (50%) as a white solid, m.p. 61–62 °C; IR: 3272, 2234, 1670 cm^−1^; ^1^H NMR (400 MHz, CDCl_3_): δ 8.81 (dd, *J* = 7.3, 2.1 Hz, 1H), 7.41 (br s, 1H), 7.36 (ddd, *J* = 8.5, 4.8, 2.1 Hz, 1H), 7.19 (dd, *J* = 10.5, 8.5 Hz, 1H), 2.43 (t, *J* = 7.5 Hz, 2H), 1.75 (m, 2H), 1.37 (m, 4H), 0.94 (distorted t, *J* = 7.7 Hz, 3H); ^13^C {^1^H} NMR (101 MHz, CDCl_3_): δ 171.6, 154.1 (d, *J* = 251.7 Hz), 128.3 (d, *J* = 8.9 Hz), 127.7 (d, *J* = 10.9 Hz), 125.2 (d, *J* = 2.6 Hz), 118.0, 116.0 (d, *J* = 21.3 Hz), 110.0 (d, *J* = 3.8 Hz), 37.7, 31.3, 25.0, 22.4, 13.9; MS (*m*/*z*): 234 (M^+·^); Anal. Calcd for C_13_H_15_FN_2_O: C, 66.65; H, 6.45; N, 11.96; Found: C, 66.60; H, 6.47; N, 11.88.

#### 3.2.13. *N*-(5-Cyano-2-fluorophenyl)pivalamide (**17**)

Yield: 1.12 g (70%) as a white solid, m.p. 59–61 °C; IR: 3255, 2232, 1660 cm^−1^; ^1^H NMR (400 MHz, CDCl_3_): δ 8.81 (dd, *J* = 7.3, 2.1 Hz, 1H), 7.69 (br s, 1H), 7.37 (ddd, *J* = 8.5, 4.9, 2.1 Hz, 1H), 7.21 (dd, *J* = 10.5, 8.5 Hz, 1H), 1.34 (s, 9H); ^13^C {^1^H} NMR (101 MHz, CDCl_3_): δ 176.9, 154.4 (d, *J* = 251.6 Hz), 128.3 (d, *J* = 8.9 Hz), 127.9 (d, *J* = 10.7 Hz), 125.2 (d, *J* = 2.6 Hz), 117.9, 116.9 (d, *J* = 21.3 Hz), 109.1 (d, *J* = 3.9 Hz), 40.2, 27.4; MS (*m*/*z*): 220 (M^+·^); Anal. Calcd for C_12_H_13_FN_2_O: C, 65.44; H, 5.95; N, 12.72; Found: C, 65.38; H, 5.96; N, 12.65.

#### 3.2.14. *N*-(5-Cyano-2-fluorophenyl)benzamide (**18**)

Yield: 1.05 g (60%) as an off-white solid, m.p. 151–152 °C; IR: 3328, 2232, 1657 cm^−1^; ^1^H NMR (400 MHz, DMSO-*d_6_*): δ 10.38 (s, 1H), 8.18 (dd, *J* = 7.0, 2.1,Hz, 1H), 7.99 (m, 2H), 7.81 (ddd, *J* = 8.6, 4.5, 2.2 Hz, 1H), 7.64 (tt, *J* = 7.4, 1.4 Hz, 1H), 7.60–7.52 (complex, 3H); ^13^C {^1^H} NMR (101 MHz, DMSO-*d_6_*): δ 166.1, 158.6 (d, *J* = 256.8 Hz), 133.9, 132.7, 131.9 (d, *J* = 9.3 Hz), 131.2 (d, *J* = 3.3 Hz), 129.2, 128.4, 127.7 (d, *J* = 133 Hz), 118.4, 118.2 (d, *J* = 21.8 Hz), 108.0 (d, *J* = 3.7 Hz); MS (*m*/*z*): 240 (M^+·^); Anal. Calcd for C_14_H_9_FN_2_O: C, 69.99; H, 3.78; N, 11.66; Found: C, 69.93; H, 3.76; N, 11.60.

#### 3.2.15. *N*-(5-Cyano-2-fluorophenyl)-3-methylbenzamide (**19**)

Yield: 1.11 g (60%) as an off-white solid, m.p. 104–105 °C; IR: 3257, 2236, 1660 cm^−1^; ^1^H NMR (400 MHz, DMSO-*d_6_*): δ 10.33 (s, 1H), 8.16 (dd, *J* = 7.0, 2.1 Hz, 1H), 7.83–7.75 (complex, 3H), 7.57 (dd, *J* = 10.3, 8.5 Hz, 1H), 7.46–7.41 (complex, 2H), 2.40 (s, 3H); ^13^C {^1^H} NMR (101 MHz, DMSO-*d_6_*): δ 165.3, 158.0 (d, *J* = 256.8 Hz,), 137.8, 133.3, 132.7, 131.2 (d, *J* = 9.4 Hz), 130.5 (d, *J* = 3.3 Hz), 128.3 (d, *J* = 5.5 Hz), 127.3 (d, *J* = 13.3 Hz), 125.0, 117.8, 117.6 (d, *J* = 21.7 Hz), 107.4 (d, *J* = 3.7 Hz), 20.8 (one aromatic carbon unresolved); MS (*m*/*z*): 254 (M^+·^); Anal. Calcd for C_15_H_11_FN_2_O: C, 70.86; H, 4.36; N, 11.02; Found: C, 70.88; H, 4.33; N, 10.94.

#### 3.2.16. *N*-(5-Cyano-2-fluorophenyl)-4-methylbenzamide (**20**)

Yield: 1.07 g (58%) as a white solid, m.p. 171–173 °C; IR: 3404, 2232, 1670 cm^−1^; ^1^H NMR (400 MHz, DMSO-*d_6_*): δ 10.29 (s, 1H), 8.17 (dd, *J* = 7.1, 2.2 Hz, 1H), 7.90 (d, *J* = 8.1 Hz, 2H), 7.80 (ddd, *J* = 8.6, 4.5, 2.2 Hz, 1H), 7.56 (dd, *J* = 10.7, 8.6 Hz, 1H), 7.36 (d, *J* = 8.1 Hz, 2H), 2.40 (s, 3H); ^13^C {^1^H} NMR (101 MHz, DMSO-*d_6_*): δ 165.9, 158.6 (d, *J* = 256.6 Hz), 142.8, 131.7 (d, *J* = 9.2 Hz), 131.1 (d, *J* = 3.4 Hz), 131.0, 129.5, 128.4, 127.8 (d, *J* = 13.3 Hz), 118.4, 118.2 (d, *J* = 21.9 Hz), 108.0 (d, *J* = 3.7 Hz), 21.5; MS (*m*/*z*): 254 (M^+·^); Anal. Calcd for C_15_H_11_FN_2_O: C, 70.86; H, 4.36; N, 11.02; Found: C, 70.81; H, 4.32; N, 10.97.

#### 3.2.17. *N*-(5-Cyano-2-fluorophenyl)-4-methoxybenzamide (**21**)

Yield: 1.18 g (60%) as a white solid, m.p. 172–174 °C; IR: 3394, 2842, 2232, 1668 cm^−1^; ^1^H NMR (400 MHz, DMSO-*d_6_*): δ 10.21 (s, 1H), 8.16 (dd, *J* = 7.1, 2.2 Hz, 1H), 7.98 (d, *J* = 8.8 Hz, 2H), 7.79 (ddd, *J* = 8.8, 4.5, 2.1 Hz, 1H), 7.56 (dd, *J* = 10.3, 8.5 Hz, 1H), 7.09 (d, *J* = 8.8 Hz, 2H, 3.85 (s, 3H); ^13^C {^1^H} NMR (101 MHz, DMSO-*d_6_*): δ 165.5, 162.8, 158.5 (d, *J* = 256.7 Hz), 131.6 (d, *J* = 9.1 Hz), 131.1 (d, *J* = 3.6 Hz), 130.4, 127.9 (d, *J* = 13.2 Hz), 125.9, 118.4, 118.2 (d, *J* = 21.9 Hz), 114.2, 108.0 (d, *J* = 3.7 Hz), 56.0; MS (*m*/*z*): 270 (M^+·^); Anal. Calcd for C_15_H_11_FN_2_O_2_: C, 66.66; H, 4.10; N, 10.37; Found: C, 66.59; H, 4.06; N, 10.32.

#### 3.2.18. *N*-(5-Cyano-2-fluorophenyl)-2-fluorobenzamide (**22**)

Yield: 1.46 g (78%) as a tan solid, m.p. 121–122 °C; IR: 3436, 2228, 1687 cm^−1^; ^1^H NMR (400 MHz, DMSO-*d_6_*): δ 10.46 (s, 1H), 8.35 (dd, *J* = 7.1, 2.2 Hz, 1H), 7.79 (ddd, *J* = 8.5, 4.5, 2.0 Hz, 1H), 7.75 (m, 1H), 7.67–7.54 (complex, 2H), 7.41–7.33 (complex, 2H); ^13^C {^1^H} NMR (101 MHz, DMSO-*d_6_*): δ 163.0, 159.1 (d, *J* = 249.8 Hz), 156.8 (d, *J* = 256.3 Hz), 133.3 (d, *J* = 8.5 Hz), 130.9 (d, *J* = 9.1 Hz), 130.3 (d, *J* = 2.5 Hz), 128.8, 126.9 (d, *J* = 13.9 Hz), 124.6 (d, *J* = 3.5 Hz), 123.2 (d, *J* = 14.1 Hz), 117.8, 117.5 (d, *J* = 21.5 Hz), 116.2 (d, *J* = 21.9 Hz), 107.6 (d, *J* = 3.9 Hz); ^19^F {^1^H} NMR (376 MHz, DMSO-*d_6_* referenced to C_6_H_5_F): δ –113.0, –113.74; MS (*m*/*z*): 258 (M^+·^); Anal. Calcd for C_14_H_8_F_2_N_2_O_2_: C, 65.12; H, 3.12; N, 10.85; Found: C, 65.03; H, 3.15; N, 10.77.

#### 3.2.19. 3-Chloro-*N*-(5-cyano-2-fluorophenyl)benzamide (**23**)

Yield: 1.44 g (72%) as a white solid, m.p. 144–145 °C; IR: 3298, 2234, 1685 cm^−1^; ^1^H NMR (400 MHz, DMSO-*d_6_*): δ 10.51 (s, 1H), 8.16 (dd, *J* = 7.1, 2.2 Hz, 1H), 8.01 (t, *J* = 1.9 Hz, 1H), 7.92 (dt, *J* = 7.8, 1.3 Hz, 1H), 7.82 (m, 1H), 7.70 (br d, *J* = 8.0 Hz, 1H), 7.62–7.53 (complex, 2H); ^13^C {^1^H} NMR (101 MHz, DMSO-*d_6_*): δ 164.3, 158.1 (d, *J* = 256.4 Hz), 135.4, 133.3, 132.0, 131.6 (d, *J* = 9.1 Hz), 130.7 (d, *J* = 3.7 Hz), 130.6, 127.7, 126.9 (d, *J* = 13.3 Hz), 126.7, 117.8, 117.7 (d, *J* = 22.1 Hz), 107.6 (d, *J* = 3.7 Hz); MS (*m*/*z*): 274, 276 (ca 3:1, M^+·^); Anal. Calcd for C_14_H_8_ClFN_2_O: C, 61.22; H, 2.94; N, 10.20; Found: C, 61.17; H, 2.98; N, 10.13.

#### 3.2.20. 4-Chloro-*N*-(5-cyano-2-fluorophenyl)benzamide (**24**)

Yield: 1.00 g (50%) as a tan solid, m.p. 184–185 °C; IR: 3409, 2232, 1680 cm^−1^; ^1^H NMR (400 MHz, DMSO-*d_6_*): δ 10.47 (s, 1H), 8.18 (dd, *J* = 7.0, 2.1 Hz, 1H), 8.01 (d, *J* = 8.6 Hz, 2H), 7.82 (ddd, *J* = 8.6, 4.5, 2.2 Hz, 1H), 7.64 (d, *J* = 8.6 Hz, 2H), 7.58 (dd, *J* = 10.5, 8.6 Hz, 1H); ^13^C {^1^H} NMR (101 MHz, DMSO-*d_6_*): δ 165.1, 158.6 (d, *J* = 256.8 Hz), 137.5, 132.6, 132.0 (d, *J* = 9.2 Hz), 131.1 (d, *J* = 3.2 Hz), 130.3, 129.1 127.5 (d, *J* = 13.3 Hz), 118.3, 118.2 (d, *J* = 22.2 Hz), 108.1 (d, *J* = 3.6 Hz); MS (*m*/*z*): 274, 276 (ca 3:1, M^+·^); Anal. Calcd for C_14_H_8_ClFN_2_O: C, 61.22; H, 2.94; N, 10.20; Found: C, 61.19; H, 3.01; N, 10.15.

#### 3.2.21. Methyl 3-benzamido-4-fluorobenzoate (**25**)

Yield: 0.84 g (52%) as a gray solid, m.p. 132–133 °C; IR: 3281, 1720, 1651 cm^−1^; ^1^H NMR (400 MHz, CDCl_3_): δ 9.15 (dd, *J* = 7.7, 2.2 Hz, 1H), 8.07 (br s, 1H), 7.92 (m, 2H), 7.85 (ddd, *J* = 8.6, 5.1, 2.2 Hz, 1H), 7.60 (m, 1H), 7.53 (m, 2H), 7.21 (dd, *J* = 10.5, 8.5 Hz, 1H), 3.93 (s, 3H); ^13^C {^1^H} NMR (101 MHz, CDCl_3_): δ 166.1, 165.4, 155.5 (d, *J* = 250.3 Hz), 134.1, 132.4, 129.0, 127.13, 127.10 (obscured d, *J* = ca 3.3 Hz), 126.6 (d, *J* = 8.6 Hz), 126.5 (d, *J* = 9.5 Hz), 123.4 (d, *J* = 2.5 Hz), 115.0 (d, *J* = 20.4 Hz), 52. 4; MS (*m*/*z*): 273 (M^+·^); Anal. Calcd for C_15_H_12_FNO_3_: C, 65.93; H, 4.43; N, 5.13; Found: C, 65.88; H, 4.39; N, 5.08.

#### 3.2.22. Methyl 4-fluoro-3-(3-methylbenzamido)benzoate (**26**)

Yield: 1.14 g (68%) as a gray solid, m.p. 111–112 °C; IR: 3281, 1710, 1651 cm^−1^; ^1^H NMR (400 MHz, CDCl_3_): δ 9.10 (dd, *J* =- 7.7, 2.2 Hz, 1H), 8.11 (br s, 1H), 7.83 (ddd, *J* = 8.6, 5.1, 2.2 Hz, 1H), 7.72 (br s, 1H), 7.68 (m, 1H), 7.39 (m, 2H), 7.18 (dd, *J* = 10.4, 8.6 Hz, 1H), 3.91 (s, 3H), 2.44 (s, 3H); ^13^C {^1^H} NMR (101 MHz, CDCl_3_): δ 166.1, 165.7, 155.5 (d, *J* = 250.5 Hz), 138.9, 134.1, 133.1, 128.8, 127.9, 127.0 (d, J = 3.3 Hz), 126.6 (d, *J* = 8.7 Hz, 126.5 (d, *J* = 11.1 Hz), 124.1, 123.5 (d, *J* = 2.5 Hz), 114.9 (d, *J* = 20.5 Hz), 52.3, 21.4; MS (*m*/*z*): 287 (M^+·^); Anal. Calcd for C_16_H_14_FNO_3_: C, 66.89; H, 4.91; N, 4.88; Found: C, 66.86; H, 4.95; N, 4.87.

#### 3.2.23. Methyl 4-fluoro-3-(4-methylbenzamido)benzoate (**27**) 

Yield: 1.26 g (71%) as a gray solid, m.p. 143–144 °C; IR: 3289, 1721, 1656 cm^−1^; ^1^H NMR (400 MHz, CDCl_3_): δ 9.13 (dd, *J* = 7.7, 2.2 Hz, 1H), 8.07 (br s, 1H), 7.83 (ddd, *J* = 8.6, 5.1, 2.2 Hz, 1H), 7.81 (d, *J* = 8.4 Hz, 2H), 7.32 (d, *J* = 8.4 Hz, 2H), 7.19 (dd, *J* = 10.5, 8.6 Hz, 1H), 3.92 (s, 3H), 2.44 (s, 3H); ^13^C {^1^H} NMR (101 MHz, CDCl_3_): δ 166.1, 165.4, 155.4 (d, *J* = 250.2 Hz), 143.1, 131.3, 129.6, 127.2, 127.0 (d, *J* = 3.2 Hz, 1H), 126.6 (d, *J* = 10.6 Hz), 126.4 (d, *J* = 8.9 Hz), 123.4 (d, *J* = 2.5 Hz), 115.0 (d, *J* = 20.5 Hz), 52.3, 21.6; MS (*m*/*z*): 287 (M^+·^); Anal. Calcd for C_16_H_14_FNO_3_: C, 66.89; H, 4.91; N, 4.88; Found: C, 66.82; H, 4.87; N, 4.81.

#### 3.2.24. Methyl 4-fluoro-3-(4-methoxybenzamido)benzoate (**28**)

Yield: 1.27 g (72%) as a gray solid, m.p. 144–145 °C; IR: 3295, 2851, 1715, 1655 cm^−1^; ^1^H NMR (400 MHz, CDCl_3_): δ 9.11 (dt, *J* = 7.7, 2.0 Hz, 1H), 8.02 (br s, 1H), 7.88 (d, *J* = 8.7 Hz, 2H), 7.82 (m, 1H), 7.18 (dd, *J* = 10.2, 8.6 Hz, 1H), 6.99 (d, *J* = 8.7 Hz, 2H), 3.92 (s, 3H), 3.88 (s, 3H); ^13^C {^1^H} NMR (101 MHz, CDCl_3_): δ 166.1, 164.9, 162.9, 155.4 (d, J = 250.1 Hz), 129.1, 127.0 (d, *J* = 3.2 Hz), 126.7 (d, *J* = 10.6 Hz), 126.32 (d, *J* = 8.9 Hz), 126.30, 123.4 (d, *J* = 2.5 Hz), 114.9 (d, *J* = 20.5 Hz), 114.5, 55.5, 52.3; MS (*m*/*z*): 303 (M^+·^); Anal. Calcd for C_16_H_14_FNO_4_: C, 63.36; H, 4.65; N, 4.62; Found: C, 63.31; H, 4.69; N, 4.57.

#### 3.2.25. Methyl 4-fluoro-3-(2-fluorobenzamido)benzoate (**29**)

Yield: 1.08 g (63%) as a gray solid, m.p. 98–99 °C; IR: 3376, 3328, 1716, 1650 cm^−1^; ^1^H NMR (400 MHz, CDCl_3_): δ 9.16 (dd, *J* = 7.6, 2.2 Hz, 1H), 8.83 (2 br s, 1H), 8.21 (td, *J* = 7.9, 1,9 Hz, 1H), 7.85 (ddd, *J* = 8.6, 5.1, 2.2 Hz, 1H), 7.59–7.53 (complex, 1H), 7.34 (td, *J* = 7.6, 1.1 Hz, 1H), 7.24–7.18 (complex, 2H), 3.93 (s, 3H); ^13^C {^1^H} NMR (101 MHz, CDCl_3_): δ 166.1, 161.3 (d, *J* = 3.7 Hz), 160.5 (d, *J* = 247.1 Hz), 155.4 (d, *J* = 251.3 Hz), 134.0 (d, *J* = 9.5 Hz), 132.3 (d, *J* = 1.7 Hz), 127.0 (d, *J* = 3.3 Hz), 126.8 (d, *J* = 8.9 Hz), 126.4 (d, *J* = 10.6 Hz), 125.2 (d, *J* = 3.2 Hz), 123.7 (d, *J* = 2.4 Hz), 120.6 (d, *J* = 10.9 Hz), 116.3 (d, *J* = 25.1 Hz), 115.0 (d, *J* = 20.4 Hz), 52.3; ^19^F {^1^H} NMR (376 MHz, DMSO-*d_6_* referenced to C_6_H_5_F): δ –113.98, –115.58; MS (*m*/*z*): 291 (M^+·^); Anal. Calcd for C_15_H_11_F_2_NO_3_: C, 61.86; H, 3.81; N, 4.81; Found: C, 61.78; H, 3.79; N, 4.75.

#### 3.2.26. Methyl 3-(3-chlorobenzamido)-4-fluorobenzoate (**30**)

Yield: 1.23 g (68%) as a gray solid, m.p. 134–135 °C; IR: 3251, 1719, 1658 cm^−1^; ^1^H NMR (400 MHz, CDCl_3_): δ 9.08 (dd, *J* = 7.6, 2.2 Hz, 1H), 8.03 (br s, 1H), 7.89 (t, *J* = 1.9 Hz, 1H), 7.86 (ddd, *J* = 8.6, 5.1, 2.2 Hz, 1H), 7.77 (ddd, *J* = 7.7, 1.8, 1.1 Hz, 1H), 7.57 (ddd, *J* = 8.0, 2.2, 1.1 Hz, 1H), 7.46 (t, *J* = 7.8 Hz, 1H), 7.21 (dd, *J* = 10.4, 8.6 Hz, 1H), 3.93 (s, 3H); ^13^C {^1^H} NMR (101 MHz, CDCl_3_): δ 166.0, 164.1, 155.5 (d, *J* = 250.9 Hz), 135.9, 135.2, 132.4, 130.3, 127.6, 127.1 (d, *J* = 3.3 Hz), 127.0 (d, *J* = 8.9 Hz), 126.2 (d, *J* = 10.6 Hz), 125.1, 123.6 (d, *J* = 2.2 Hz), 115.1 (d, *J* = 20.2 Hz), 52.3; MS (*m*/*z*): 307, 309 (ca. 3:1, M^+·^); Anal. Calcd for C_15_H_11_ClFNO_3_: C, 58.55; H, 3.60; N, 4.55; Found: C, 58.56; H, 3.58; N, 4.43.

#### 3.2.27. Methyl 3-(4-chlorobenzamido)-4-chlorobenzoate (**31**)

Yield: 1.23 g (68%) as a gray solid, m.p. 150–151 °C; IR: 3251, 1711, 1658 cm^−1^; ^1^H NMR (400 MHz, CDCl_3_): δ 9.10 (dd, *J* = 7.6, 1.7 Hz, 1H), 8.02 (br s, 1H), 7.86 (obscured ddd, 1H), 7.84 (d, *J* = 8.6 Hz, 2H), 7.51 (d, *J* = 8.6 Hz, 2H), 7.21 (dd, *J* = 10.4, 8.6 Hz, 1H), 3.93 (s, 3H); ^13^C {^1^H} NMR (101 MHz, CDCl_3_): δ 166.0, 164.4, 155.4 (d, *J* = 250.5 Hz), 138.8, 132.5, 129.3, 128.6, 127.1 (d, *J* = 3.2 Hz), 126.9 (d, *J* = 8.9 Hz), 126.3 (d, *J* = 10.6 Hz), 123.5 (d, *J* = 2.4 Hz), 115.1 (d, *J* = 20.4 Hz), 52.4; MS (*m*/*z*): 307, 309 (ca. 3:1, M^+·^); Anal. Calcd for C_15_H_11_ClFNO_3_: C, 58.55; H, 3.60; N, 4.55; Found: C, 58.51; H, 3.63; N, 4.52.

#### 3.2.28. *N*-(2-Fluoro-5-trifluoromethyl)phenyl)benzamide (**32**)

Yield: 1.08 g (69%) as a white solid, m.p. 123–124 °C; IR: 3267, 1658, 1104 cm^−1^; ^1^H NMR (400 MHz, DMSO-*d_6_*): δ 10.38 (s, 1H), 8.12 (dd, *J* = 7.0, 2.4 Hz, 1H), 8.00 (d, *J* = 7.2 Hz, 2H), 7.70–7.61 (complex, 2H), 7.61–7.53 (complex, 3H); ^13^C {^1^H} NMR (101 MHz, DMSO-*d_6_*): δ 166.2, 157.9 (d, *J* = 253.8 Hz), 134.0, 132.6, 129.0, 128.4, 127.4 (d, *J* = 13.1 Hz), 125.7 (dq, *J* = 32.6, 3.5 Hz), 124.3 (m), 124.2 (q, *J* = 272.0 Hz), 124.1 (m), 117.6 (d, *J* = 21.5 Hz); ^19^F {^1^H} NMR (376 MHz, CDCl_3_ referenced to C_6_H_5_F): δ –62.15 (d, *J* = 2.1 Hz), –126.02 (q, *J* = 1.9 Hz); MS (*m*/*z*): 283 (M^+·^); Anal. Calcd for C_14_H_9_F_4_NO: C, 59.37; H, 3.20; N, 4.95; Found: C, 59.27; H, 3.17; N, 4.88.

#### 3.2.29. *N*-(2-Fluoro-5-(trifluoromethyl)phenyl)-3-methylbenzamide (**33**)

Yield: 1.28 g (78%) as a white solid, m.p. 93–94 °C; IR: 3296, 1653, 1125 cm^−1^; ^1^H NMR (400 MHz, CDCl_3_): δ 8.89 (dd, *J* = 7.2, 2.3 Hz, 1H), 8.13 (br s, 1H), 7.71 (s, 1H), 7.67 (m, 1H), 7.41 (m, 2H), 7.37 (ddd, *J* = obscured, 5.1, 2.3 Hz, 1H), 7.24 (dd, *J* = 10.4, 8.4 Hz, 1H), 2.45 (s, 3H); ^13^C {^1^H} NMR (101 MHz, CDCl_3_): δ 165.7, 154.0 (d, *J* = 248.8 Hz), 139.0, 133.9, 133.1, 128.9, 127.8, 127.4 (dq, *J* = 33.1, 3.6 Hz), 127.3 (d, *J* = 10.5 Hz), 124.1, 123.6 (q, *J* = 272.2 Hz), 121.5 (dq, *J* = 8.1, 4.0 Hz), 119.1 (m), 115.3 (d, *J* = 20.8 Hz), 21.4; ^19^F {^1^H} NMR (376 MHz, CDCl_3_ referenced to C_6_H_5_F): δ –62.16 (d, *J* = 1.7 Hz), –126.31 (q, *J* = 1.7 Hz); MS (*m*/*z*): 297 (M^+·^); Anal. Calcd for C_15_H_11_F_4_NO: C, 60.61; H, 3.73; N, 4.71; Found: C, 60.55; H, 3.70; N, 4.66.

#### 3.2.30. *N*-(2-Fluoro-5-(trifluoromethyl)phenyl)-4-methylbenzamide (**34**)

Yield: 1.17 g (71%) as a white solid, m.p. 90–91 °C; IR: 3336, 1658, 1123 cm^−1^; ^1^H NMR (400 MHz, CDCl_3_): δ 8.89 (dd, *J* = 7.3, 2.3 Hz, 1H), 8.12 (br s, 1H), 7.79 (d, *J* = 8.2 Hz, 2H), 7.37 (dddd, *J* = 8.6, 4.8, 2.3, 0.8 Hz, 1H), 7.32 (d, *J* = 8.2 Hz, 2H), 7.23 (dd, *J* = 10.5, 8.6 Hz, 1H), 2.44 (s, 3H); ^13^C {^1^H} NMR (101 MHz, CDCl_3_): δ 165.4, 153.9 (d, *J* = 247.7 Hz), 143.3, 131.1, 129.7, 127.4 (dq, *J* = 33.8, 3.4 Hz), 127.3 (d, *J* = 10.5 Hz), 127.1, 123.6 (q, *J* = 272.2 Hz), 122.4 (dq, *J* = 8.6, 4.0 Hz), 119.1 (m), 115.2 (d, *J* = 20.8 Hz), 21.6; ^19^F {^1^H} NMR (376 MHz, CDCl_3_ referenced to C_6_H_5_F): δ –62.16 (d, *J* = 2.1 Hz), –126.45 (q, *J* = 2.1 Hz); MS (*m*/*z*): 297 (M^+·^); Anal. Calcd for C_15_H_11_F_4_NO: C, 60.61; H, 3.73; N, 4.71; Found: C, 60.58; H, 3.74; N, 4.63.

#### 3.2.31. *N*-(2-Fluoro-5-(trifluoromethyl)phenyl)-4-methoxybenzamide (**35**)

Yield: 1.16 g (67%) as a white solid, m.p. 105–107 °C; IR: 3300, 2848, 1652, 1111 cm^−1^; ^1^H NMR (400 MHz, CDCl_3_): δ 8.88 (dd, *J* = 7.2, 2.3 Hz, 1H), 8.06 (br s, 1H), 7.87 (d, *J* = 8.8 Hz, 2H), 7.36 (ddd, *J* = 8.7, 4.8, 2.3 Hz, 1H), 7.23 (dd, *J* = 10.5, 8.6 Hz, 1H), 7.01 (d, *J* = 8.8 Hz, 2H), 3.89 (s, 3H); ^13^C {^1^H} NMR (101 MHz, CDCl_3_): δ 165.0, 163.0, 154.0 (d, *J* = 247.5 Hz), 129.1, 127.4 (d, *J* = 10.6 Hz), 127.4 (dq, *J* = 29.6, 3.5 Hz), 126.1, 123.6 (q, *J* = 272.4 Hz), 121.2 (dq, *J* = 8.4, 4.0 Hz), 119.0 (m), 115.2 (d, *J* = 20.8 Hz), 114.2, 55.6; ^19^F {^1^H} NMR (376 MHz, CDCl_3_ referenced to C_6_H_5_F): δ –62.16 (d, *J* = 2.1 Hz), –126.02 (q, *J* = 2.0 Hz); MS (*m*/*z*): 313 (M^+·^); Anal. Calcd for C_15_H_11_F_4_NO_2_: C, 57.51; H, 3.54; N, 4.47; Found: C, 57.44; H, 3.53; N, 4.41.

#### 3.2.32. 2-Fluoro-*N*-(2-fluoro-5-(trifluoromethyl)phenyl)benzamide (**36**)

Yield: 1.33 g (80%) as a white solid, m.p. 92–94 °C; IR: 3444, 1650, 1118 cm^−1^; ^1^H NMR (400 MHz, CDCl_3_): δ 8.92 (dd, *J* = 7.1, 2.3 Hz, 1H), 8.88 (br s, 1H), 8.20 (td, *J* = 8.0, 1.9 Hz, 1H), 7.57 (m, 1H), 7.42–7.35 (complex, 2H), 7.26–7.18 (complex, 2H); ^13^C {^1^H} NMR (101 MHz, CDCl_3_): δ 161.4 (d, *J* = 3.8 Hz), 160.6 (d, *J* = 247.1 Hz), 154.0 (d, *J* = 249.6 Hz), 134.5 (d, *J* = 9.6 Hz), 132.3 (d, *J* = 1.7 Hz), 127.4 (dq, *J* = 33.0, 3.6 Hz), 127.1 (d, *J* = 10.8 Hz), 125.3 (d, *J* = 3.3 Hz), 123.6 (q, *J* = 272.2 Hz), 121.7 (dq, *J* = 8.0, 4.1 Hz), 120.4 (d, *J* = 10.9 Hz), 119.4 (m), 116.4 (d, *J* = 25.0 Hz), 115.3 (d, *J* = 20.7 Hz); ^19^F {^1^H} NMR (376 MHz, CDCl_3_ referenced to C_6_H_5_F): δ –62.15 (d, *J* = 2.1 Hz), –112.93, –125.74 (q, *J* = 1.9 Hz); MS (*m*/*z*): 301 (M^+·^); Anal. Calcd for C_14_H_8_F_5_NO: C, 55.83; H, 2.68; N, 4.65; Found: C, 55.86; H, 2.71; N, 4.58.

#### 3.2.33. 3-Chloro-*N*-(2-fluoro-5-(trifluoromethyl)phenyl)benzamide (**37**)

Yield: 1.32 g (75%) as a white solid, m.p. 119–121 °C; IR: 3234, 1656, 1123 cm^−1^; ^1^H NMR (400 MHz, CDCl_3_): δ 8.79 (dd, *J* = 7.2, 2.3 Hz, 1H), 8.14 (br s, 1H), 7.86 (t, *J* = 1.9 Hz, 1H), 7.74 (dt, *J* = 7.8, 1.4 Hz, 1H), 7.55 (ddd, *J* = 8.0, 2.1, 1.1 Hz, 1H), 7.44 (t, *J* = 7.8 Hz, 1H), 7.39 (ddd, *J* = 8.0,4.8, 2.1 Hz, 1H), 7.24 (dd, *J* = 10.6, 8.8 Hz, 1H); ^13^C {^1^H} NMR (101 MHz, CDCl_3_): δ 164.2, 154.2 (d, *J* = 248.9 Hz), 135.6, 135.3, 132.6, 130.3, 127.6, 127.4 (dq, *J* = 33.1, 3.5 Hz), 126.8 (d, *J* = 10.8 Hz), 125.1, 123.6 (q, *J* = 272.3 Hz), 122.1 (dq, *J* = 8.0, 3.9 Hz), 119.3 (m), 115.4 (d, *J* = 20.7 Hz); ^19^F {^1^H} NMR (376 MHz, DMSO-*d_6_* referenced to C_6_H_5_F): δ –60.70 (d = *J* = 1.7 Hz), 114.78 (q, *J* = 1.7 Hz); MS (*m*/*z*): 317, 319 (ca. 3:1, M^+·^); Anal. Calcd for C_14_H_8_ClF_4_NO: C, 52.93; H, 2.54; N, 4.41; Found: C, 52.89; H, 2.55; N, 4.39.

#### 3.2.34. 4-Chloro-*N*-(2-fluoro-5-(trifluoromethyl)phenyl)benzamide (**38**)

Yield: 1.25 g (71%) as a white solid, m.p. 97–98 °C; IR: 3297, 1656, 1120 cm^−1^; ^1^H NMR (400 MHz, CDCl_3_): δ 8.85 (dd, *J* = 7.2, 2.3 Hz, 1H), 8.07 (br s, 1H), 7.84 (d, *J* = 8.5 Hz, 2H), 7.51 (d, *J* = 8.5 Hz, 2H), 7.40 (ddd, *J* = 8.2, 4.8, 2.2 Hz, 1H), 7.25 (t, *J* = 8.9 Hz, 1H); ^13^C {^1^H} NMR (101 MHz, CDCl_3_): δ 164.4, 154.0 (d, *J* = 248.6 Hz), 139.0, 132.3, 129.4, 128.6, 128.3 (dq, *J* = 29.7, 3.6 Hz), 127.0 (d, *J* = 10.5 Hz), 123.6 (d, *J* = 172.3 Hz), 121.9 (dq, *J* = 8.7, 4.0 Hz), 119.2 (m), 115.4 (d, *J* = 20.8 Hz); ^19^F {^1^H} NMR (376 MHz, CDCl_3_ referenced to C_6_H_5_F): δ –62.20 (d, *J* = 2.1 Hz), –126.25 (q, *J* = 2.1 Hz); MS (*m*/*z*): 317, 319 (ca. 3:1, M^+·^); Anal. Calcd for C_14_H_8_ClF_4_NO: C, 52.93; H, 2.54; N, 4.41; Found: C, 52.94; H, 2.56; N, 4.36.

### 3.3. Representative Predure for Cyclizing Benzo[d]oxazole Derivatives

In a 50 mL round-bottomed flask equipped with a magnetic stirrer and a N_2_ inlet, the anilide (100 mg, 1 equiv.) was dissolved in 4 mL of dry *N,N*-dimethylformamide. To this solution, 2 equiv. of anhydrous K_2_CO_3_ was added, and the reaction was heated at 90 °C for 1 h (NO_2_ activation), 115 °C for 1 h (CN activation), 120 °C for 2 h (CO_2_Me activation), or 130 °C for 3 h (CF_3_ activation). The reaction was cooled, added to saturated aqueous NaCl (25 mL) and extracted with ethyl acetate (3 × 25 mL). The combined organic extracts were washed with saturated aqueous NaCl (3 × 25 mL), dried (Na_2_SO_4_), and concentrated under vacuum. Two methods were used to purify the products: *Method A*: The crude product obtained after concentration was crystallized from 1 to 5% ethyl acetate in petroleum ether to give the pure product. *Method B*: The crude product was concentrated in the presence of 1–2 g of silica gel, added to a 25 cm × 1 cm silica gel column, and eluted with 5–25% diethyl ether in petroleum ether. Upon removal of the solvent under vacuum, band 1 gave the benzo[*d*]oxazole. Method B generally gave cleaner products and also allowed for recovery of unreacted starting material, which eluted with 30–50% ether in petroleum ether. All cyclizations were performed using 100 mg of the corresponding anilide with other reagents scaled accordingly. 

#### 3.3.1. 2-Methyl-5-nitrobenzo[*d*]oxazole (**39**)

Yield: 46 mg (51%) as a white solid, m.p. 152–154 °C (lit. [33] m.p. 154.7–155.5 °C^)^; Purification method B; IR: 1617, 1521, 1349 cm^−1^; ^1^H NMR (400 MHz, CDCl_3_): δ 8.55 (d, *J* = 2.3 Hz, 1H), 8.29 (dd, *J* = 8.9, 2.3 Hz, 1H), 7.58 (d, *J* = 8.9 Hz, 1H), 2.71 (s, 3H); ^13^C {^1^H} NMR (101 MHz, CDCl_3_): δ 167.1, 154.5, 145.2, 142.0, 120.8, 115.9, 110.4, 14.7; MS: *m*/*z* 178 (M^+·^).

#### 3.3.2. 5-Nitro-2-pentylbenzo[*d*]oxazole (**40**)

Yield: 65 mg (74%) as a light yellow oil; Purification method B; IR: 1617, 1525, 1353 cm^−1^; ^1^H NMR (400 MHz, CDCl_3_): δ 8.56 (d, *J* = 2.3 Hz, 1H), 8.29 (dd, *J* = 8.9, 2.3 Hz, 1H), 7.59 (d, *J* = 8.9 Hz, 1H), 2.98 (d, *J* = 7.6 Hz, 2H), 1.92 (pentet, *J* = 7.6 Hz, 2H), 1.42 (m, 4H), 0.92 (t, *J* = 7.6 Hz, 3H); ^13^C {^1^H} NMR (101 MHz, CDCl_3_): δ 170.7, 154.4, 145.1, 141.9, 120.7, 116.0, 110.4, 31.2, 28.7, 26.2, 22.3, 13.9; MS: *m*/*z* 234 (M^+·^); Anal. Calcd for C_12_H_14_N_2_O_3_: C, 61.53; H, 6.02; N, 11.96; Found: C, 61.48; H, 5.99; N, 11.87.

#### 3.3.3. 2-(tert-Butyl)-5-nitrobenzo[*d*]oxazole (**41**)

Yield: 77 mg (84%) as a tan solid, m.p. 161–162 °C; Purification method A; IR: 1606, 1521, 1340 cm^−1^; ^1^H NMR (400 MHz, CDCl_3_): δ 8.58 (d, *J* = 2.3 Hz, 1H), 8.28 (dd, *J* = 8.9, 2.3 Hz, 1H), 7.59 (d, *J* = 8.9 Hz, 1H), 1.52 (s, 9H); ^13^C {^1^H} NMR (101 MHz, CDCl_3_): δ 176.8, 154.5, 145.1, 141.7, 120.7, 116.2, 110.5, 34.5, 28.3; MS: *m*/*z* 220 (M^+·^); Anal. Calcd for C_11_H_12_N_2_O_3_: C, 59.99; H, 5.49; N, 12.72; Found: C, 59.95; H, 5.46; N, 12.66.

#### 3.3.4. 5-Nitro-2-phenylbenzo[*d*]oxazole (**42**)

Yield: 78 mg (84%) as a tan solid, m.p. 171–173 °C (lit. [34] m.p. 171.5–172.5 °C^)^; Purification method A; IR: 1626, 1522, 1352 cm^−1^; ^1^H NMR (400 MHz, CDCl_3_): δ 8.65 (d, *J* = 2.3 Hz, 1H), 8.32 (dd, *J* = 8.9, 2.3 Hz, 1H), 8.28 (m, 1H), 8.26 (m, 1H), 7.68 (d, *J* = 8.9 Hz, 1H), 7.64–7.54 (complex, 3H); ^13^C {^1^H} NMR (101 MHz, CDCl_3_): δ 166.0, 154.3, 145.5, 142.6, 132.6, 129.2, 128.1, 126.0, 121.2, 116.3, 110.8; MS: *m*/*z* 240 (M^+·^).

#### 3.3.5. 2-(3-Methylphenyl)-5-nitrobenzo[*d*]oxazole (**43**)

Yield: 76 mg (82%) as a tan solid, m.p. 155–156 °C; Purification method A; IR: 1617, 1527, 1353 cm^−1^; ^1^H NMR (400 MHz, CDCl_3_): δ 8.65 (d, *J* = 2.3 Hz, 1H), 8.33 (dd, *J* = 8.9, 2.3 Hz, 1H), 8.11 (br d, 1H), 8.07 (d, *J* = 7.3 Hz, 1H), 7.68 (d, *J* = 8.9 Hz, 1H), 7.48–7.41 (complex, 2H), 2.48 (s, 3H); ^13^C {^1^H} NMR (101 MHz, CDCl_3_): δ 166.3, 154.3, 145.5, 142.6, 139.1, 133.5, 129.1, 128.6, 125.9, 125.2, 121.1, 116.2, 110.7, 21.4; MS: *m*/*z* 254 (M^+·^); Anal. Calcd for C_14_H_10_N_2_O_3_: C, 66.14; H, 3.96; N, 11.02; Found: C, 66.08; H, 3.93; N, 10.95.

#### 3.3.6. 2-(4-Methylphenyl)-5-nitrobenzo[*d*]oxazole (**44**)

Yield: 82 mg (88%) as a tan solid, m.p. 168–169 °C; Purification method A; IR: 1625, 1524, 1352 cm^−1^; ^1^H NMR (400 MHz, CDCl_3_): δ 8.64 (d, *J* = 2.3 Hz, 1H), 8.31 (dd, *J* = 8.9, 2.3 Hz, 1H), 8.16 (d, *J* = 8.3 Hz, 2H), 7.67 (d, *J* = 8.9 Hz, 1H), 7.38 (d, *J* = 8.3 Hz, 2H), 2.47 (s, 3H); ^13^C {^1^H} NMR (101 MHz, CDCl_3_): δ 166.3, 154.3, 145.4, 143.5, 142.7, 129.9, 128.1, 123.2, 121.0, 116.1, 110.6, 21.8; MS: *m*/*z* 254 (M^+·^); Anal. Calcd for C_14_H_10_N_2_O_3_: C, 66.14; H, 3.96; N, 11.02; Found: C, 66.11; H, 3.94; N, 10.98.

#### 3.3.7. 2-(4-Methoxyphenyl)-5-nitrobenzo[*d*]oxazole (**45**)

Yield: 80 mg (86%) as a tan solid, m.p. 184–185 °C (lit. [34] m.p. 184–186 °C^)^; Purification method A; IR: 2840, 1521, 1340 cm^−1^; ^1^H NMR (400 MHz, CDCl_3_): δ 8.61 (d, *J* = 2.3 Hz, 1H), 8.29 (dd, *J* = 8.9, 2.3 Hz, 1H), 8.21 (d, *J* = 8.9 Hz, 2H), 7.64 (d, *J* = 8.9 Hz, 1H), 7.07 (d, *J* = 8.9 Hz, 2H), 3.92 (s, 3H); ^13^C {^1^H} NMR (101 MHz, CDCl_3_): δ 166.2, 163.2, 154.3, 145.4, 142.9, 130.0, 120.7, 118.4, 115.8, 114.6, 110.4, 55.6; MS: *m*/*z* 270 (M^+·^).

#### 3.3.8. 2-(2-Fluorophenyl)-5-nitrobenzo[*d*]oxazole (**46**)

Yield: 82 mg (88%) as a tan solid, m.p. 172–173 °C; Purification method A; IR: 1617, 1521, 1340 cm^−1^; ^1^H NMR (400 MHz, CDCl_3_): δ 8.72 (d, *J* = 2.3 Hz, 1H), 8.36 (dd, *J* = 8.9, 2.3 Hz, 1H), 8.27 (td, *J* = 7.5, 1.8 Hz, 1H), 7.73 (d, *J* = 8.9 Hz, 1H), 7.61 (m, 1H), 7.37 (td, *J* = 7.6, 1.1 Hz, 1H), 7.32 (ddd, *J* = 10.9, 8.3, 1.1 Hz, 1H); ^13^C {^1^H} NMR (101 MHz, CDCl_3_): δ 163.5, 161.1 (d, *J* = 260.5 Hz), 154.0, 145.6, 142.2, 134.3 (d, *J* = 8.8 Hz), 130.8, 124.8 (d, *J* = 3.8 Hz), 121.5, 117.3 (d, *J* = 21.3 Hz), 116.7, 114.5, 111.0; MS: *m*/*z* 258 (M^+·^); Anal. Calcd for C_13_H_7_FN_2_O_3_: C, 60.47; H, 2.73; N, 7.36; Found: C, 60.41; H, 2.70; N, 7.32.

#### 3.3.9. 2-(3-Chlorophenyl)-5-nitrobenzo[*d*]oxazole (**47**)

Yield: 63 mg (68%) as a tan solid, m.p. 149–151 °C; Purification method A; IR: 1617, 1522, 1351 cm^−1^; ^1^H NMR (400 MHz, CDCl_3_): δ 8.67 (d, *J* = 2.3 Hz, 1H), 8.36 (dd, *J* = 8.9, 2.3 Hz, 1H), 8.28 (t, *J* = 1.9 Hz, 1H), 8.17 (dt, *J* = 7.7, 1.4 Hz, 1H), 7.71 (d, *J* = 8.9 Hz, 1H), 7.59 (ddd, *J* = 8.1, 1.9, 1.4 Hz, 1H), 7.51 (t, *J* = 7.8 Hz, 1H); ^13^C {^1^H} NMR (101 MHz, CDCl_3_): δ 164.6, 154.3, 145.6, 142.4, 135.4, 132.6, 130.5, 128.0, 127.7, 126.1, 121.,5, 116.6, 110.9; MS: *m*/*z* 274, 276 (*ca* 3:1, M^+·^); Anal. Calcd for C_13_H_7_ClN_2_O_3_: C, 56.85; H, 2.57; N, 10.20; Found: C, 56.77; H, 2.54; N, 10.12.

#### 3.3.10. 2-(4-Chlorophenyl)-5-nitrobenzo[*d*]oxazole (**48**)

Yield: 71 mg (76%) as a tan solid, m.p. 217–218 °C (lit. [34] m.p. 218–219 °C^)^; Purification method A; IR: 1617, 1524, 1351 cm^−1^; ^1^H NMR (400 MHz, CDCl_3_): δ 8.66 (d, *J* = 2.3 Hz, 1H), 8.34 (dd, *J* = 8.9, 2.3 Hz, 1H), 8.22 (d, *J* = 8.6 Hz, 2H), 7.69 (d, *J* = 8.9 Hz, 1H), 7.56 (d, *J* = 8.6 Hz, 2H); ^13^C {^1^H} NMR (101 MHz, CDCl_3_): δ 165.1, 154.3, 145.6, 142.5, 139.1, 129.6, 129.3, 124.5, 121.4, 116.4, 110.8; MS: *m*/*z* 274, 276 (*ca* 3:1, M^+·^).

#### 3.3.11. 2-Methylbenzo[*d*]oxazole-5-carbonitrile (**49**)

Yield: 37 mg (42%) as a white solid, m.p. 119–121 °C (lit. [35] m.p. 120–121 °C); 22 mg of recovered starting material; Purification method B; IR: 2217, 1622 cm^−1^; ^1^H NMR (400 MHz, CDCl_3_): δ 7.98 (dd, *J* = 1.6, 0.7 Hz, 1H), 7.62 (A of AB, dd, *J* = 8.4, 1.6 Hz, 1H), 7.58 (B of AB, dd, *J* = 8.4, 0.7 Hz, 1H), 2.69 (s, 3H); ^13^C {^1^H} NMR (101 MHz, CDCl_3_): δ 166.2, 153.4, 142.0, 128.8, 124.1, 118.8, 111.5, 108.2, 14.6; MS: *m*/*z* 158 (M^+·^). This product was accompanied by 22 mg of recovered **15**, which eluted with 35–50% ether in hexane.

#### 3.3.12. 2-Pentylbenzo[*d*]oxazole-5-carbonitrile (50)

Yield: 50 mg (55%) as a white solid, m.p. 64–65 °C; 28 mg of recovered starting material; Purification method B; IR: 2221, 1623 cm^−1^; ^1^H NMR (400 MHz, CDCl_3_): δ 7.99 (dd, *J* = 1.5, 0.7 Hz, 1H), 7.61 (A of AB, dd, *J* = 8.4, 1.5 Hz, 1H), 7.58 (B of AB dd, *J* = 8.4, 0.7 Hz, 1H), 2.97 (t, *J* = 7.5 Hz, 2H), 1.90 (pentet, *J* = 7.5 Hz, 2H), 1.39 (m, 4H), 0.91 (t, *J* = 7.5 Hz, 3H); ^13^C {^1^H} NMR (101 MHz, CDCl_3_): δ 169.7, 153.2, 141.9, 128.7, 124.2, 118.9, 111.6, 108.1, 31.2, 28.6, 26.2, 22.3, 13.9; MS: *m*/*z* 214 (M^+·^); Anal. Calcd for C_13_H_14_N_2_O: C, 72.87; H, 6.59; N, 13.07; Found: C, 72.81; H, 6.55; N, 13.01. This product was accompanied by 28 mg of recovered **16**, which eluted with 35-50% ether in hexane.

#### 3.3.13. 2-(tert-Butyl) benzo[*d*]oxazole-5-carbonitrile (**51**)

Yield: 64 mg (70%) as a white solid, m.p. 133–134 °C; Purification method B; IR: 2230, 1617 cm^−1^; ^1^H NMR (400 MHz, CDCl_3_): δ 8.00 (m, 1H), 7.62 (A of AB, dd, *J* = 8.4, 1.5 Hz, 1H), 7.58 (B of AB dd, *J* = 8.4, 0.8 Hz, 1H), 1.51 (s, 9H); ^13^C {^1^H} NMR (101 MHz, CDCl_3_): δ 175.9, 153.3, 141.8, 128.7, 124.4, 118.9, 111.6, 108.0, 34.4, 28.3; MS: *m*/*z* 200 (M^+·^); Anal. Calcd for C_12_H_12_N_2_O: C, 71.98; H, 6.04; N, 13.99; Found: C, 71.95; H, 6.03; N, 13.93.

#### 3.3.14. 2-Phenylbenzo[*d*]oxazole-5-carbonitrile (**52**)

Yield: 77 mg (84%) as a tan solid, m.p. 188–189 °C (lit. [36] m.p. 189–190 °C); Purification method B; IR: 2230, 1617 cm^−1^; ^1^H NMR (400 MHz, CDCl_3_): δ 8.27 (m, 2H), 8.09 (m, 1H), 7.69 (A of AB, dd, *J* = 8.4, 0.8 Hz, 1H), 7.66 (B of AB, dd, *J* = 8.4, 1.5 Hz, 1H), 7.63–7.53 (complex, 3H); ^13^C {^1^H} NMR (101 MHz, CDCl_3_): δ 165.2, 153.2, 142.6, 132.5, 129.2, 128.0, 126.1, 124.6, 118.8, 111.9, 108.6 (one aromatic carbon unresolved); MS: *m*/*z* 220 (M^+·^).

#### 3.3.15. 2-(3-Methylphenyl)benzo[*d*]oxazole-5-carbonitrile (**53**)

Yield: 76 mg (82%) as a tan solid, m.p. 161–163 °C; Purification method B; IR: 2232, 1617 cm^−1^; ^1^H NMR (400 MHz, CDCl_3_): δ 8.10–8.03 (complex, 3H), 7.68 (A of AB, dd, *J* = 8.4, 0.8 Hz, 1H), 7.65 (B of AB, dd, *J* = 8.4, 1.5 Hz, 1H), 7.45 (t, *J* = 7.7 Hz, 1H), 7.42 (d, *J* = 7.7 Hz, 1H), 2.47 (s, 3H); ^13^C {^1^H} NMR (101 MHz, CDCl_3_): δ 165.4, 153.1, 142.6, 139.1, 133.4, 129.07, 129.05, 128.6, 125.9, 125.2, 124.5, 118.8, 111.8, 108.5, 21.4; MS: *m*/*z* 234 (M^+·^); Anal. Calcd for C_15_H_10_N_2_O: C, 76.91; H, 4.30; N, 11.96; Found: C, 76.83; H, 4.27; N, 11.90.

#### 3.3.16. 2-(4-Methylphenyl)benzo[*d*]oxazole-5-carbonitrile (**54**)

Yield: 80 mg (87%) as a tan solid, m.p. 209–210 °C; Purification method B; IR: 2230, 1615 cm^−1^; ^1^H NMR (400 MHz, CDCl_3_): δ 8.14 (d, *J* = 8.2 Hz, 2H), 8.05 (s, 1H), 7.66 (A of AB, dd, *J* = 8.4, 0.8 Hz, 1H), 7.63 (B of AB, dd, *J* = 8.4, 1.5 Hz, 1H), 7.36 (d, *J* = 8.1 Hz, 2H), 2.46 (s, 3H); ^13^C {^1^H} NMR (101 MHz, CDCl_3_): δ 165.4, 153.1, 143.3, 142.7, 129.9, 128.9, 128.0, 124.3, 123.3, 118.9, 111.7, 108.5, 21.8; MS: *m*/*z* 234 (M^+·^); Anal. Calcd for C_15_H_10_N_2_O: C, 76.91; H, 4.30; N, 11.96; Found: C, 76.85; H, 4.29; N, 11.94.

#### 3.3.17. 2-(4-Methoxyphenyl)benzo[*d*]oxazole-5-carbonitrile (**55**)

Yield: 80 mg (86%) as a tan solid, m.p. 194–196 °C; Purification method B; IR: 2840, 2230, 1617 cm^−1^; ^1^H NMR (400 MHz, CDCl_3_): δ 8.20 (d, *J* = 8.9 Hz, 2H), 8.03 (s, 1H), 7.64 (A of AB, dd, *J* = 8.5, 0.8 Hz, 1H), 7.62 (B of AB, dd, *J* = 8.5, 1.5 Hz, 1H), 7.06 (d, *J* = 8.9 Hz, 2H), 3.91 (s, 3H); ^13^C NMR {^1^H} (101 MHz, CDCl_3_): δ 165.3, 163.1, 153.1, 142.9, 129.9, 128.7, 124.1, 118.9, 118.4, 114.6, 111.6, 108.4, 55.6; MS: *m*/*z* 250 (M^+·^); Anal. Calcd for C_15_H_10_N_2_O_2_: C, 71.99; H, 4.03; N, 11.19; Found: C, 71.95; H, 4.01; N, 11.12.

#### 3.3.18. 2-(2-Fluorophenyl)benzo[*d*]oxazole-5-carbonitrile (**56**)

Yield: 79 mg (86%) as a tan solid, m.p. 155–156 °C; Purification method B; IR: 2231, 1623 cm^−1^; ^1^H NMR (400 MHz, DMSO-*d_6_*): δ 8.48 (d, *J* = 1.6 Hz, 1H), 8.25 (dt, *J* = 7.7, 1.8 Hz, 1H), 8.06 (d, *J* = 8.5 Hz, 1H), 7.94 (dd, *J* = 8.5, 1.6 Hz, 1H), 7.75 (m, 1H), 7.52–7.45 (complex, 2H); ^13^C NMR {^1^H} (101 MHz, DMSO-*d_6_*): δ 160.7 (d, *J* = 5.0 Hz), 160.1 (d, *J* = 258.1 Hz), 152.4, 141.4, 134.8 (d, *J* = 8.9 Hz), 130.6 129.9, 125.3 (d, *J* = 3.6 Hz), 124.8, 118.6, 117.3 (d, *J* = 20.9 Hz), 113.8 (d, *J* = 10.4 Hz), 112.6, 107.7; MS: *m*/*z* 238 (M^+·^); Anal. Calcd for C_14_H_7_FN_2_O: C, 70.59; H, 4.30; N, 11.96; Found: C, 70.52; H, 4.28; N, 11.91.

#### 3.3.19. 2-(3-Chlorophenyl)benzo[*d*]oxazole-5-carbonitrile (**57**)

Yield: 67 mg (72%) as a tan solid, m.p. 190–192 °C; Purification method A; IR: 2236, 1617 cm^−1^; ^1^H NMR (400 MHz, CDCl_3_): δ 8.26 (t, *J* = 1.8 Hz, 1H), 8.16 (dt, *J* = 7.8, 1.4 Hz, 1H), 8.10 (t, *J* = 1.2 Hz, 1H), 7.72–7.67 (complex, 2H), 7.57 (ddd, *J* = 8.1, 2.1, 1.2 Hz, 1H), 7.51 (t, *J* = 7.8, 1H); ^13^C {^1^H} NMR (101 MHz, CDCl_3_): δ 163.8, 153.1, 142.4, 135.4, 132.5, 130.5, 129.5, 128.0, 127.8, 126.1, 124.9, 118.6, 112.0, 108.9; MS: *m*/*z* 254, 256 (ca. 3:1, M^+·^); Anal. Calcd for C_14_H_7_ClN_2_O: C, 66.03; H, 2.77; N, 11.19; Found: C, 65.97; H, 2.79; N, 11.11.

#### 3.3.20. 2-(4-Chlorophenyl)benzo[*d*]oxazole-5-carbonitrile (**58**)

Yield: 69 mg (74%) as a tan solid, m.p. 225–226 °C; Purification method A; IR: 2232, 1617 cm^−1^; ^1^H NMR (400 MHz, CDCl_3_): δ 8.20 (d, *J* = 8.2 Hz, 2H), 8.08 (s, 1H), 7.68 (s, 2H), 7.54 (d, *J* = 8.2 Hz, 2H); ^13^C {^1^H} NMR (101 MHz, CDCl_3_): δ 164.2, 153.1, 142.5, 138.9, 129.6, 129.34, 129.25, 124.7, 124.5, 118.7, 111.9, 108.8; MS: *m*/*z* 254, 256 (ca. 3:1, M^+·^); Anal. Calcd for C_14_H_7_ClN_2_O: C, 66.03; H, 2.77; N, 11.19; Found: C, 65.99; H, 2.76; N, 11.15.

#### 3.3.21. Methyl 2-phenylbenzo[*d*]oxazole-5-carboxylate (**59**)

Yield: 76 mg (82%) as a white solid, m.p. 151–153 °C; Purification method B; IR: 1711 cm^−1^; ^1^H NMR (400 MHz, CDCl_3_): δ 8.47 (dd, *J* = 1.7, 0.6 Hz, 1H), 8.27 (m, 2H), 8.12 (dd, *J* = 8.6, 1.7 Hz, 1H), 7.62 (dd, *J* = 8.6, 0.6 Hz, 1H), 7.60–7.32 (complex, 3H), 3.97 (s, 3H); ^13^C {^1^H} NMR (101 MHz, CDCl_3_): δ 166.8, 164.4, 153.7, 142.3, 132.0, 129.0, 127.8, 127.1, 126.7, 122.0, 110.4, 52.4 (one aromatic carbon unresolved); MS (*m*/*z*): 253 (M^+·^); Anal. Calcd for C_15_H_11_NO_3_: C, 71.14; H, 4.38; N, 5.53; Found: C, 71.11; H, 4.37; N, 5.50.

#### 3.3.22. Methyl 2-(3-methylphenyl)benzo[*d*]oxazole-5-carboxylate (**60**)

Yield: 78 mg (84%) as a white solid, m.p. 122–123 °C; Purification method B; IR: 1716 cm^−1^; ^1^H NMR (400 MHz, CDCl_3_): δ 8.46 (dd, *J* = 1.7, 0.6 Hz, 1H), 8.11 (dd, *J* = 8.5, 1.7 Hz, 1H), 8.10 (s, 1H), 8.06 (br d, *J* = 7.6 Hz,1H), 7.61 (dd, *J* = 8.5, 0.6 Hz, 1H), 7.43 (t, *J* = 7.6 Hz, 1H), 7.38 (br d, *J* = 7.9 Hz, 1H), 3.97 (s, 3H), 2.47 (s, 3H); ^13^C NMR {^1^H} (101 MHz, CDCl_3_): δ 166.8, 164.6, 153.7, 142.3, 138.9, 132.9, 128.9, 128.4, 127.04, 127.02, 126.5, 124.5, 122.9, 110.4, 52.3, 21.4; MS (*m*/*z*): 267 (M^+·^); Anal. Calcd for C_16_H_13_NO_3_: C, 71.90; H, 4.90; N, 5.24; Found: C, 71.83; H, 4.89; N, 5.19.

#### 3.3.23. Methyl 2-(4-methylphenyl)benzo[*d*]oxazole-5-carboxylate (**61**)

Yield: 83 mg (89%) as a white solid, m.p. 137–138 °C; Purification method B; IR: 1711 cm^−1^; ^1^H NMR (400 MHz, CDCl_3_): δ 8.44 (dd, *J* = 1.7, 0.6 Hz, 1H), 8.15 (d, *J* = 8.3 Hz, 2H), 8.09 (dd, *J* = 8.5, 1.7 Hz, 1H), 7.59 (dd, *J* = 8.5, 0.6 Hz, 1H), 7.35 (d, *J* = 8.3 Hz, 2H), 3.96 (s, 3H), 2.45 (s, 3H); ^13^C {^1^H} NMR (101 MHz, CDCl_3_): δ 166.8, 164.6, 153.7, 142.7, 142.3, 129.8, 127.8, 127.0, 126.9, 123.9, 121.8, 110.3, 52.3, 21.7; MS (*m*/*z*): 267 (M^+·^); Anal. Calcd for C_16_H_13_NO_3_: C, 71.90; H, 4.90; N, 5.24; Found: C, 71.88; H, 4.87; N, 5.17

#### 3.3.24. Methyl 2-(4-methoxyphenyl)benzo[*d*]oxazole-5-carboxylate (**62**)

Yield: 78 mg (83%) as a white solid, m.p. 166–168 °C; Purification method B; IR: 2844, 1703 cm^−1^; ^1^H NMR (400 MHz, CDCl_3_): δ 8.42 (dd, *J* = 1.7, 0.6 Hz, 1H), 8.20 (d, *J* = 8.9 Hz, 2H), 8.08 (dd, *J* = 8.6, 1.7 Hz, 1H), 7.58 (dd, *J* = 8.6, 0.6 Hz, 1H), 7.04 (d, *J* = 8.9 Hz, 2H), 3.96 (s, 3H), 3.90 (s, 3H); ^13^C {^1^H} NMR (101 MHz, CDCl_3_): δ 166.9, 164.5, 162.7, 153.7, 142.5, 129.7, 126.9, 126.6, 121.6, 119.1, 114.5, 110.2, 55.5, 52.3; MS (*m*/*z*): 283 (M^+·^); Anal. Calcd for C_16_H_13_NO_4_: C, 67.84; H, 4.63; N, 4.94; Found: C, 67.81; H, 4.62; N, 4.88.

#### 3.3.25. Methyl 2-(2-fluorophenyl)benzo[*d*]oxazole-5-carboxylate (**63**)

Yield: 84 mg (90%) as a white solid, m.p. 133–135 °C; Purification method B; IR: 1711 cm^−1^; ^1^H NMR (400 MHz, CDCl_3_): δ 8.53 (dd, *J* = 1.7, 0.6 Hz, 1H), 8.26 (td, *J* = 7.6, 1.8 Hz, 1H), 8.15 (dd, *J* = 8.6, 1.7 Hz, 1H), 7.66 (dd, *J* = 8.6, 0.6 Hz, 1H), 7.60–7.53 (complex, 1H), 7.36–7.26 (complex, 2H), 3.97 (s, 3H); ^13^C {^1^H} NMR (101 MHz, CDCl_3_): δ 166.7, 160.9 (d, *J* = 259.7 Hz), 160.8 (d, *J* = 5.0 Hz), 153.4, 141.8, 133.6 (d, *J* = 9.1 Hz,), 130.7 (d, *J* = 1.1 Hz), 127.4, 127.2, 124.6 (d, *J* = 3.0 Hz), 122.4, 117.2 (d, *J* = 21.1 Hz), 115.1 (d, *J* = 11.1 Hz), 110.6, 52.4; MS (*m*/*z*): 271 (M^+·^); Anal. Calcd for C_15_H_10_FNO_3_: C, 66.42; H, 3.72; N, 5.16; Found: C, 66.36; H, 3.68; N, 5.09.

#### 3.3.26. Methyl 2-(3-chlorophenyl)benzo[*d*]oxazole-5-carboxylate (**64**)

Yield: 86 mg (92%) as a white solid, m.p. 138–139 °C; Purification method B; IR: 1715 cm^−1^; ^1^H NMR (400 MHz, CDCl_3_): δ 8.47 (dd, *J* = 1.7, 0.6 Hz, 1H), 8.26 (t, *J* = 1.9 Hz, 1H), 8.14 (m, 2H), 7.62 (dd, *J* = 8.5, 0.6 Hz, 1H), 7.53 (ddd, *J* = 8.0, 2.1, 1.2 Hz, 1H), 7.48 (t, *J* = 7.8 Hz, 1H), 3.97 (s, 3H); ^13^C {^1^H} NMR (101 MHz, CDCl_3_): δ 166.6, 162.9, 153.7, 142.0, 135.2, 132.0, 130.3, 128.3, 127.8, 127.5, 127.3, 125.9, 122.2, 110.5, 52.4; MS (*m*/*z*): 287, 289 (ca 3:1, M^+·^); Anal. Calcd for C_15_H_10_ClNO_3_: C, 62.62; H, 3.50; N, 4.87; Found: C, 62.56; H, 3.48; N, 4.82.

#### 3.3.27. Methyl 2-(4-chlorophenyl)benzo[*d*]oxazole-5-carboxylate (**65**)

Yield: 82 mg (88%) as a white solid, m.p. 164–165 °C; Purification method B; IR: 1711 cm^−1^; ^1^H NMR (400 MHz, CDCl_3_): δ 8.46 (dd, *J* = 1.7, 0.6 Hz, 1H), 8.20 (d, *J* = 8.7 Hz, 2H), 8.12 (dd, *J* = 8.6, 1.7 Hz, 1H), 7.61 (dd, *J* = 8.6, 0.6, 1H), 7.52 (d, *J* = 8.7 Hz, 2H), 3.97 (s, 3H); ^13^C {^1^H} NMR (101 MHz, CDCl_3_): δ 166.7, 163.4, 153.7, 142.2, 138.3, 129.4, 129.1, 127.29, 127.25, 125.1, 122.1, 110.4, 52.4; MS (*m*/*z*): 287, 289 (ca 3:1, M^+·^); Anal. Calcd for C_15_H_10_ClNO_3_: C, 62.62; H, 3.50; N, 4.87; Found: C, 62.60; H, 3.47; N, 4.81.

#### 3.3.28. 2-Phenyl-5-(trifluoromethyl)benzo[*d*]oxazole (**66**)

Yield: 83 mg (89%) as a white solid, m.p. 84–86 °C (lit. [9] m.p. 86–87 °C^)^; Purification method B; IR: 1614, 1109 cm^−1^; ^1^H NMR (400 MHz, CDCl_3_): δ 8.26 (d, *J* = 8.3 Hz, 2H), 8.05 (s, 1H), 7.70–7.62 (complex, 2H), 7.61–7.52 (complex, 3H); ^13^C {^1^H} NMR (101 MHz, CDCl_3_): δ 164.8, 152.5, 142.3, 132.2, 129.1, 127.9, 127.4 (q, *J* = 32.7 Hz), 126.5, 124.2 (q, *J* = 272.1 Hz), 123.3 (q, *J* = 3.8 Hz), 117.2 (q, *J* = 4.0 Hz), 111.0; ^19^F {^1^H} NMR (376 MHz, CDCl_3_ referenced to C_6_H_5_F): δ –61.18; MS (*m*/*z*): 263 (M^+·^).

#### 3.3.29. 2-(3-Methylphenyl)-5-(trifluoromethyl)benzo[*d*]oxazole (**67**)

Yield: 88 mg (94%) as a white solid, m.p. 97–98 °C; Purification method B; IR: 1625, 1117 cm^−1^; ^1^H NMR (400 MHz, CDCl_3_): δ 8.10 (br s, 1H), 8.06 (d, *J* = 7.6 Hz, 1H), 8.04 (br s, 1H), 7.67 (A of AB, d, *J* = 8.6 Hz, 1H), 7.35 (B of AB, dd, *J* = 8.6, 1.7 Hz, 1H), 7.44 (t, *J* = 7.6 Hz, 1H), 7.39 (d, *J* = 7.6 Hz, 1H), 2.47 (s, 3H); ^13^C {^1^H} NMR (101 MHz, CDCl_3_): δ 165.0, 152.5, 142.3, 139.0, 133.0, 129.0, 128.5, 127.4 (q, *J* = 32.6 Hz), 126.3, 125.1, 124.2 (q, *J* = 272.2 Hz), 122.3, (q, *J* = 3.7 Hz), 117.6 (q, *J* = 4.1 Hz), 111.0, 21.4; ^19^F {^1^H} NMR (376 MHz, CDCl_3_ referenced to C_6_H_5_F): δ –61.17; MS (*m*/*z*): 277 (M^+·^); Anal. Calcd for C_15_H_10_F_3_NO: C, 64.98; H, 3.64; N, 5.05; Found: C, 65.01; H, 3.66; N, 4.99.

#### 3.3.30. 2-(4-Methylphenyl)-5-(trifluoromethyl)benzo[*d*]oxazole (**68**)

Yield: 86 mg (92%) as a white solid, m.p. 104–105 °C; Purification method B; IR: 1625, 1117 cm^−1^; ^1^H NMR (400 MHz, CDCl_3_): δ 8.15 (d, *J* = 8.0 Hz, 2H), 8.02 (br s, 1H), 7.65 (A of AB, d, *J* = 8.5 Hz, 1H), 7.61 (B of AB, dd, *J* = 8.5, 1.7 Hz, 1H), 7.35 (d, *J* = 8.0 Hz, 2H), 2.45 (s, 3H); ^13^C {^1^H} NMR (101 MHz, CDCl_3_): δ 165.0, 152.4, 142.9, 142.4, 129.8, 127.8, 127.3 (q, *J* = 32.6 Hz), 124.3 (q, *J* = 272.2 Hz), 123.7, 122.1 (q, *J* = 3.7 Hz), 117.5 (q, *J* = 4.0 Hz), 110.9, 21.7; ^19^F {^1^H} NMR (376 MHz, CDCl_3_ referenced to C_6_H_5_F): δ –61.16; MS (*m*/*z*): 277 (M^+·^); Anal. Calcd for C_15_H_10_F_3_NO: C, 64.98; H, 3.64; N, 5.05; Found: C, 64.92; H, 3.61; N, 4.96.

#### 3.3.31. 2-(4-Methoxyphenyl)-5-(trifluoromethyl)benzo[*d*]oxazole (**69**)

Yield: 77 mg (82%) as a white solid, m.p. 88–89 °C; Purification method B; IR: 2843, 1608, 1113 cm^−1^; ^1^H NMR (400 MHz, CDCl_3_): δ 8.21 (d, *J* = 8.9 Hz, 2H), 8.00 (br s, 1H), 7.64 (A of AB, d, *J* = 8.6 Hz, 1H), 7.59 (B of AB, dd, *J* = 8.6, 1.7 Hz, 1H), 7.05 (d, *J* = 8.9 Hz, 2H), 3.91 (s, 3H); ^13^C {^1^H} NMR (101 MHz, CDCl_3_): δ 164.9, 162.8, 152.4, 142.5, 129.7, 127.2 (q, *J* = 32.3 Hz), 124.3 (q, *J* = 271.9 Hz), 121.8 (q, *J* = 3.7 Hz), 118.9, 117.2 (q, *J* = 4.0 Hz), 114.5, 110.8, 55.5; ^19^F {^1^H} NMR (376 MHz, CDCl_3_ referenced to C_6_H_5_F): δ –61.14; MS (*m*/*z*): 293 (M^+·^); Anal. Calcd for C_15_H_10_F_3_NO_2_: C, 61.44; H, 3.44; N, 5.32; Found: C, 61.43; H, 3.44; N, 5.29.

#### 3.3.32. 2-(2-Fluorophenyl)-5-(trifluoromethyl)benzo[*d*]oxazole (**70**)

Yield: 85 mg (91%) as a white solid, m.p. 76–77 °C; Purification method B; IR: 1626, 1117 cm^−1^; ^1^H NMR (400 MHz, CDCl_3_): δ 8.24 (td, *J* = 7.5, 1.8 Hz, 1H), 8.11 (br s, 1H), 7.71 (A of AB, d, *J* = 8.6 Hz, 1H), 7.66 (B of AB, dd, *J* = 8.6, 1.8 Hz, 1H), 7.60–7.54 (complex, 1H), 7.34 (td, *J* = 7.6, 1.1 Hz, 1H), 7.30 (ddd, *J* = 10.8, 2.7, 1.1 Hz, 1H); ^13^C {^1^H} NMR (101 MHz, CDCl_3_): δ 161.2 (d, *J* = 5.2 Hz), 161.1 (d, *J* = 259.8 Hz), 152.2, 141.9, 133.8 (d, *J* = 8.8 Hz), 130.7 (d, *J* = 1.3 Hz), 127.5 (d, *J* = 32.7 Hz), 124.7 (d, *J* = 3.7 Hz), 124.2 (q, *J* = 272.2 Hz), 122.7 (q, *J* = 3.7 Hz), 118.1 (q, *J* = 4.1 Hz), 117.2 (d, *J* = 21.4 Hz), 114.9 (d, *J* = 10.5 Hz), 111.2; ^19^F {^1^H} NMR (376 MHz, CDCl_3_ referenced to C_6_H_5_F): δ –61.21, –109.62; MS (*m*/*z*): 281 (M+·); Anal. Calcd for C_14_H_7_F_4_NO: C, 61.44; H, 3.44; N, 5.32; Found: C, 61.39; H, 3.42; N, 5.28.

#### 3.3.33. 2-(3-Chlorophenyl)-5-(trifluoromethyl)benzo[*d*]oxazole (**71**)

Yield: 78 mg (83%) as a white solid, m.p. 97–98 °C; Purification method B; IR: 1626, 1109 cm^−1^; ^1^H NMR (400 MHz, CDCl_3_): δ 8.27 (t, *J* = 1.9 Hz, 1H), 8.16 (dt, *J* = 7.7, 1.5 Hz, 1H), 8.06 (br s, 1H), 7.70 (A of AB, d, *J* = 8.6 Hz, 1H), 7.66 (B of AB, dd, *J* = 8.6, 1.7 Hz, 1H), 7.56 (ddd, *J* = 8.1, 2.1, 1.2 Hz, 1H), 7.49 (t, *J* = 7.7 Hz, 1H); ^13^C {^1^H} NMR (101 MHz, CDCl_3_): δ 163.4, 152.5, 142.1, 135.3, 132.2, 130.4, 128.2, 127.9, 127.7 (q, *J* = 32.7 Hz), 125.9, 124.1 (q, *J* = 272.2 Hz), 122.8 (q, *J* = 3.8 Hz), 118.0 (q, *J* = 4.1 Hz), 111.2; ^19^F {^1^H} NMR (376 MHz, CDCl_3_ referenced to C_6_H_5_F): δ –61.23; MS (*m*/*z*): 297, 299 (ca. 3:1, M^+·^); Anal. Calcd for C_14_H_7_ClF_3_NO: C, 56.49; H, 2.37; N, 4.71; Found: C, 56.45; H, 2.34; N, 4.69.

#### 3.3.34. 2-(4-Chlorophenyl)-5-(trifluoromethyl)benzo[*d*]oxazole (**72**)

Yield: 80 mg (85%) as a white solid, m.p. 107–108 °C; Purification method A; IR: 1621, 1120 cm^−1^; ^1^H NMR (400 MHz, CDCl_3_): δ 8.20 (d, *J* = 8.6 Hz, 2H), 8.04 (br s, 1H), 7.68 (A of AB, d, *J* = 8.6 Hz, 1H), 7.64 (B of AB, dd, *J* = 8.6, 1.7 Hz, 1H), 7.53 (d, *J* = 8.6 Hz, 2H); ^13^C {^1^H} NMR (101 MHz, CDCl_3_): δ 163.8, 152.5, 142.2, 138.6, 129.5, 129.1, 127.6 (q, *J* = 32.7 Hz), 125.0, 124.2 (q, *J* = 272.2 Hz), 122.6 (q, *J* = 3.6 Hz), 117.8 (q, *J* = 4.0 Hz), 111.0; ^19^F {^1^H} NMR (376 MHz, CDCl_3_ referenced to C_6_H_5_F): δ –61.15; MS (*m*/*z*): 297, 299 (ca. 3:1, M^+·^); Anal. Calcd for C_14_H_7_ClF_3_NO: C, 56.49; H, 2.37; N, 4.71; Found: C, 56.43; H, 2.34; N, 4.66.

### 3.4. Control Reaction: Attempted Cyclization of N-(2-Fluorophenyl)benzamide (**73**)

A mixture of 100 mg (0.47 mmol) of *N*-(2-fluorophenyl)benzamide (**73**) in 4 mL of anhydrous DMF was treated with 128 mg (0.93 mmol, 2 equiv.) of anhydrous K_2_CO_3_ and heated under N_2_ at 130 °C for 24 h. Thin layer chromatography indicated no reaction. Work-up as described in the standard procedure yielded 93 mg (0.43 mmol, 93%) of recovered starting material.

## 4. Conclusions

Benzo[*d*]oxazoles substituted at C2 and C5 have been prepared by an *N*-deprotonation–*O*-S_N_Ar sequence from acetanilide and benzanilide precursors. Substrates were prepared by acylating 2-fluoroanilines with a C5 by electron-withdrawing substituent (NO_2_, CN, CO_2_Me, or CF_3_) to activate the system toward S_N_Ar ring closure. The treatment of these precursors with K_2_CO_3_ (2 equiv.) in anhydrous DMF at elevated temperatures gave 2-alkyl or aryl 5-substituted benzo[*d*]oxazoles in high yields. No other additives or catalysts were required to promote the cyclization. The temperature and reaction time for benzanilide closure correlated with the activating power of the C5 electron-withdrawing substituent with NO_2_ being most effective (90 °C, 1 h), CN and CO_2_Me being intermediate (115–120 °C, 2 h), and CF_3_ being the least effective (130 °C, 3 h). Acetanilides were harder to predict but usually required 4–6 h at the same temperatures for NO_2_ and CN; acetanilides incorporating CO_2_Me and CF_3_ activation were not investigated as the higher temperatures, and extended reaction times caused a significant degradation of the enolizable alkyl groups. Purification was best accomplished by passing the crude product through a short silica gel column with 5–25% ether in petroleum ether. Thirty-four examples were prepared in yields ranging from 51 to 94%, with benzanilides giving the highest yields.

## Figures and Tables

**Table 1:** Synthesis of 2,5-disubstituted benzo[*d*]oxazoles from anilide derivatives ^a^.

^ 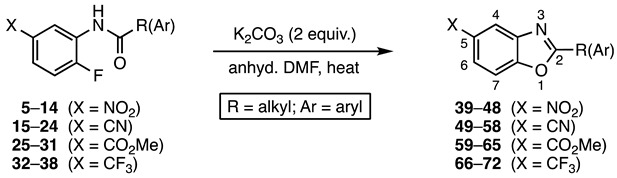 ^					
X	R(Ar)	Substrate Compound Number	Product Compound Number	Purification Method ^b^	Benzo[*d*]oxazole Yield (%)
NO_2_	CH_3_	**5**	**39**	A B	34 51
NO_2_	C_5_H_11_	**6**	**40**	A B	39 74
NO_2_	*t*-C_4_H_9_	**7**	**41**	B	84
NO_2_	Ph	**8**	**42**	A	84
NO_2_	3-Me-Ph	**9**	**43**	A	82
NO_2_	4-Me-Ph	**10**	**44**	A	88
NO_2_	4-MeO-Ph	**11**	**45**	A	86
NO_2_	2-F-Ph	**12**	**46**	A	88
NO_2_	3-Cl-Ph	**13**	**47**	B	68
NO_2_	4-Cl-Ph	**14**	**48**	A	76
CN	CH_3_	**15**	**49**	B	48 ^c^
CN	C_5_H_11_	**16**	**50**	B	55 ^d^
CN	*t*-C_4_H_9_	**17**	**51**	B	70
CN	Ph	**18**	**52**	B	84
CN	3-Me-Ph	**19**	**53**	B	82
CN	4-Me-Ph	**20**	**54**	B	87
CN	4-MeO-Ph	**21**	**55**	B	86
CN	2-F-Ph	**22**	**56**	B	86
CN	3-Cl-Ph	**23**	**57**	A	72
CN	4-Cl-Ph	**24**	**58**	A	74
CO_2_Me	Ph	**25**	**59**	B	82
CO_2_Me	3-Me-Ph	**26**	**60**	B	84
CO_2_Me	4-Me-Ph	**27**	**61**	B	89
CO_2_Me	4-MeO-Ph	**28**	**62**	B	83
CO_2_Me	2-F-Ph	**29**	**63**	B	90
CO_2_Me	3-Cl-Ph	**30**	**64**	B	92
CO_2_Me	4-Cl-Ph	**31**	**65**	B	88
CF_3_	Ph	**32**	**66**	B	89
CF_3_	3-Me-Ph	**33**	**67**	B	94
CF_3_	4-Me-Ph	**34**	**68**	A B	52 92
CF_3_	4-MeO-Ph	**35**	**69**	B	82
CF_3_	2-F-Ph	**36**	**70**	B	91
CF_3_	3-Cl-Ph	**37**	**71**	A B	46 83
CF_3_	4-Cl-Ph	**38**	**72**	B	85

^a^ All cyclizations were run on 100 mg of the anilide substrate. ^b^ Method A: Repeated recrystallization from absolute ethanol; Method B: Chromatography on a 25 cm × 1 cm silica gel column eluted with 5–25% ether in petroleum ether. ^c^ After 4 h at 115 °C, this product was accompanied by 22 mg of recovered 15. ^d^ After 4 h at 115 °C, this product was accompanied by 28 mg of recovered 16.

## Data Availability

The only data available is the spectral data in the Supplemental Materials.

## Supplementary Materials

The following supporting information can be downloaded at: https://www.mdpi.com/article/10.3390/molecules29184322/s1. Copies of ^1^H-NMR and ^13^C-NMR spectra are provided for all anilides and products. ^19^F-NMR spectra are given for all anilides bearing more than one fluorine as well as cyclized products with fluorine substituents.
