# Supplementary material for: Benzo[d]oxazoles from Anilides by N-Deprotonation–O-SNAr Cyclization
Source: Molecules. 2024 Sep 12;29(18):4322. doi: 10.3390/molecules29184322 (PMC11434243; doi:10.3390/molecules29184322)

Benzo[*d*]oxazoles from Anilides by *N*-Deprotonation–*O*-SNAr Cyclization

Nash Nevels, Luke Subera and Richard A. Bunce*
Department of Chemistry, Oklahoma State University, Stillwater, OK 74078-3071, U.S.A.
nnevels@okstate.edu (N.N.); luke.subera@okstate.edu
*Correspondence: rab@okstate.edu; Tel. 01-405-744-5952

Spectra were run at 400 MHz (^{1}H NMR), 101 MHz (^{13}C NMR) or 376 MHz (^{19}F NMR). Spectra were run in $CDCl_3$, DMSO-d_6 or mixtures of these solvents. DMSO-d_6 was ocassionally required due to low solubility of the anilides which made observation of C–F couplings in low intensity signals difficult to observe. The internal standard for ^{1}H and ^{13}C NMRs was tetramethylsilane at δ 0.00; for ^{19}F NMRs, fluorobenzene was the standard at δ -113.15.

Compound **page**

^{1}H and ^{13}C NMR for *N*-(2-Fluoro-5-nitrophenyl)acetamide (**5**) …… S4
^{1}H and ^{13}C NMR for *N*-(2-Fluoro-5-nitrophenyl)hexanamide (**6**) …… S5
^{1}H and ^{13}C NMR for *N*-(2-Fluoro-5-nitrophenyl)pivalamide (**7**) …… S6
^{1}H and ^{13}C NMR for *N*-(2-Fluoro-5-nitrophenyl)benzamide (**8**) …… S7
^{1}H and ^{13}C NMR for *N*-(2-Fluoro-5-nitrophenyl)-3-methylbenzamide (**9**) …… S8
^{1}H and ^{13}C NMR for *N*-(2-Fluoro-5-nitrophenyl)-4-methylbenzamide (**10**) …… S9
^{1}H and ^{13}C NMR for *N*-(2-Fluoro-5-nitrophenyl)-4-methoxybenzamide (**11**) …… S10
^{1}H and ^{13}C NMR for 2-Fluoro-*N*-(2-fluoro-5-nitrophenyl)benzamide (**12**) …… S11
^{19}F NMR for 2-Fluoro-*N*-(2-fluoro-5-nitrophenyl)benzamide (**12**) …… S12
^{1}H and ^{13}C NMR for 3-Chloro-*N*-(2-fluoro-5-nitrophenyl)benzamide (**13**) …… S13
^{1}H and ^{13}C NMR for 4-Chloro-*N*-(2-fluoro-5-nitrophenyl)benzamide (**14**) …… S14
^{1}H and ^{13}C NMR for *N*-(5-Cyano-2-fluorophenyl)acetamide (**15**) …… S15
^{1}H and ^{13}C NMR for *N*-(5-Cyano-2-fluorophenyl)hexanamide (**16**) …… S16
^{1}H and ^{13}C NMR for *N*-(5-Cyano-2-fluorophenyl)pivalamide (**17**) …… S17
^{1}H and ^{13}C NMR for *N*-(5-Cyano-2-fluorophenyl)benzamide (**18**) …… S18
^{1}H and ^{13}C NMR for *N*-(5-Cyano-2-fluorophenyl)-3-methylbenzamide (**19**) …… S19
^{1}H and ^{13}C NMR for *N*-(5-Cyano-2-fluorophenyl)-4-methylbenzamide (**20**) …… S20
^{1}H and ^{13}C NMR for *N*-(5-Cyano-2-fluorophenyl)-4-methoxybenzamide (**21**) …… S21
^{1}H and ^{13}C NMR for *N*-(5-Cyano-2-fluorophenyl)-2-fluorobenzamide (**22**) …… S22
^{19}F NMR for *N*-(5-Cyano-2-fluorophenyl)-2-fluorobenzamide (**22**) …… S23
^{1}H and ^{13}C NMR for 3-Chloro-*N*-(5-cyano-2-fluorophenyl)benzamide (**23**) …… S24
^{1}H and ^{13}C NMR for 4-Chloro-*N*-(5-cyano-2-fluorophenyl)benzamide (**24**) …… S25
^{1}H and ^{13}C NMR for Methyl 3-benzamido-4-fluorobenzoate (**25**) …… S26
^{1}H and ^{13}C NMR for Methyl 4-fluoro-3-(3-methylbenzamido)benzoate (**26**) …… S27
^{1}H and ^{13}C NMR for Methyl 4-fluoro-3-(4-methylbenzamido)benzoate (**27**) …… S28
^{1}H and ^{13}C NMR for Methyl 4-fluoro-3-(4-methoxybenzamido)benzoate (**28**) …… S29
^{1}H and ^{13}C NMR for Methyl 4-fluoro-3-(2-fluorobenzamido)benzoate (**29**) …… S30

^{19}F NMR for Methyl 4-fluoro-3-(2-fluorobenzamido)benzoate (**29**) ... S31
^{1}H and ^{13}C NMR for Methyl 3-(3-chlorobenzamido)-4-fluorobenzoate (**30**) ... S32
^{1}H and ^{13}C NMR for Methyl 3-(4-chlorobenzamido)-4-chlorobenzoate (**31**) ... S33
^{1}H and ^{13}C NMR for *N*-(2-Fluoro-5-trifluoromethyl)phenyl)benzamide (**32**) ... S34
^{19}F NMR for *N*-(2-Fluoro-5-trifluoromethyl)phenyl)benzamide (**32**) ... S35
^{1}H and ^{13}C NMR for *N*-(2-Fluoro-5-(trifluoromethyl)phenyl)-3-methylbenzamide (**33**) ... S36
^{19}F NMR for *N*-(2-Fluoro-5-(trifluoromethyl)phenyl)-3-methylbenzamide (**33**) ... S37
^{1}H and ^{13}C NMR for *N*-(2-Fluoro-5-(trifluoromethyl)phenyl)-4-methylbenzamide (**34**) ... S38
^{19}F NMR for *N*-(2-Fluoro-5-(trifluoromethyl)phenyl)-4-methylbenzamide (**34**) ... S39
^{1}H and ^{13}C NMR for *N*-(2-Fluoro-5-(trifluoromethyl)phenyl)-4-methoxybenzamide (**35**) ... S40
^{19}F NMR for *N*-(2-Fluoro-5-(trifluoromethyl)phenyl)-4-methoxybenzamide (**35**) ... S41
^{1}H and ^{13}C NMR for 2-Fluoro-*N*-(2-fluoro-5-(trifluoromethyl)phenyl)benzamide (**36**) ...S42
^{19}F NMR for 2-Fluoro-*N*-(2-fluoro-5-(trifluoromethyl)phenyl)benzamide (**36**) ... S43
^{1}H and ^{13}C NMR for 3-Chloro-*N*-(2-fluoro-5-(trifluoromethyl)phenyl)benzamide (**37**) ... S44
^{19}F NMR for 3-Chloro-*N*-(2-fluoro-5-(trifluoromethyl)phenyl)benzamide (**37**) ... S45
^{1}H and ^{13}C NMR for 4-Chloro-*N*-(2-fluoro-5-(trifluoromethyl)phenyl)benzamide (**38**) ... S46
^{19}F NMR for 4-Chloro-*N*-(2-fluoro-5-(trifluoromethyl)phenyl)benzamide (**38**) ... S47
^{1}H and ^{13}C NMR for 2-Methyl-5-nitrobenzo[*d*]oxazole (**39**) ... S48
^{1}H and ^{13}C NMR for 5-Nitro-2-pentylbenzo[*d*]oxazole (**40**) ... S49
^{1}H and ^{13}C NMR for 2-(*tert*-Butyl)-5-nitrobenzo[*d*]oxazole (**41**) ... S50
^{1}H and ^{13}C NMR for 5-Nitro-2-phenylbenzo[*d*]oxazole (**42**) ... S51
^{1}H and ^{13}C NMR for 2-(3-Methylphenyl)-5-nitrobenzo[*d*]oxazole (**43**) ... S52
^{1}H and ^{13}C NMR for 2-(4-Methylphenyl)-5-nitrobenzo[*d*]oxazole (**44**) ... S53
^{1}H and ^{13}C NMR for 2-(4-Methoxyphenyl)-5-nitrobenzo[*d*]oxazole (**45**)... S54
^{1}H and ^{13}C NMR for 2-(2-Fluorophenyl)-5-nitrobenzo[*d*]oxazole (**46**) ... S55
^{1}H and ^{13}C NMR for 2-(3-Chlorophenyl)-5-nitrobenzo[*d*]oxazole (**47**) ... S56
^{1}H and ^{13}C NMR for 2-(4-Chlorophenyl)-5-nitrobenzo[*d*]oxazole (**48**) ... S57
^{1}H and ^{13}C NMR for 2-Methylbenzo[*d*]oxazole-5-carbonitrile (**49**) ... S58
^{1}H and ^{13}C NMR for 2-Pentylbenzo[*d*]oxazole-5-carbonitrile (**50**) ... S59
^{1}H and ^{13}C NMR for 2-(*tert*-Butyl) benzo[*d*]oxazole-5-carbonitrile (**51**) ... S60
^{1}H and ^{13}C NMR for 2-Phenylbenzo[*d*]oxazole-5-carbonitrile (**52**) ... S61
^{1}H and ^{13}C NMR for 2-(3-Methylphenyl)benzo[*d*]oxazole-5-carbonitrile (**53**) ... S62
^{1}H and ^{13}C NMR for 2-(4-Methylphenyl)benzo[*d*]oxazole-5-carbonitrile (**54**) ... S63
^{1}H and ^{13}C NMR for 2-(4-Methoxyphenyl)benzo[*d*]oxazole-5-carbonitrile (**55**) ... S64
^{1}H and ^{13}C NMR for 2-(2-Fluorophenyl)benzo[*d*]oxazole-5-carbonitrile (**56**) ... S65
^{1}H and ^{13}C NMR for 2-(3-Chlorophenyl)benzo[*d*]oxazole-5-carbonitrile (**57**) ... S66
^{1}H and ^{13}C NMR for 2-(4-Chlorophenyl)benzo[*d*]oxazole-5-carbonitrile (**58**) ... S67
^{1}H and ^{13}C NMR for Methyl 2-phenylbenzo[*d*]oxazole-5-carboxylate (**59**) ... S68
^{1}H and ^{13}C NMR for Methyl 2-(3-methylphenyl)benzo[*d*]oxazole-5-carboxylate (**60**) ... S69
^{1}H and ^{13}C NMR for Methyl 2-(4-methylphenyl)benzo[*d*]oxazole-5-carboxylate (**61**) ... S70
^{1}H and ^{13}C NMR for Methyl 2-(4-methoxyphenyl)benzo[*d*]oxazole-5-carboxylate (**62**) ... S71
^{1}H and ^{13}C NMR for Methyl 2-(2-fluorophenyl)benzo[*d*]oxazole-5-carboxylate (**63**) ... S72
^{1}H and ^{13}C NMR for Methyl 2-(3-chlorophenyl)benzo[*d*]oxazole-5-carboxylate (**64**) ... S73
^{1}H and ^{13}C NMR for Methyl 2-(4-chlorophenyl)benzo[*d*]oxazole-5-carboxylate (**65**) ... S74

^{1}H and ^{13}C NMR for 2-Phenyl-5-(trifluoromethyl)benzo[*d*]oxazole (**66**) …………………………………… S75
^{19}F NMR for 2-Phenyl-5-(trifluoromethyl)benzo[*d*]oxazole (**66**) ……………………………………………….. S79
^{19}F NMR for 2-(4-Methylphenyl)-5-(trifluoromethyl)benzo[*d*]oxazole (**68**)…………………………………… S80
^{1}H and ^{13}C NMR for 2-(4-Methoxyphenyl)-5-(trifluoromethyl)benzo[*d*]oxazole (**69**) ……………………… S81
^{19}F NMR for 2-(4-Methoxyphenyl)-5-(trifluoromethyl)benzo[*d*]oxazole (**69**) …………………………………… S82
^{1}H and ^{13}C NMR for 2-(2-Fluorophenyl)-5-(trifluoromethyl)benzo[*d*]oxazole (**70**) ……………………….. S83
^{19}F NMR for 2-(2-Fluorophenyl)-5-(trifluoromethyl)benzo[*d*]oxazole (**70**) …………………………………… S84
^{1}H and ^{13}C NMR for 2-(3-Chlorophenyl)-5-(trifluoromethyl)benzo[*d*]oxazole (**71**) ………………………… S85
^{19}F NMR for 2-(3-Chlorophenyl)-5-(trifluoromethyl)benzo[*d*]oxazole (**71**) …………………………………… S86
^{1}H and ^{13}C NMR for 2-(4-Chlorophenyl)-5-(trifluoromethyl)benzo[*d*]oxazole (**72**) ……………………….. S87
^{19}F NMR for 2-(4-Chlorophenyl)-5-(trifluoromethyl)benzo[*d*]oxazole (**72**) ……………………………….. S88

^{1}H and ^{13}C NMR for *N*-(2-Fluoro-5-nitrophenyl)acetamide (**5**)

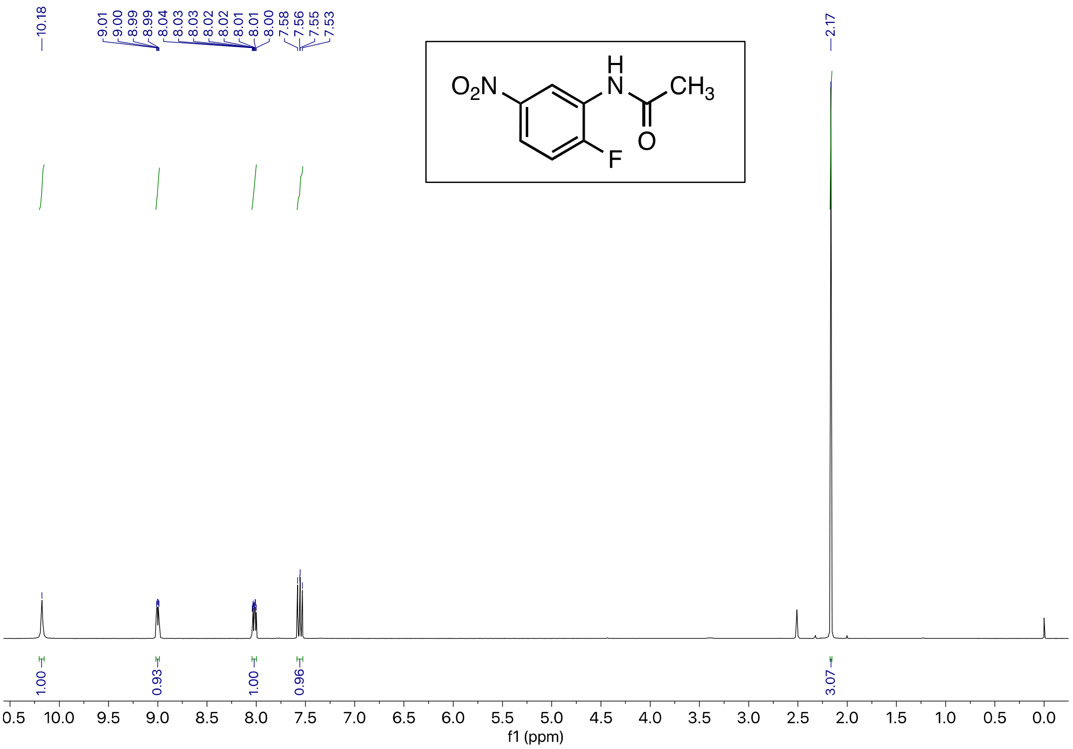

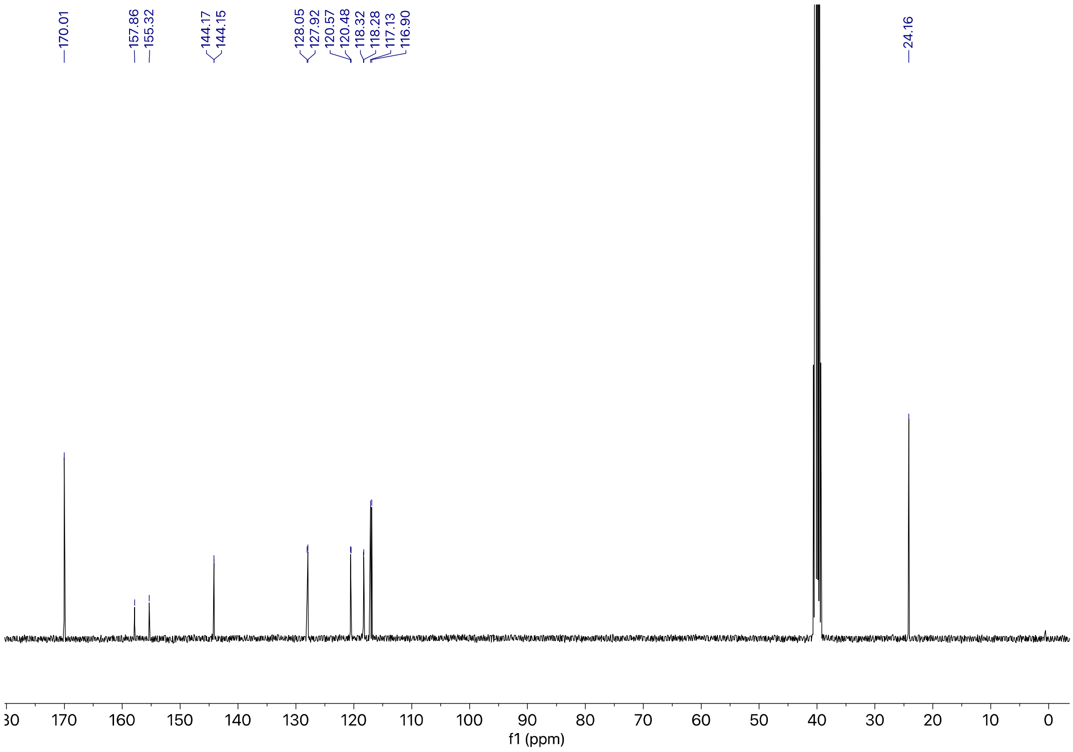

^{1}H and ^{13}C NMR for *N*-(2-Fluoro-5-nitrophenyl)hexanamide (**6**)

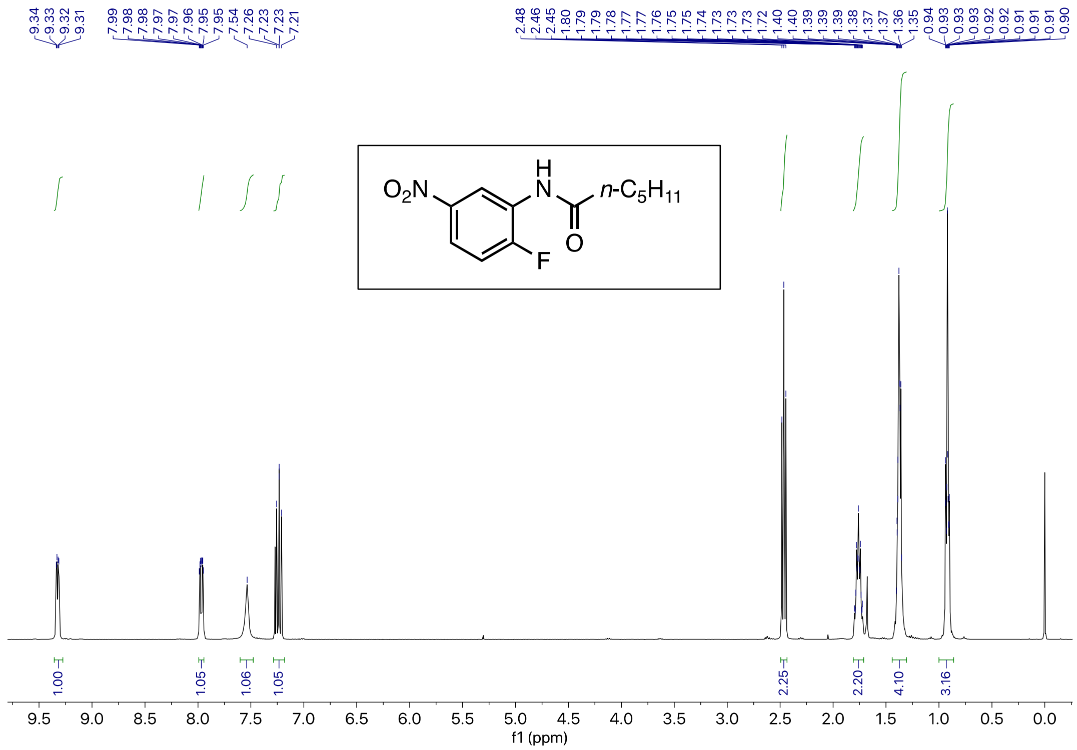

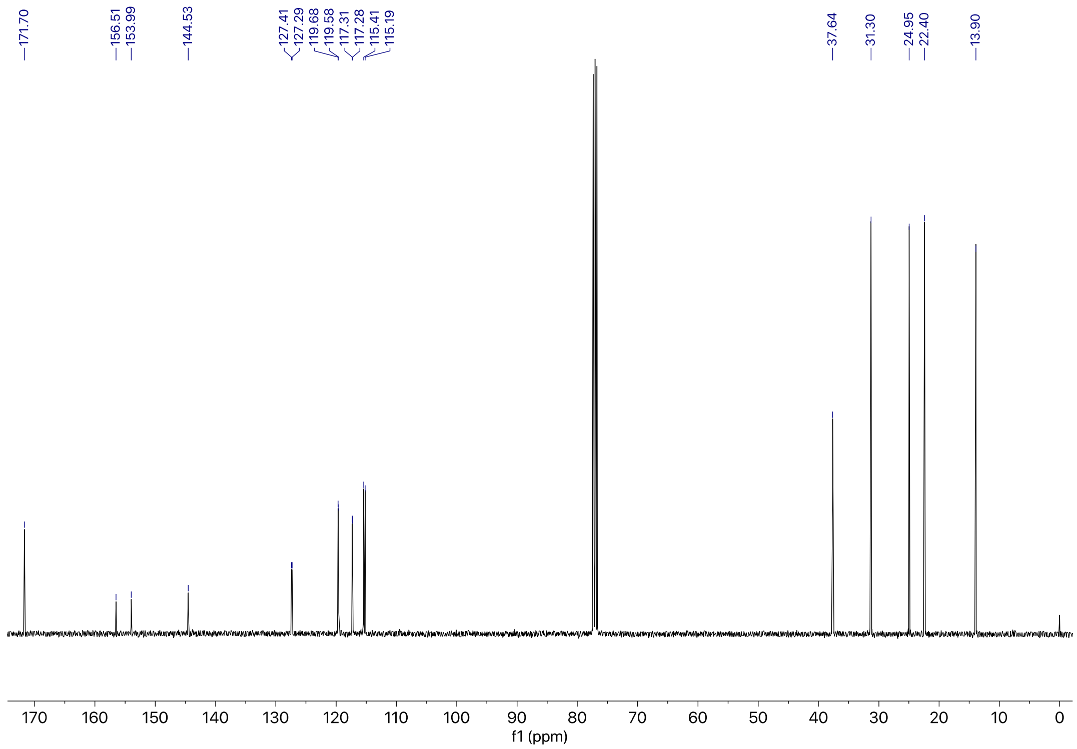

^{1}H and ^{13}C NMR for *N*-(2-Fluoro-5-nitrophenyl)pivalamide (**7**)

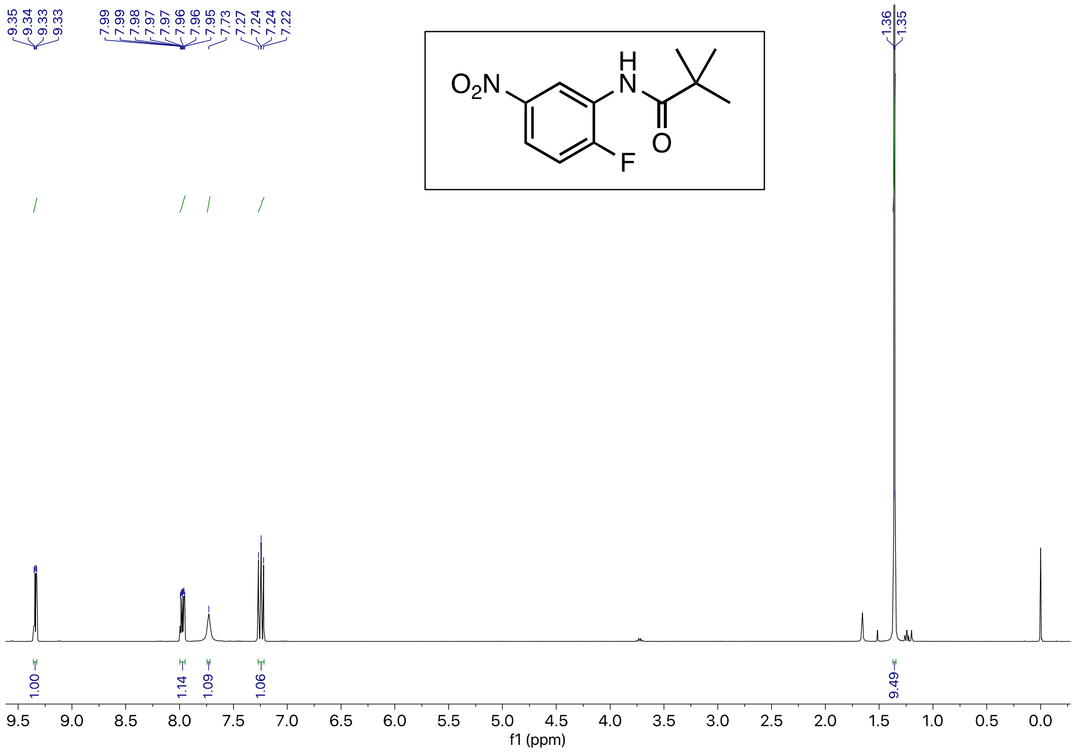

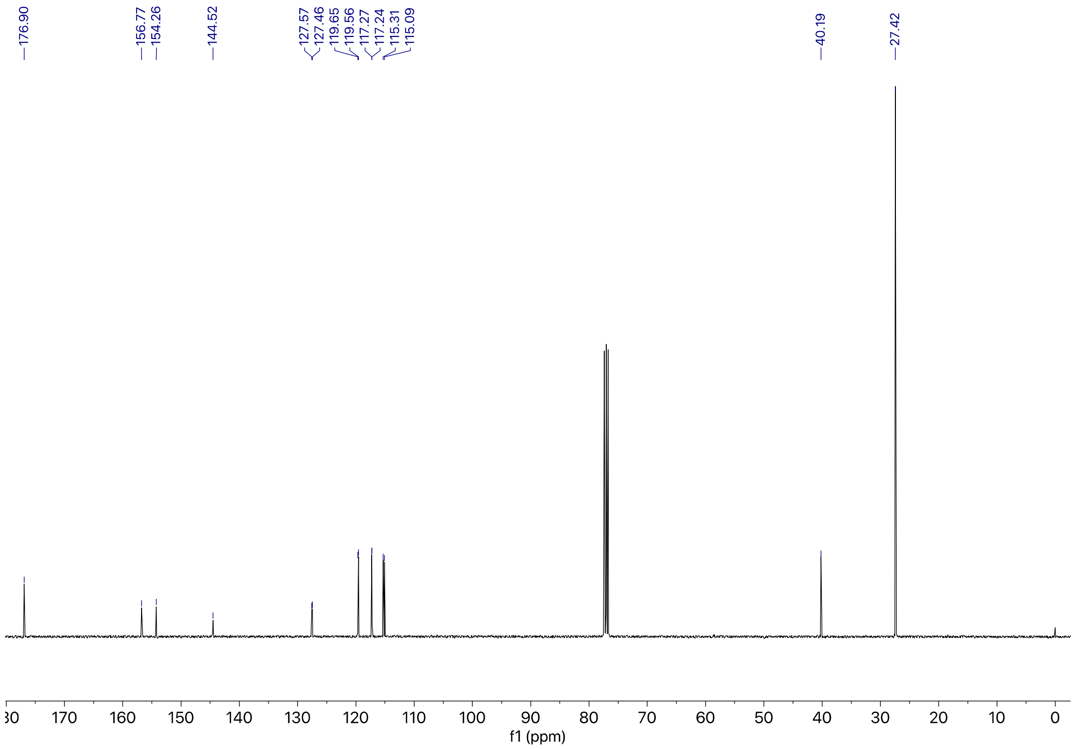

^{1}H and ^{13}C NMR for *N*-(2-Fluoro-5-nitrophenyl)benzamide (**8**)

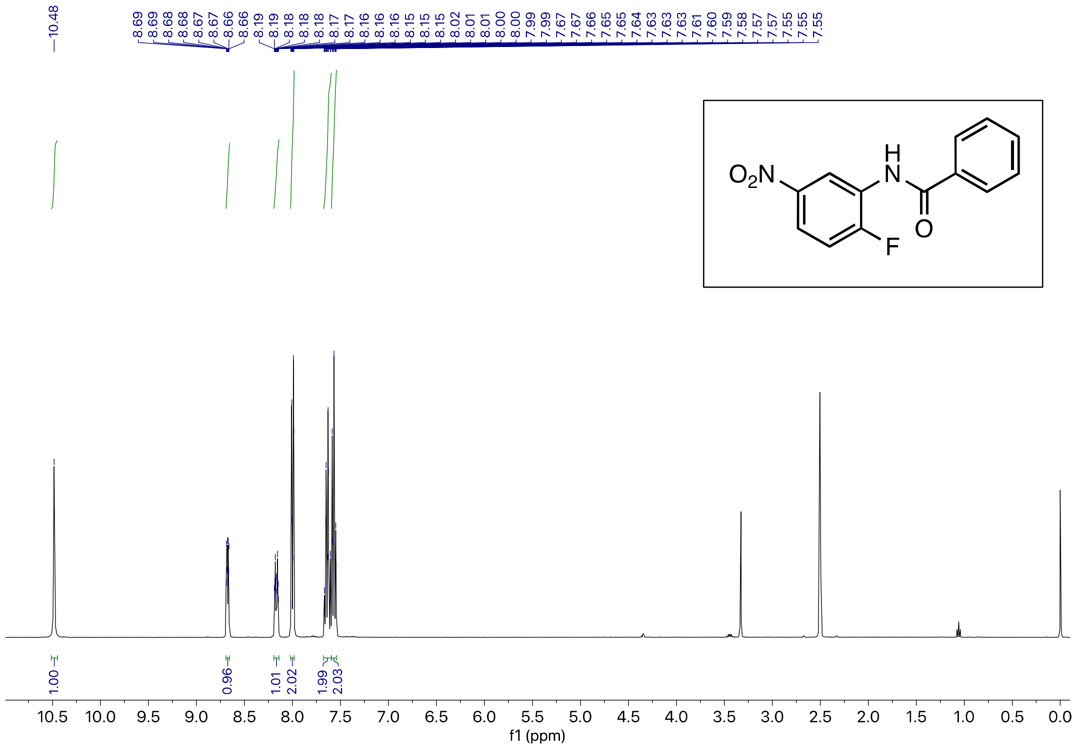

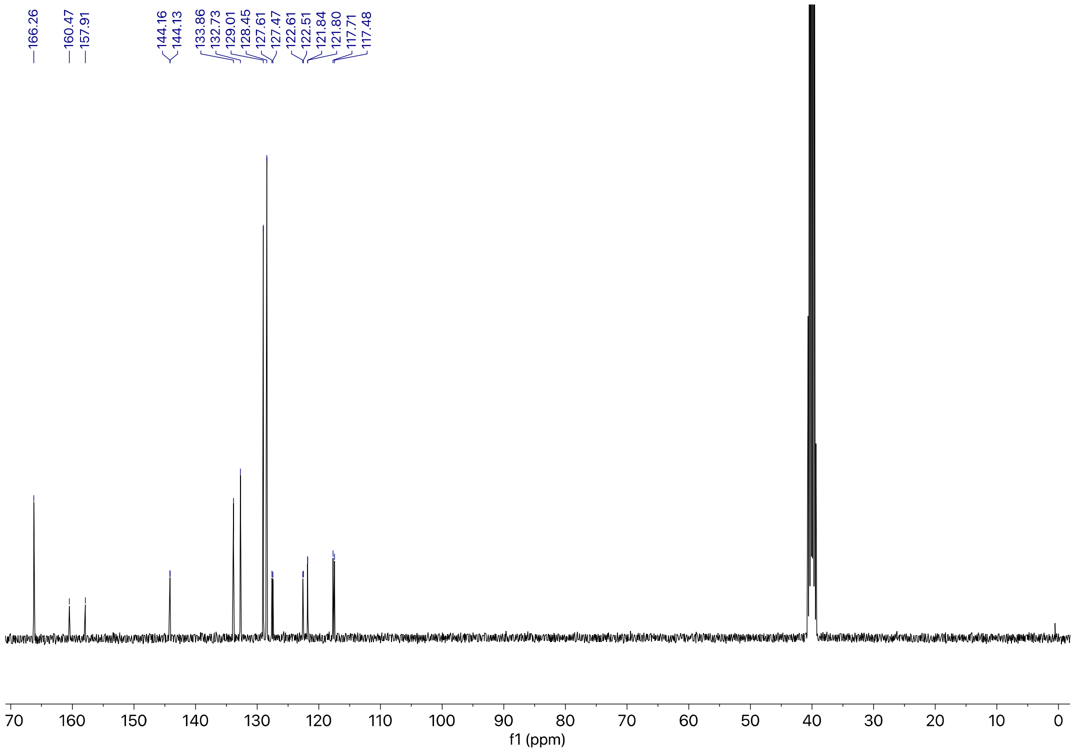

^{1}H and ^{13}C NMR for *N*-(2-Fluoro-5-nitrophenyl)-3-methylbenzamide (**9**)

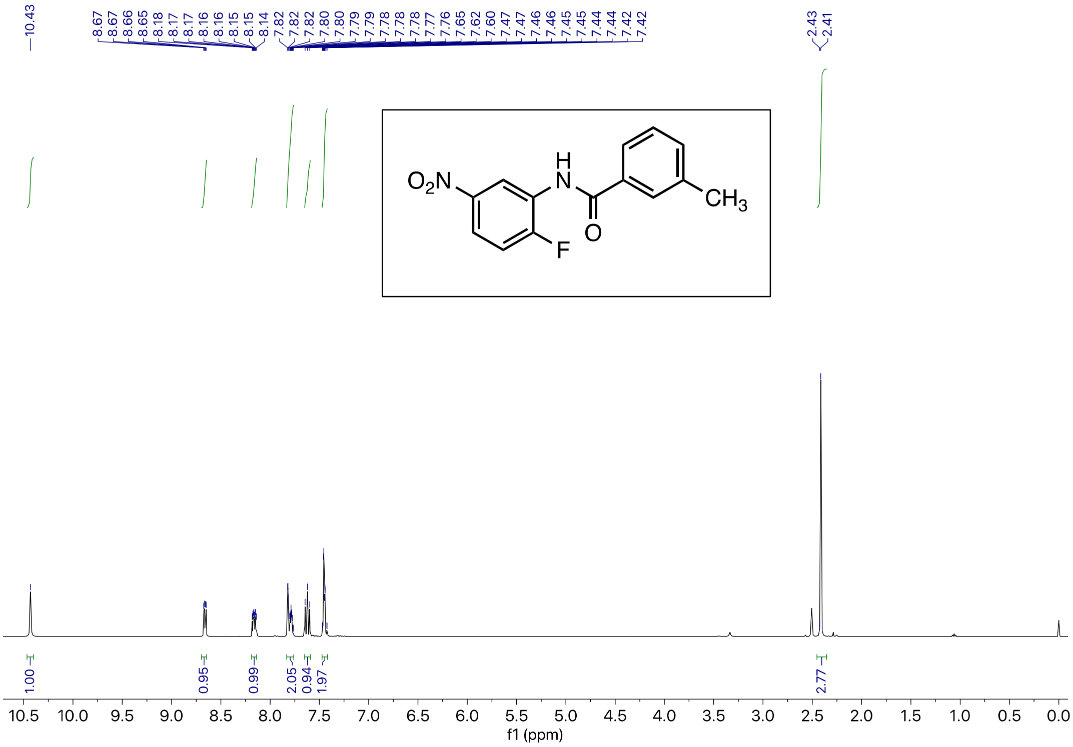

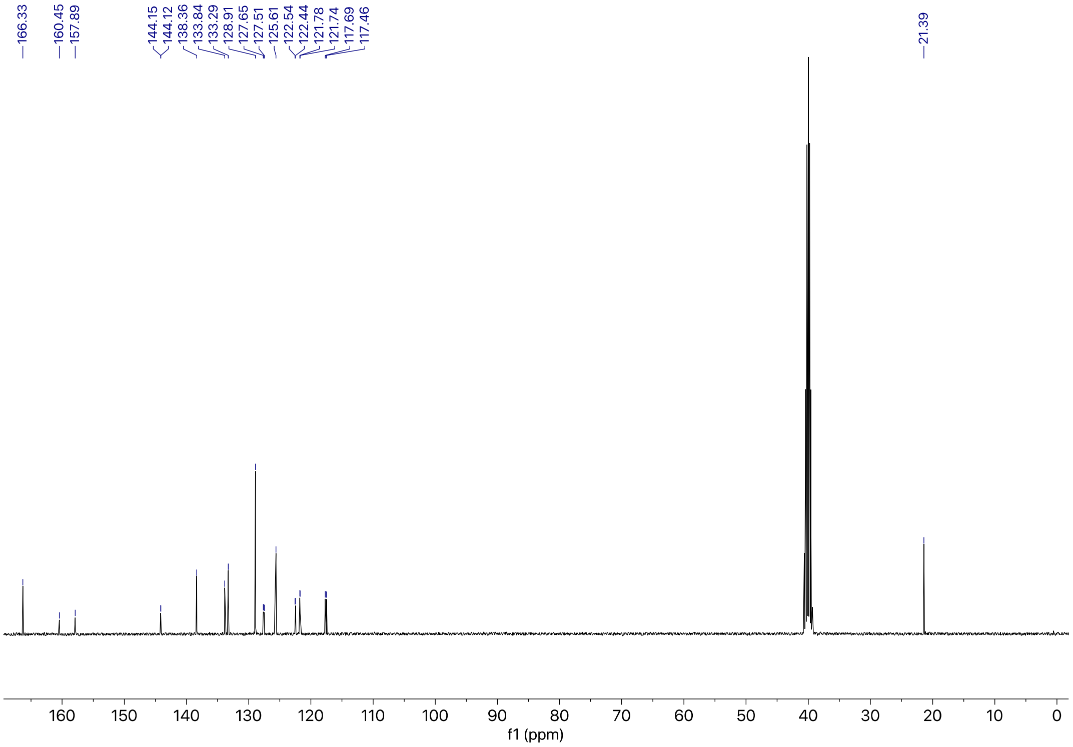

^{1}H and ^{13}C NMR for *N*-(2-Fluoro-5-nitrophenyl)-4-methylbenzamide (**10**)

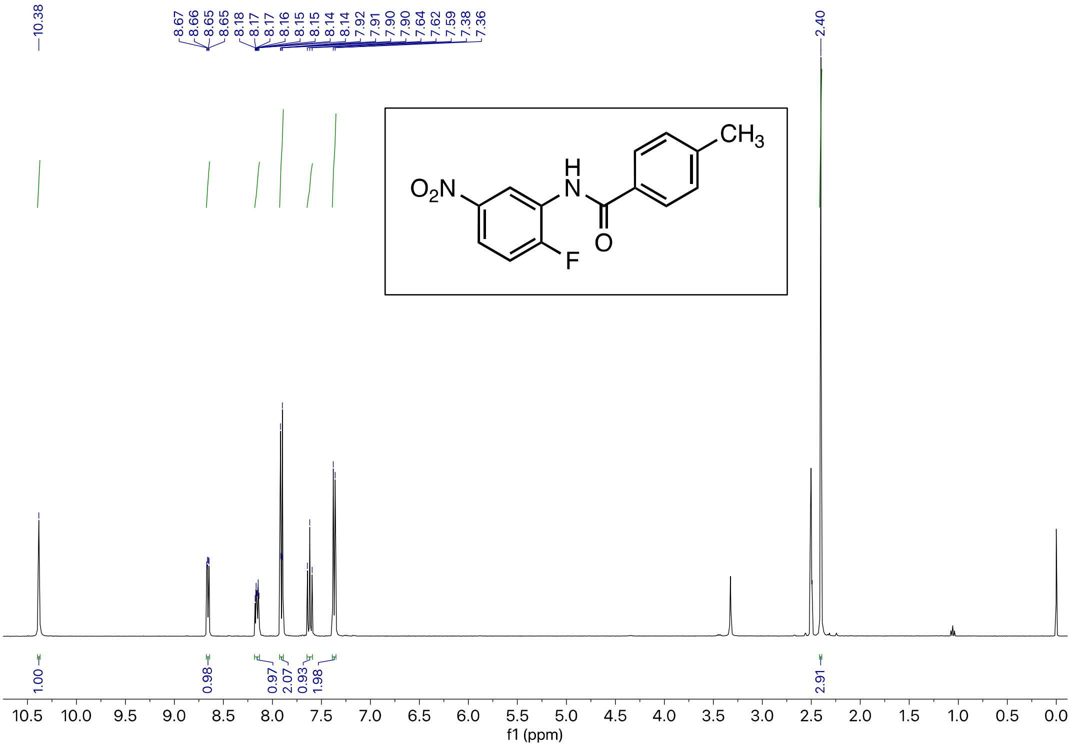

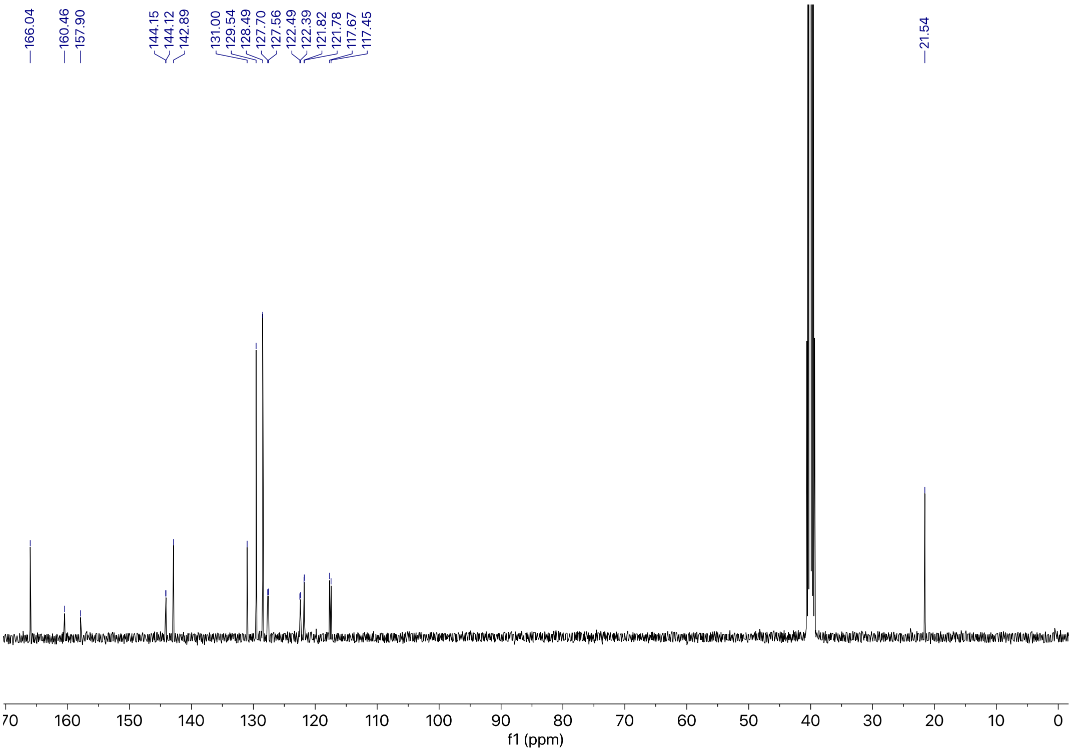

^{1}H and ^{13}C NMR for *N*-(2-Fluoro-5-nitrophenyl)-4-methoxybenzamide (**11**)

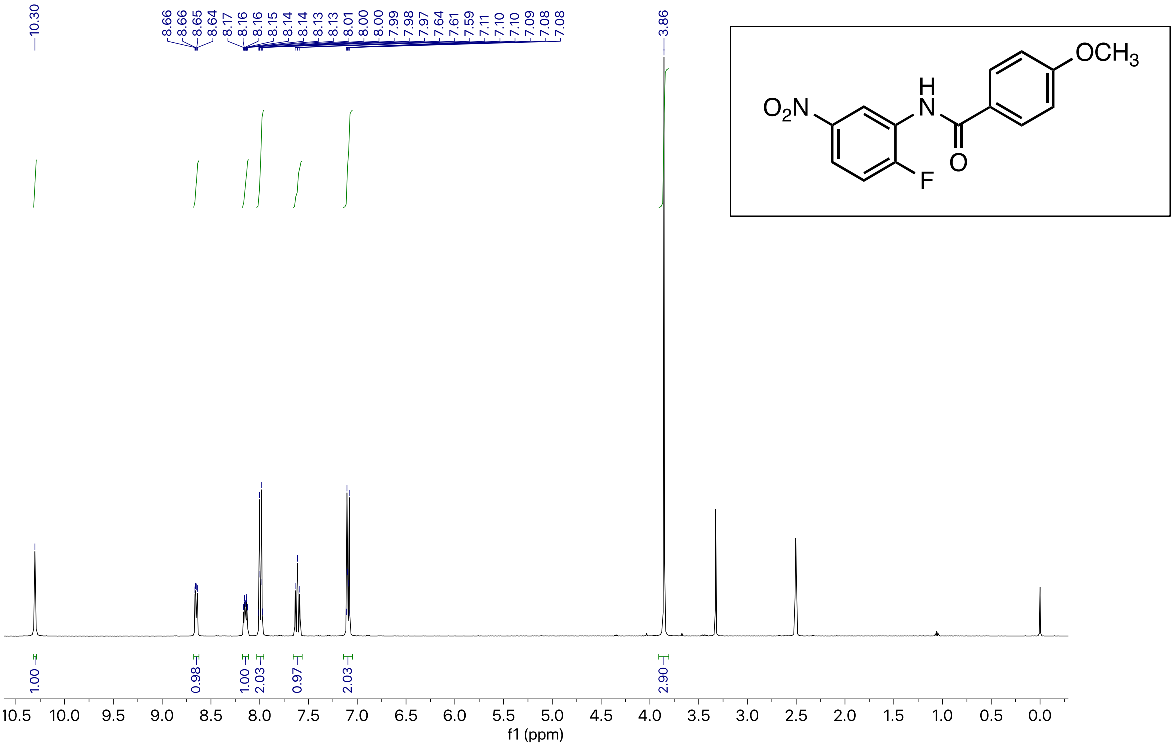

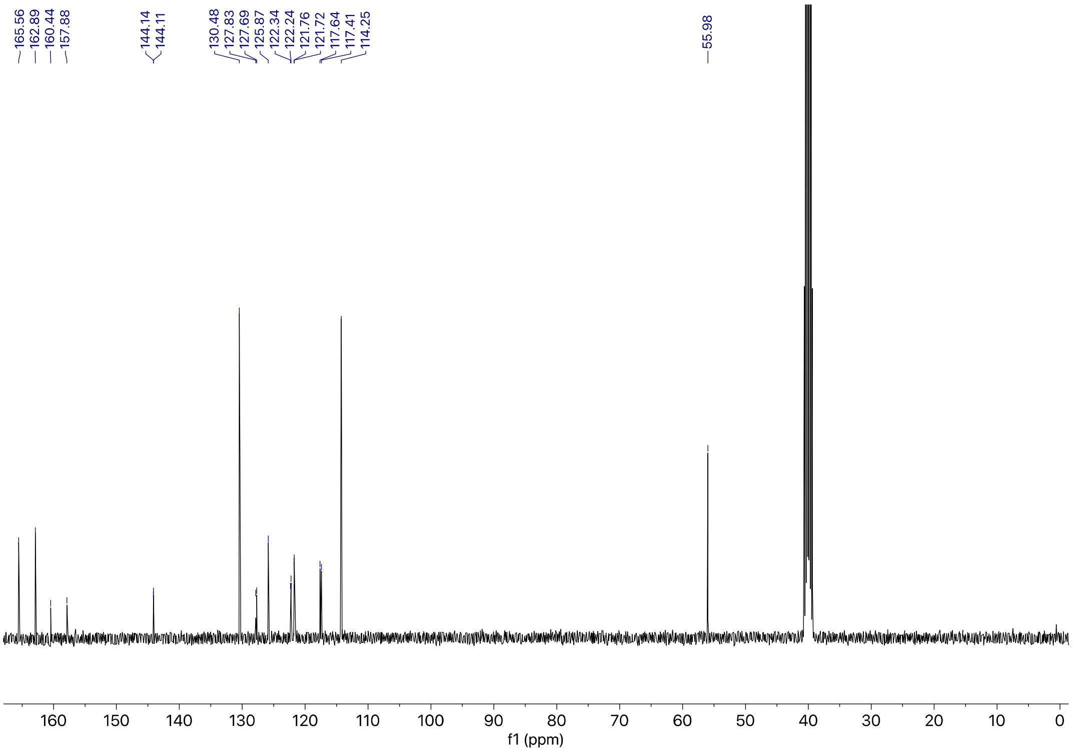

^{1}H and ^{13}C NMR for 2-Fluoro-*N*-(2-fluoro-5-nitrophenyl)benzamide (**12**)

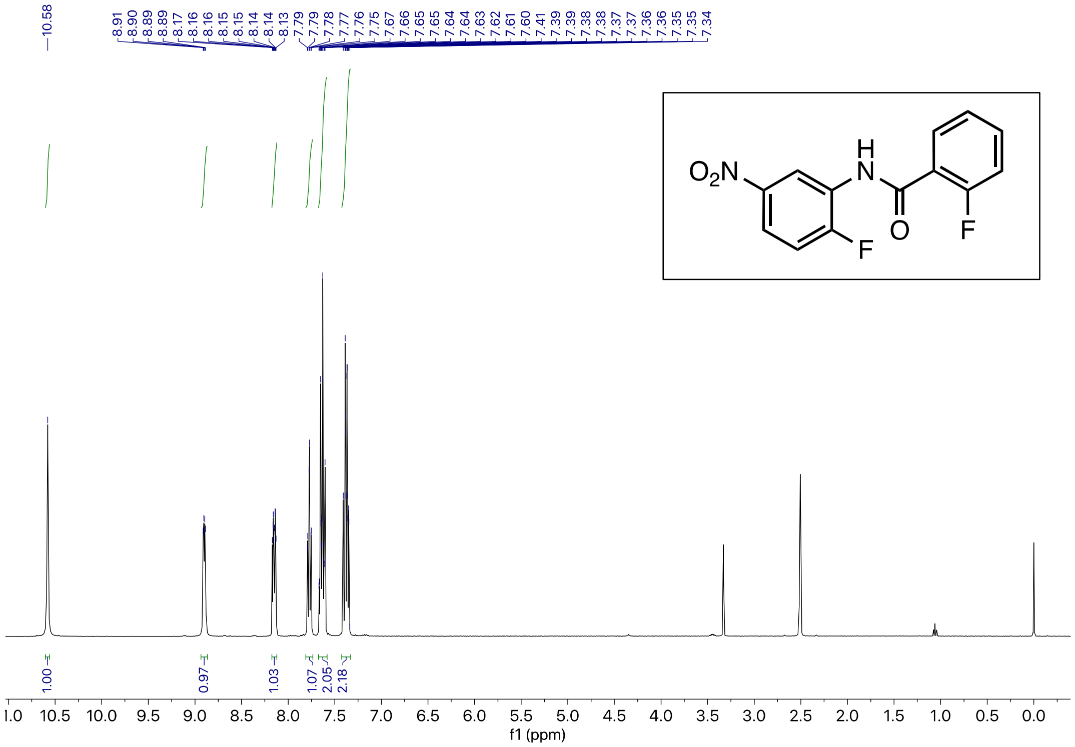

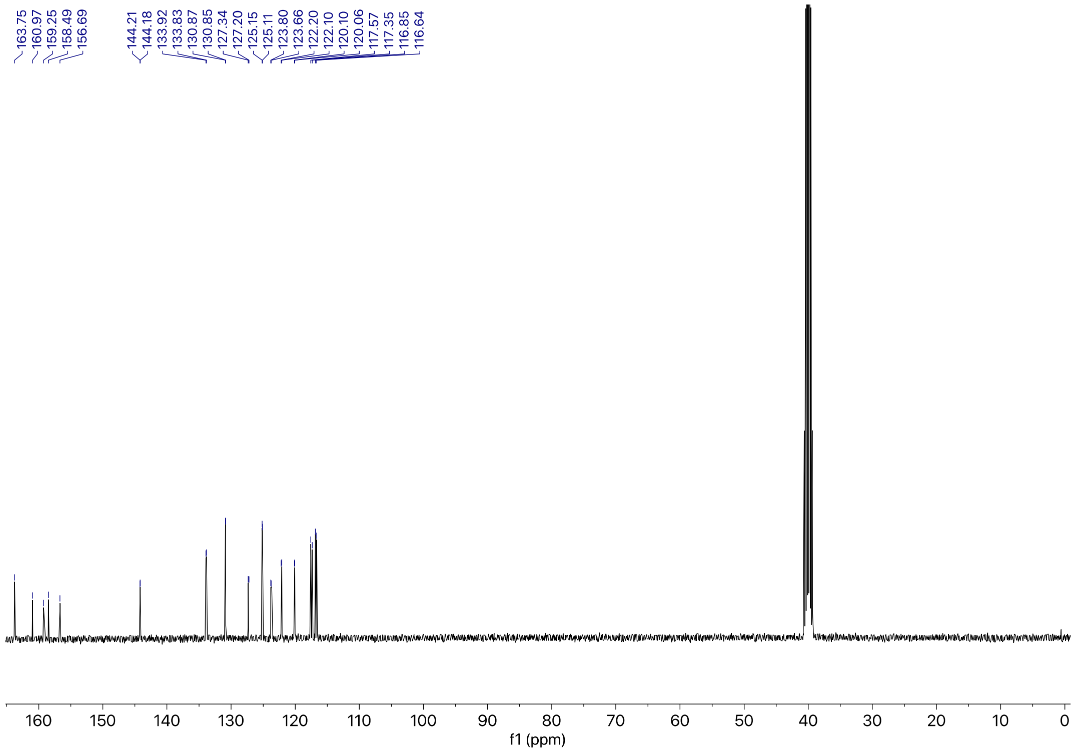

^{19}F NMR for 2-Fluoro-*N*-(2-fluoro-5-nitrophenyl)benzamide (**12**)

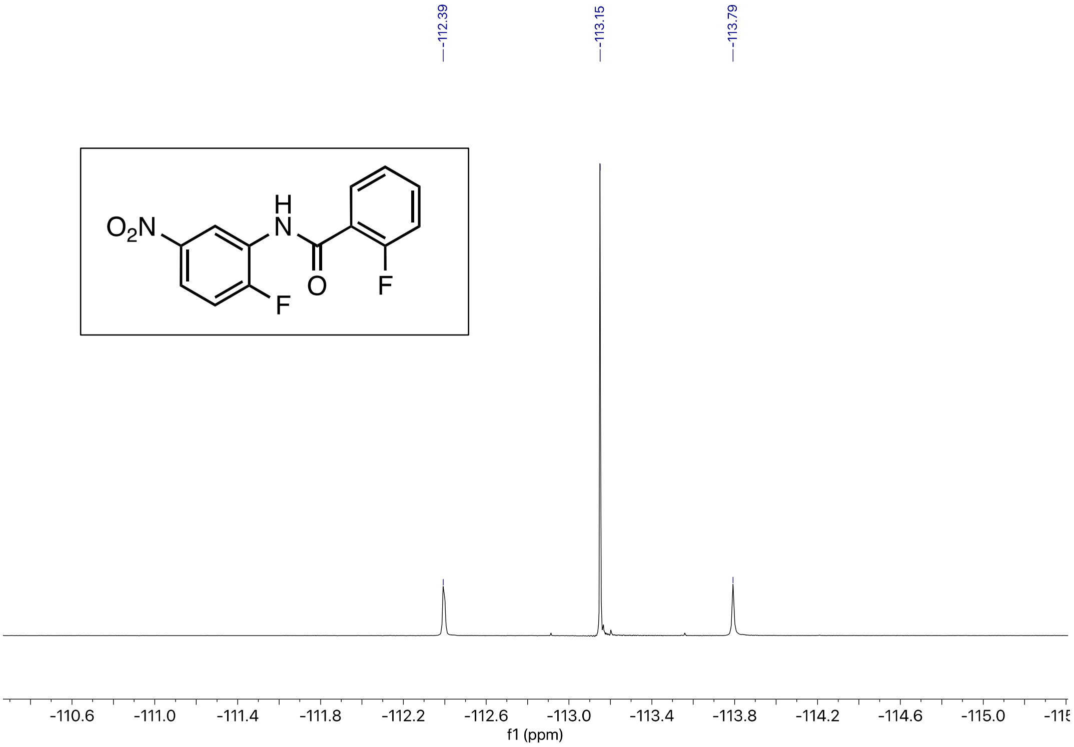

^{1}H and ^{13}C NMR for 3-Chloro-*N*-(2-fluoro-5-nitrophenyl)benzamide (**13**)

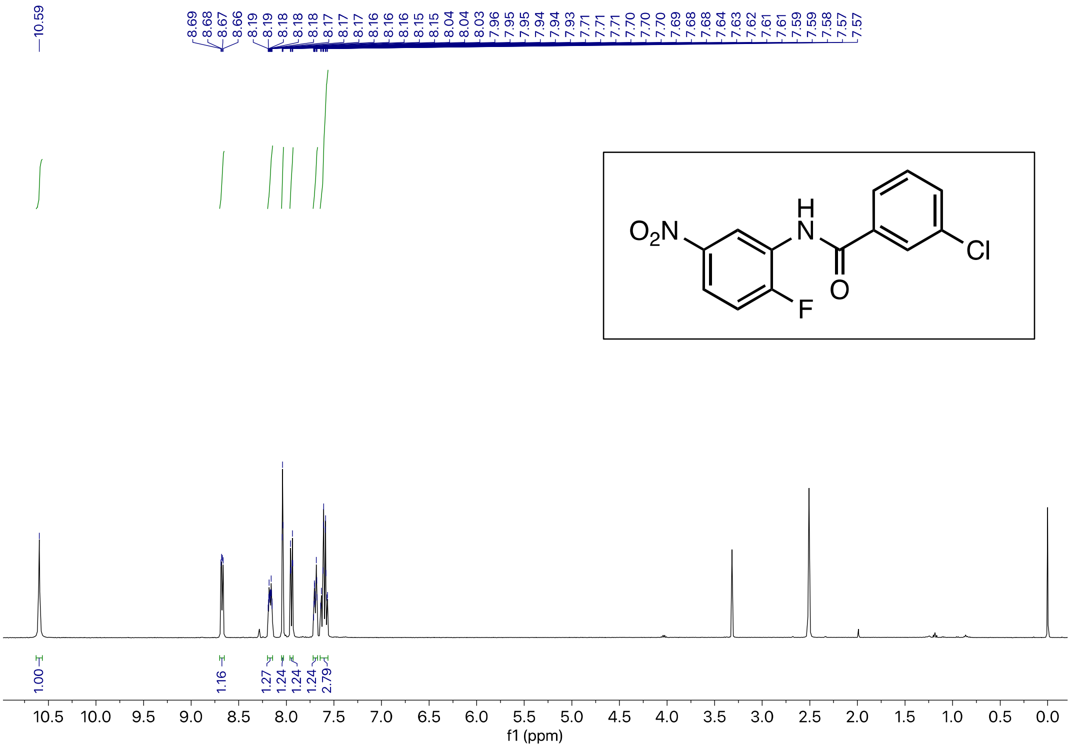

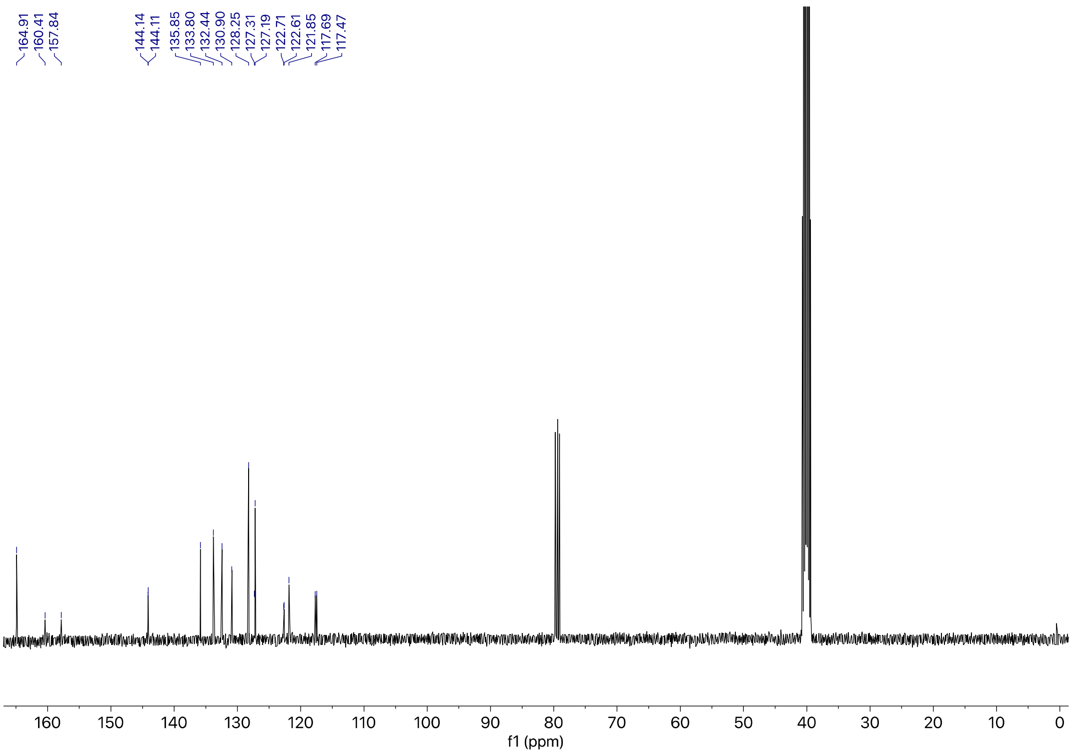

^{1}H and ^{13}C NMR for 4-Chloro-*N*-(2-fluoro-5-nitrophenyl)benzamide (**14**)

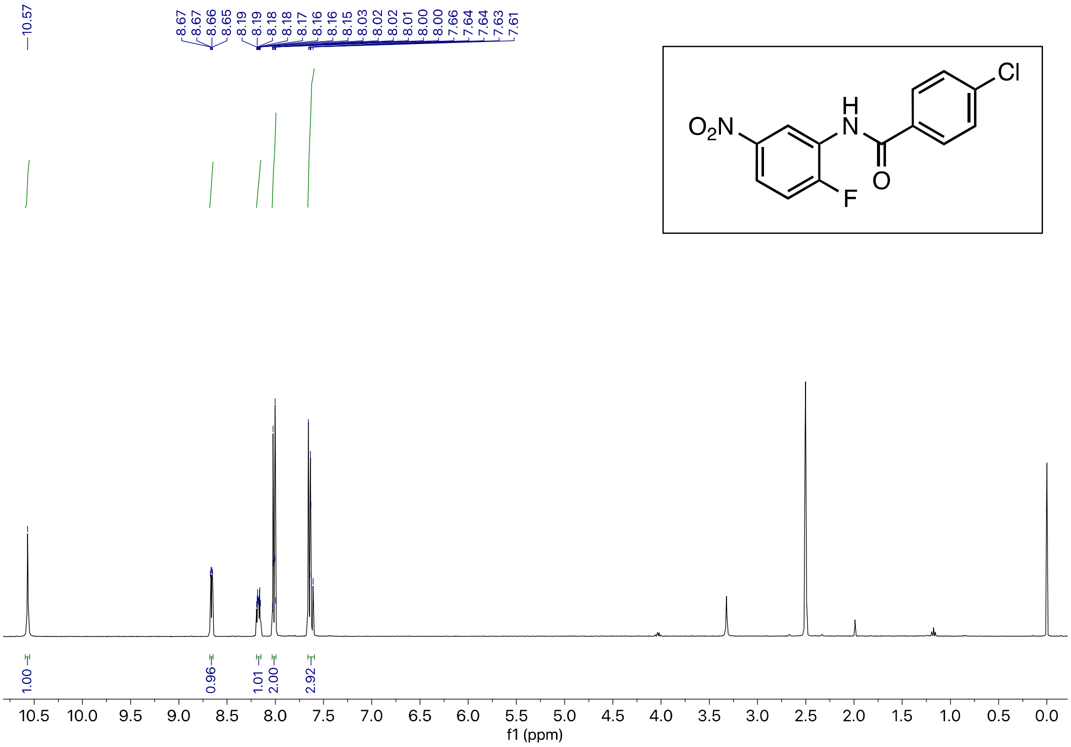

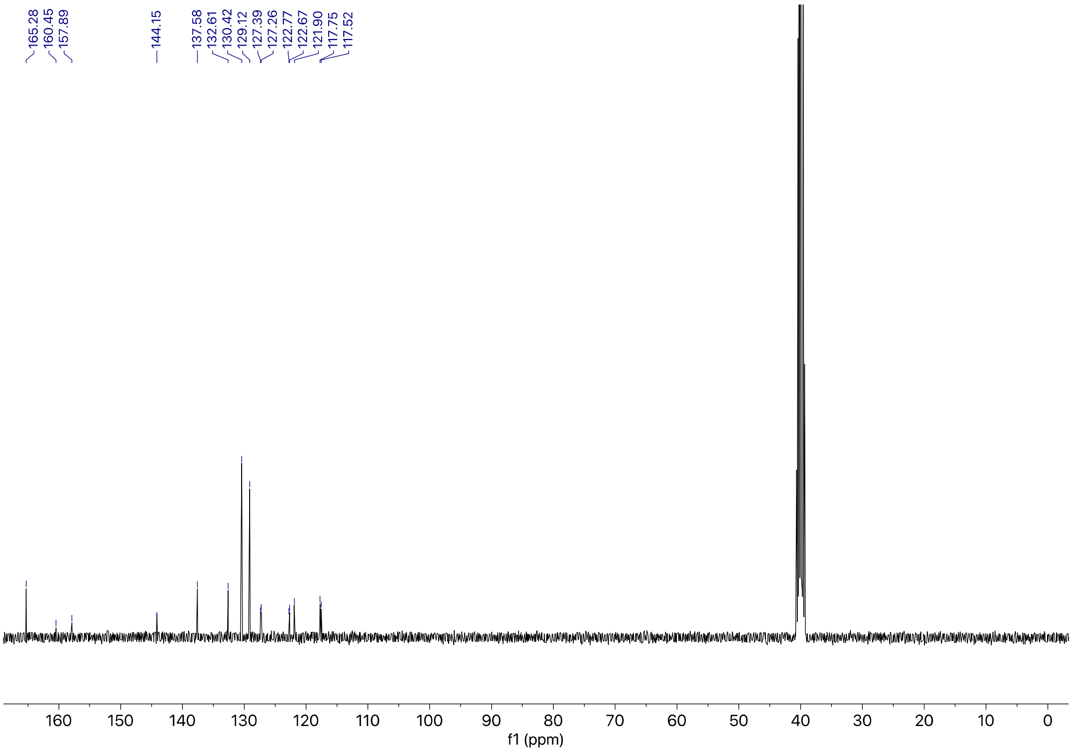

^{1}H and ^{13}C NMR for *N*-(5-Cyano-2-fluorophenyl)acetamide (**15**)

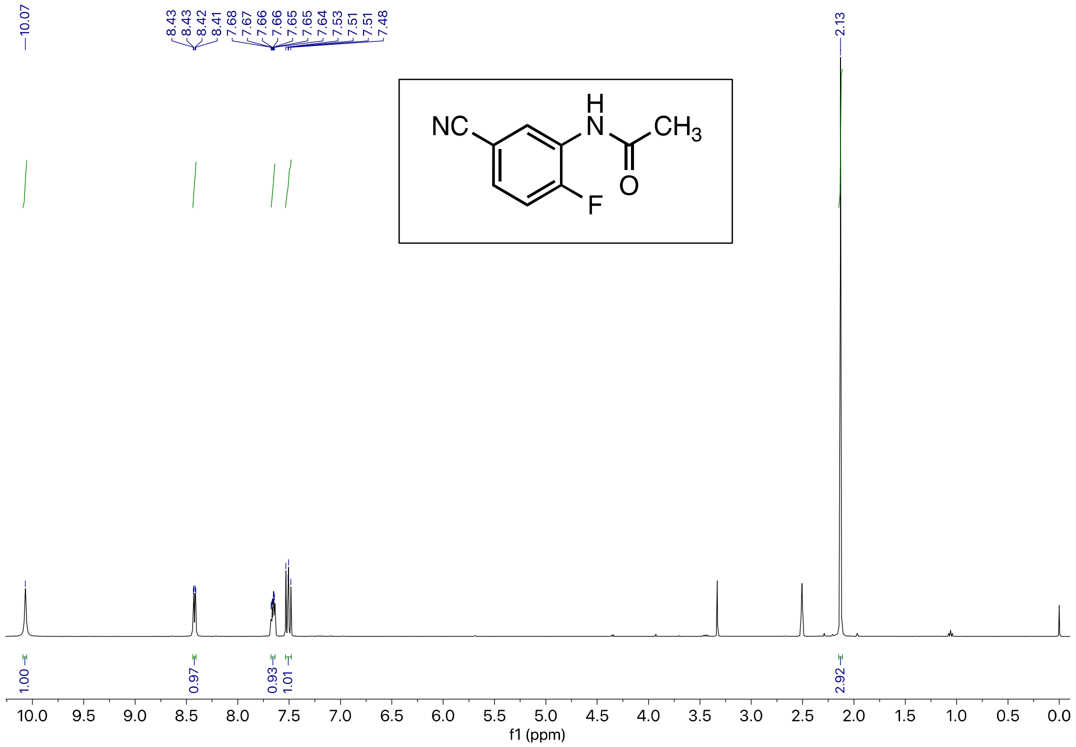

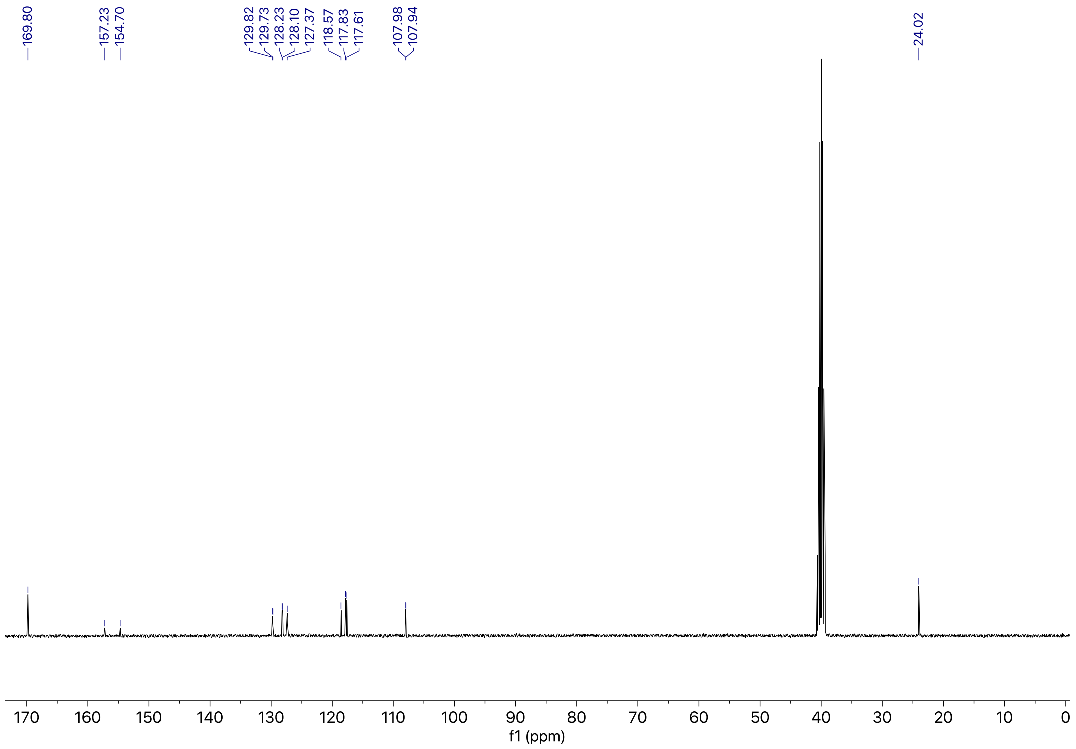

^{1}H and ^{13}C NMR for *N*-(5-Cyano-2-fluorophenyl)hexanamide (**16**)

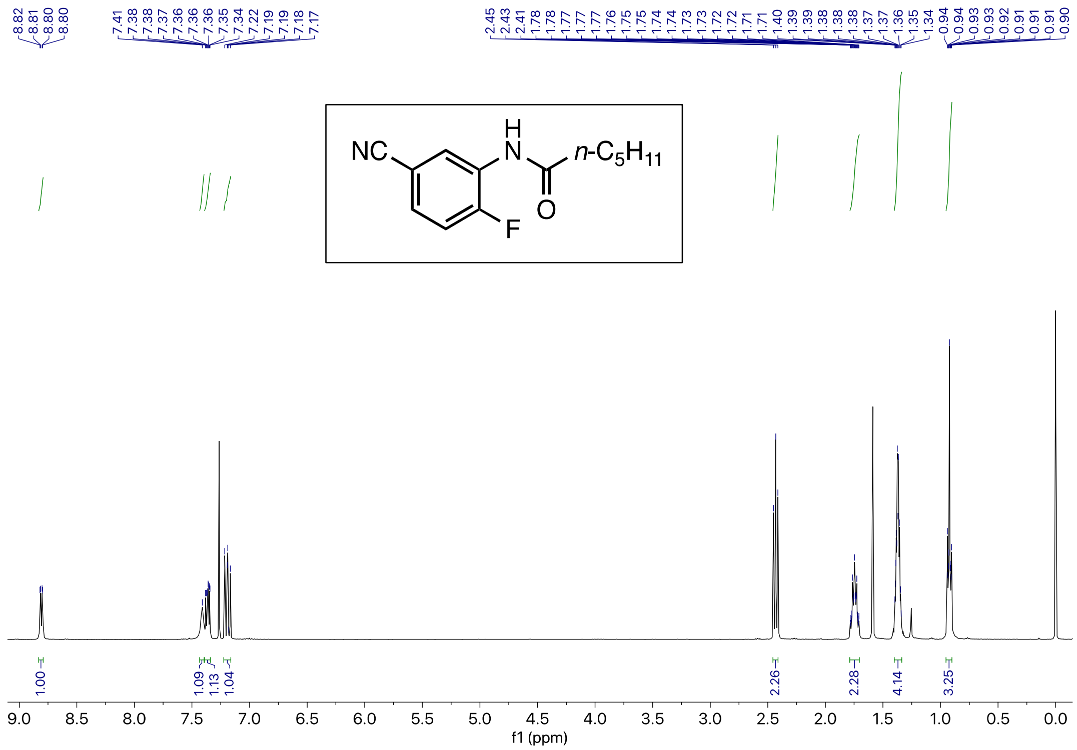

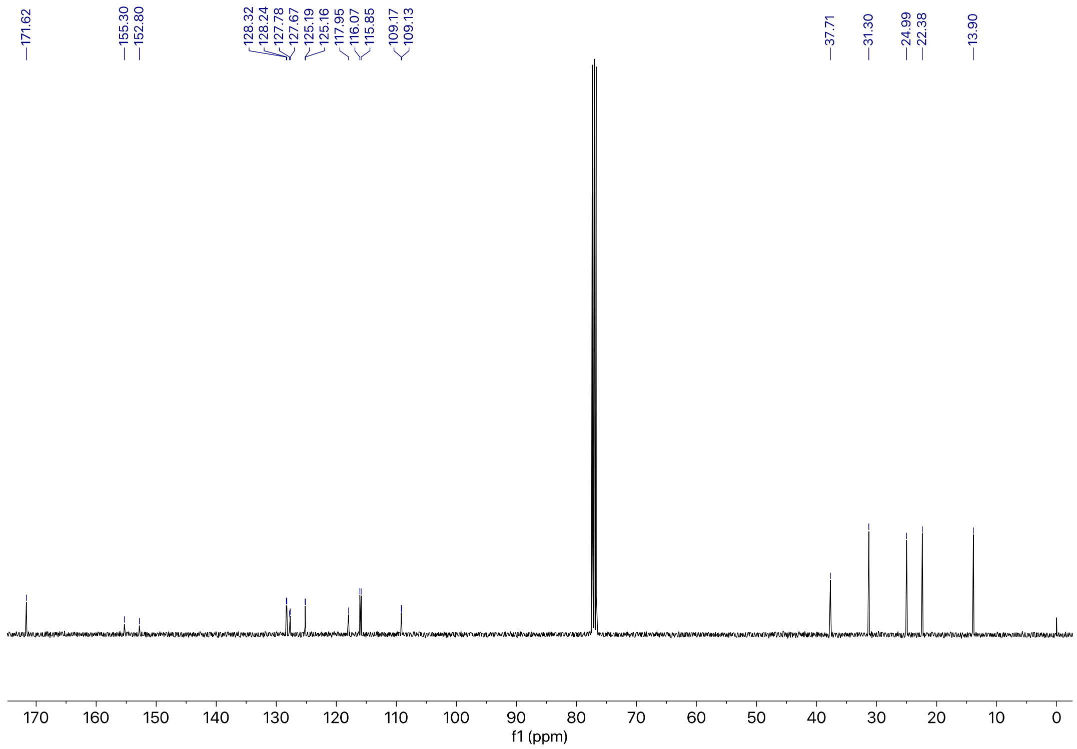

¹H and ¹³C NMR for *N*-(5-Cyano-2-fluorophenyl)pivalamide (**17**)

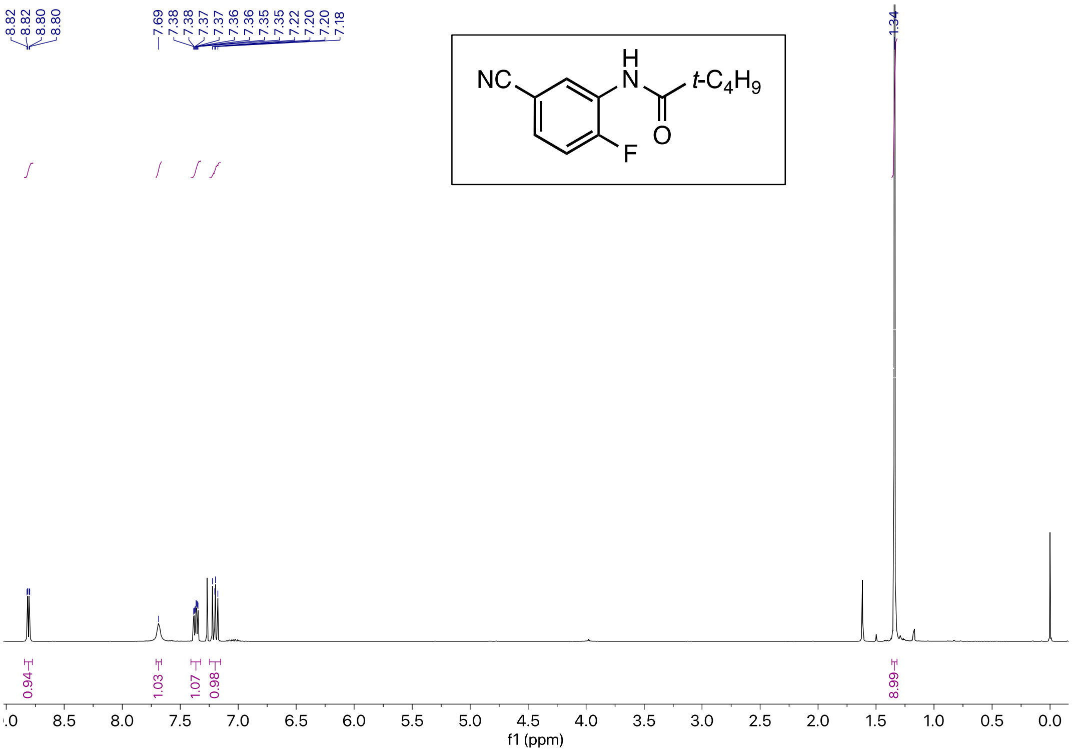

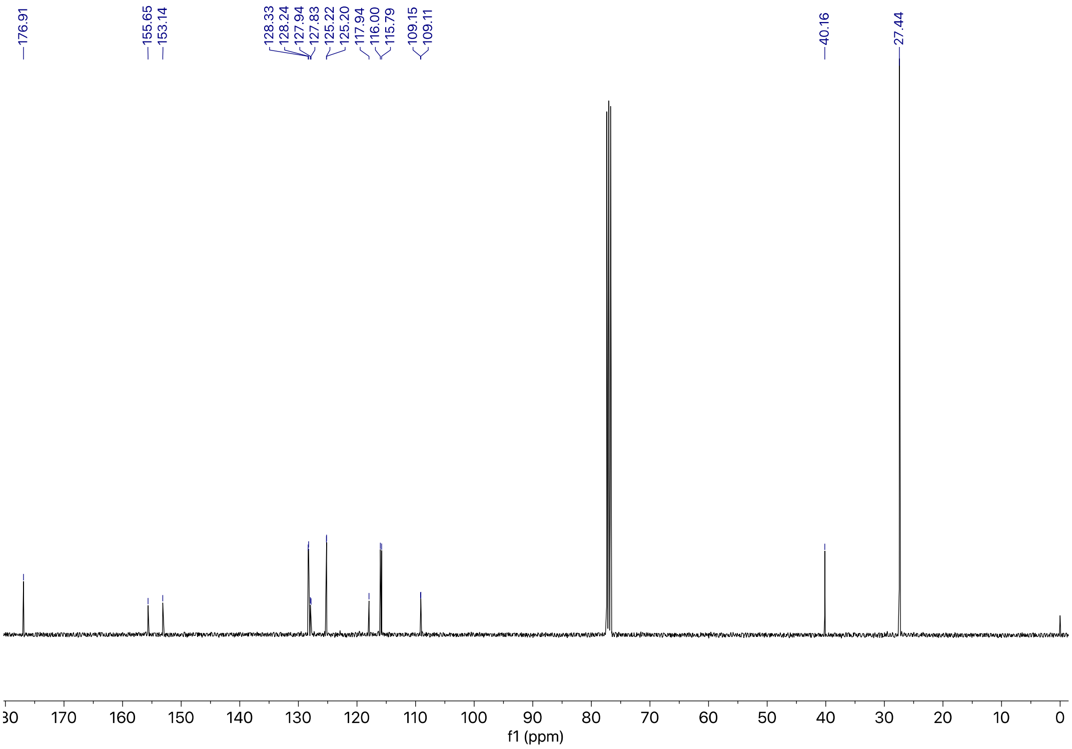

^{1}H and ^{13}C NMR for *N*-(5-Cyano-2-fluorophenyl)benzamide (**18**)

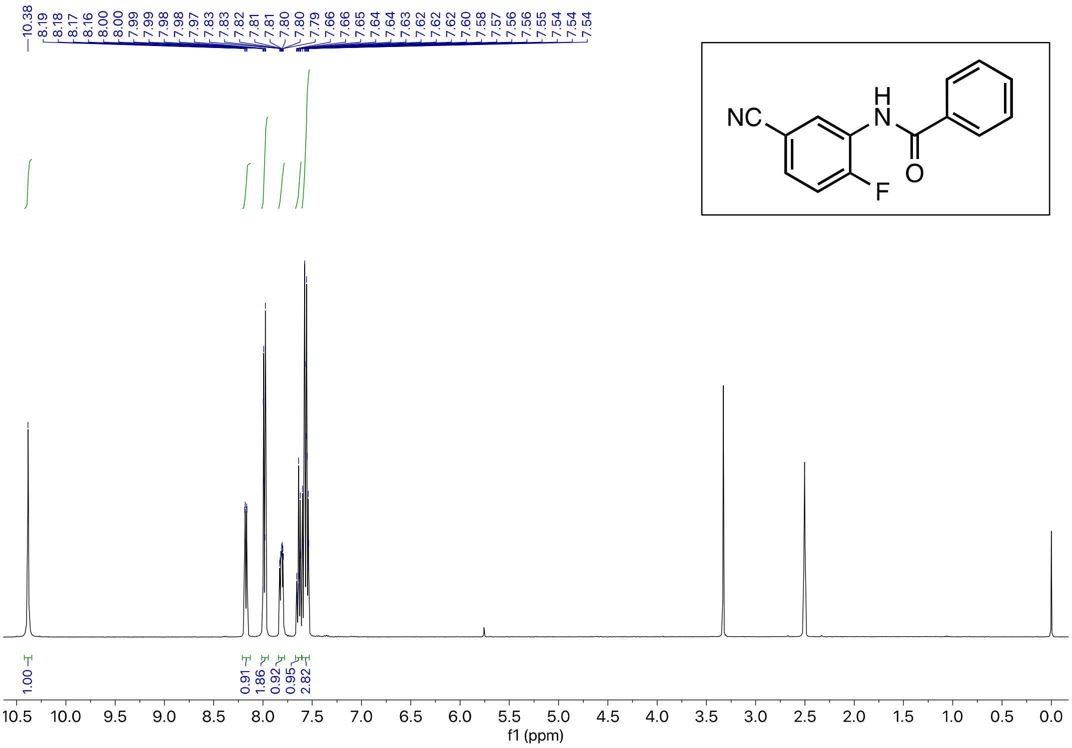

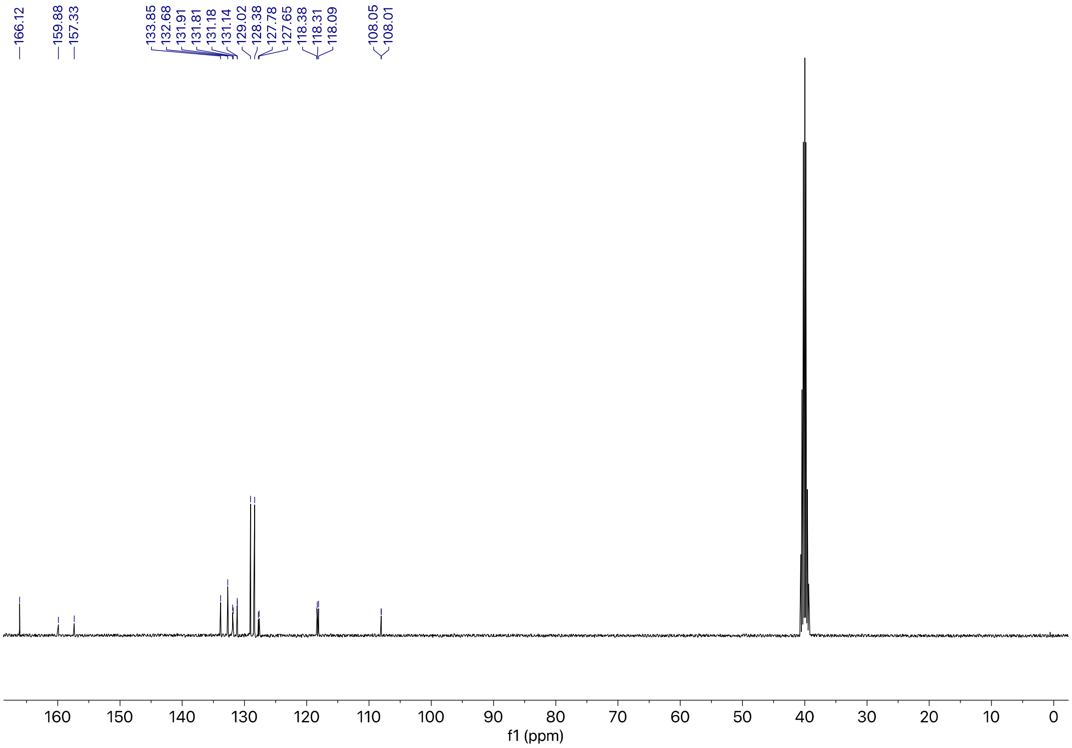

^{1}H and ^{13}C NMR for *N*-(5-Cyano-2-fluorophenyl)-3-methylbenzamide (**19**)

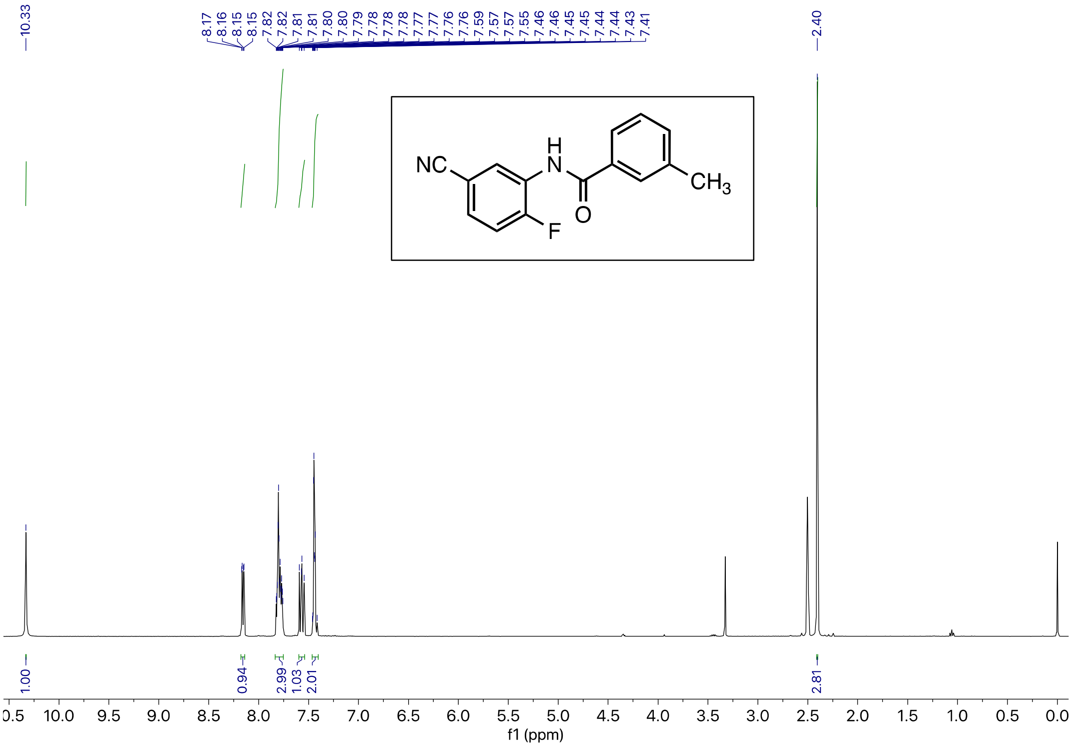

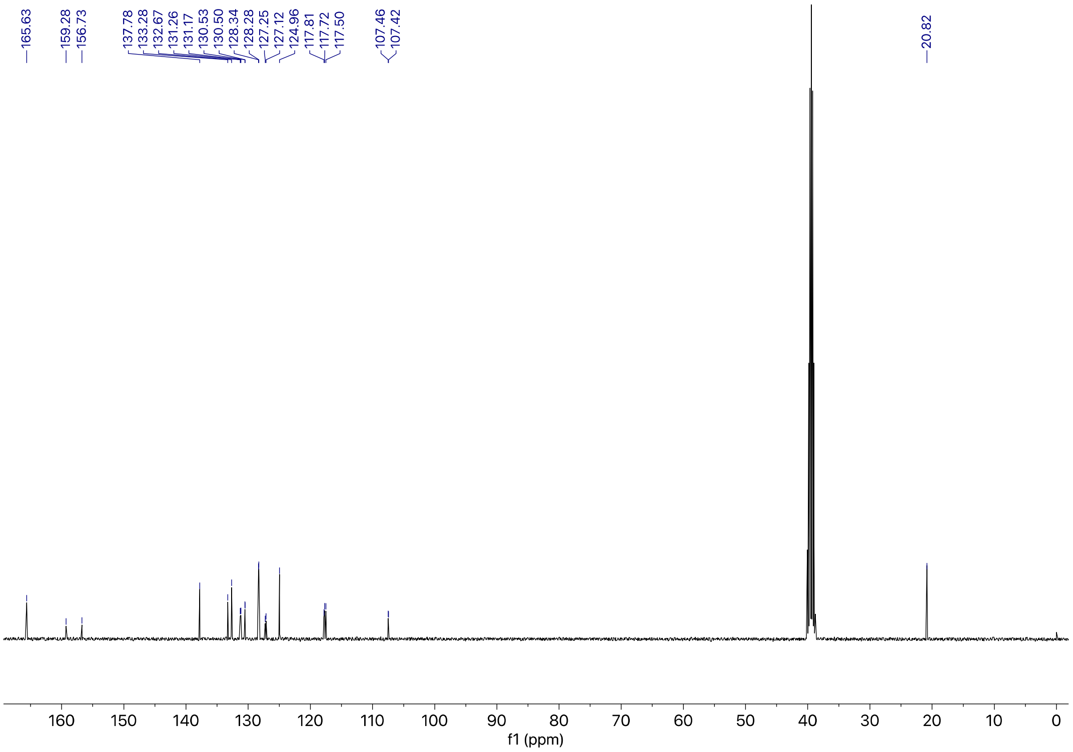

^{1}H and ^{13}C NMR for *N*-(5-Cyano-2-fluorophenyl)-4-methylbenzamide (**20**)

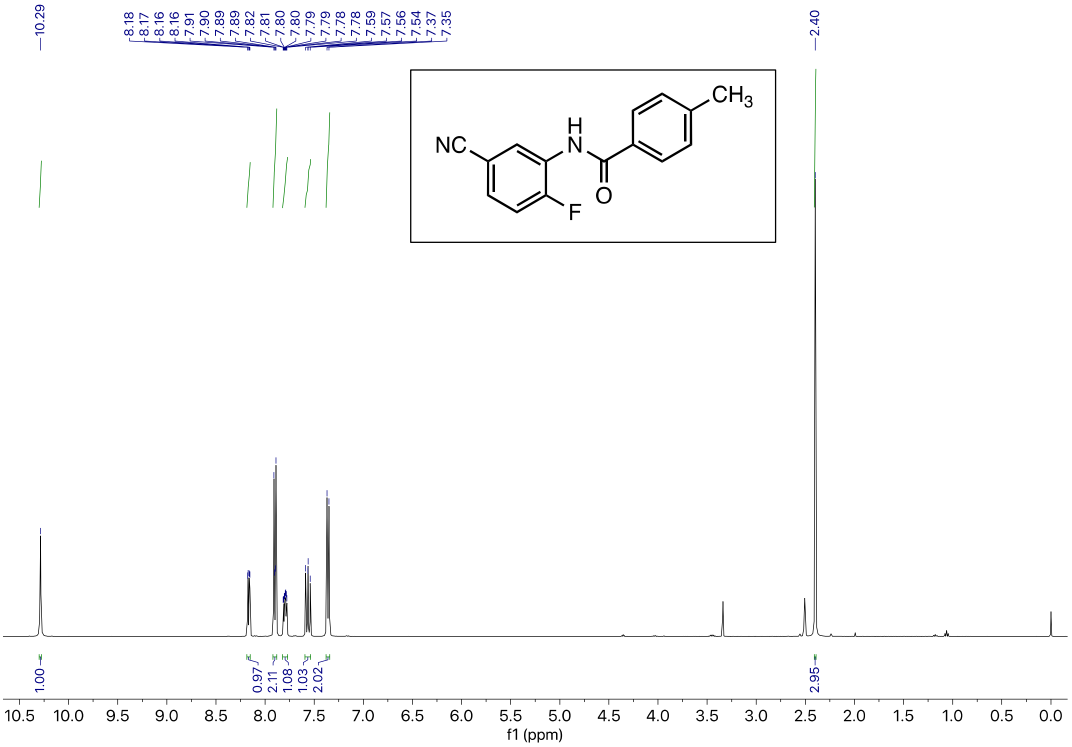

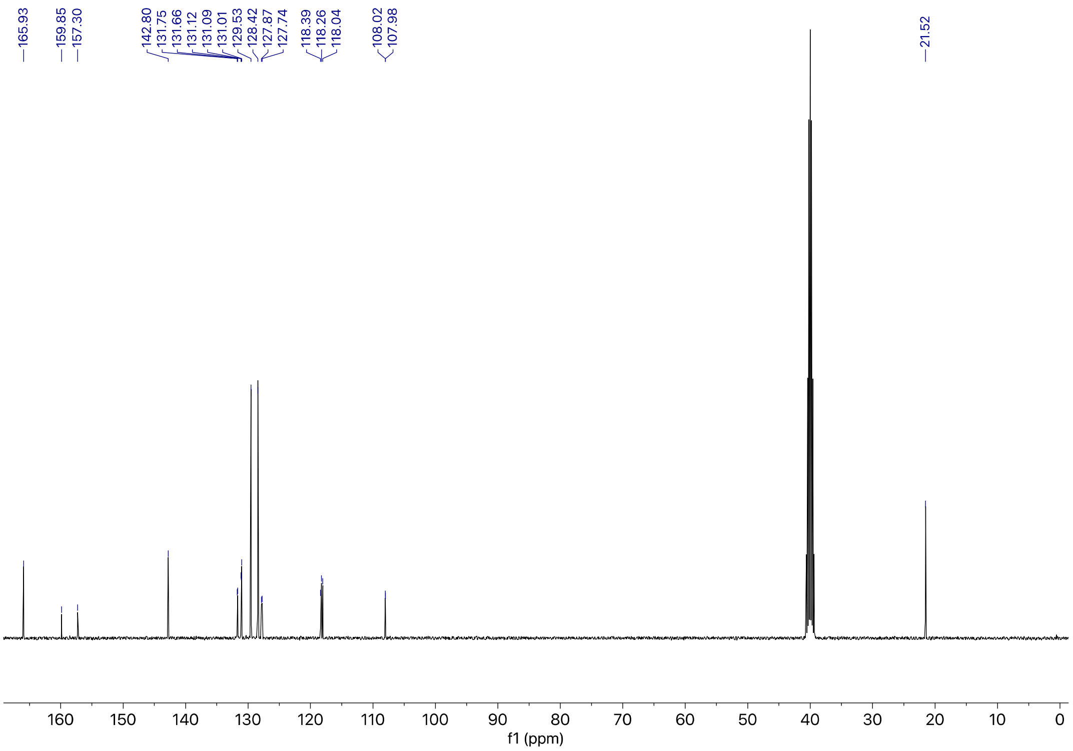

^{1}H and ^{13}C NMR for *N*-(5-Cyano-2-fluorophenyl)-4-methoxybenzamide (**21**)

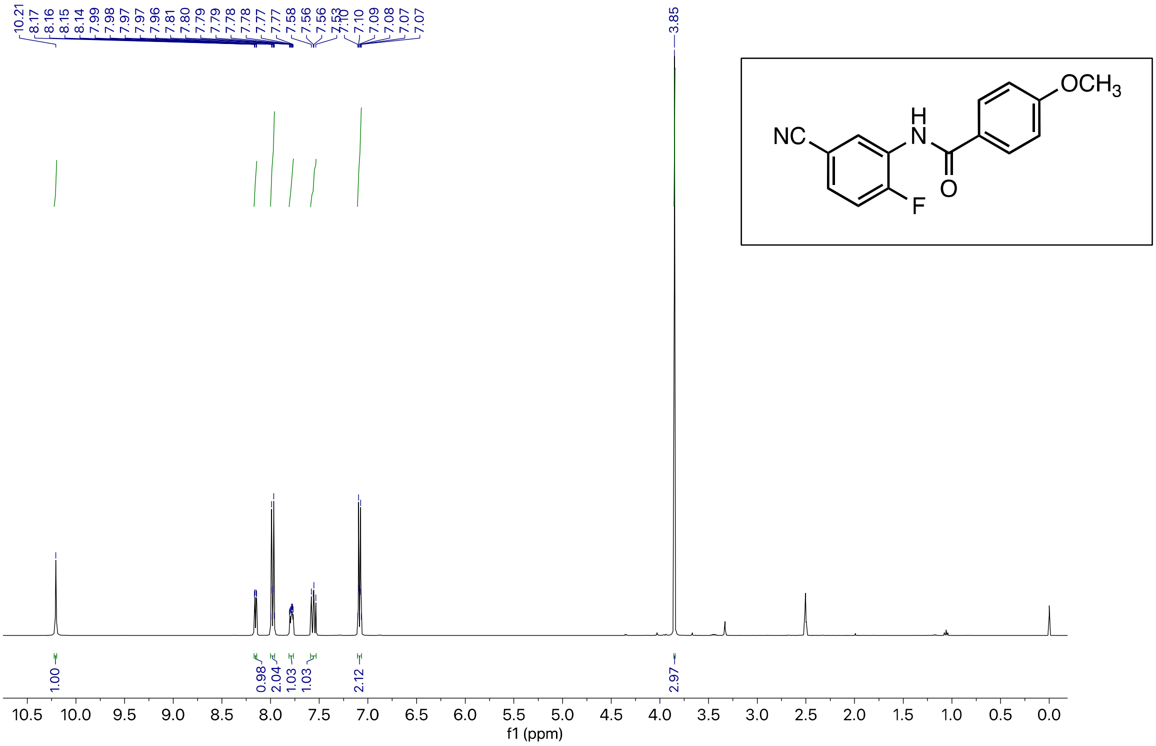

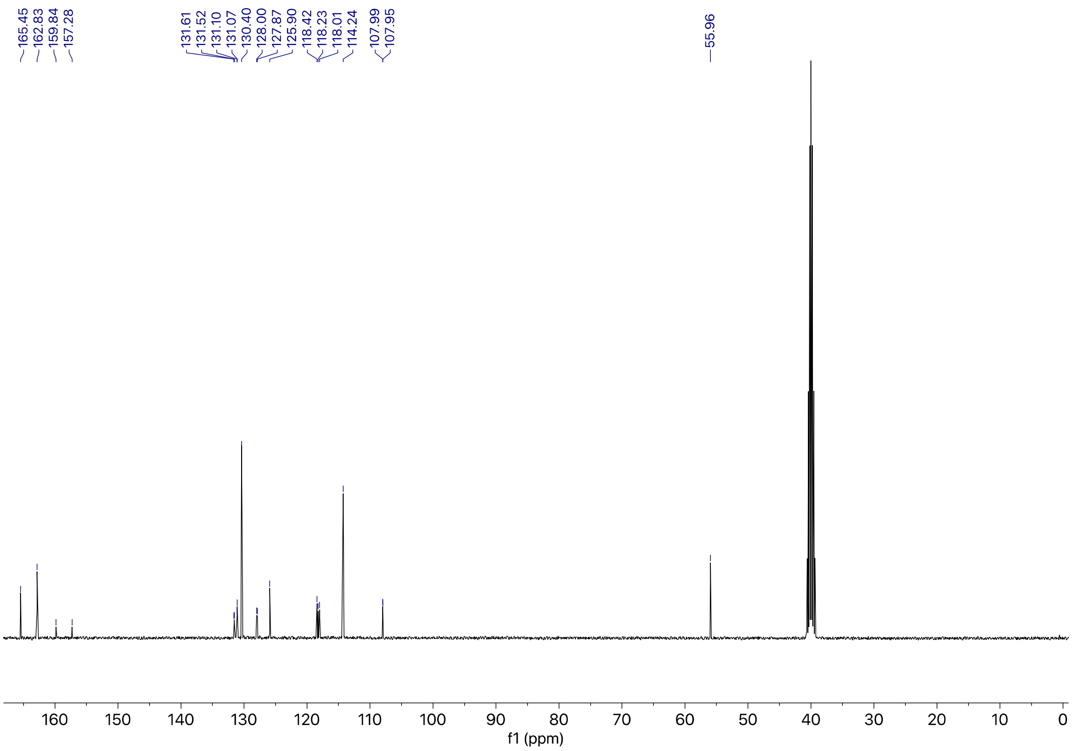

^{1}H and ^{13}C NMR for *N*-(5-Cyano-2-fluorophenyl)-2-fluorobenzamide (**22**)

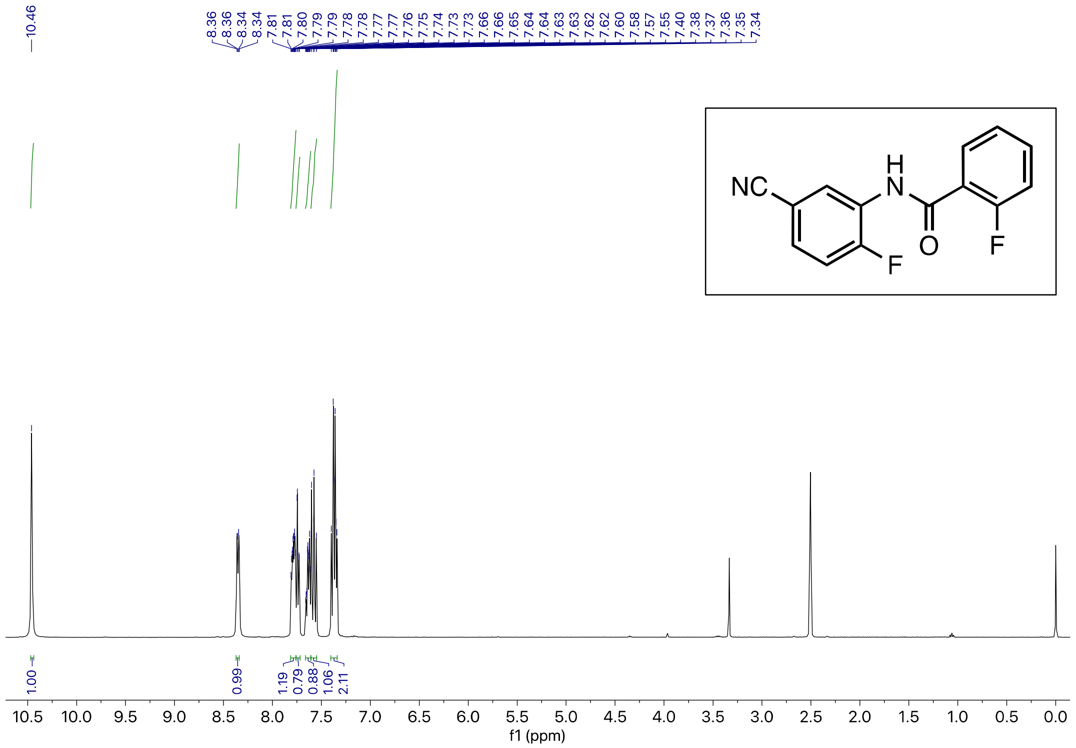

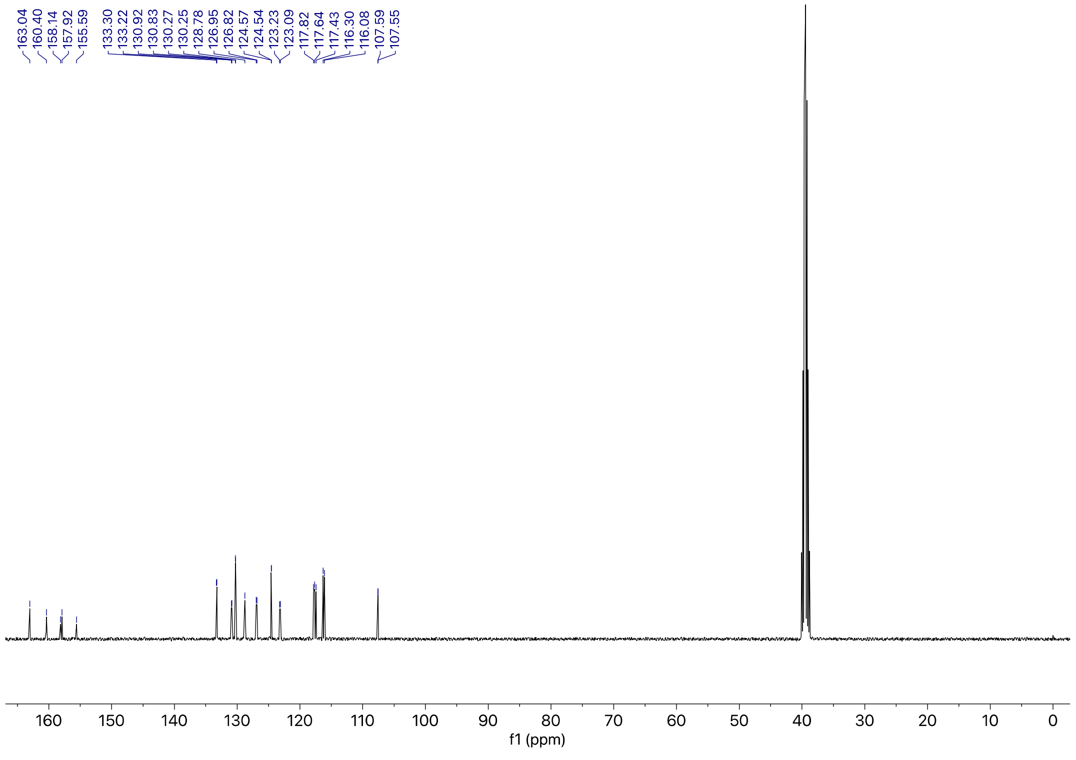

¹⁹F NMR for *N*-(5-Cyano-2-fluorophenyl)-2-fluorobenzamide (**22**)

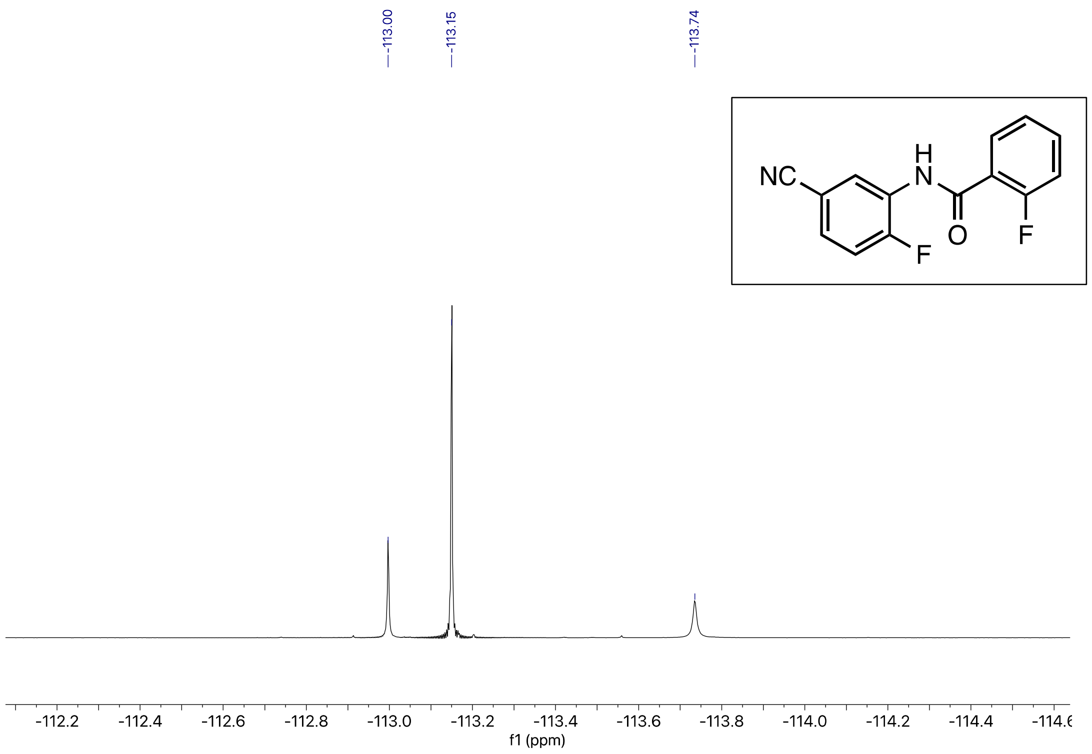

^{1}H and ^{13}C NMR for 3-Chloro-*N*-(5-cyano-2-fluorophenyl)benzamide (**23**)

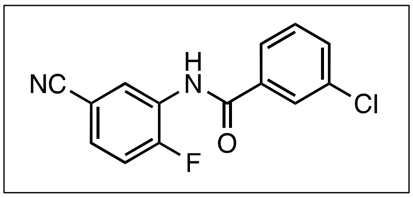

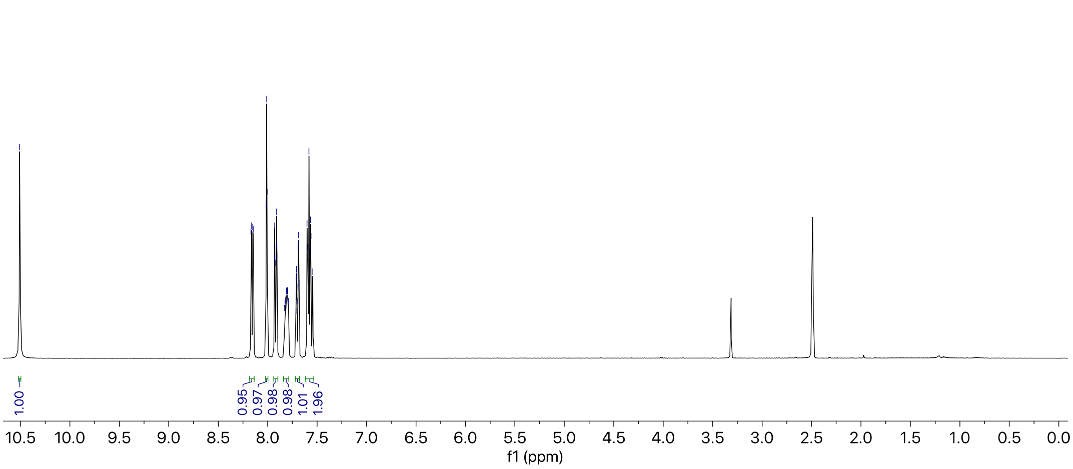

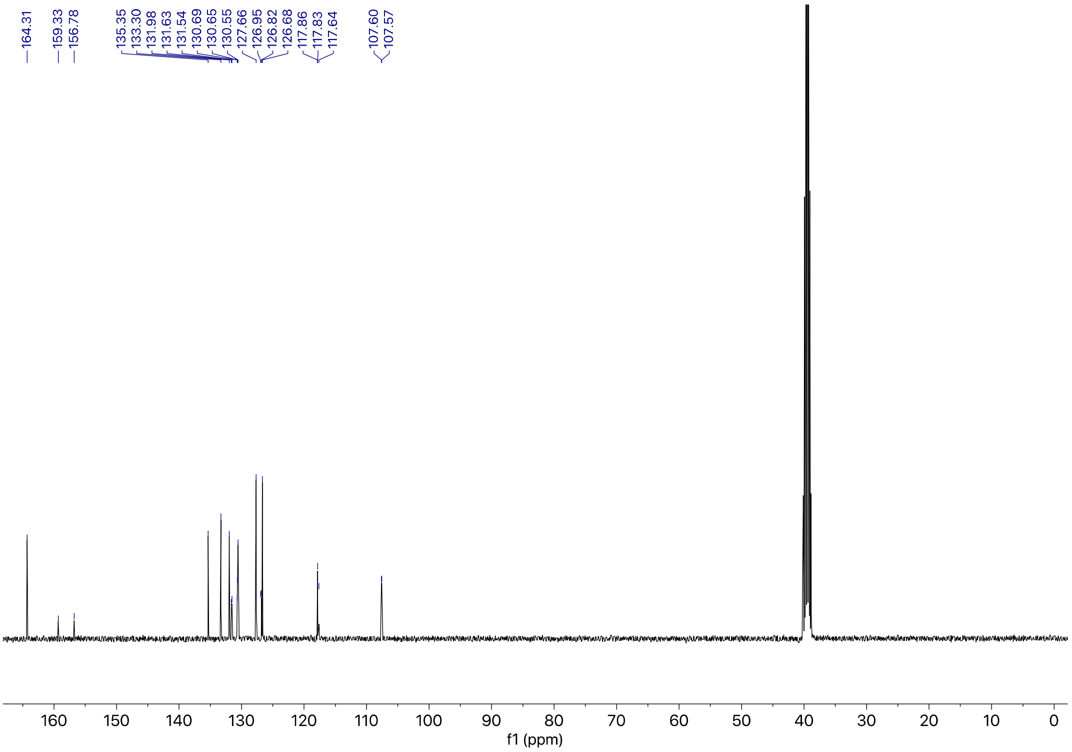

^{1}H and ^{13}C NMR for 4-Chloro-*N*-(5-cyano-2-fluorophenyl)benzamide (**24**)

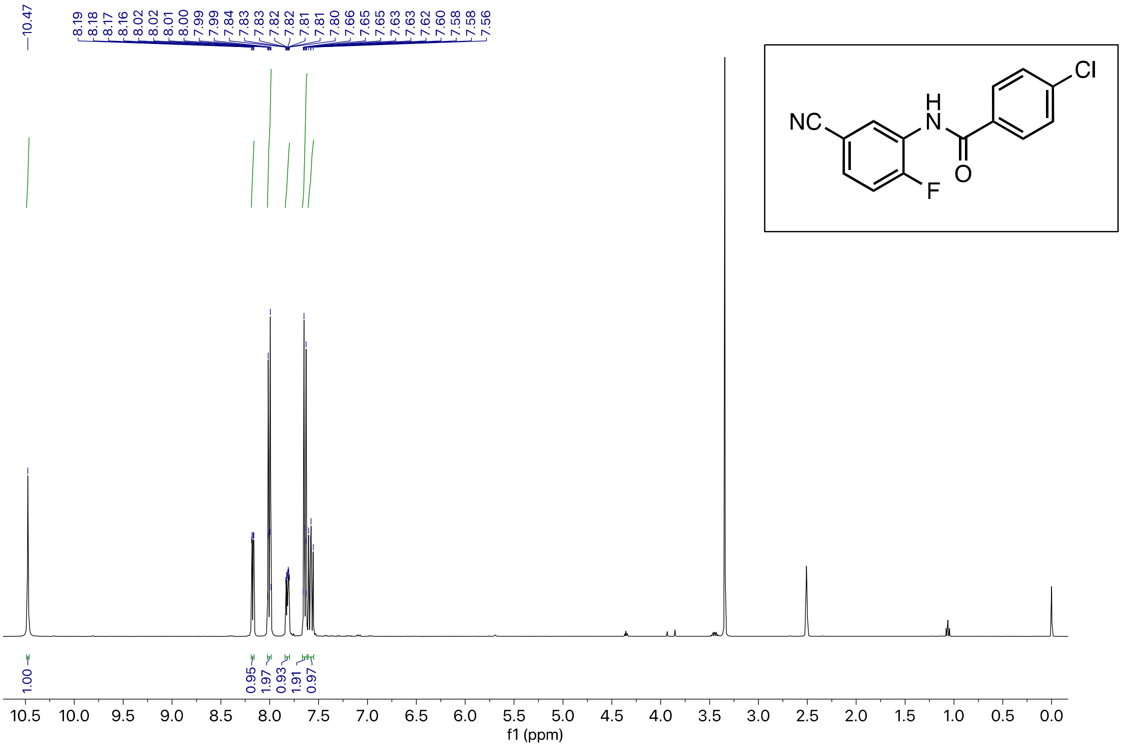

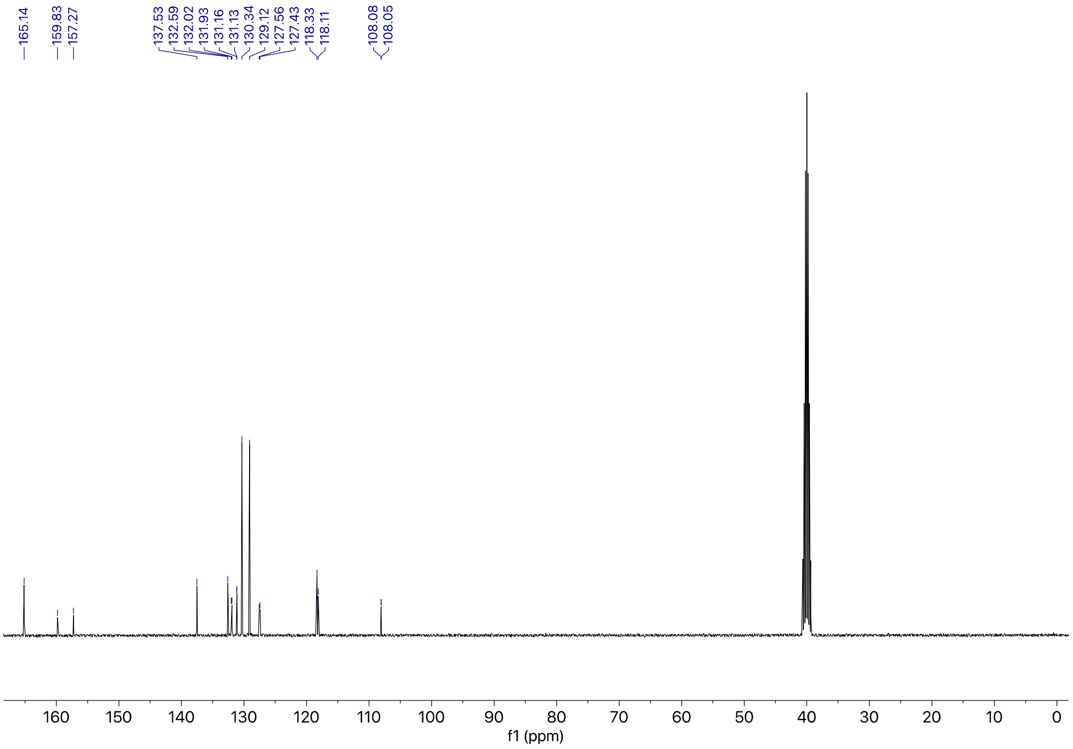

^{1}H and ^{13}C NMR for Methyl 3-benzamido-4-fluorobenzoate (**25**)

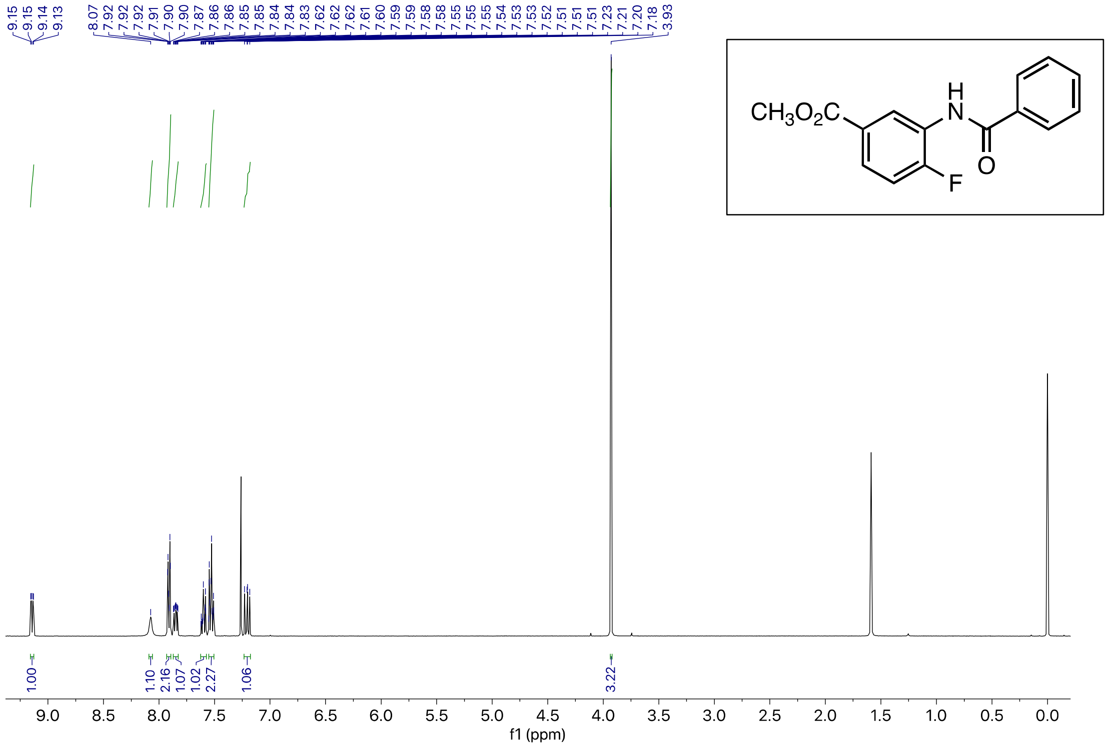

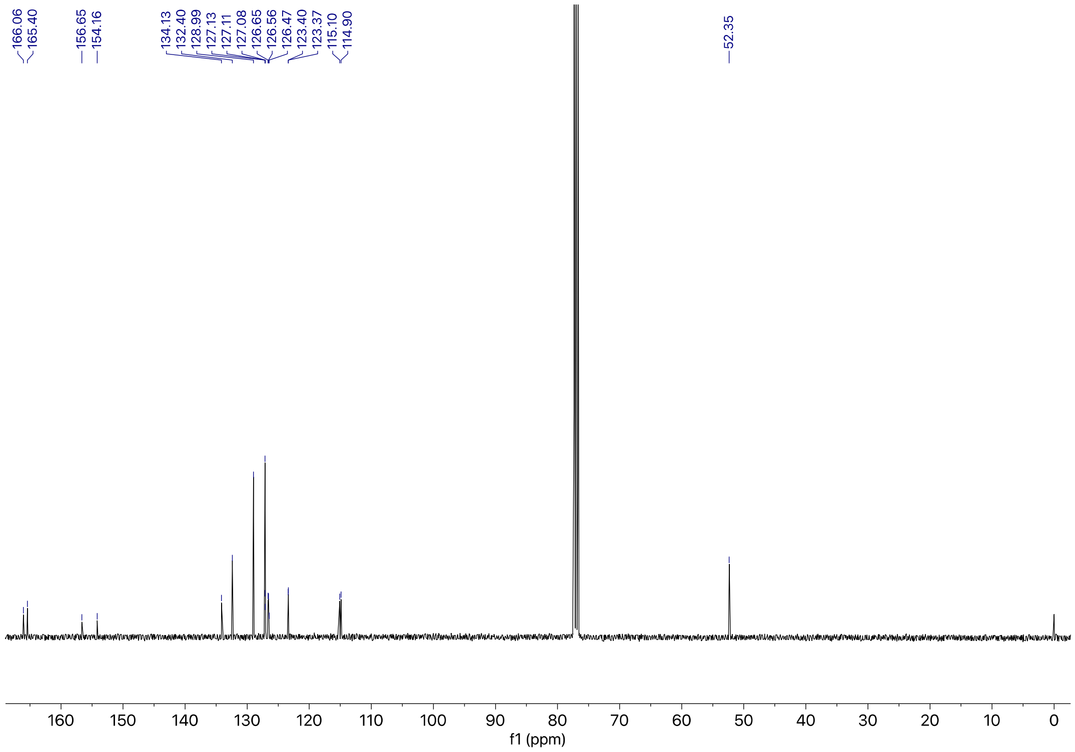

^{1}H and ^{13}C NMR for Methyl 4-fluoro-3-(3-methylbenzamido)benzoate (**26**)

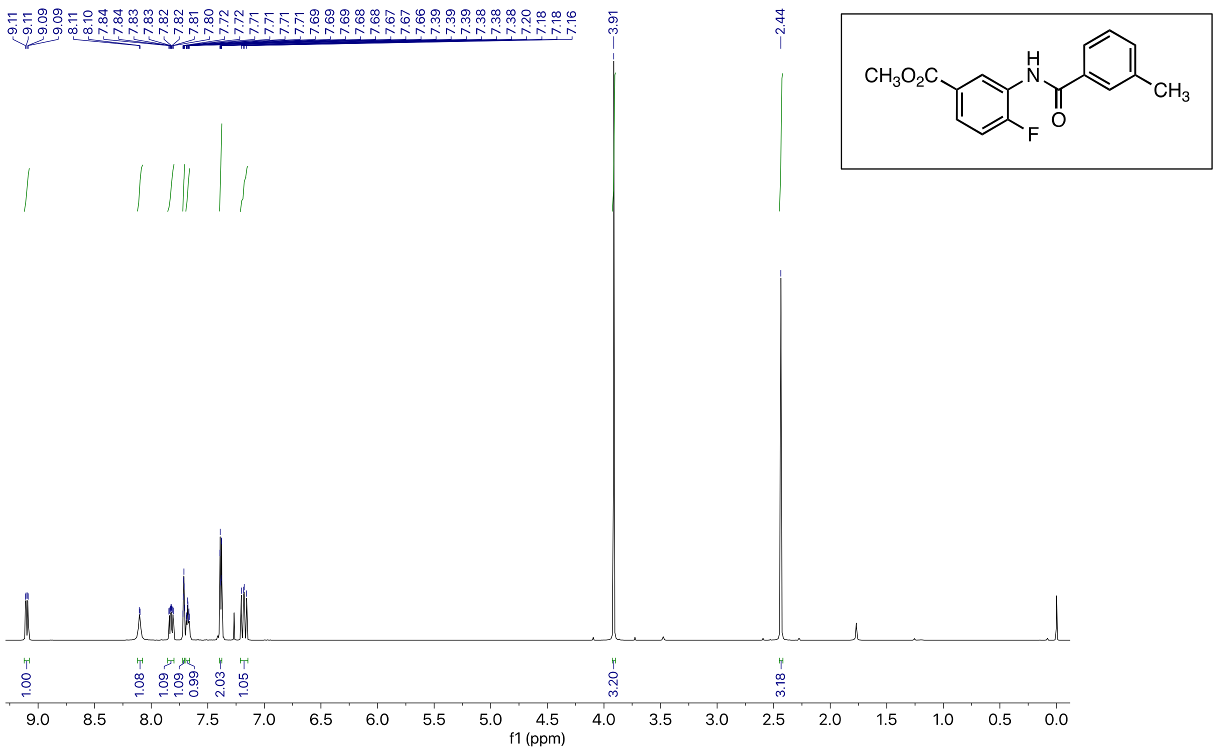

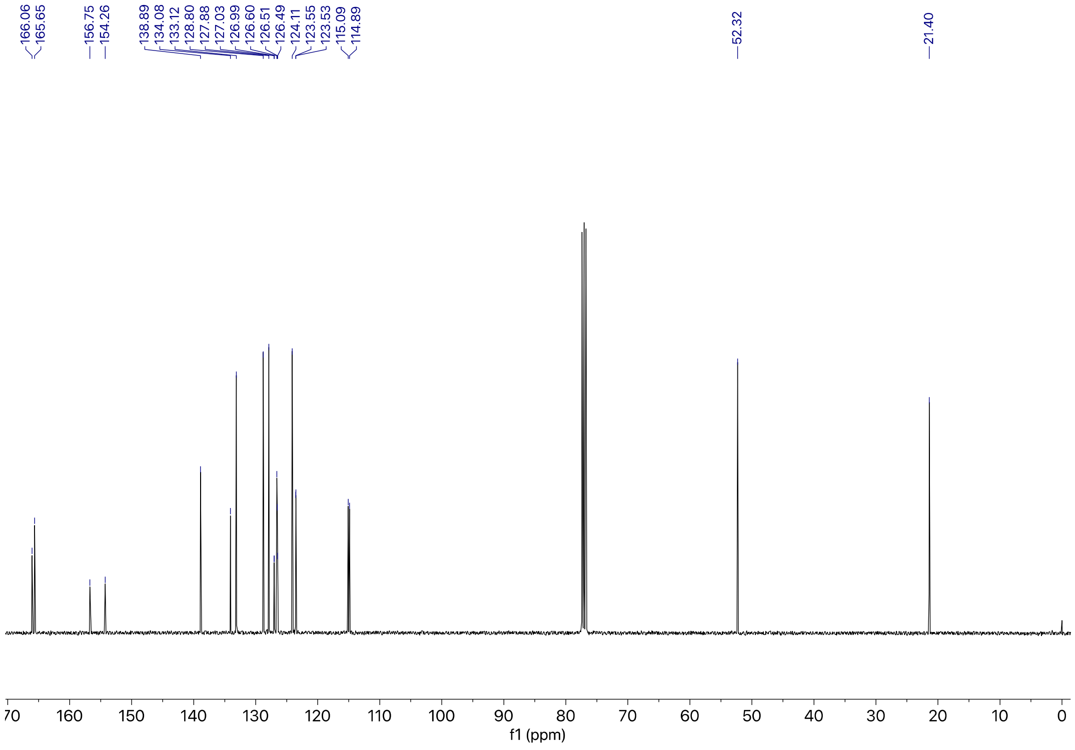

^{1}H and ^{13}C NMR for Methyl 4-fluoro-3-(4-methylbenzamido)benzoate (**27**)

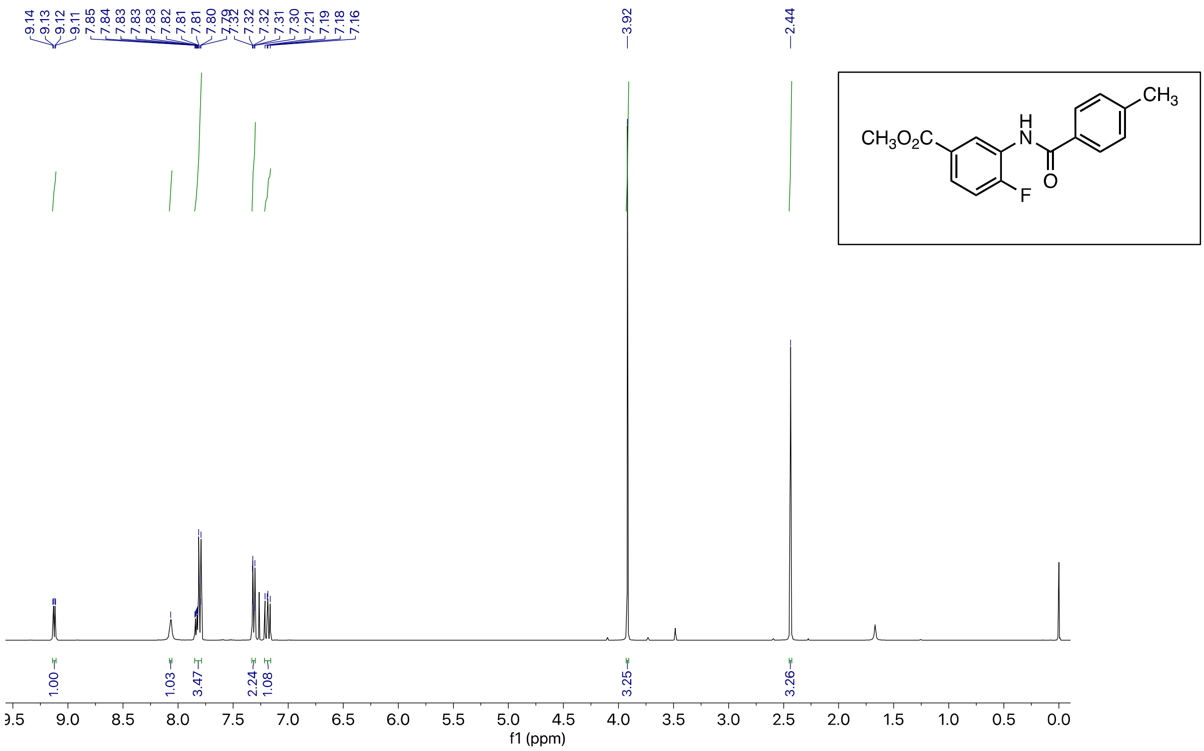

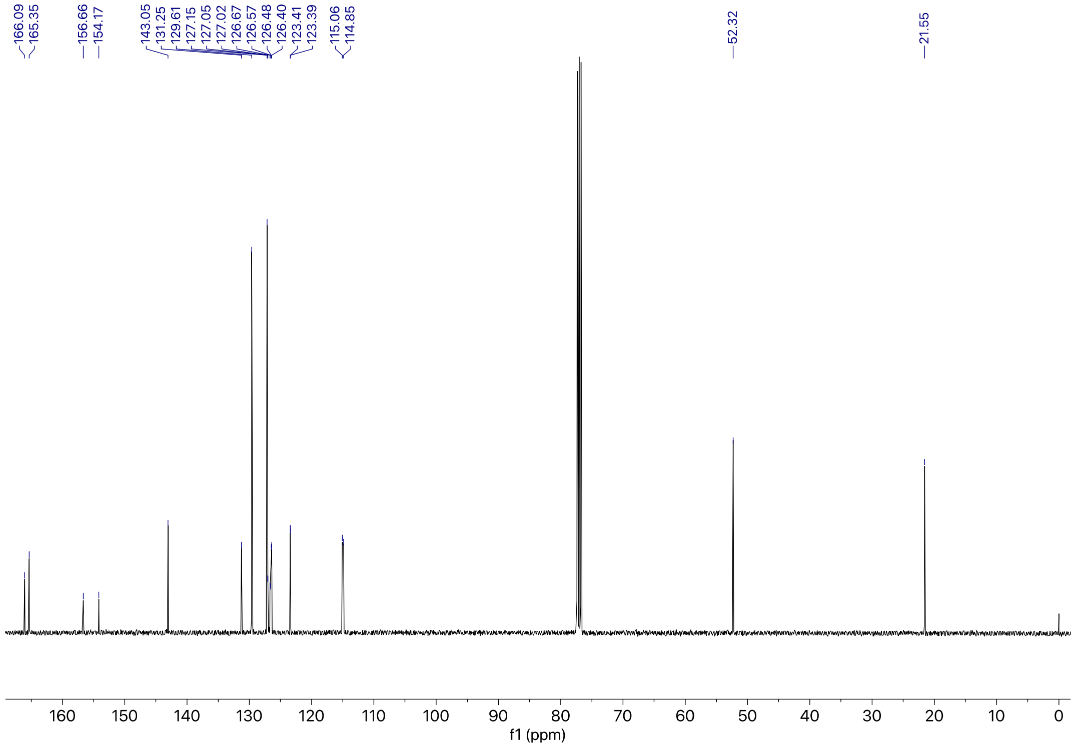

^{1}H and ^{13}C NMR for Methyl 4-fluoro-3-(4-methoxybenzamido)benzoate (**28**)

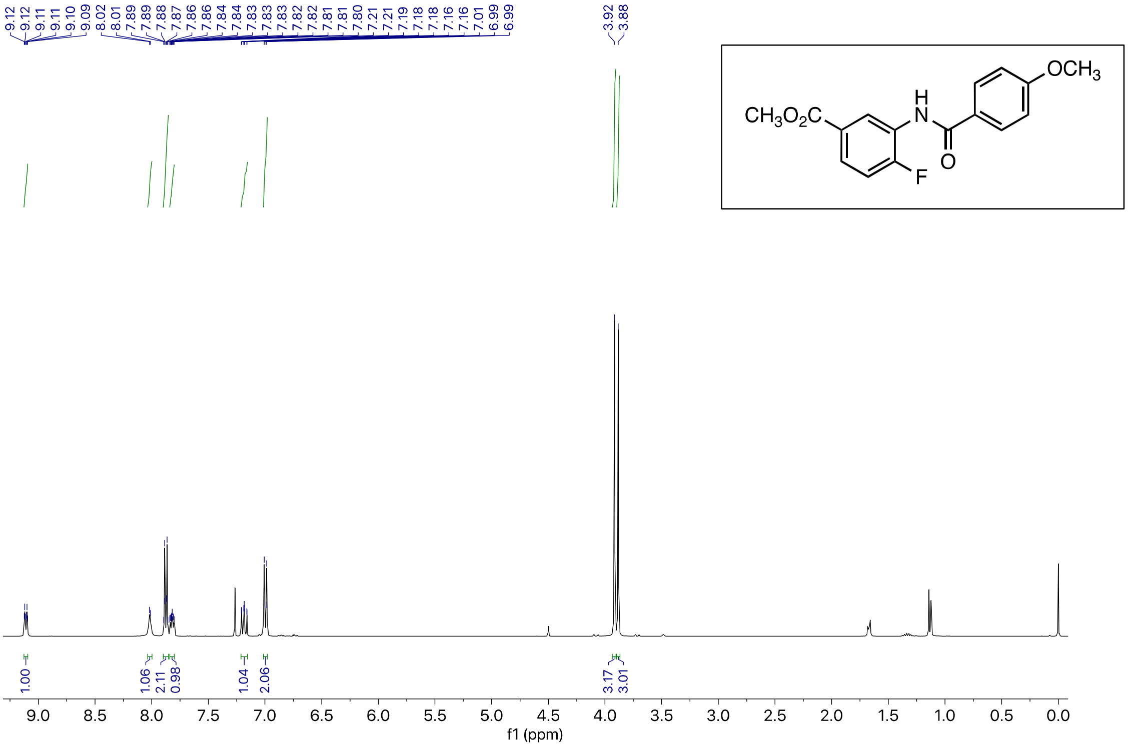

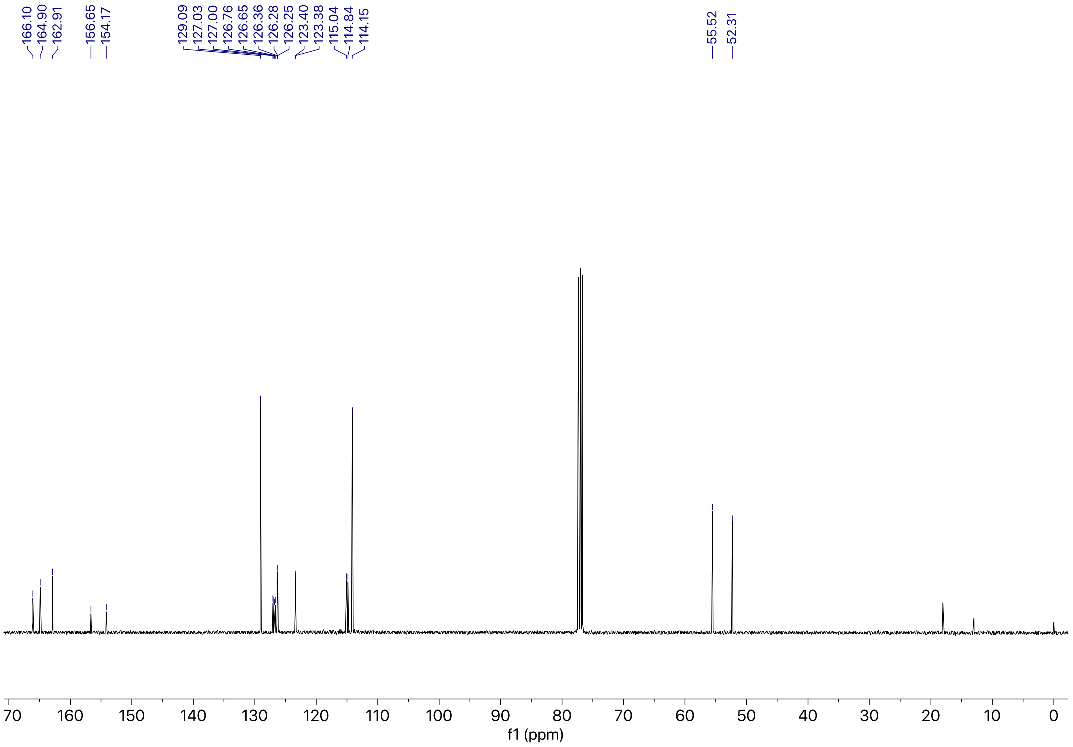

^{1}H and ^{13}C NMR for Methyl 4-fluoro-3-(2-fluorobenzamido)benzoate (**29**)

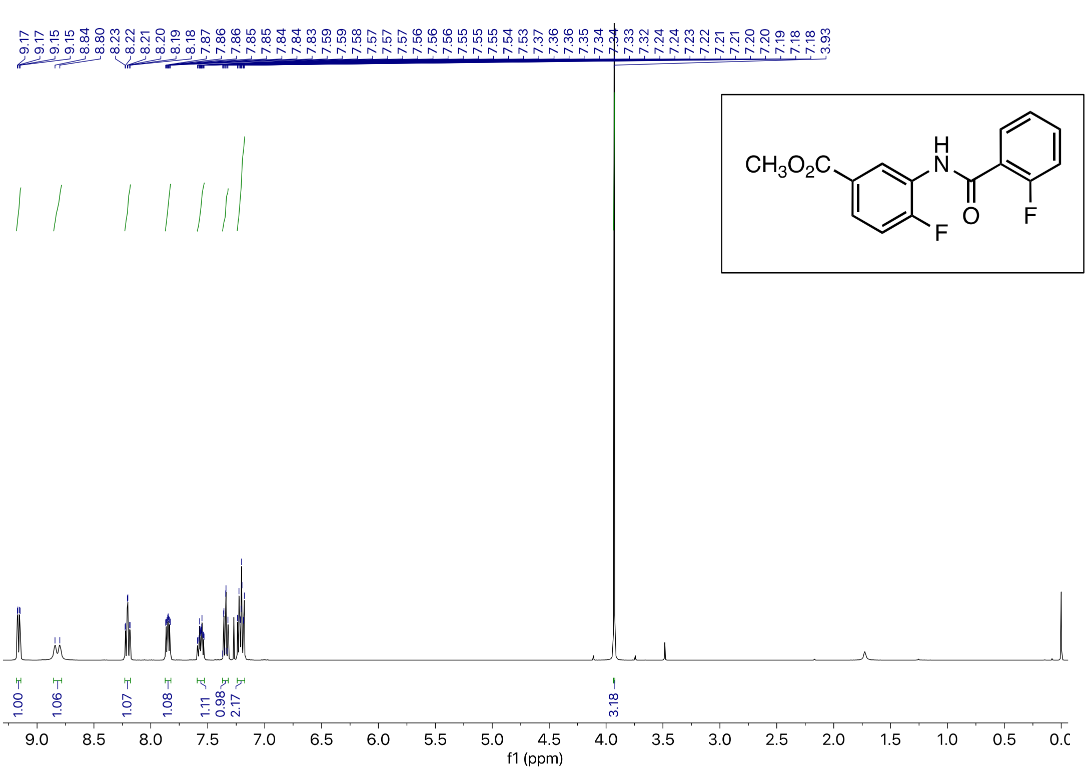

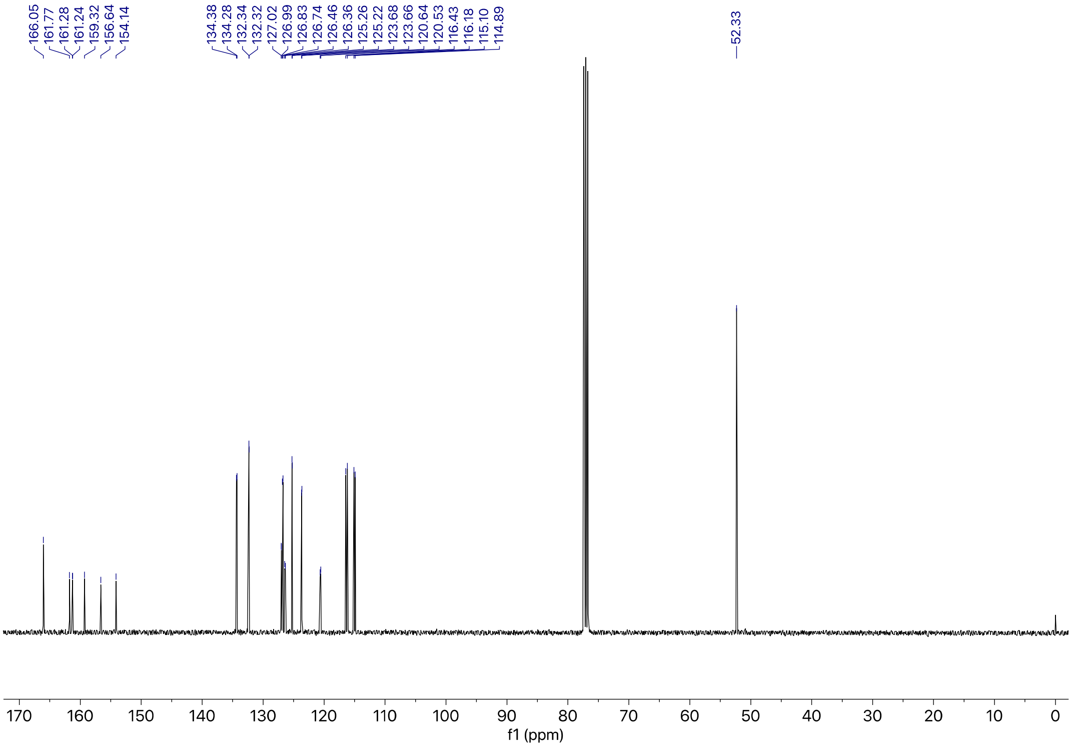

^{19}F NMR for Methyl 4-fluoro-3-(2-fluorobenzamido)benzoate (**29**)

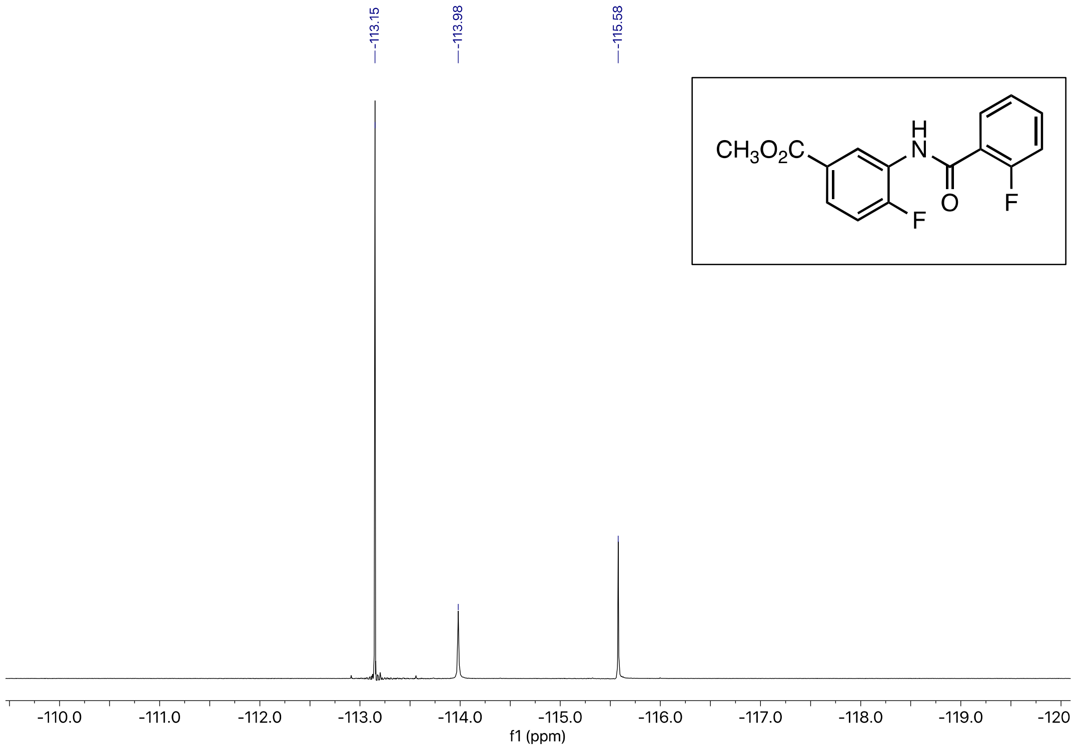

^{1}H and ^{13}C NMR for Methyl 3-(3-chlorobenzamido)-4-fluorobenzoate (**30**)

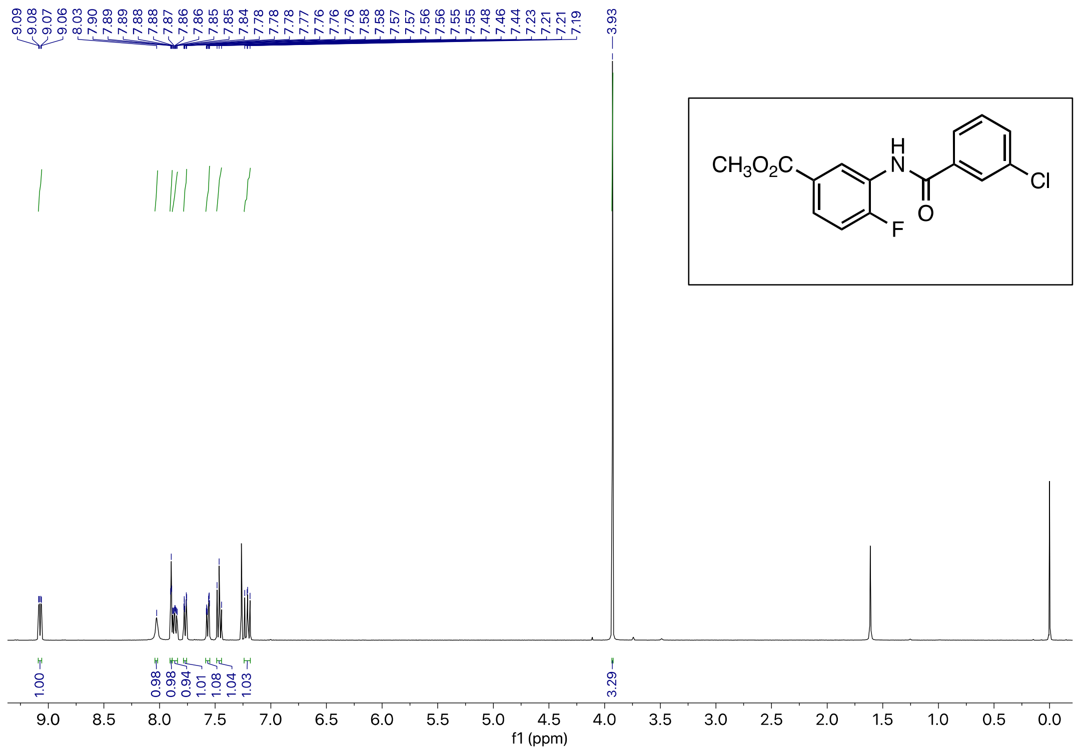

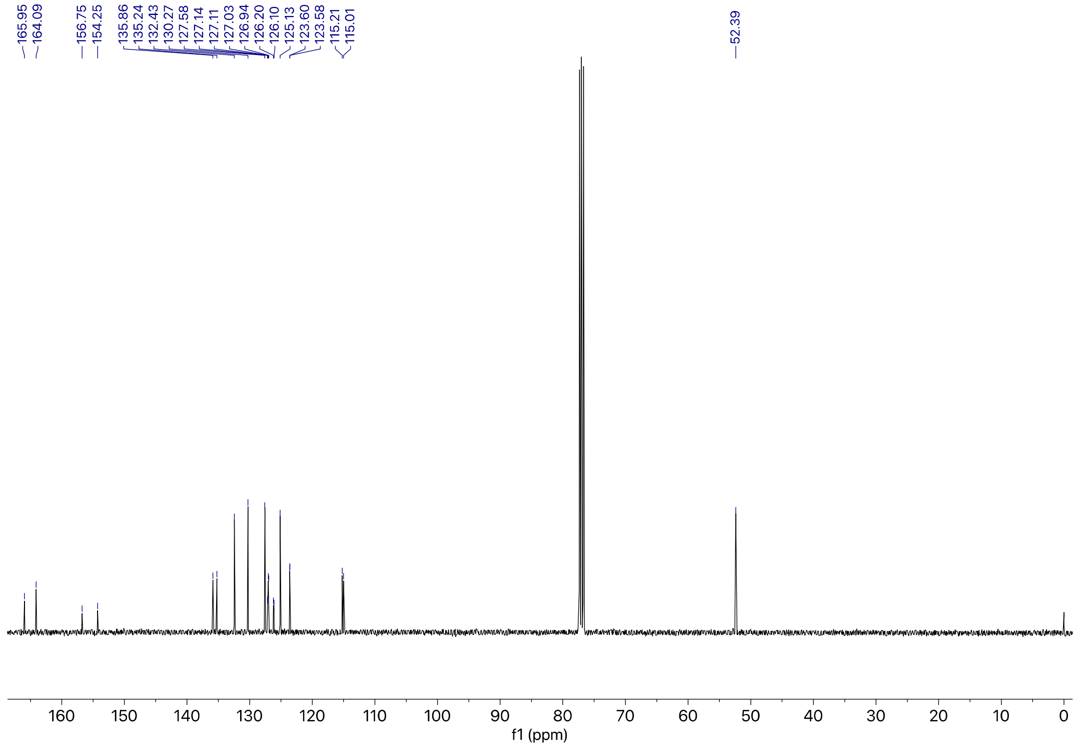

^{1}H and ^{13}C NMR for Methyl 3-(4-chlorobenzamido)-4-chlorobenzoate (**31**)

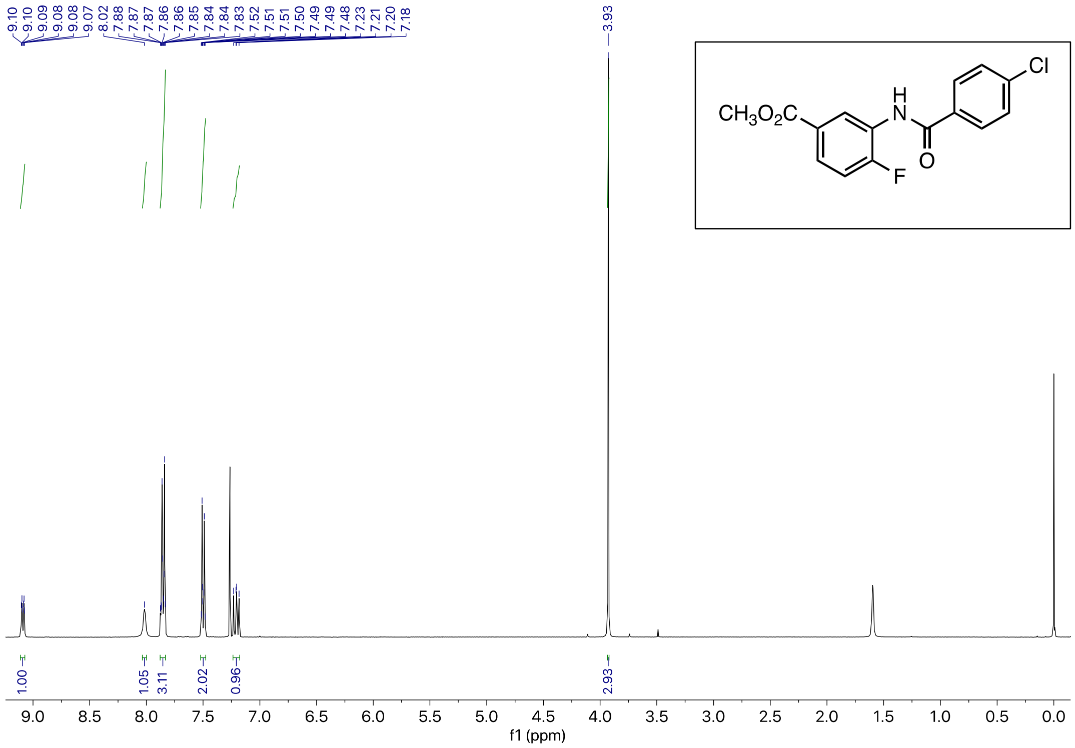

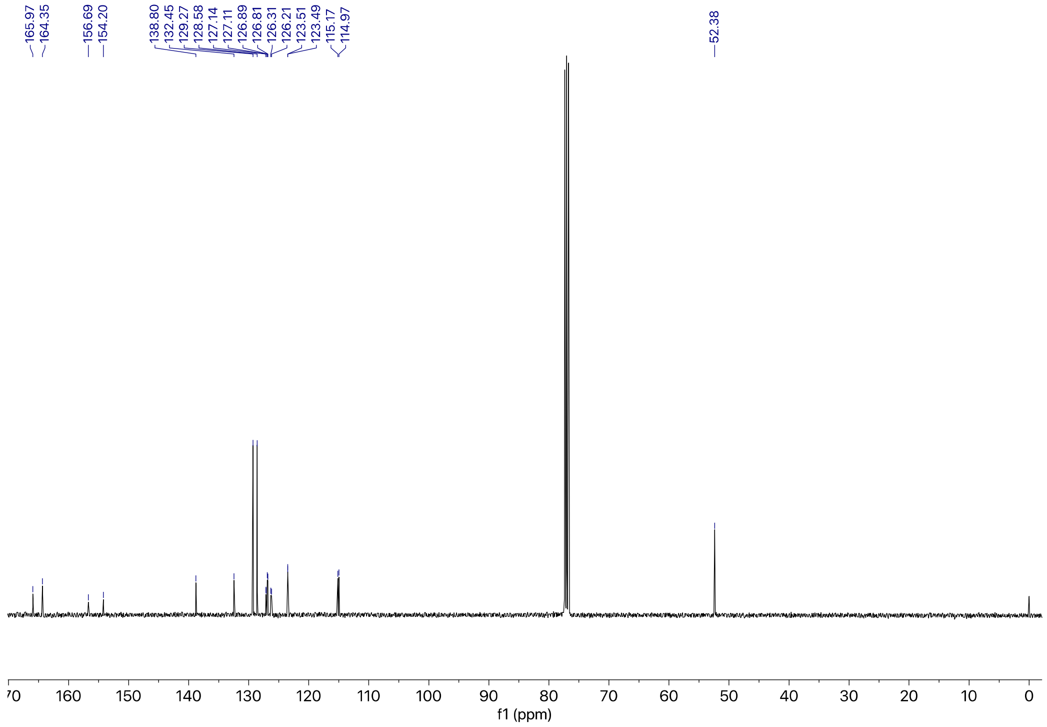

^{1}H and ^{13}C NMR for *N*-(2-Fluoro-5-trifluoromethyl)phenyl)benzamide (**32**)

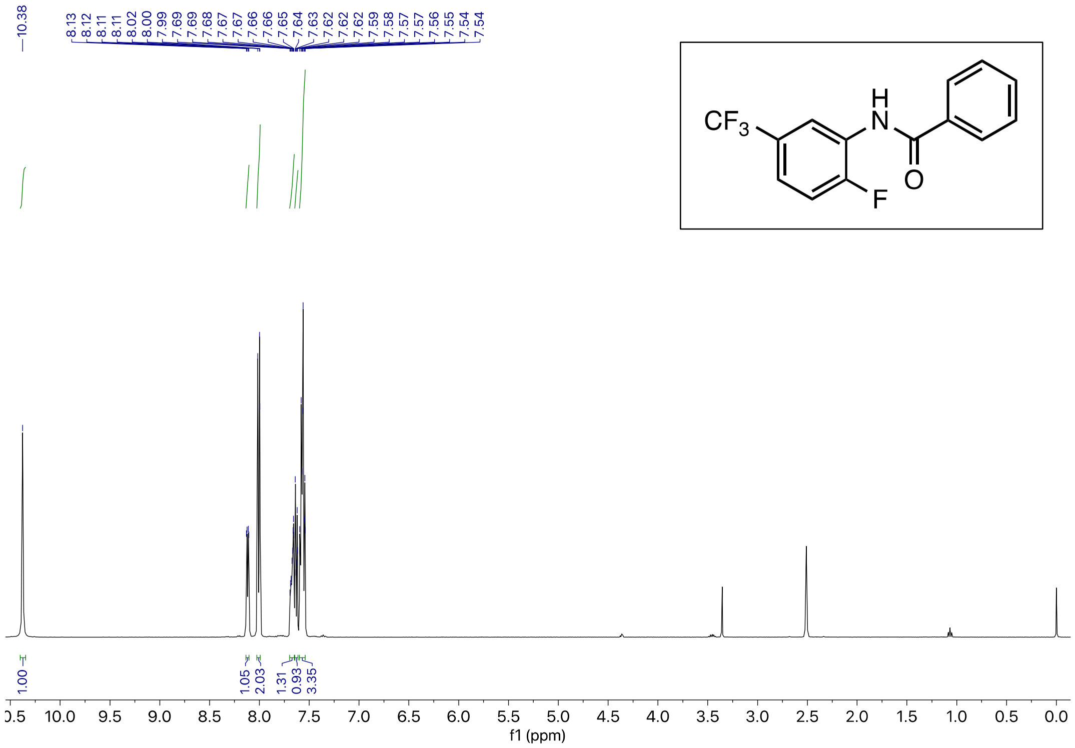

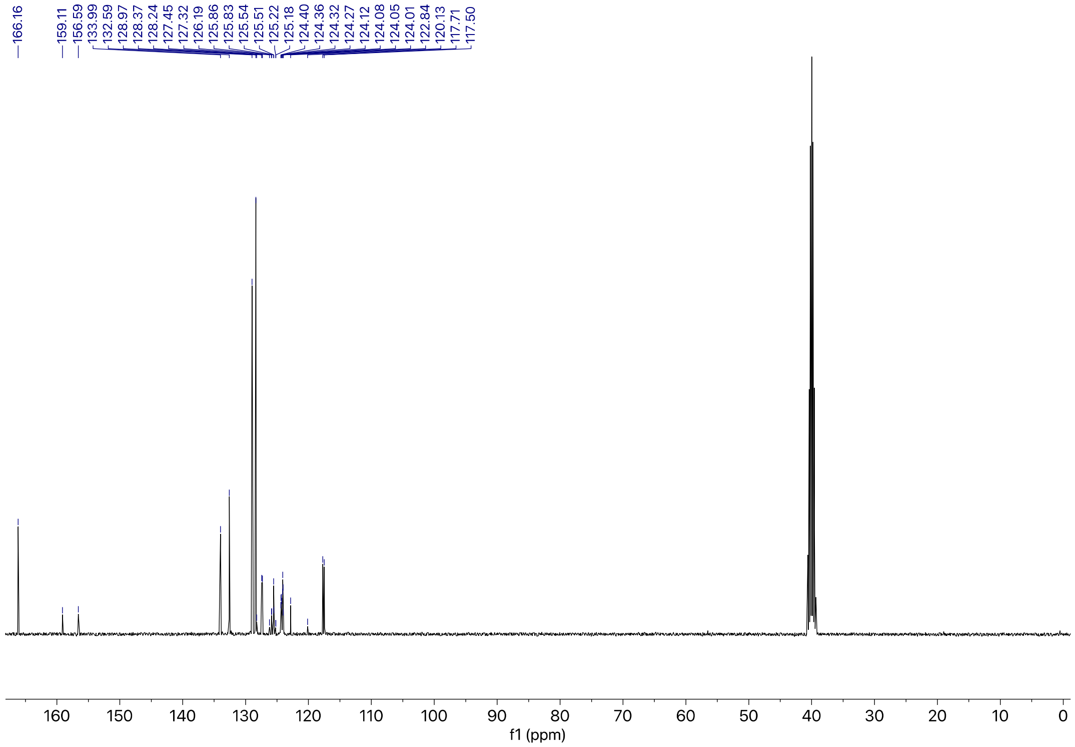

¹⁹F NMR for *N*-(2-Fluoro-5-trifluoromethyl)phenyl)benzamide (**32**)

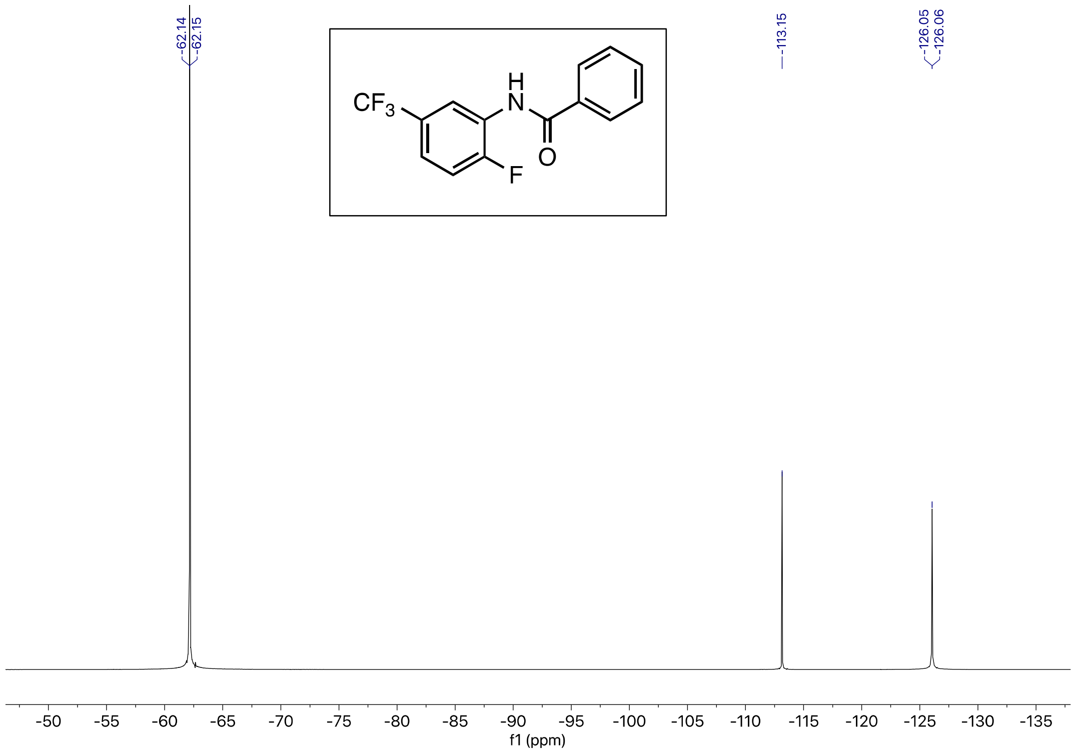

^{1}H and ^{13}C NMR for *N*-(2-Fluoro-5-(trifluoromethyl)phenyl)-3-methylbenzamide (**33**)

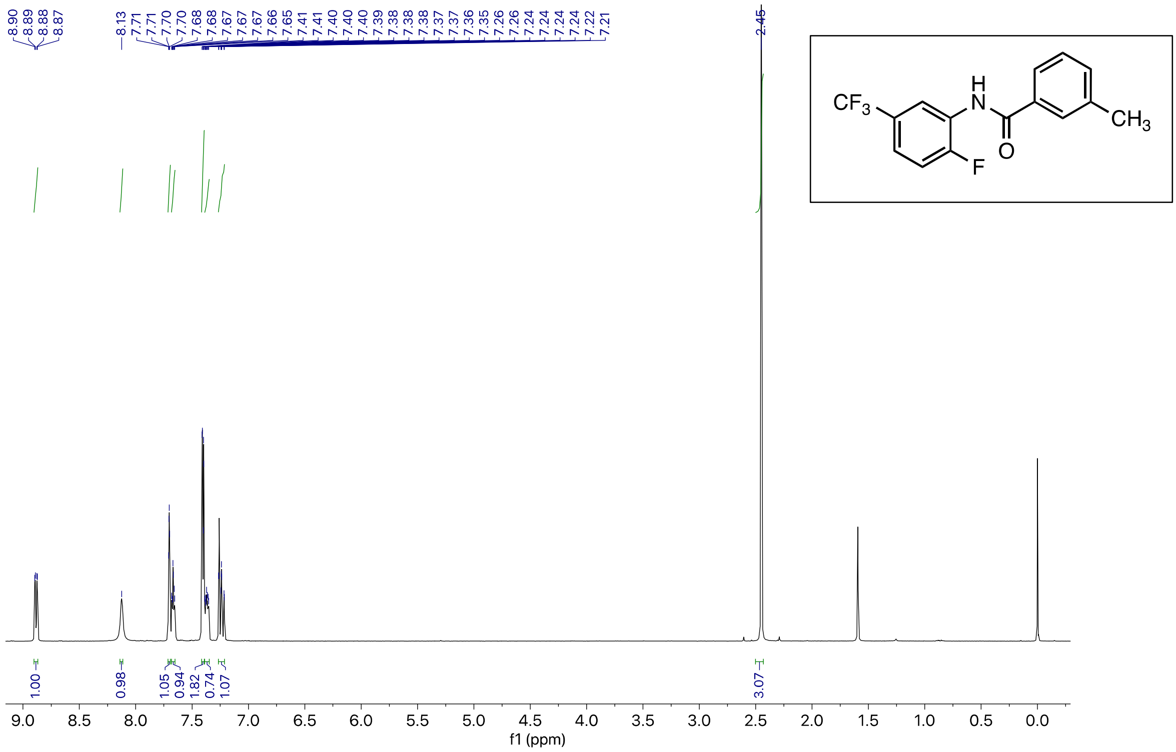

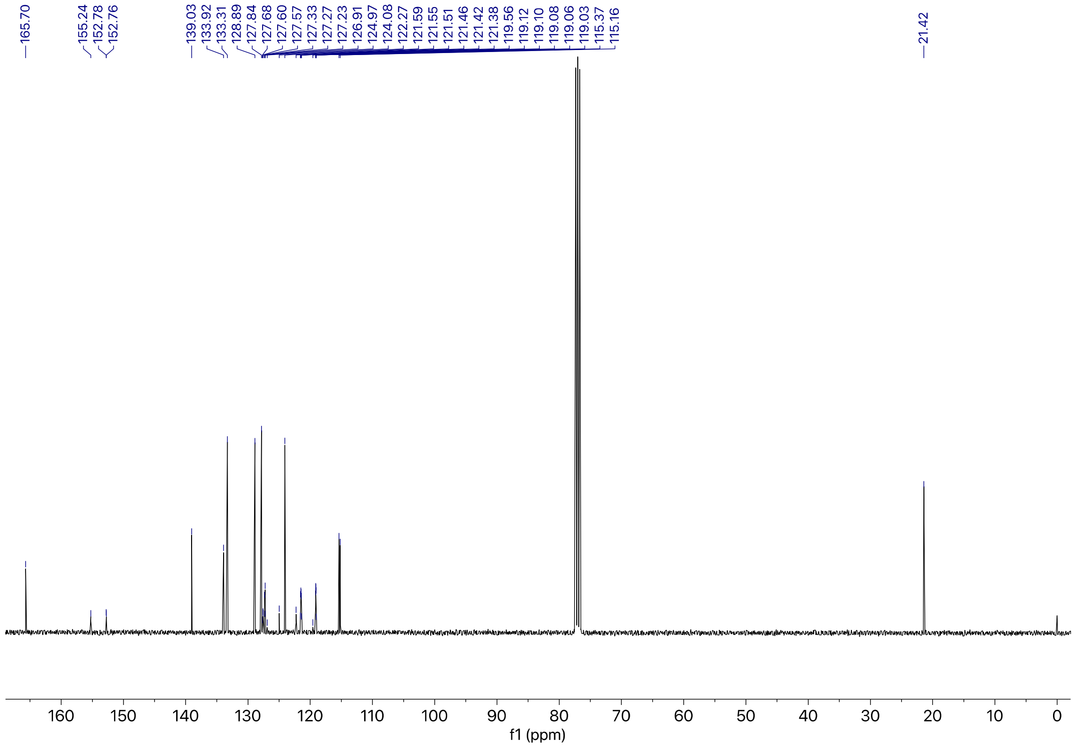

^{19}F NMR for *N*-(2-Fluoro-5-(trifluoromethyl)phenyl)-3-methylbenzamide (**33**)

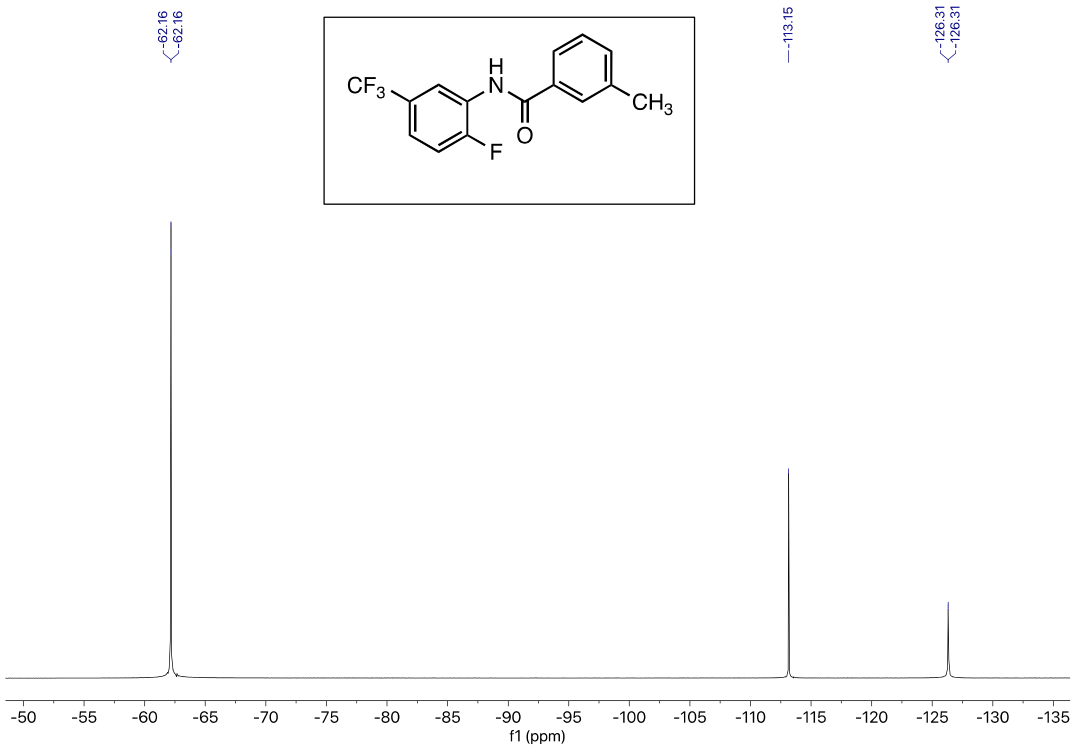

^{1}H and ^{13}C NMR for *N*-(2-Fluoro-5-(trifluoromethyl)phenyl)-4-methylbenzamide (**34**)

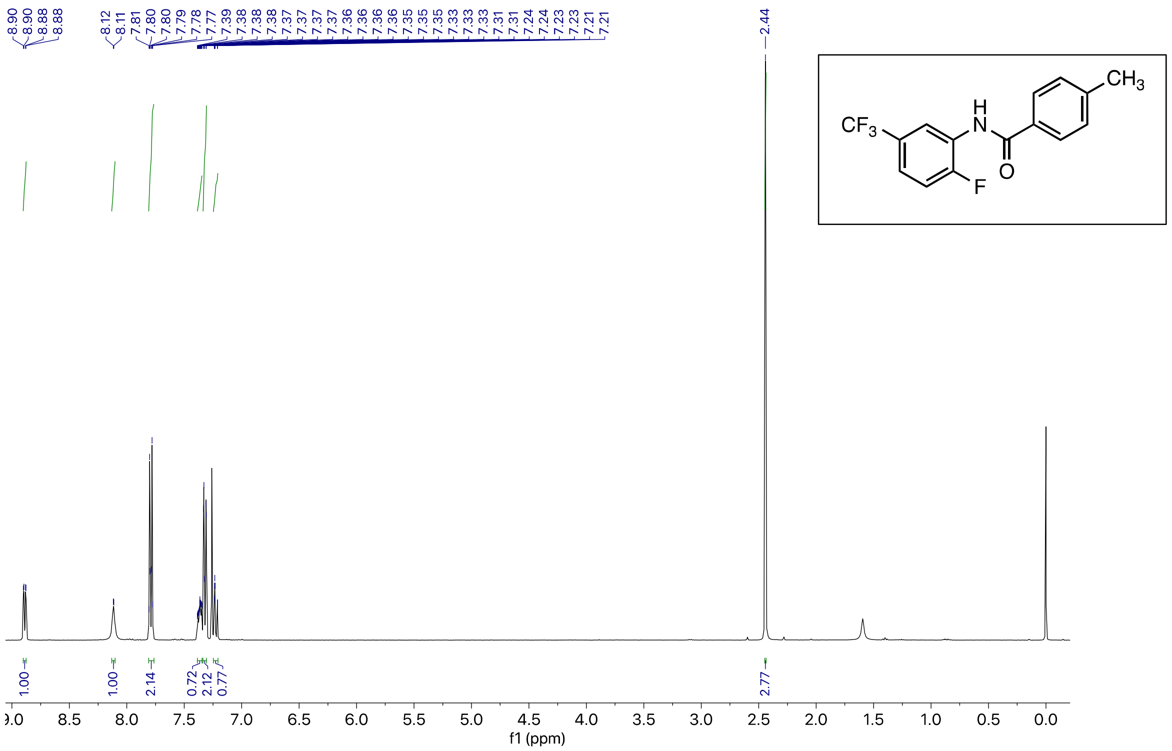

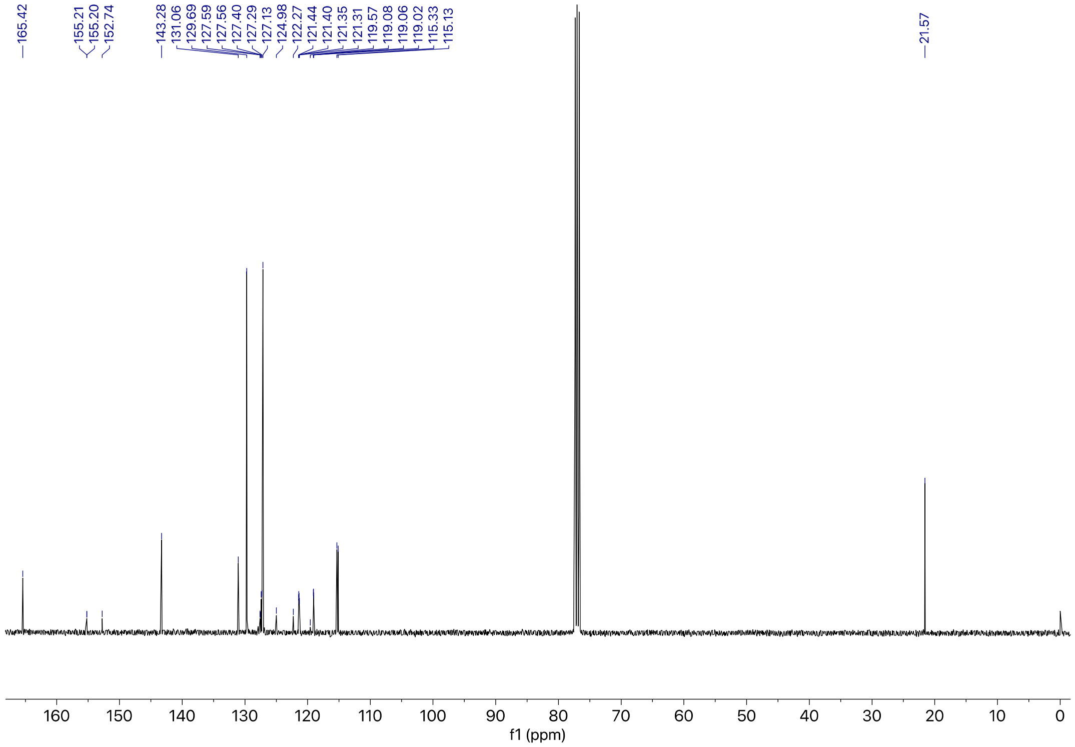

^{19}F NMR for *N*-(2-Fluoro-5-(trifluoromethyl)phenyl)-4-methylbenzamide (**34**)

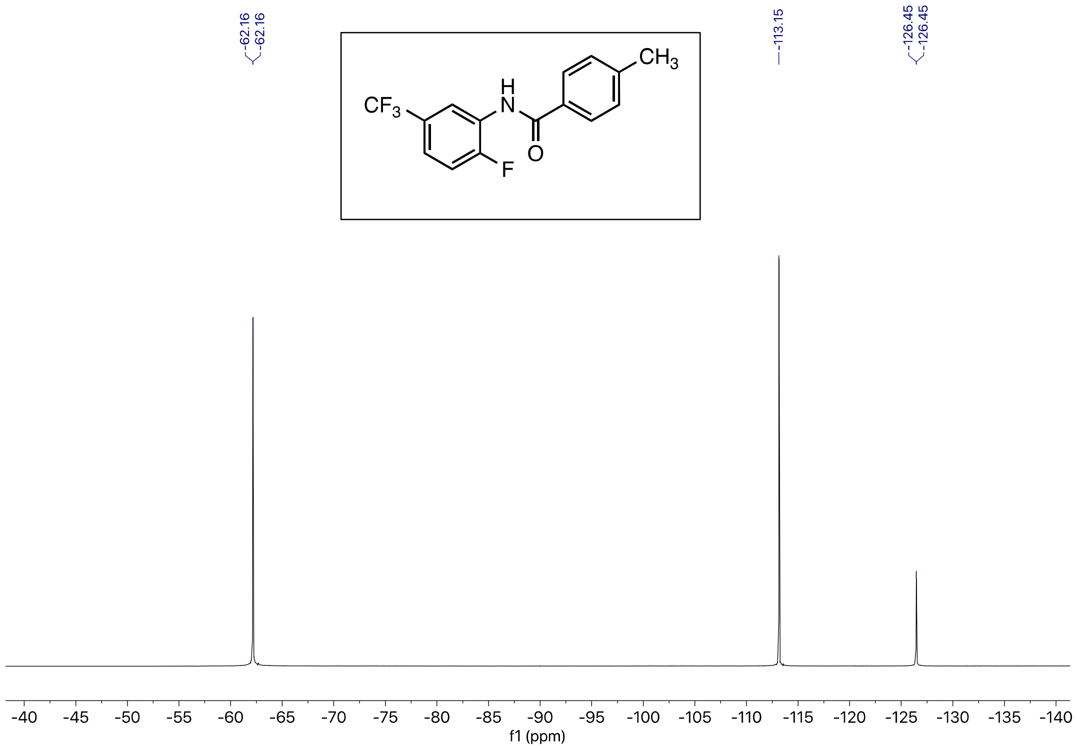

^{1}H and ^{13}C NMR for *N*-(2-Fluoro-5-(trifluoromethyl)phenyl)-4-methoxybenzamide (**35**)

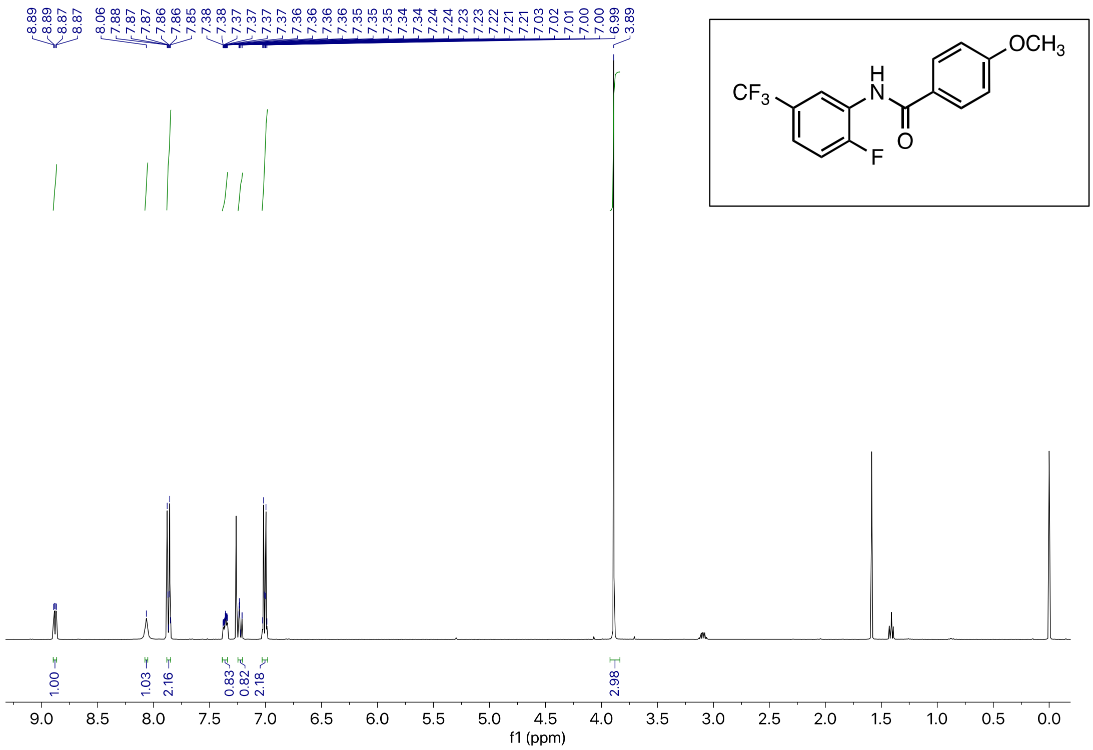

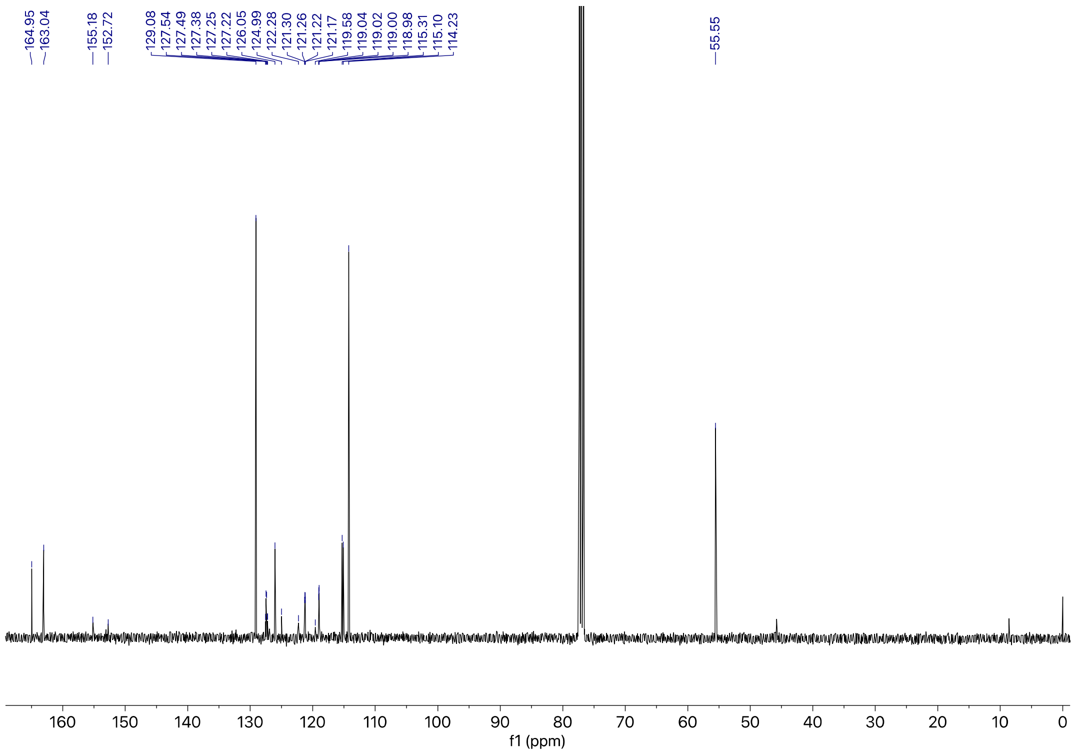

^{13}C NMR for *N*-(2-Fluoro-5-(trifluoromethyl)phenyl)-4-methoxybenzamide (**35**)

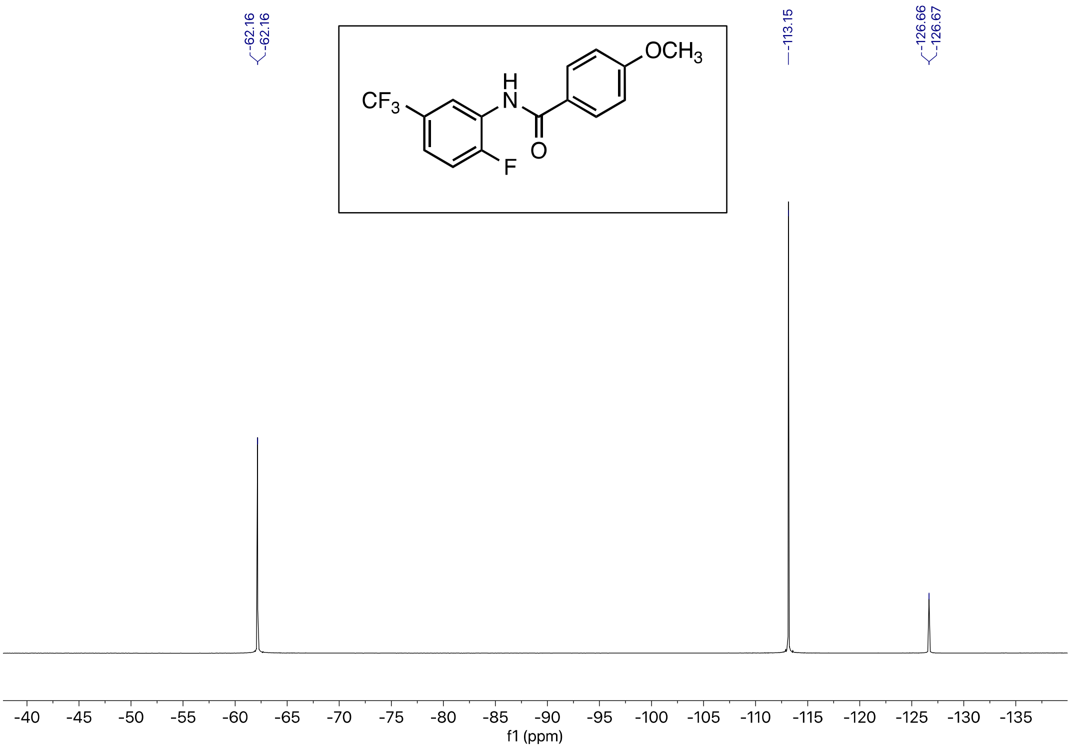

^{1}H and ^{13}C NMR for 2-Fluoro-*N*-(2-fluoro-5-(trifluoromethyl)phenyl)benzamide (**36**)

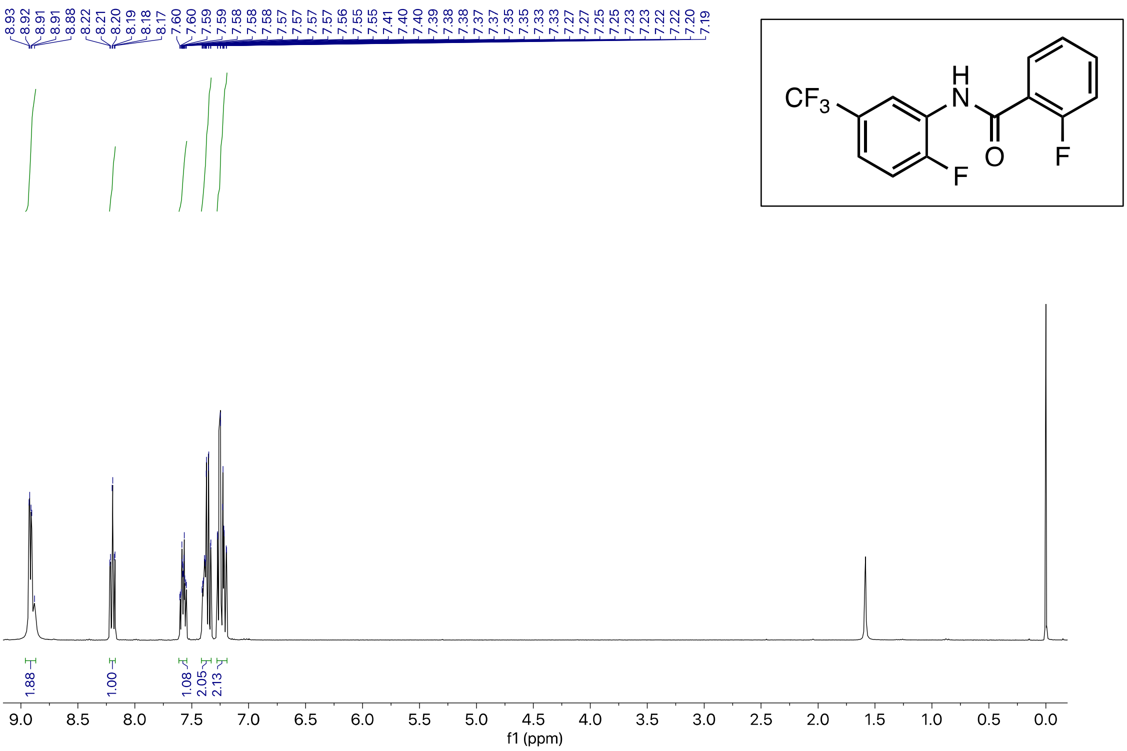

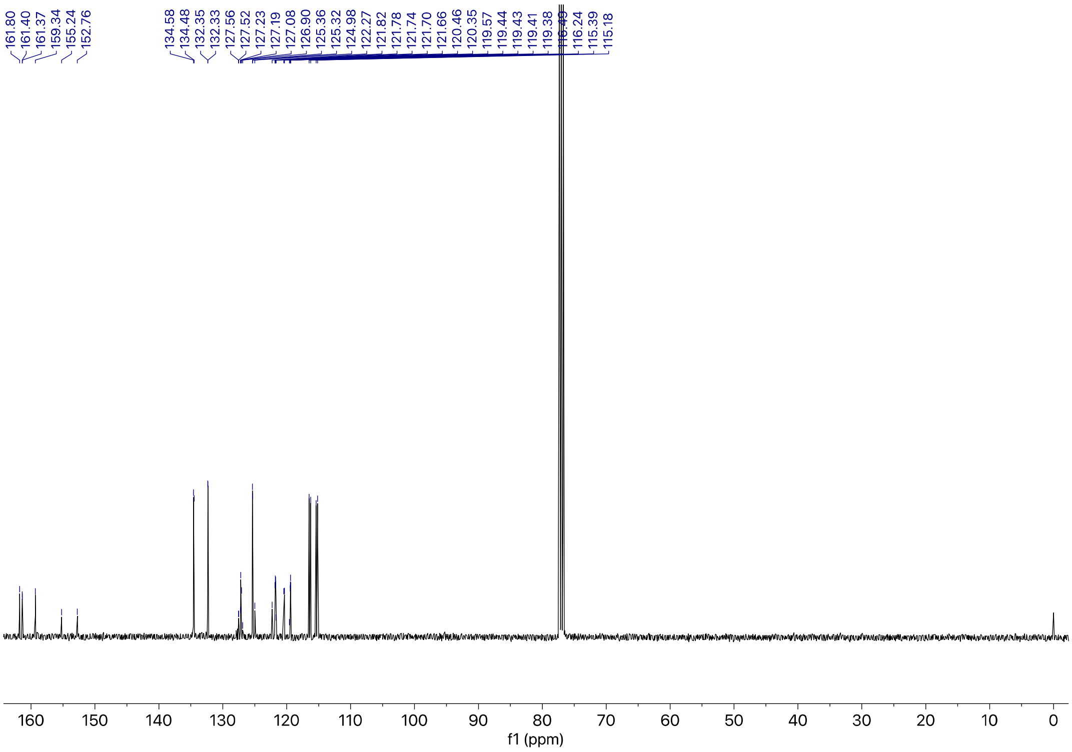

^{19}F NMR for 2-Fluoro-*N*-(2-fluoro-5-(trifluoromethyl)phenyl)benzamide (**36**)

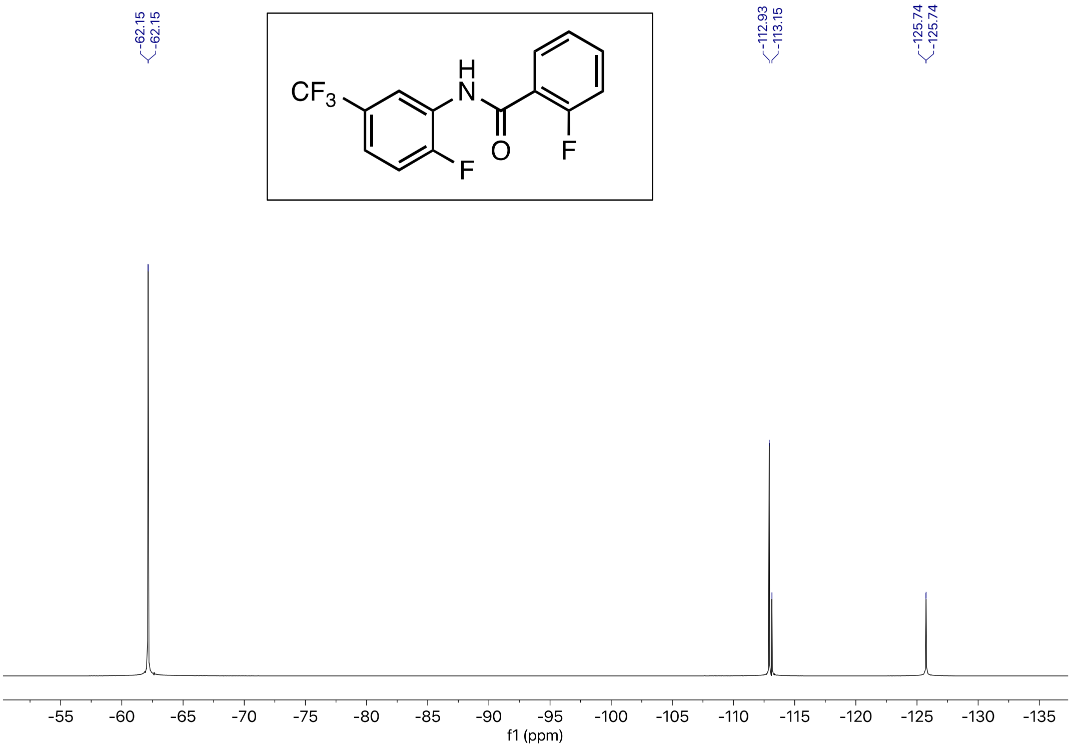

^{1}H and ^{13}C NMR for 3-Chloro-*N*-(2-fluoro-5-(trifluoromethyl)phenyl)benzamide (**37**)

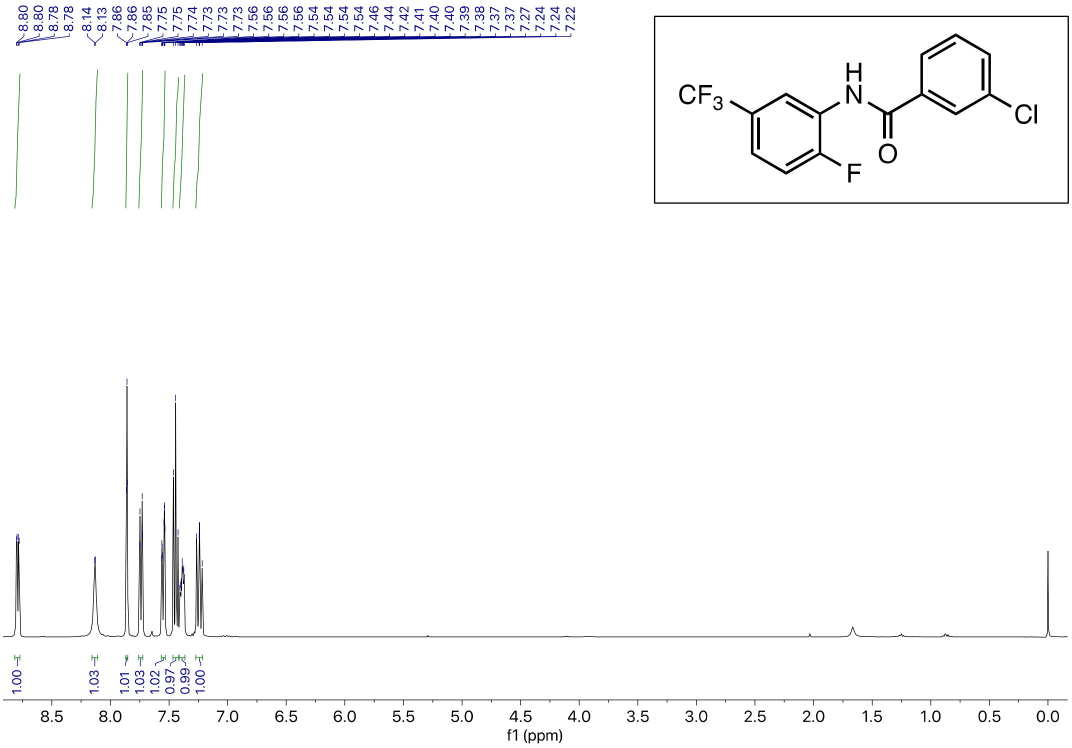

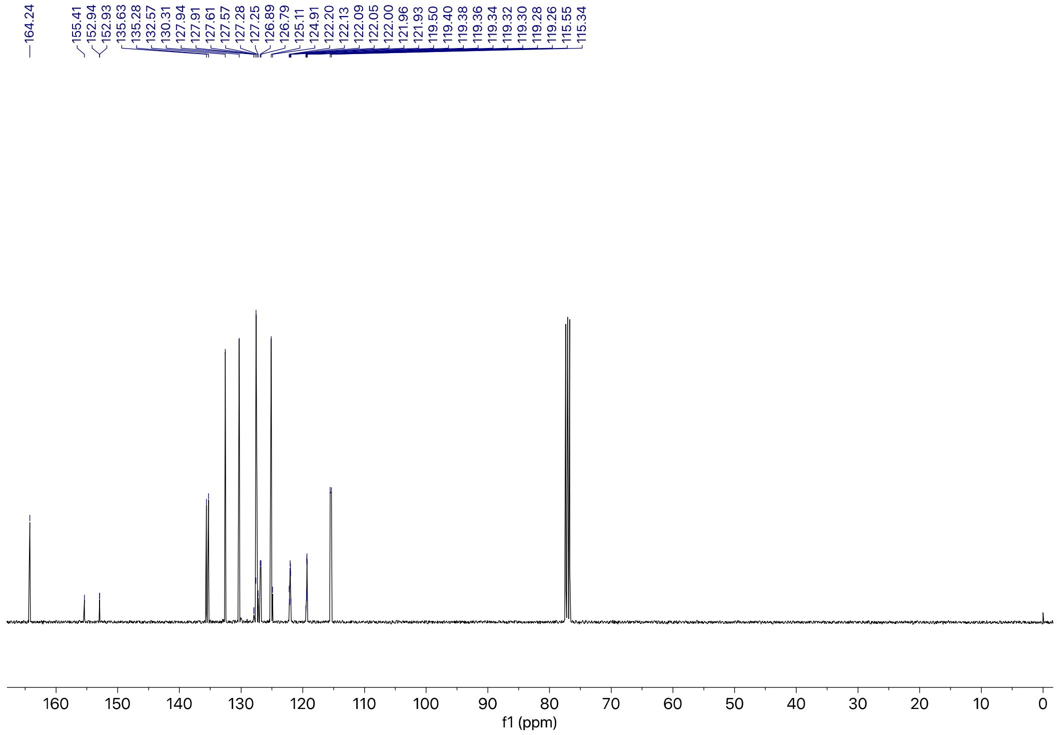

^{19}F NMR for 3-Chloro-*N*-(2-fluoro-5-(trifluoromethyl)phenyl)benzamide (**37**)

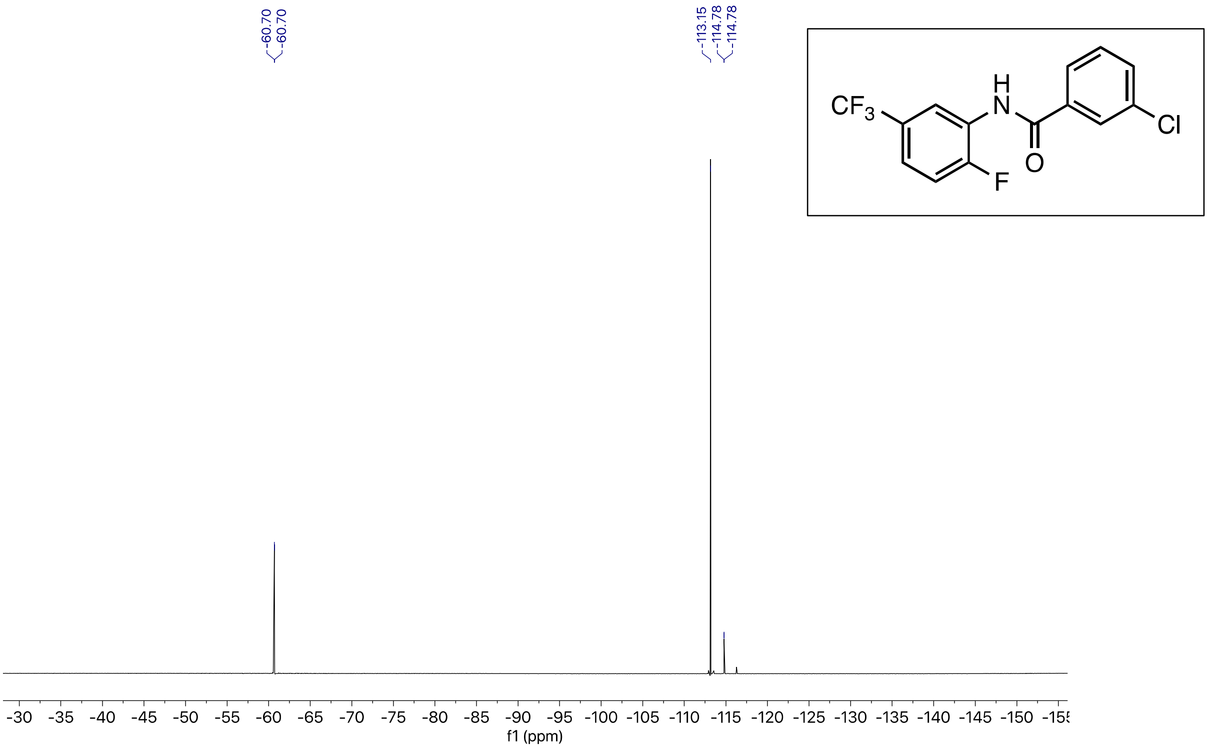

^{1}H and ^{13}C NMR for 4-Chloro-*N*-(2-fluoro-5-(trifluoromethyl)phenyl)benzamide (**38**)

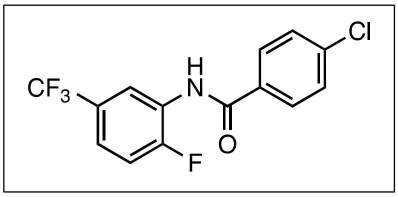

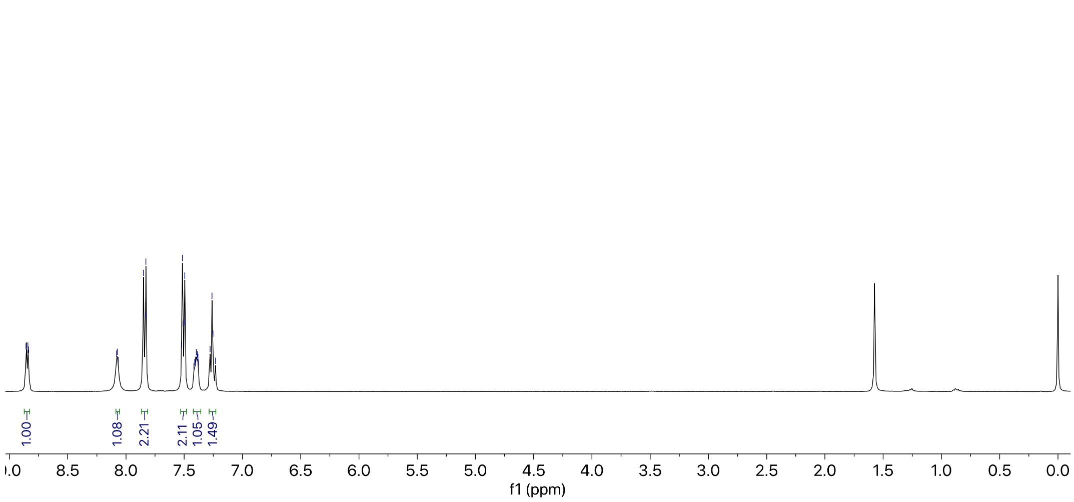

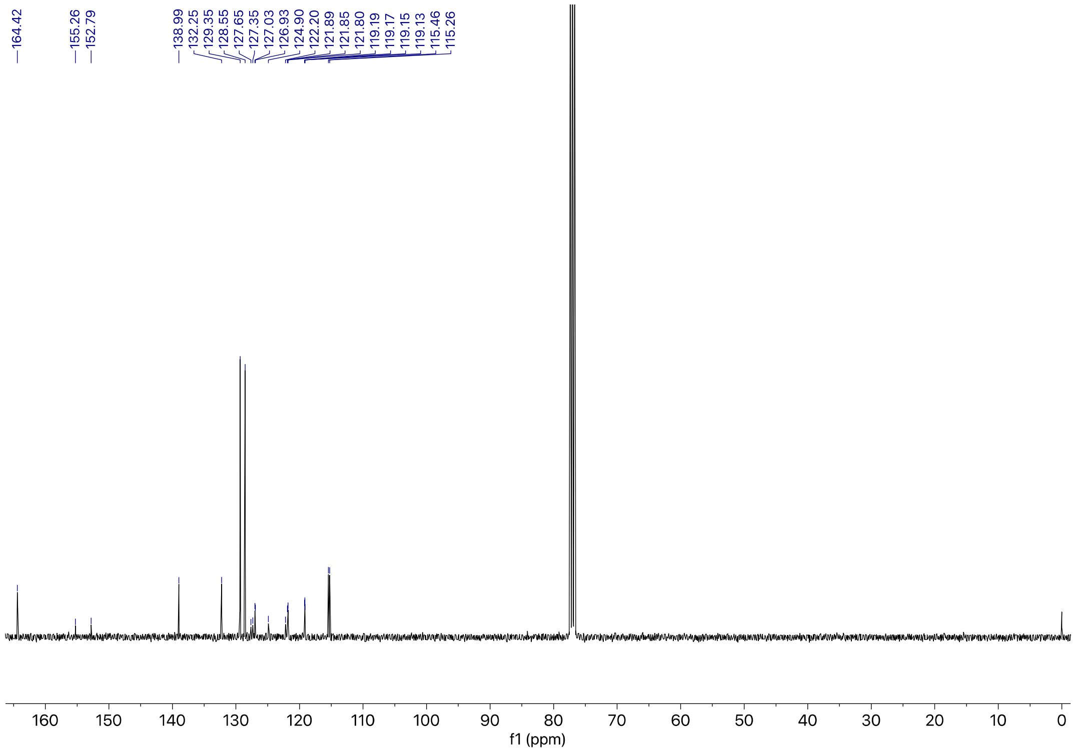

^{19}F NMR for 4-Chloro-*N*-(2-fluoro-5-(trifluoromethyl)phenyl)benzamide (**38**)

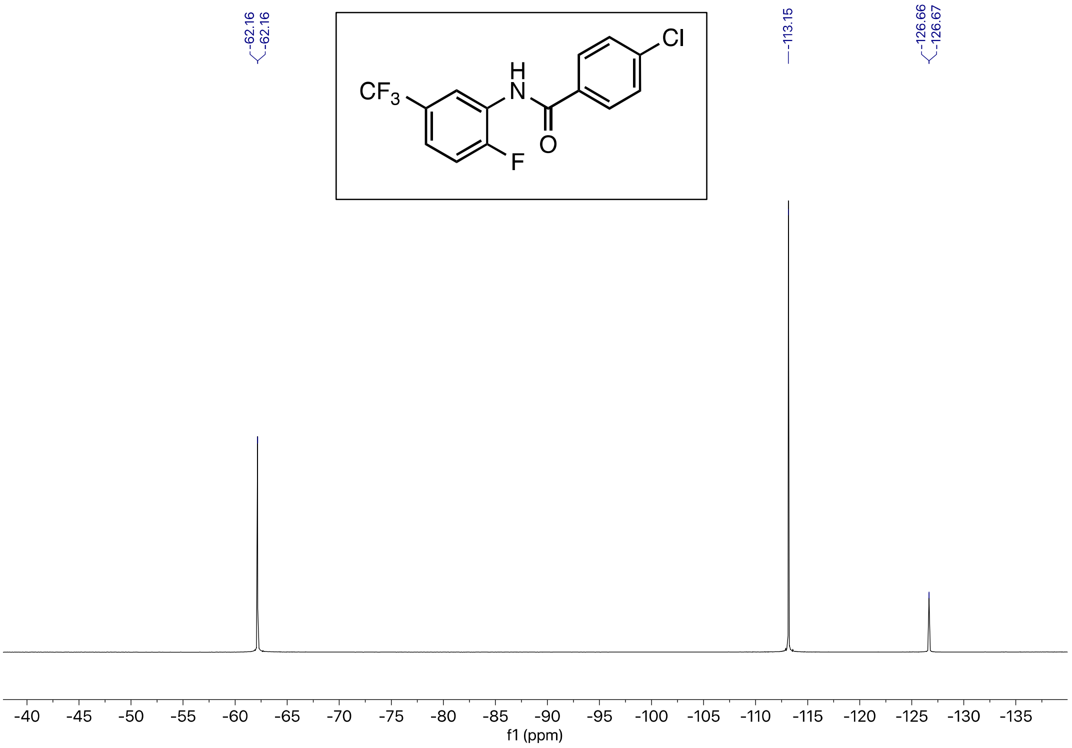

^{1}H and ^{13}C NMR for 2-Methyl-5-nitrobenzo[*d*]oxazole (**39**)

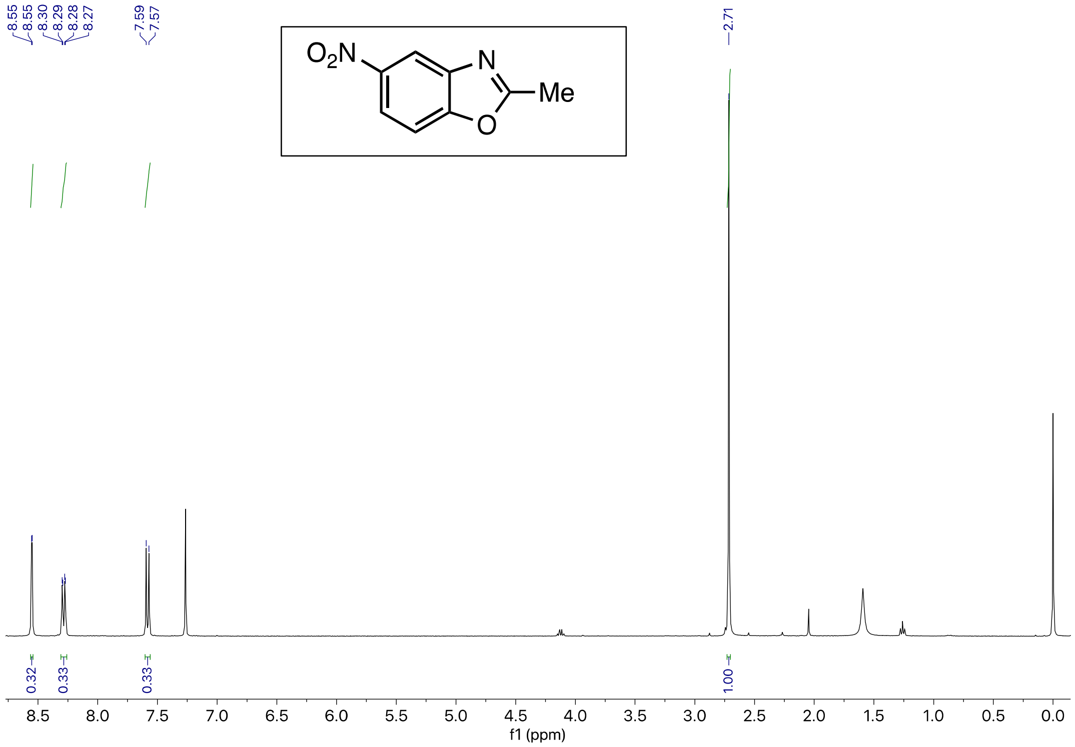

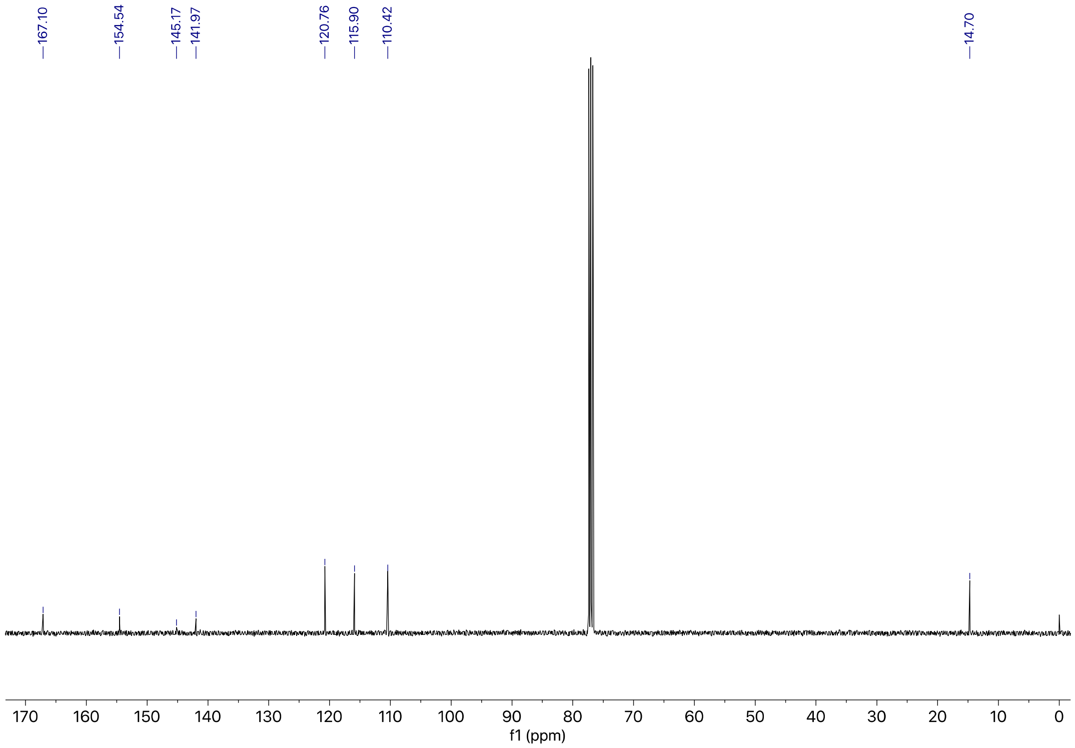

^{1}H and ^{13}C NMR for 5-Nitro-2-pentylbenzo[*d*]oxazole (**40**)

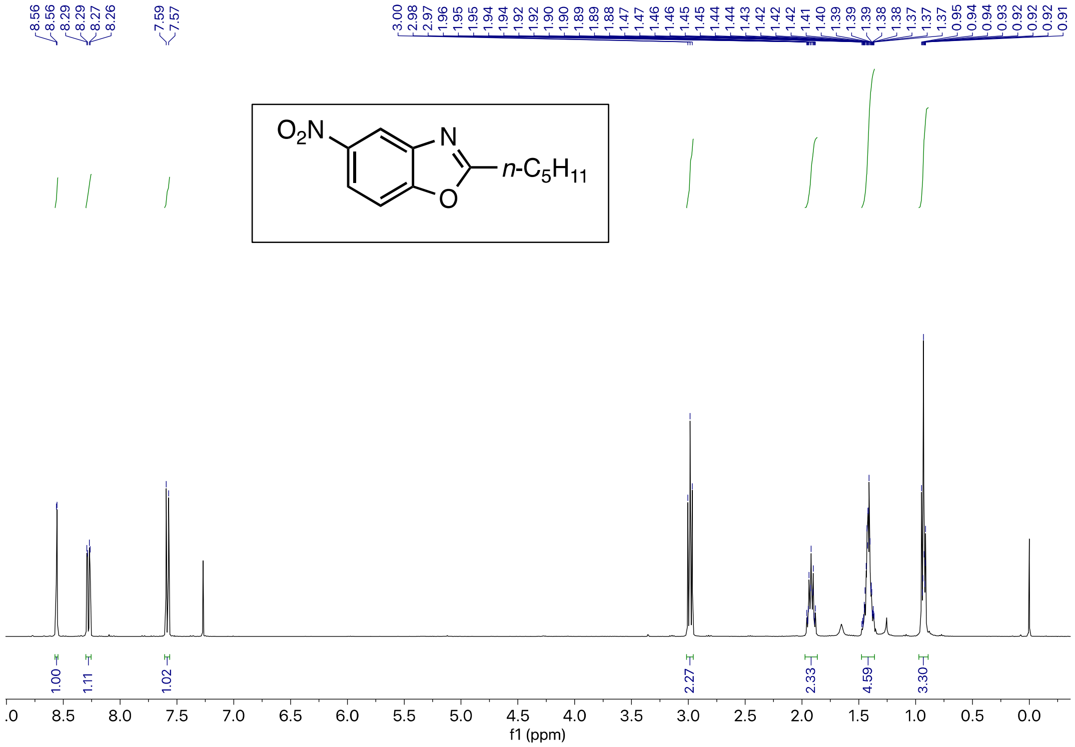

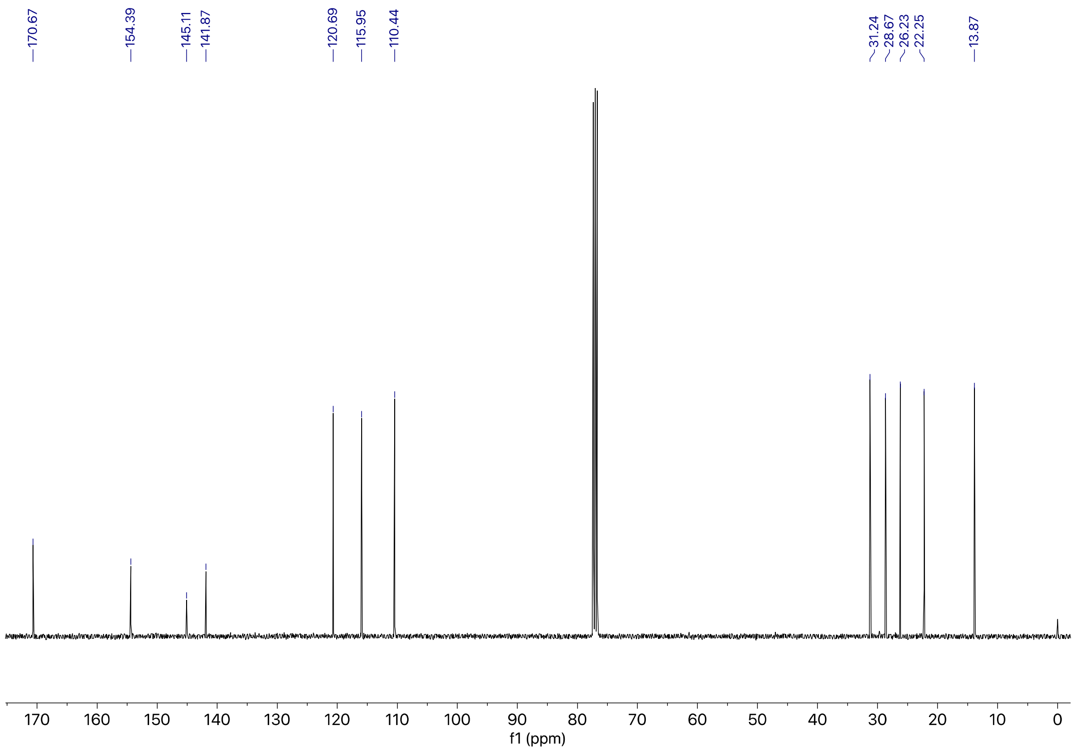

^{1}H and ^{13}C NMR for 2-(*tert*-Butyl)-5-nitrobenzo[*d*]oxazole (**41**)

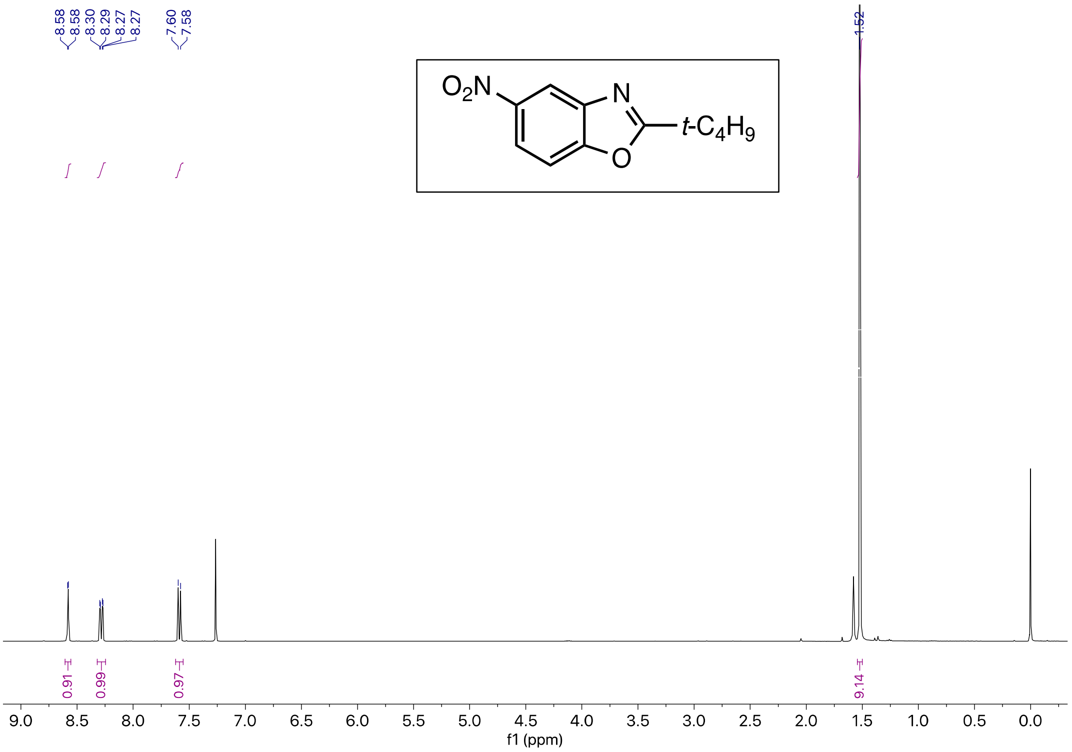

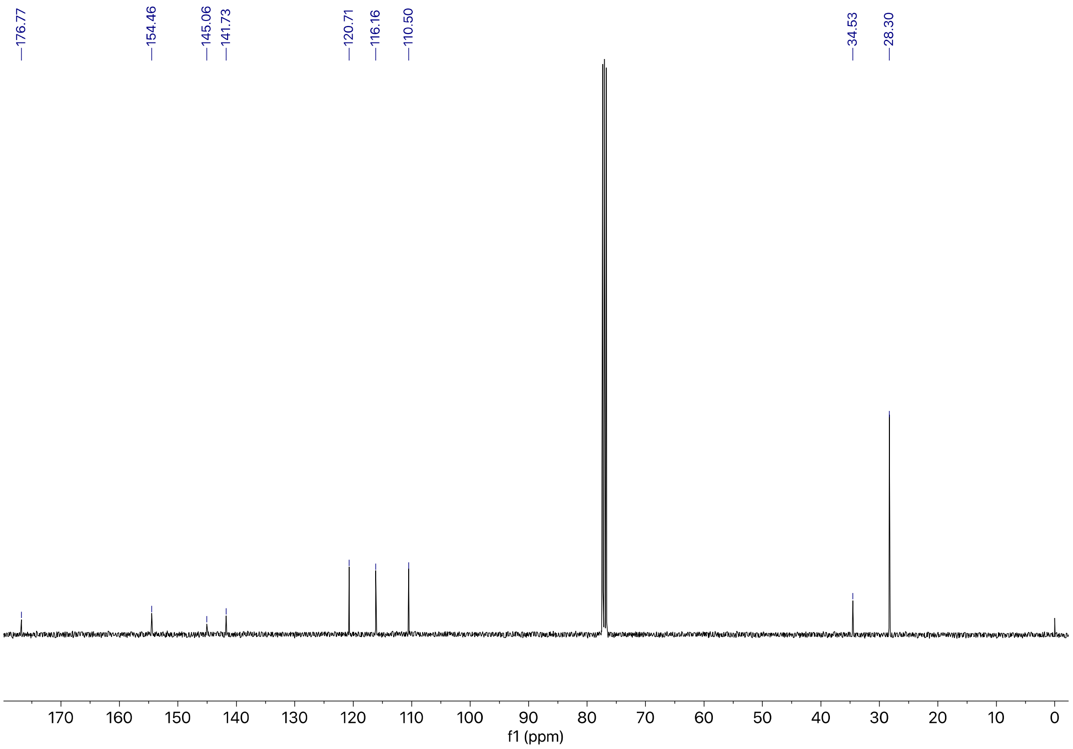

^{1}H and ^{13}C NMR for 5-Nitro-2-phenylbenzo[*d*]oxazole (**42**)

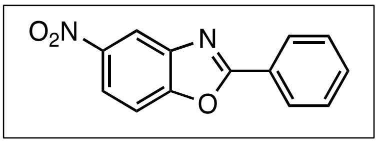

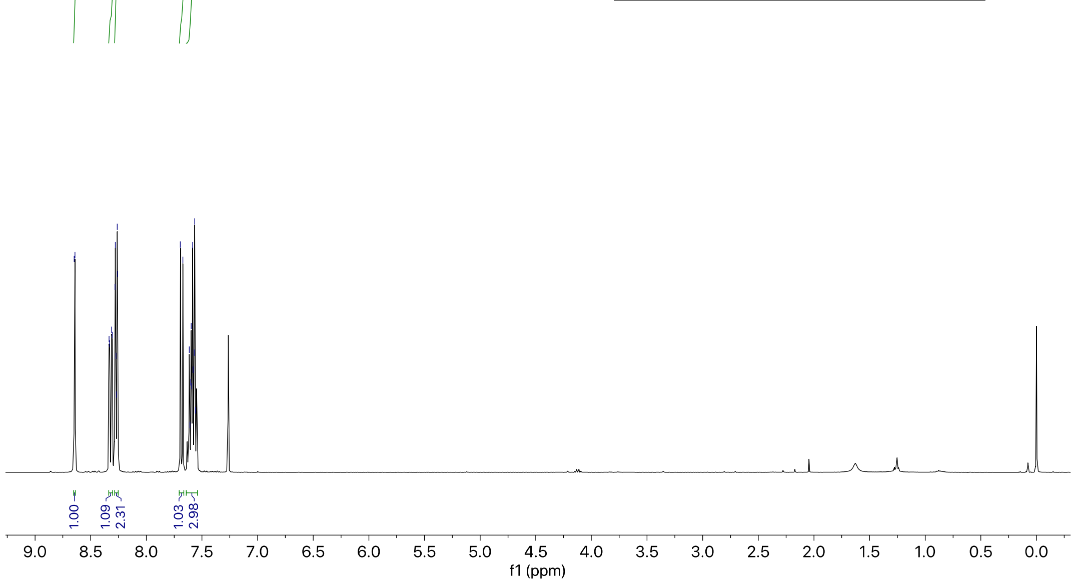

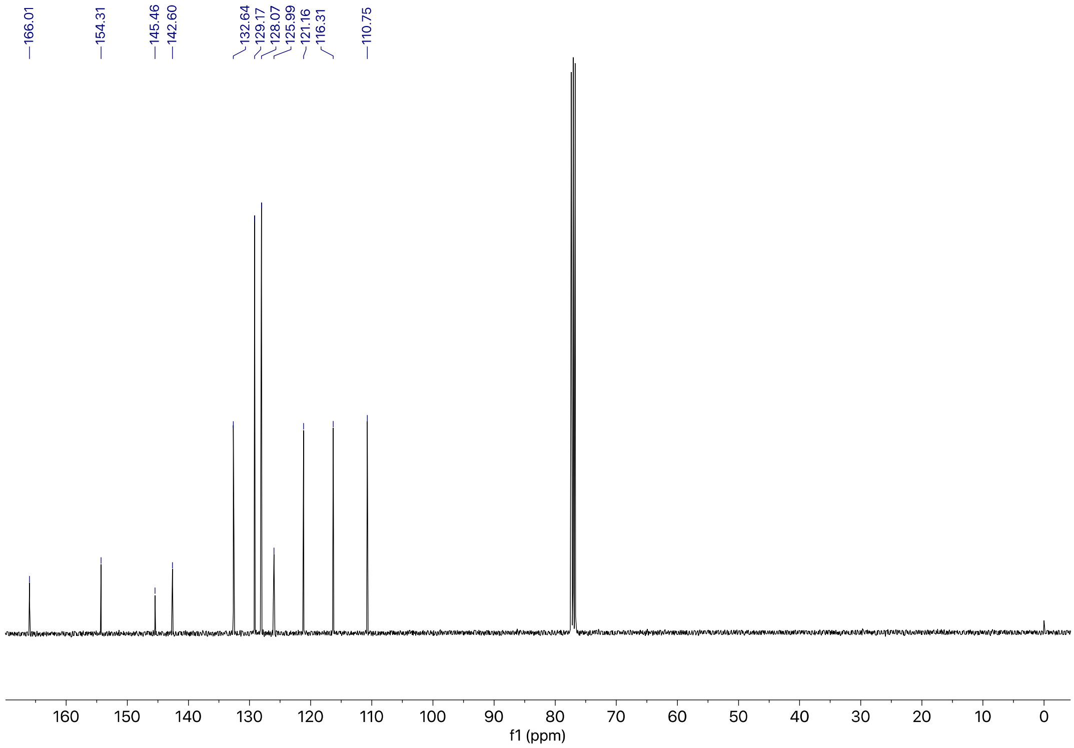

^{1}H and ^{13}C NMR for 2-(3-Methylphenyl)-5-nitrobenzo[*d*]oxazole (**43**)

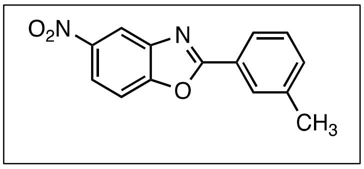

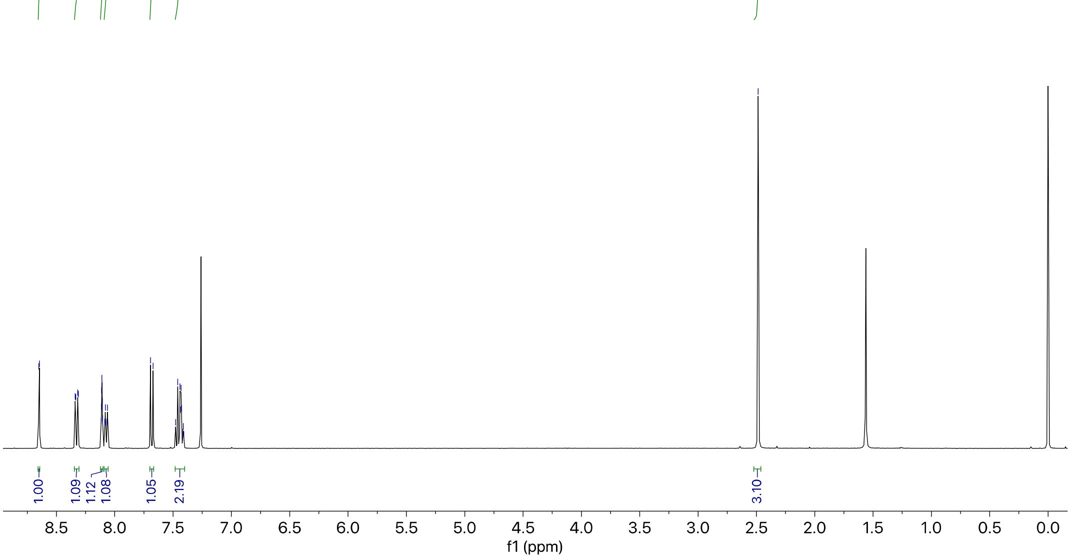

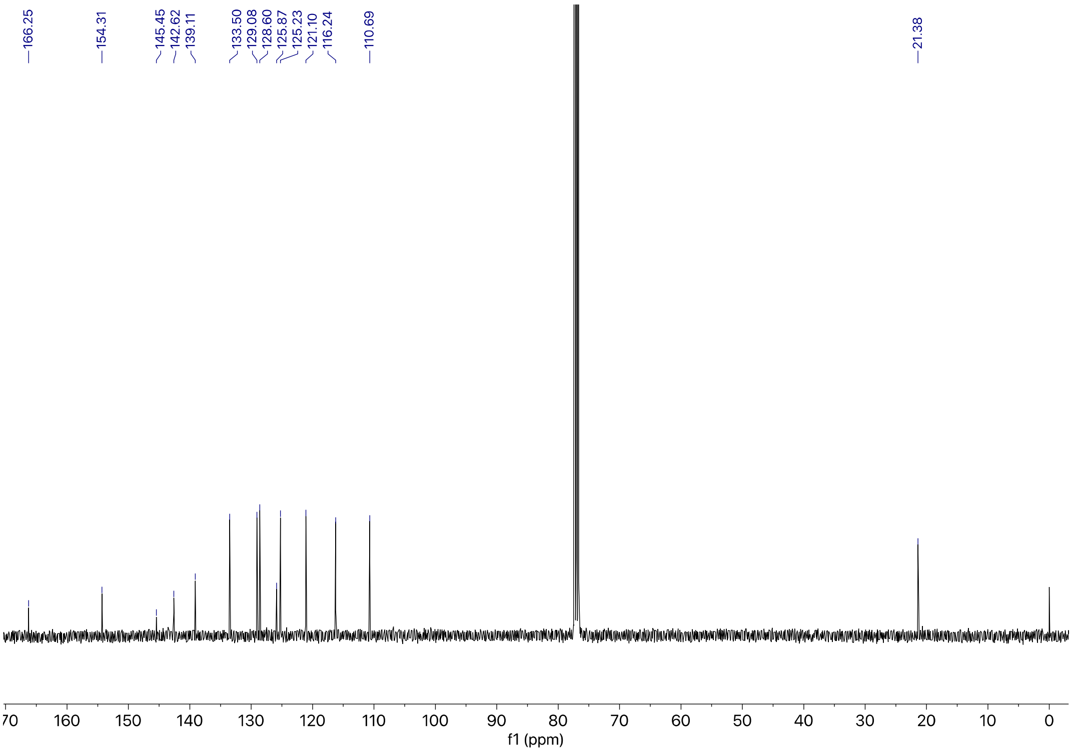

^{1}H and ^{13}C NMR for 2-(4-Methylphenyl)-5-nitrobenzo[*d*]oxazole (**44**)

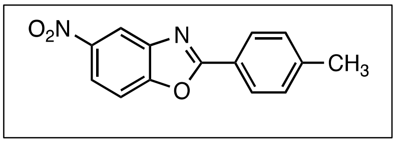

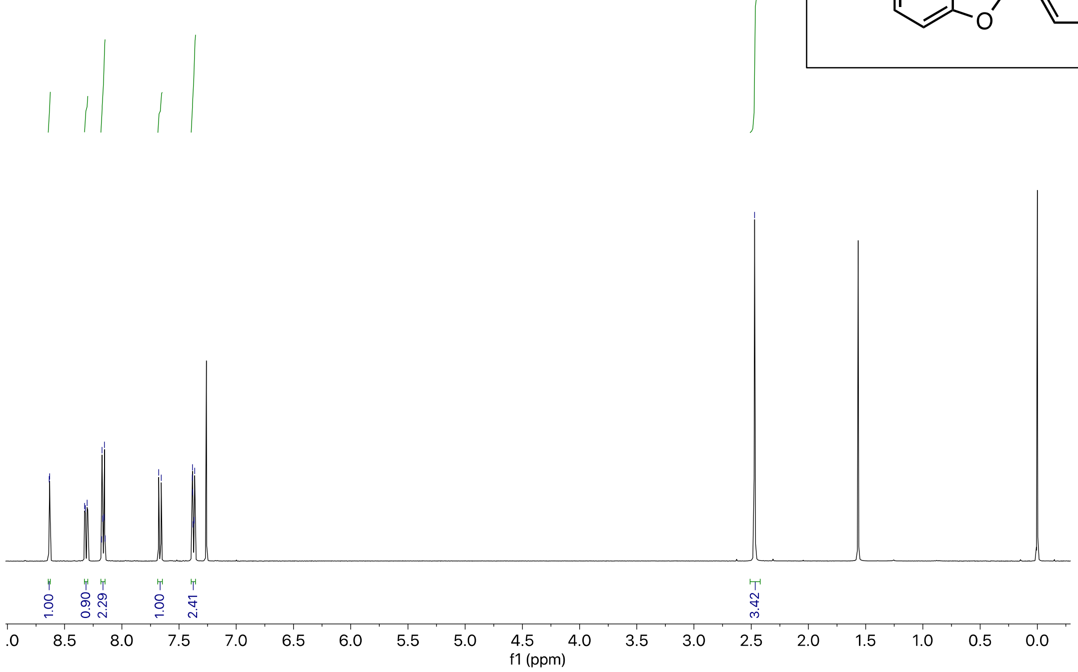

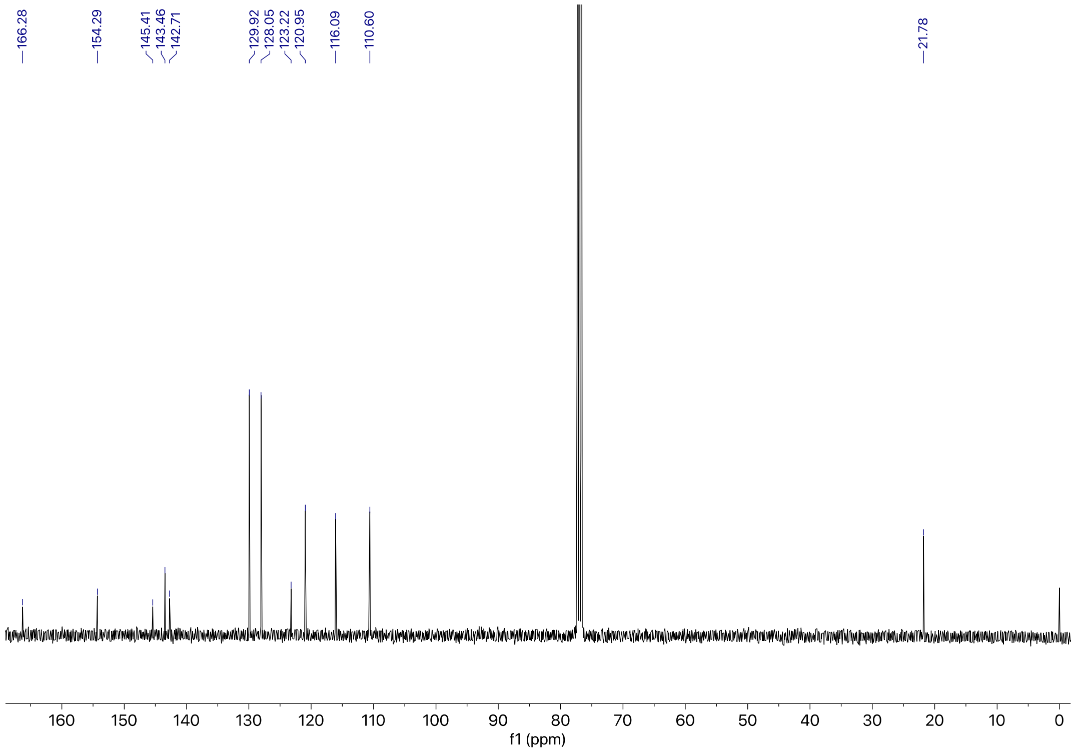

^{1}H and ^{13}C NMR for 2-(4-Methoxyphenyl)-5-nitrobenzo[*d*]oxazole (**45**)

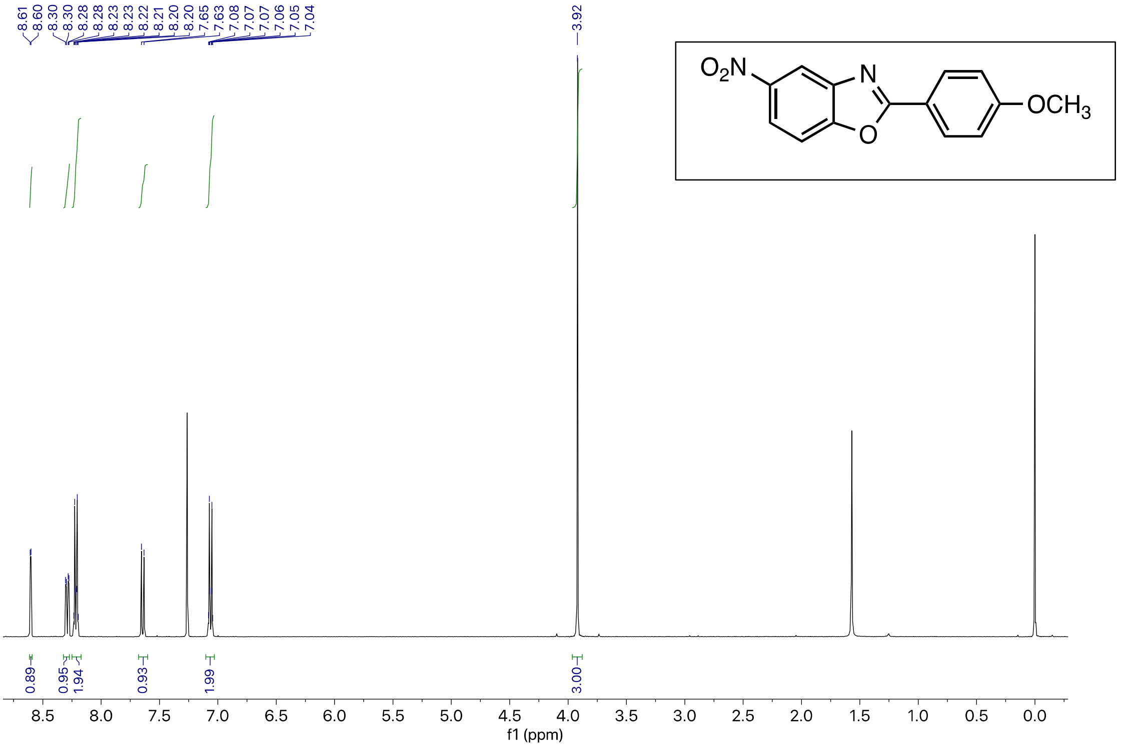

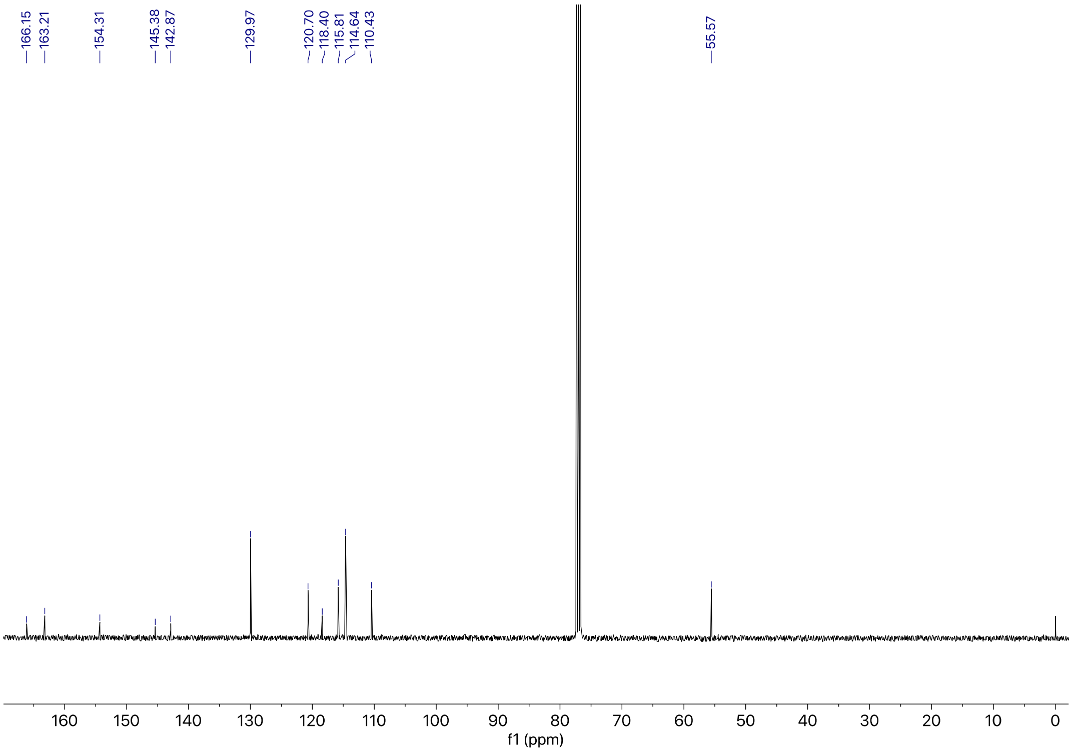

^{1}H and ^{13}C NMR for 2-(2-Fluorophenyl)-5-nitrobenzo[*d*]oxazole (**46**)

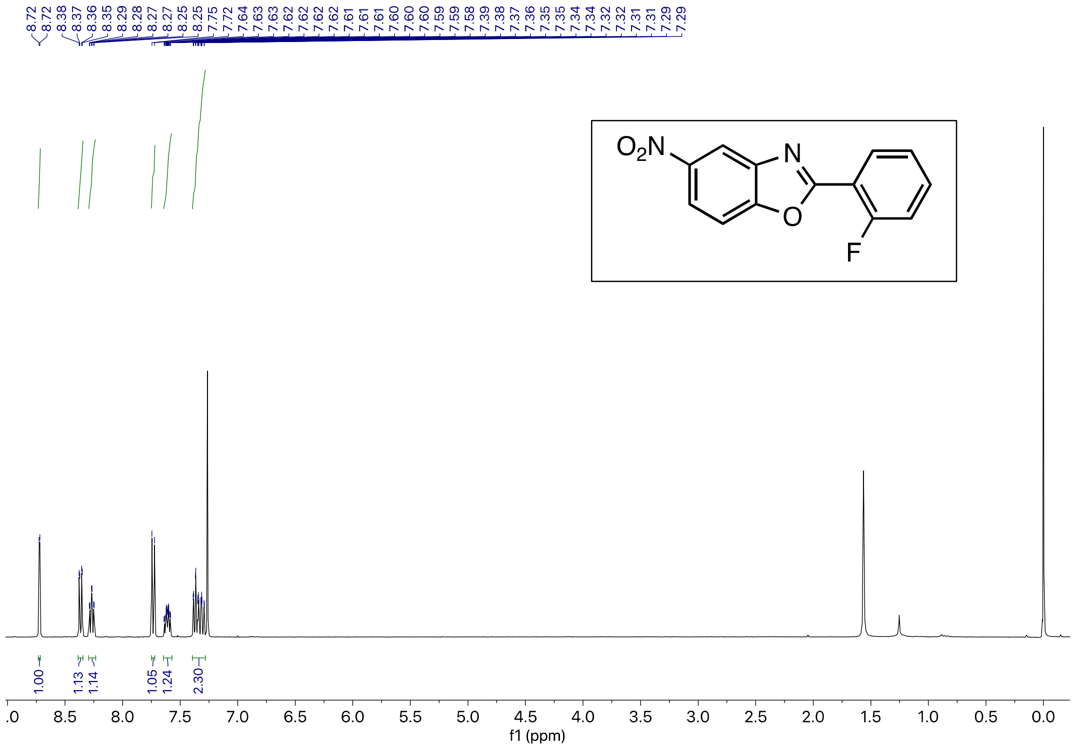

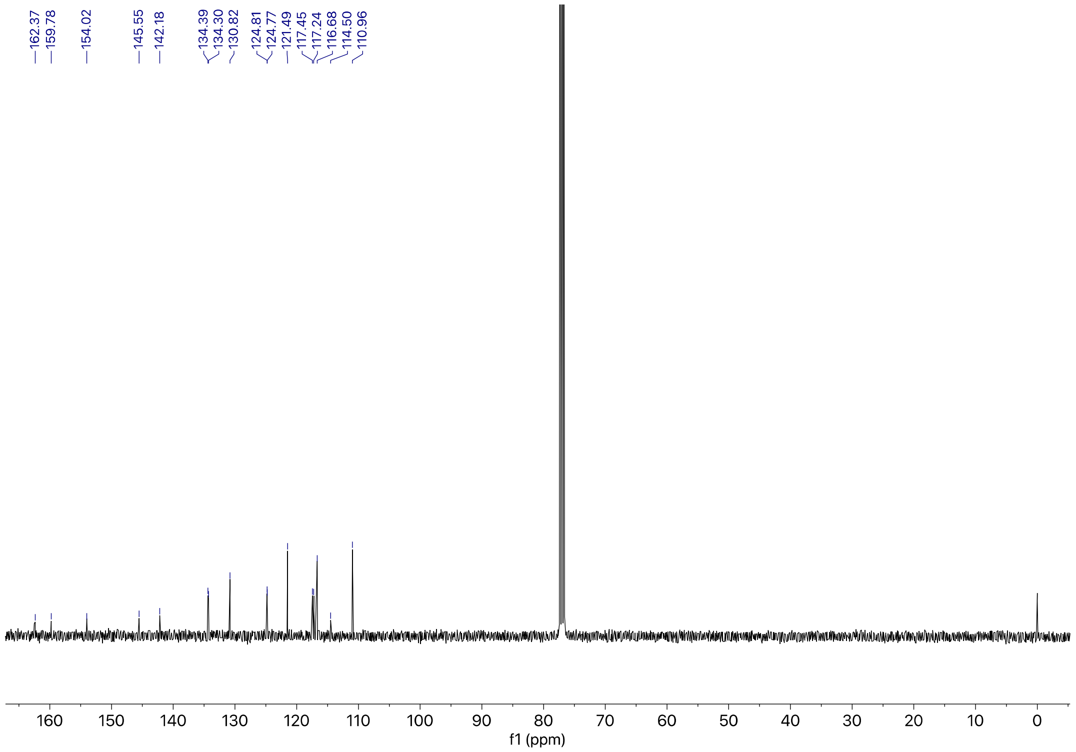

^{1}H and ^{13}C NMR for 2-(3-Chlorophenyl)-5-nitrobenzo[*d*]oxazole (**47**)

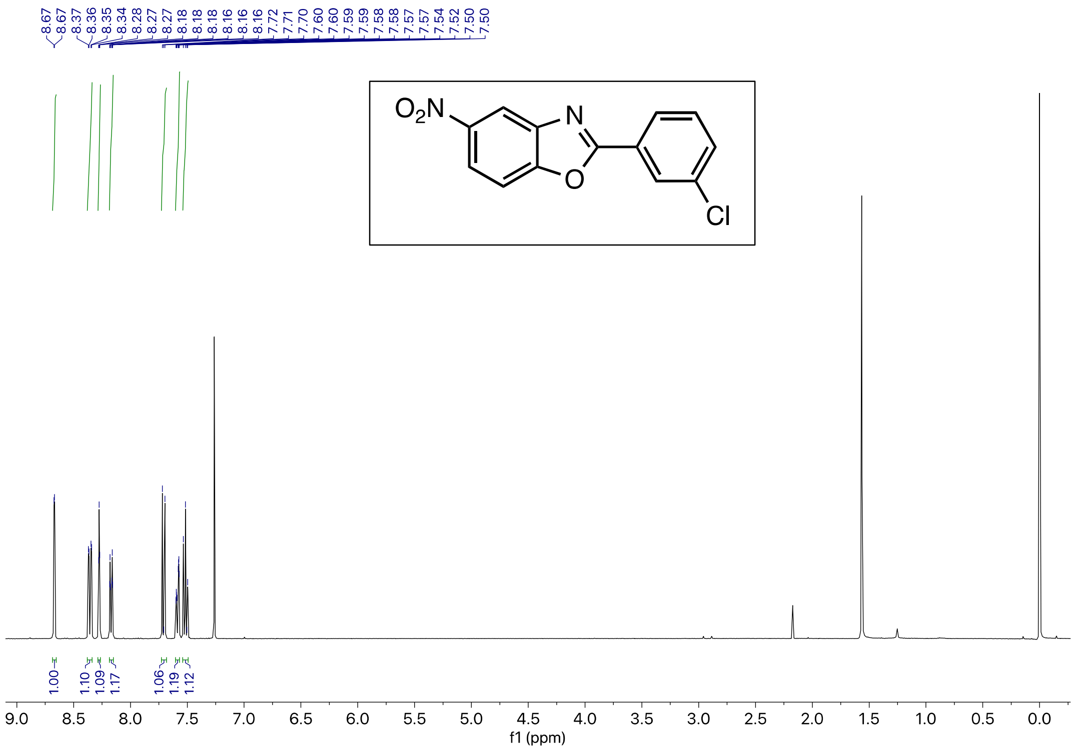

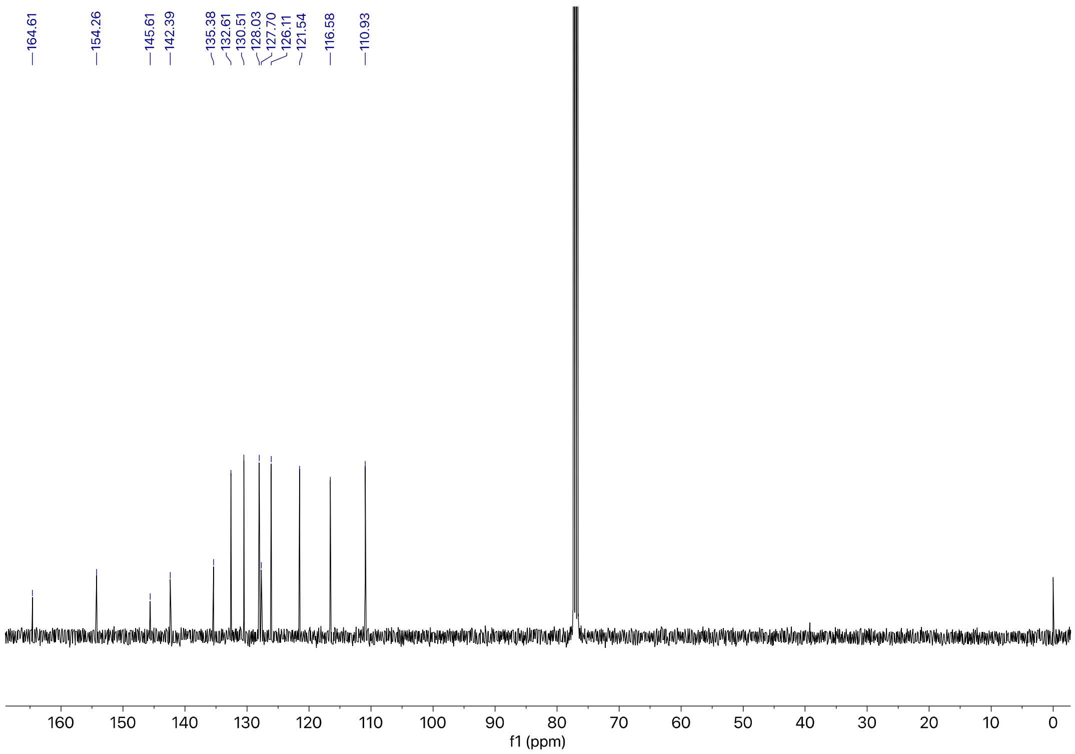

^{1}H and ^{13}C NMR for 2-(4-Chlorophenyl)-5-nitrobenzo[*d*]oxazole (**48**)

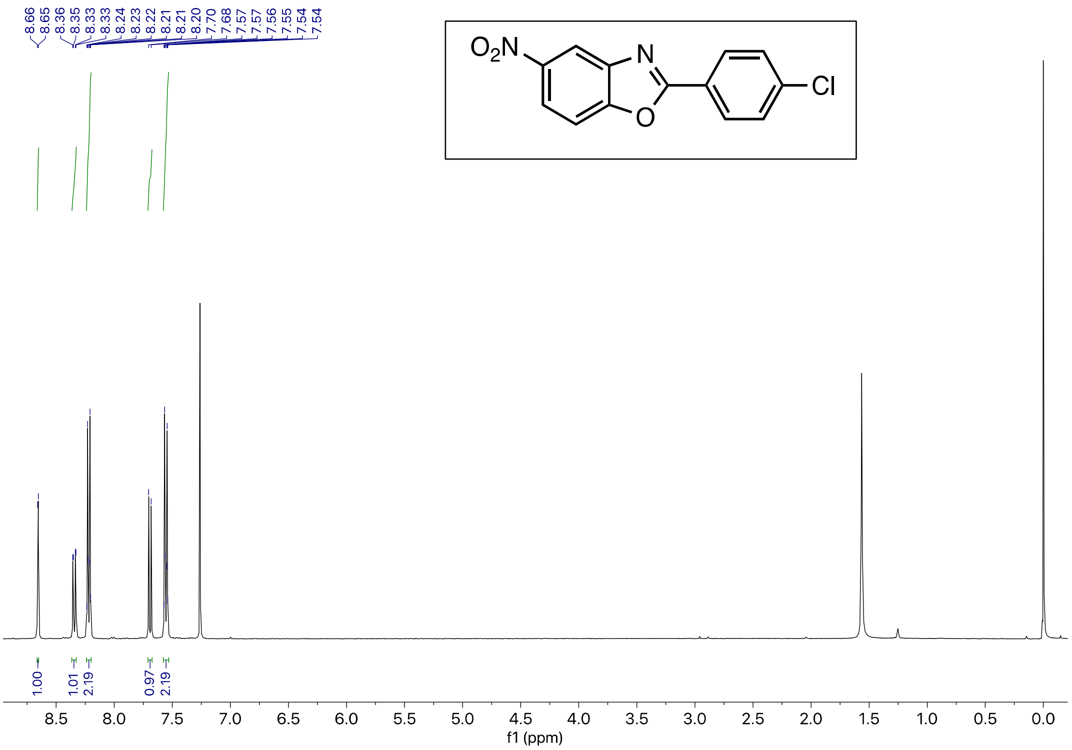

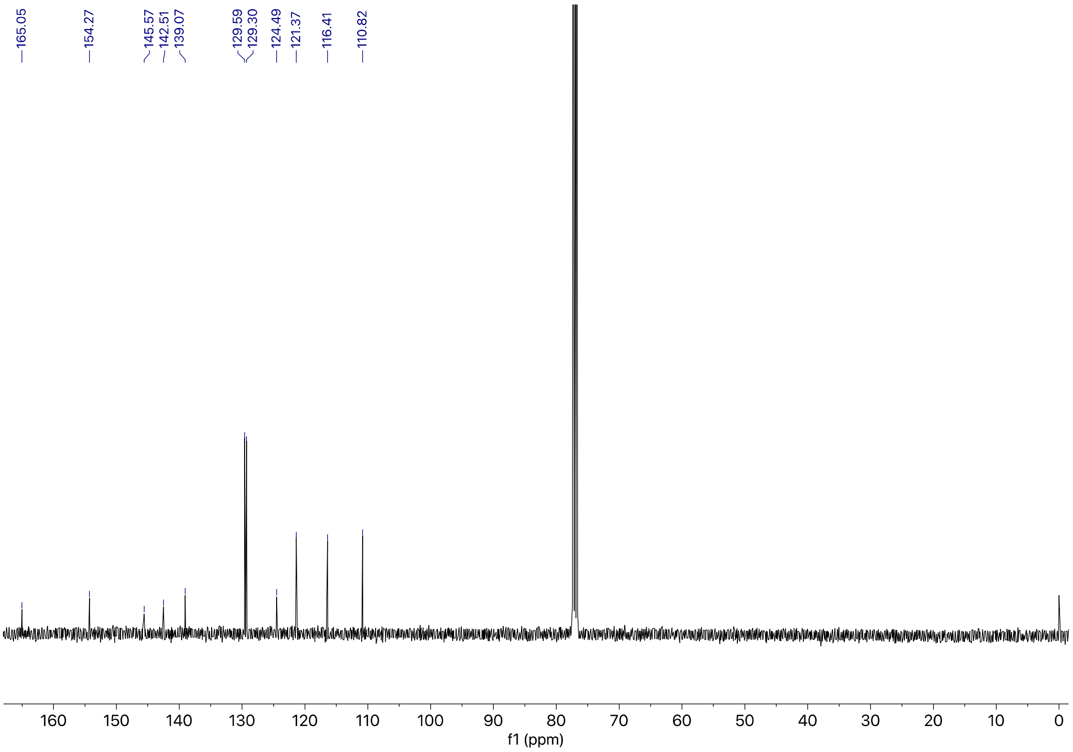

^{1}H and ^{13}C NMR for 2-Methylbenzo[*d*]oxazole-5-carbonitrile (**49**)

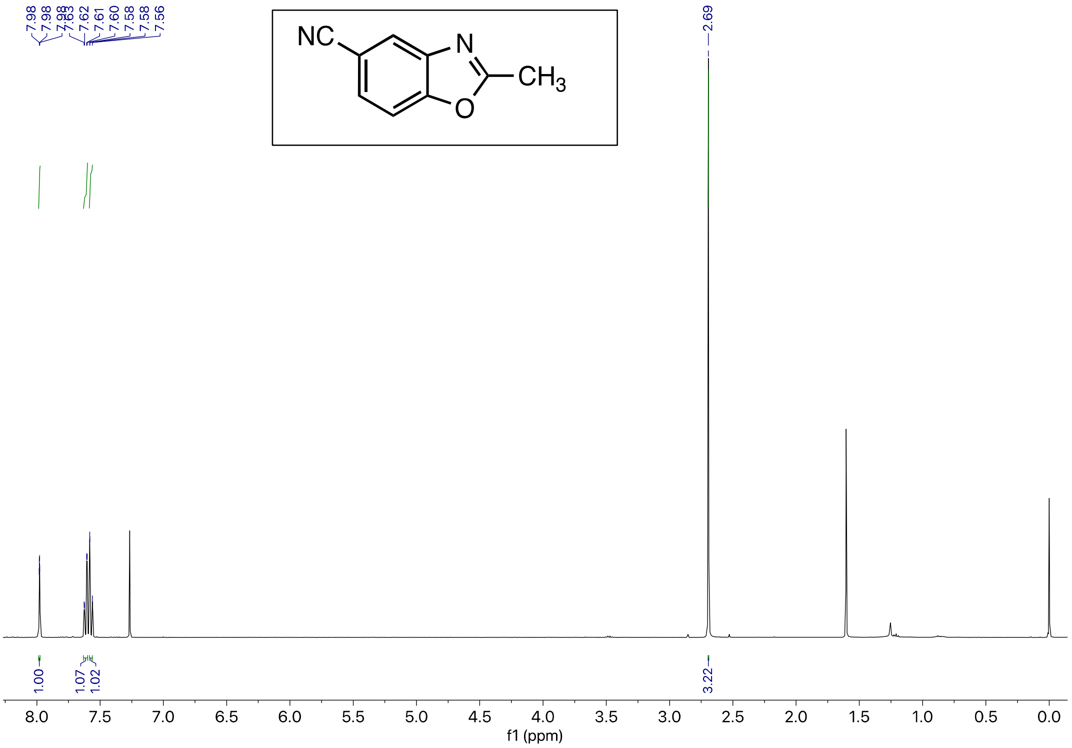

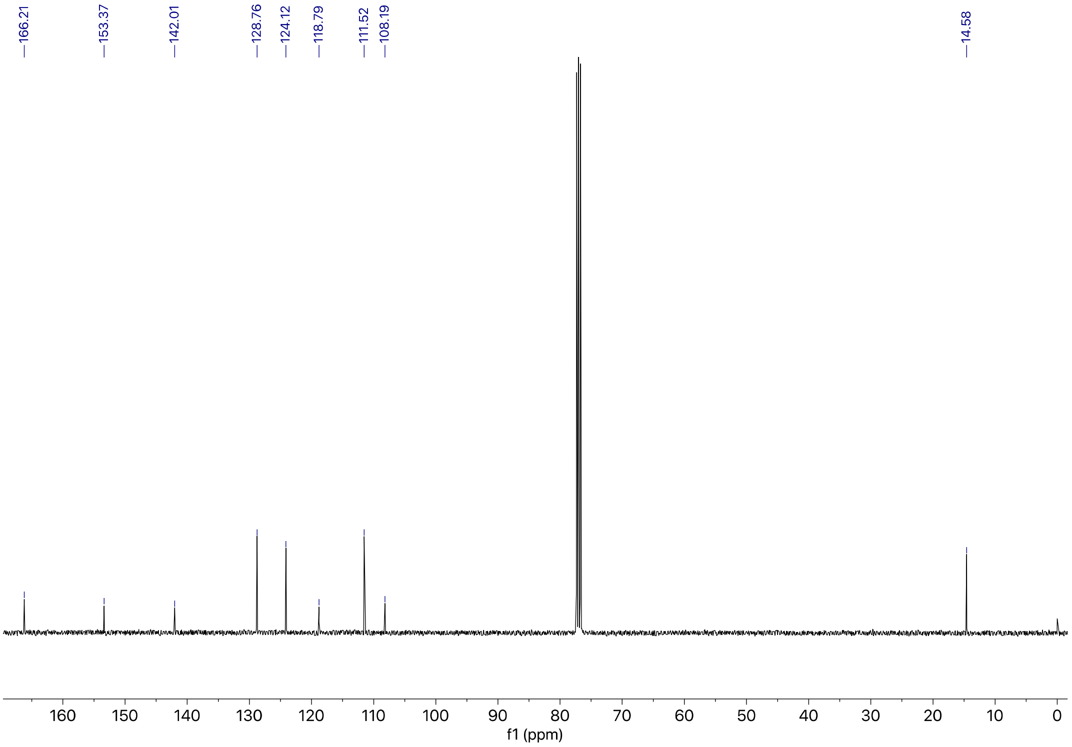

^{1}H and ^{13}C NMR for 2-Pentylbenzo[*d*]oxazole-5-carbonitrile (**50**)

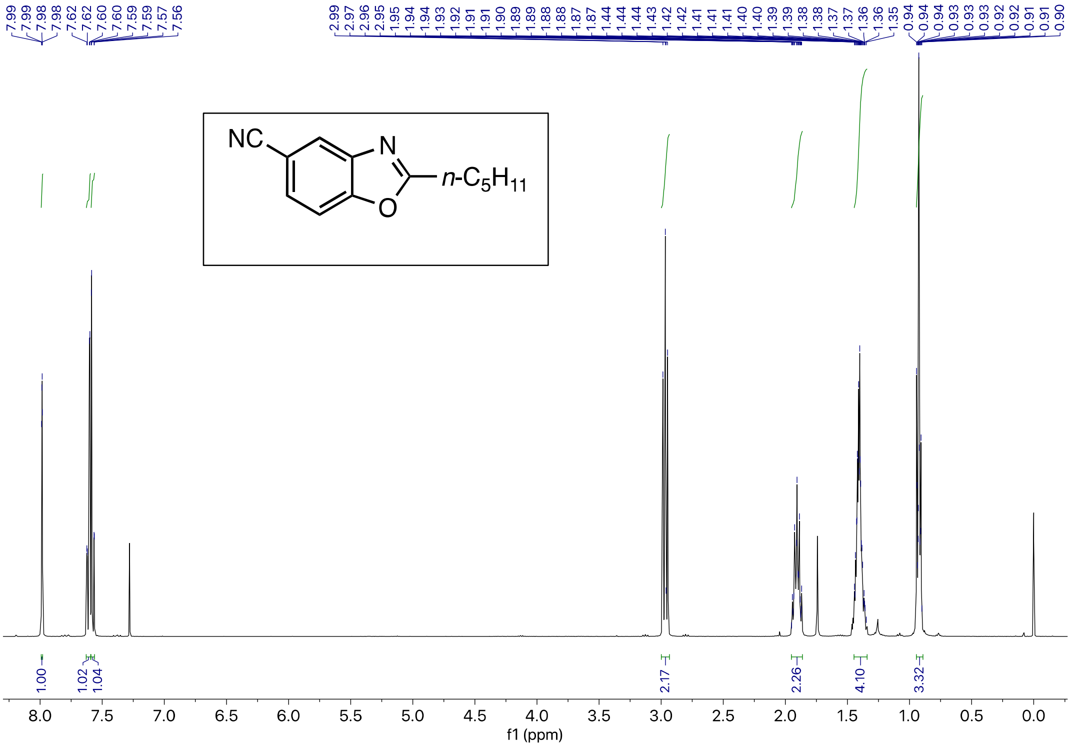

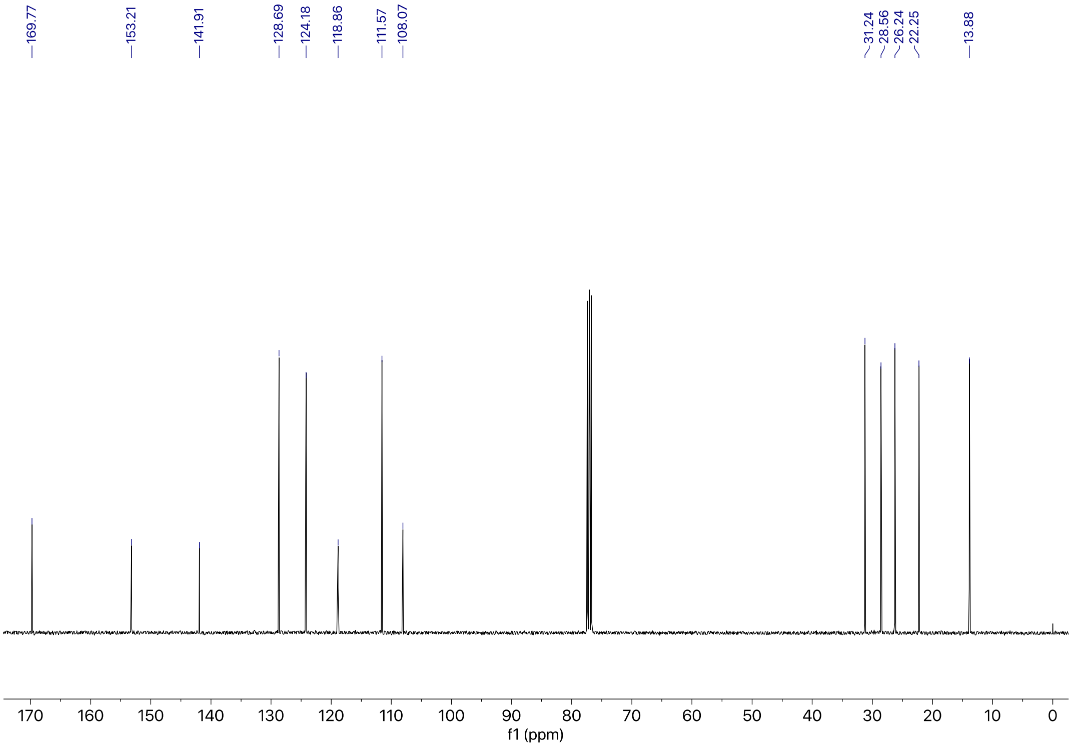

^{1}H and ^{13}C NMR for 2-(*tert*-Butyl) benzo[*d*]oxazole-5-carbonitrile (**51**)

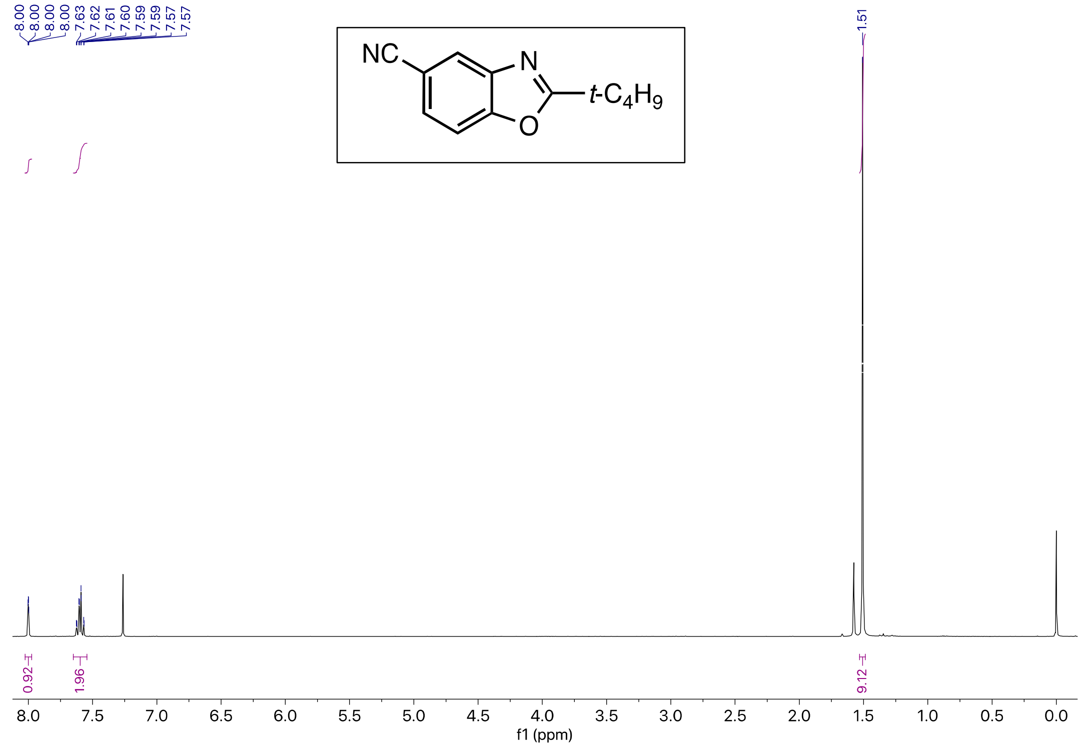

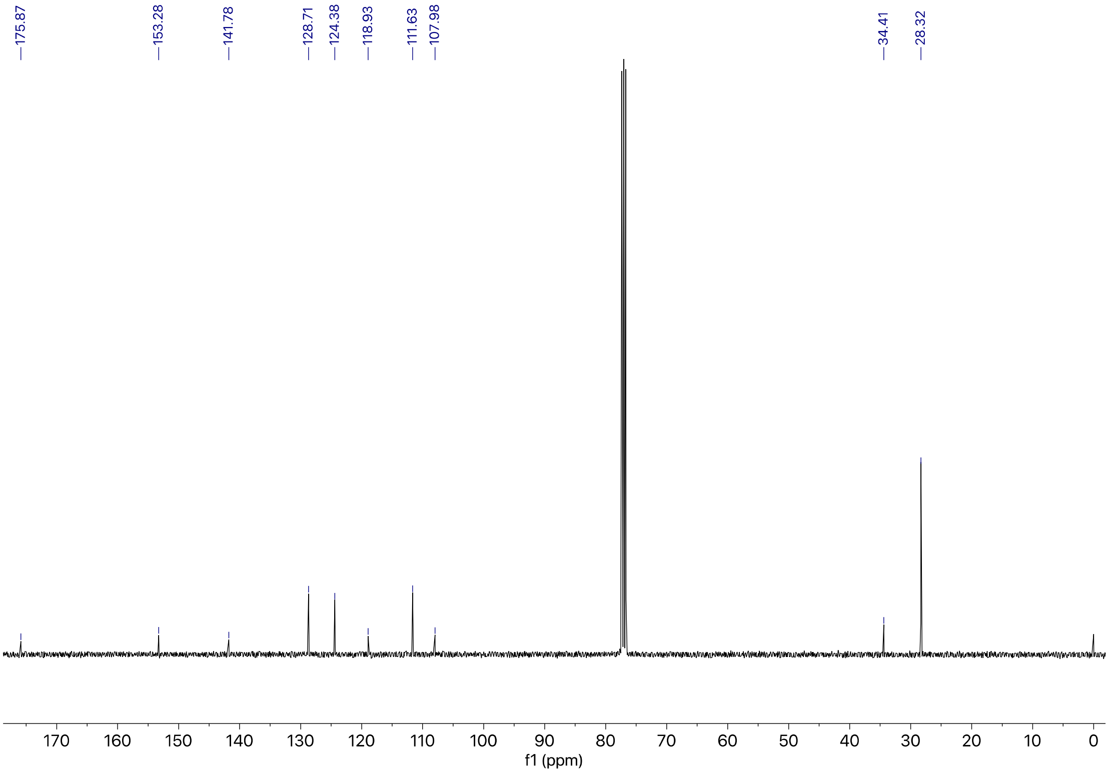

^1H and ^{13}C NMR for 2-Phenylbenzo[*d*]oxazole-5-carbonitrile (**52**)

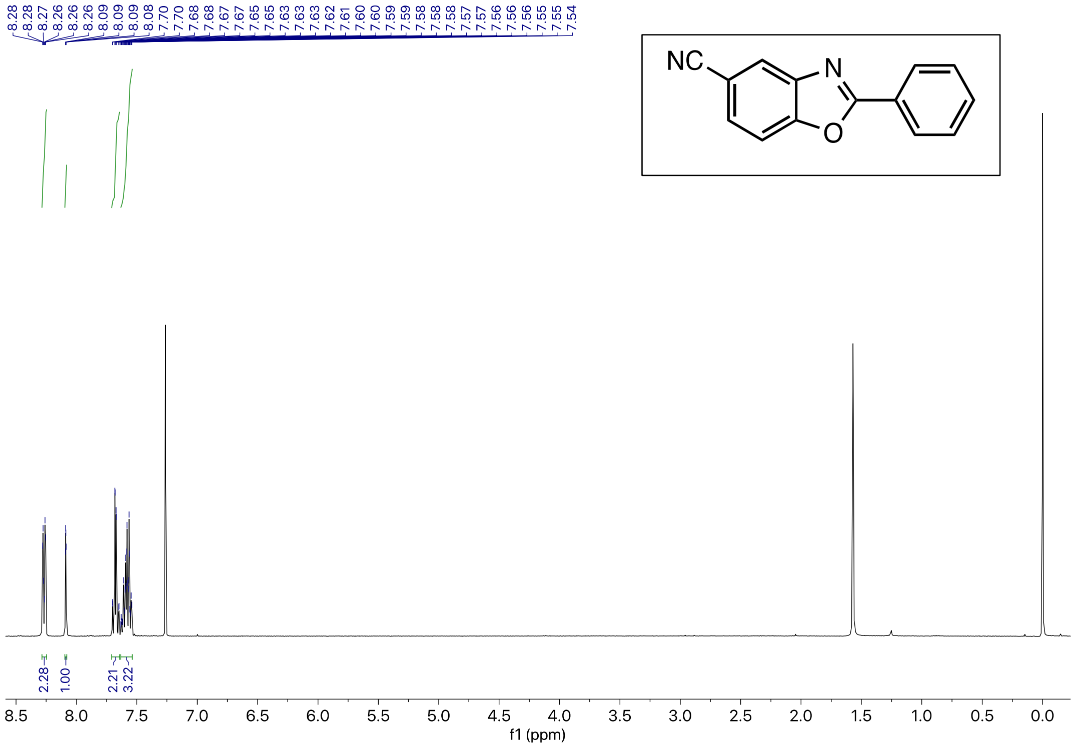

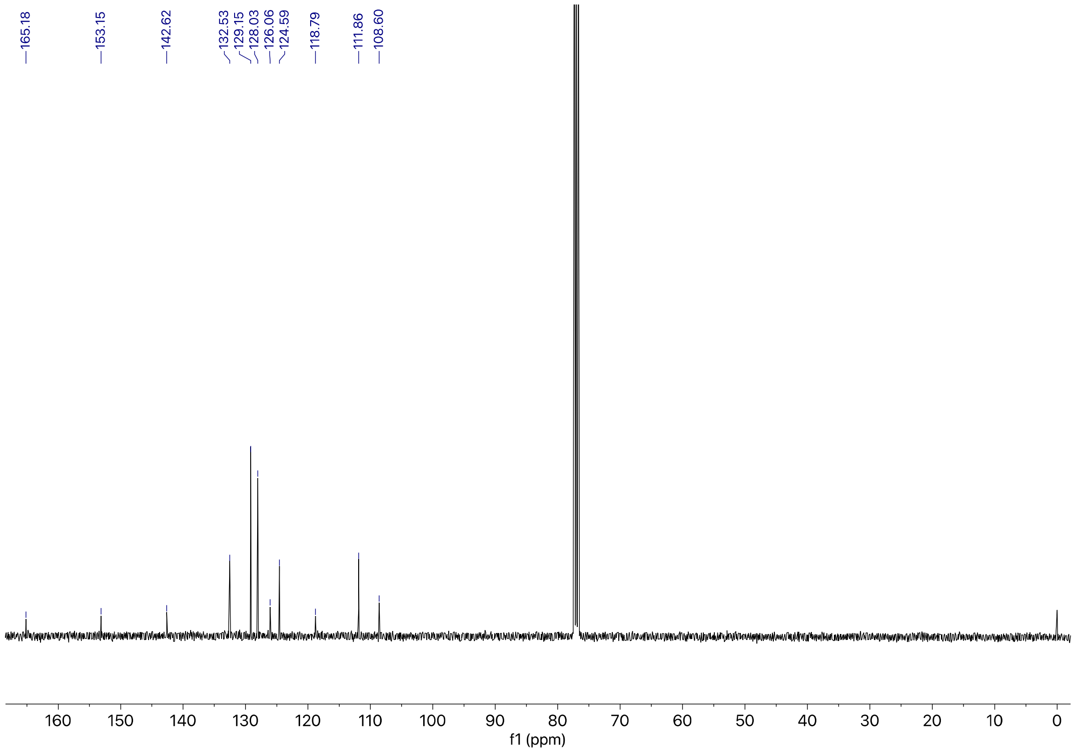

^{1}H and ^{13}C NMR for 2-(3-Methylphenyl)benzo[*d*]oxazole-5-carbonitrile (**53**)

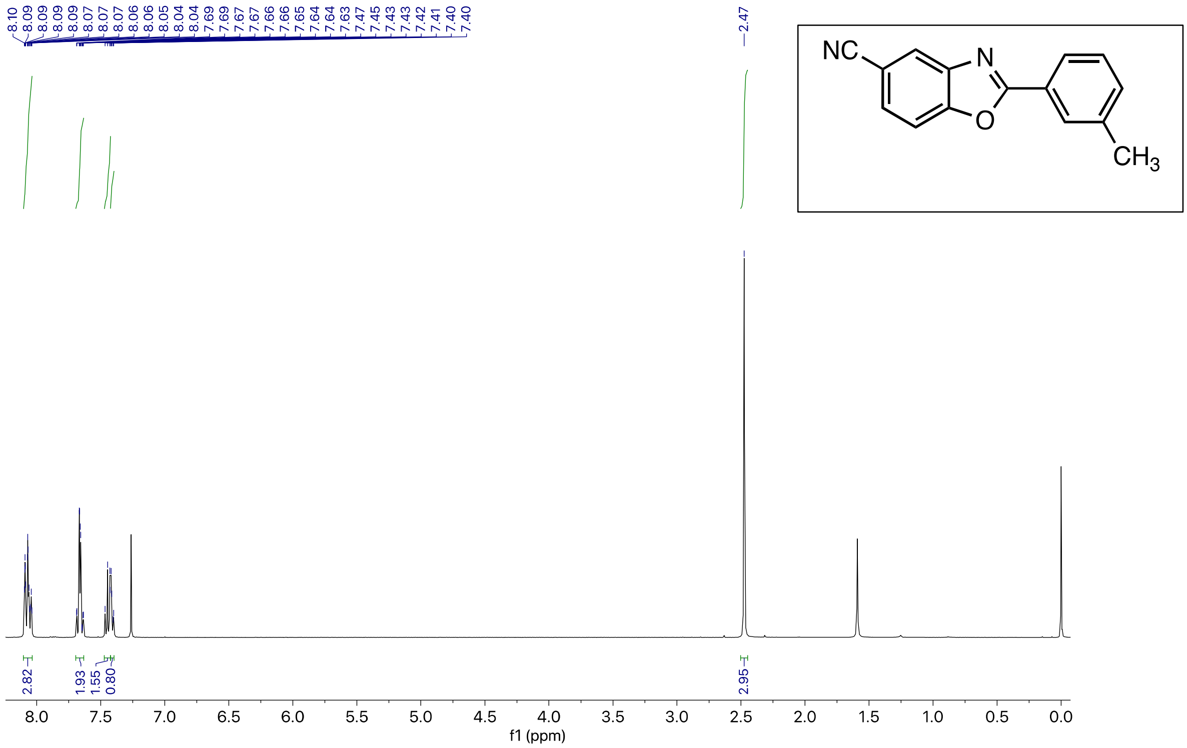

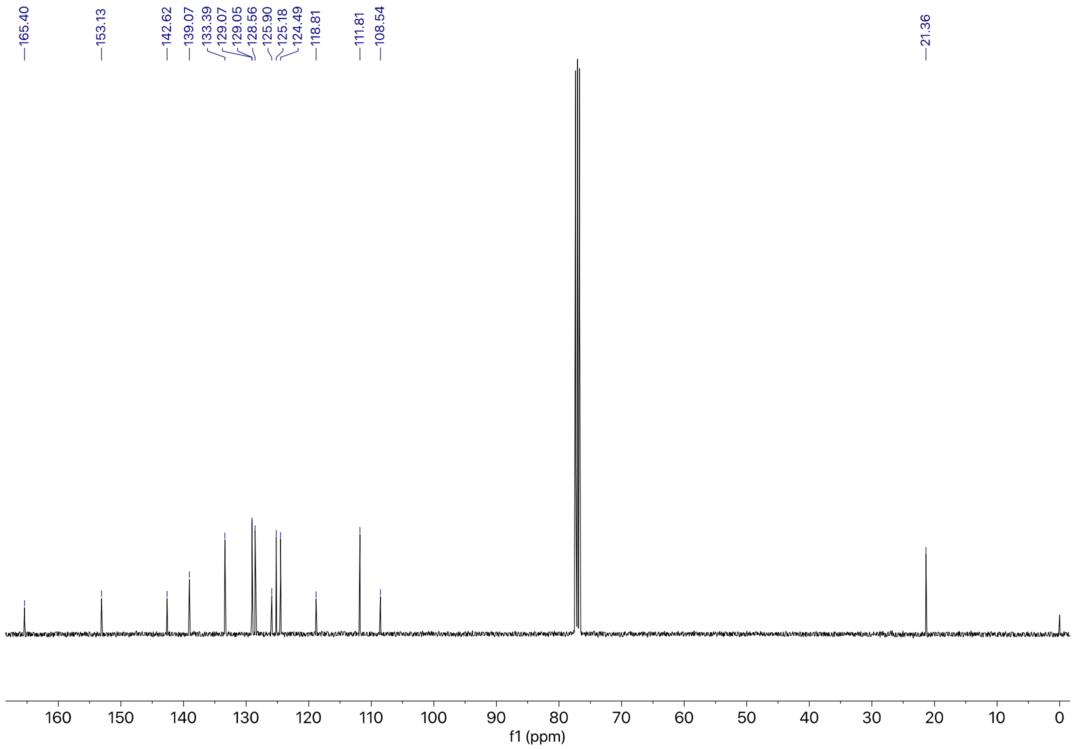

^{1}H and ^{13}C NMR for 2-(4-Methylphenyl)benzo[*d*]oxazole-5-carbonitrile (**54**)

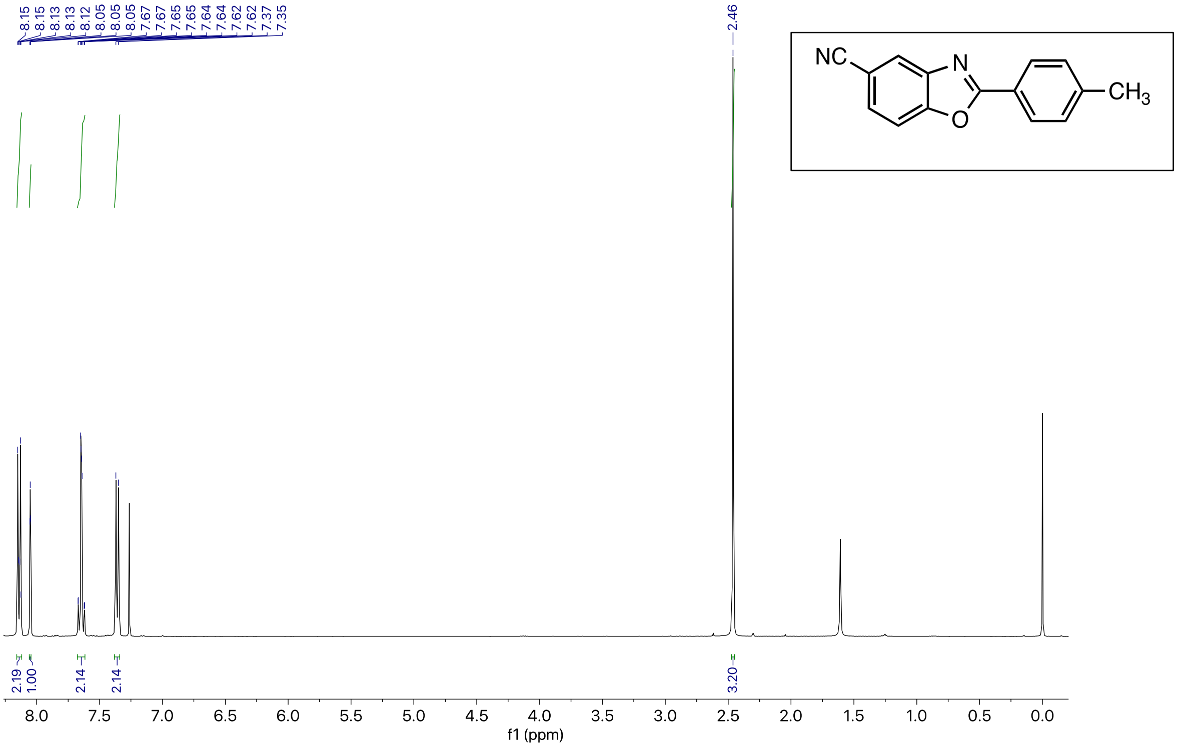

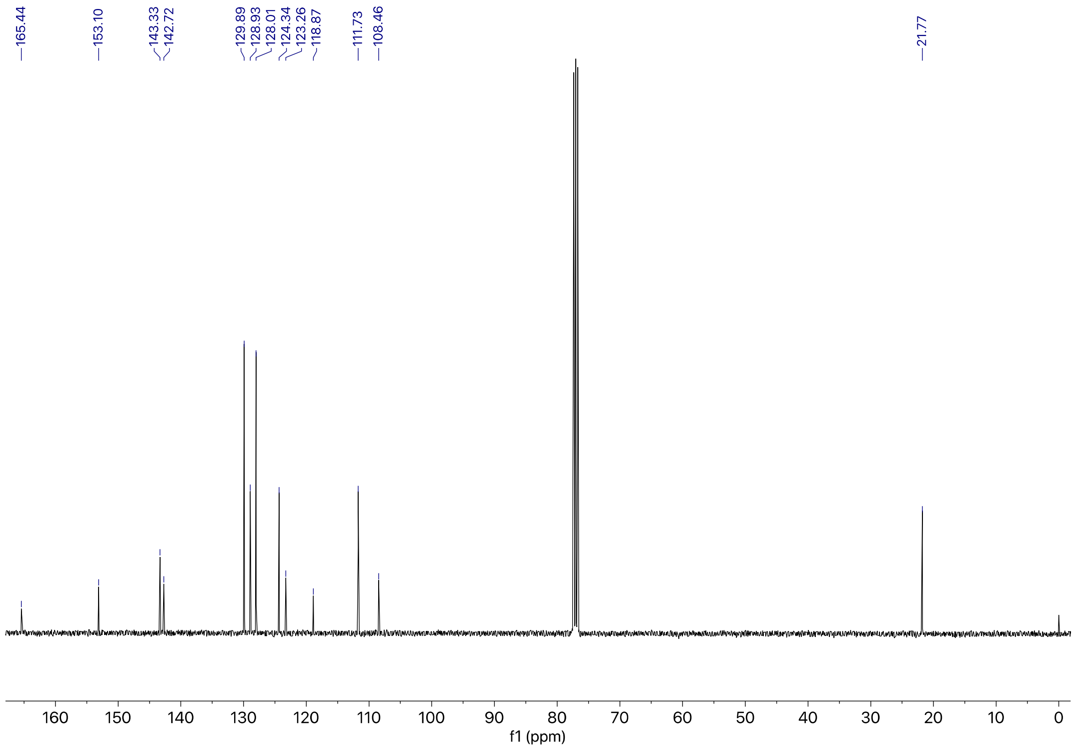

^{1}H and ^{13}C NMR for 2-(4-Methoxyphenyl)benzo[*d*]oxazole-5-carbonitrile (**55**)

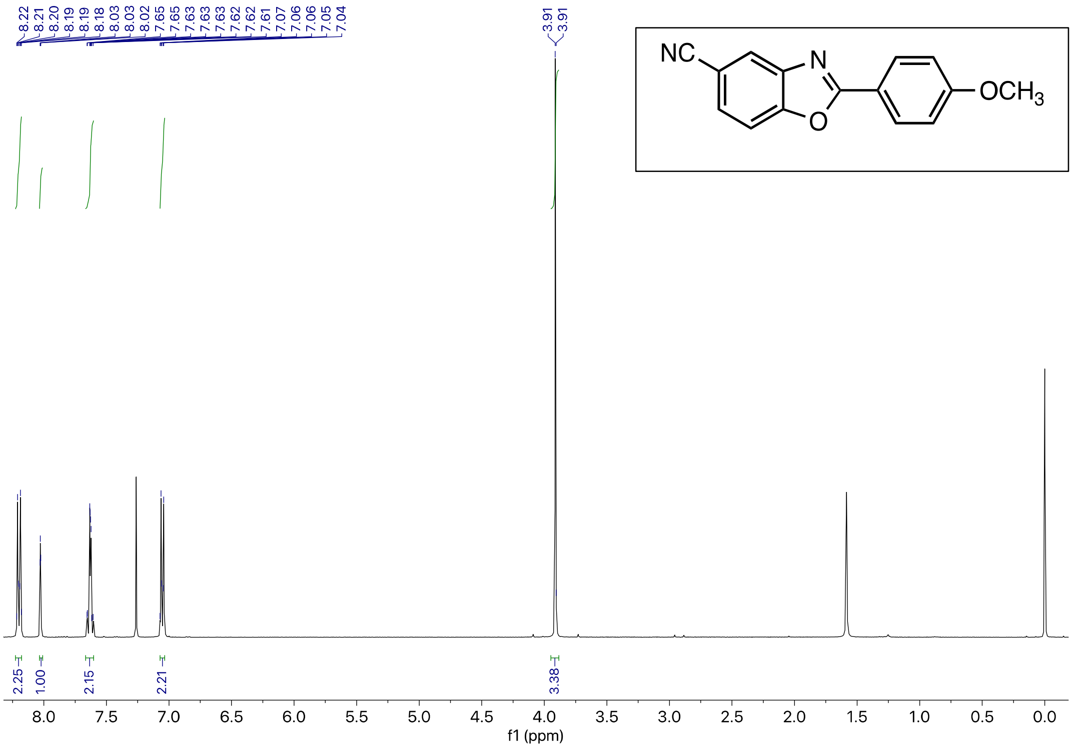

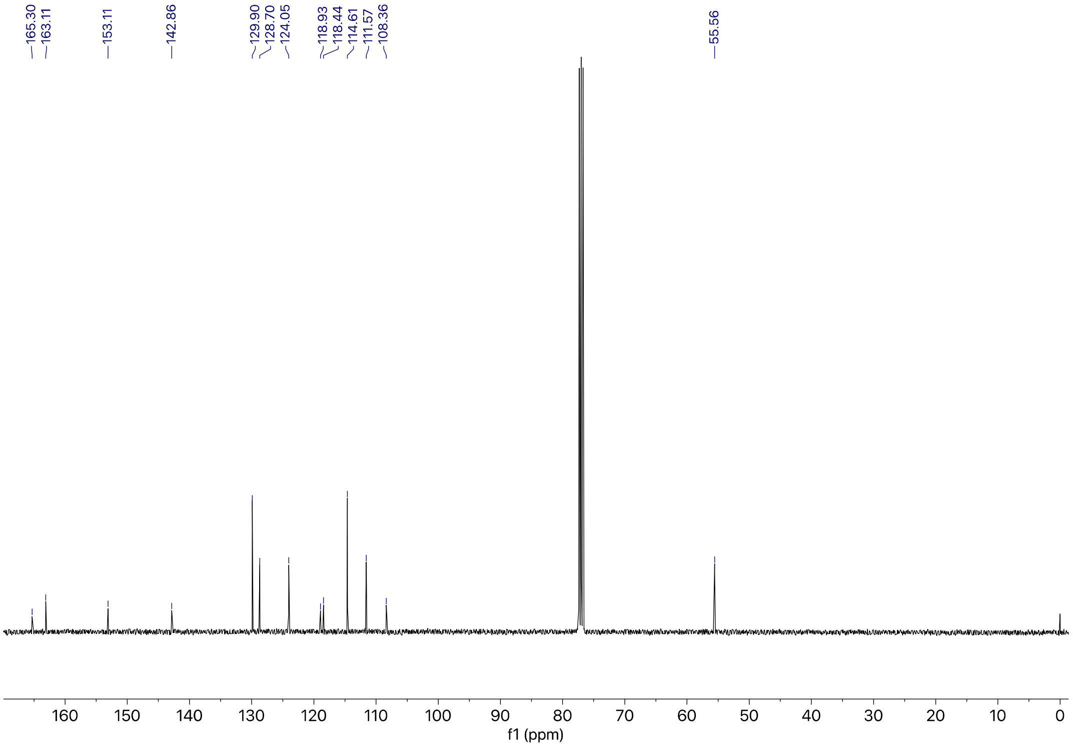

^{1}H and ^{13}C NMR for 2-(2-Fluorophenyl)benzo[*d*]oxazole-5-carbonitrile (**56**)

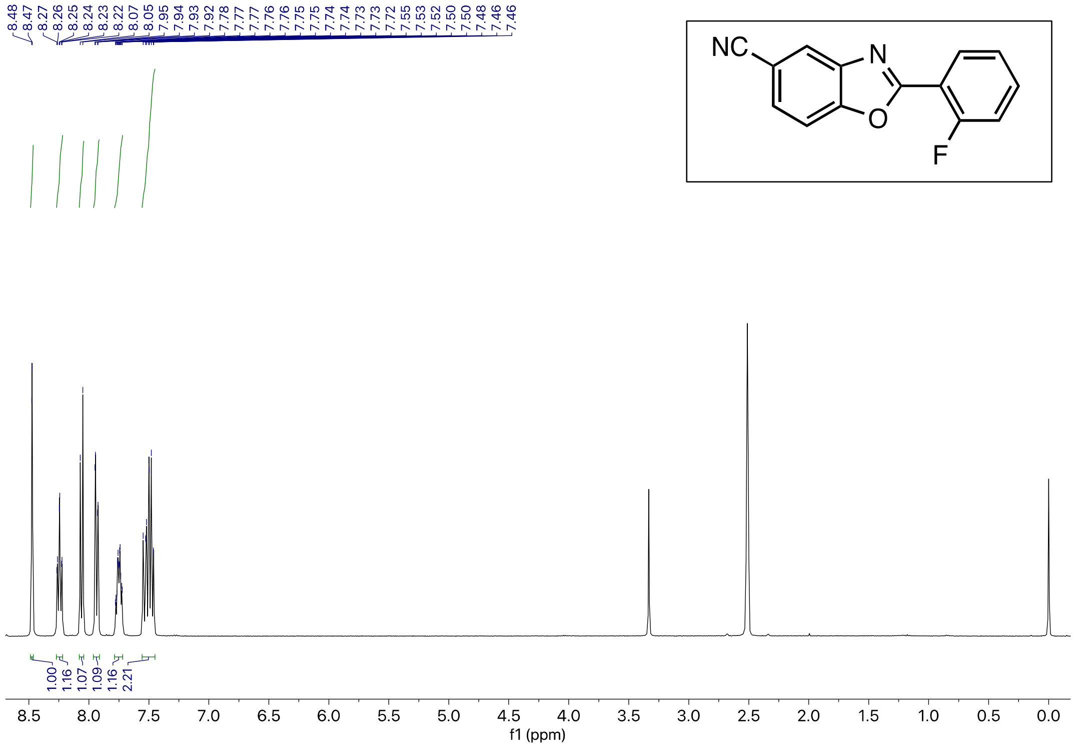

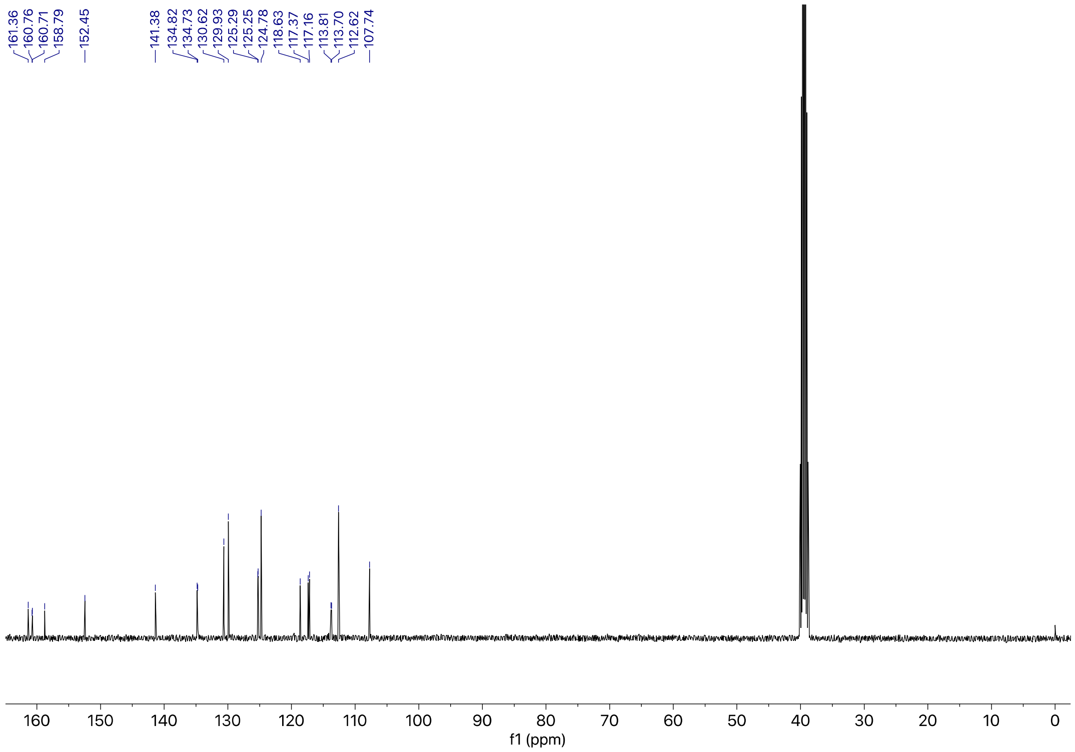

^{1}H and ^{13}C NMR for 2-(3-Chlorophenyl)benzo[*d*]oxazole-5-carbonitrile (**57**)

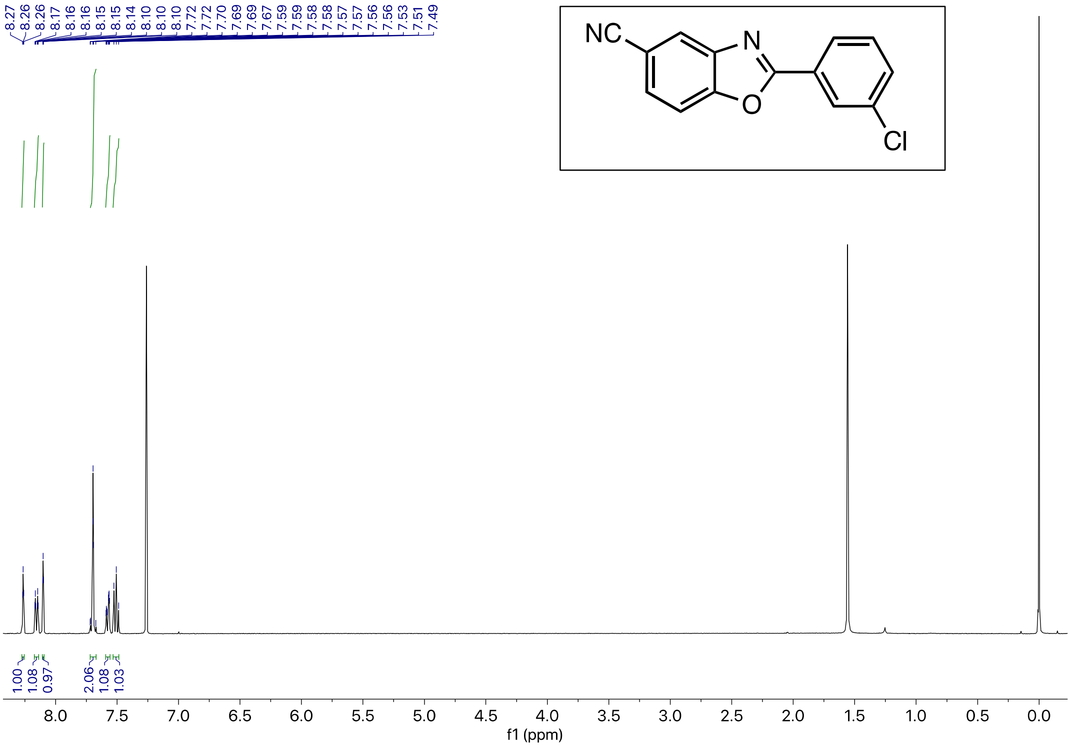

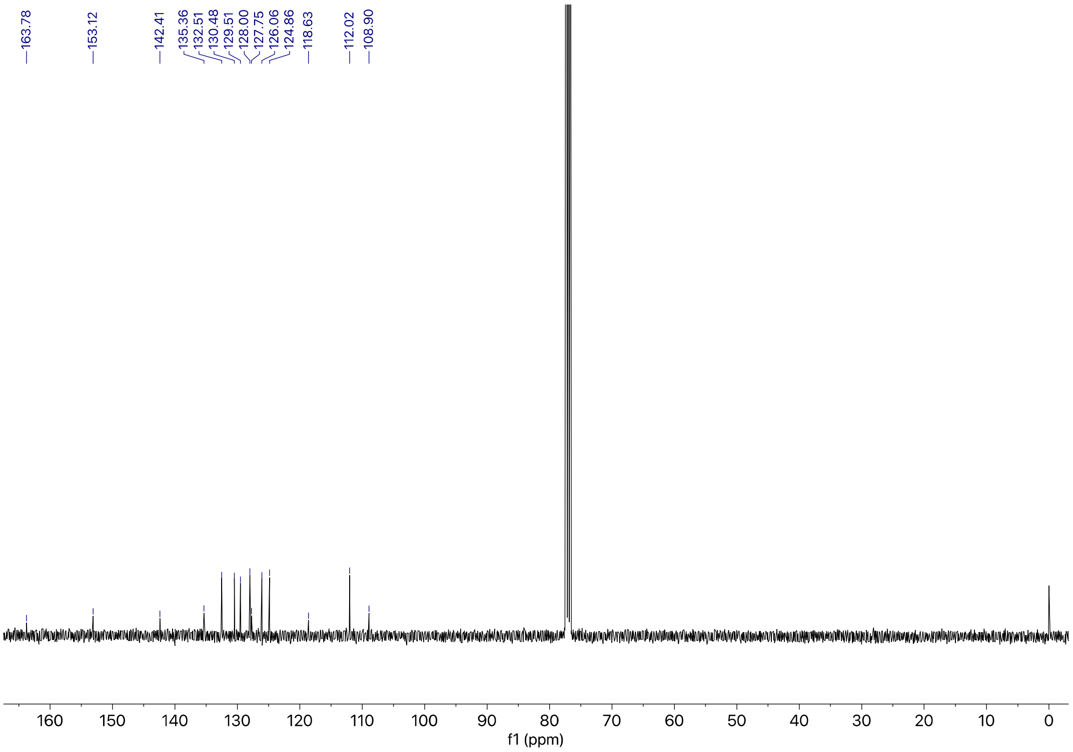

^{1}H and ^{13}C NMR for 2-(4-Chlorophenyl)benzo[*d*]oxazole-5-carbonitrile (**58**)

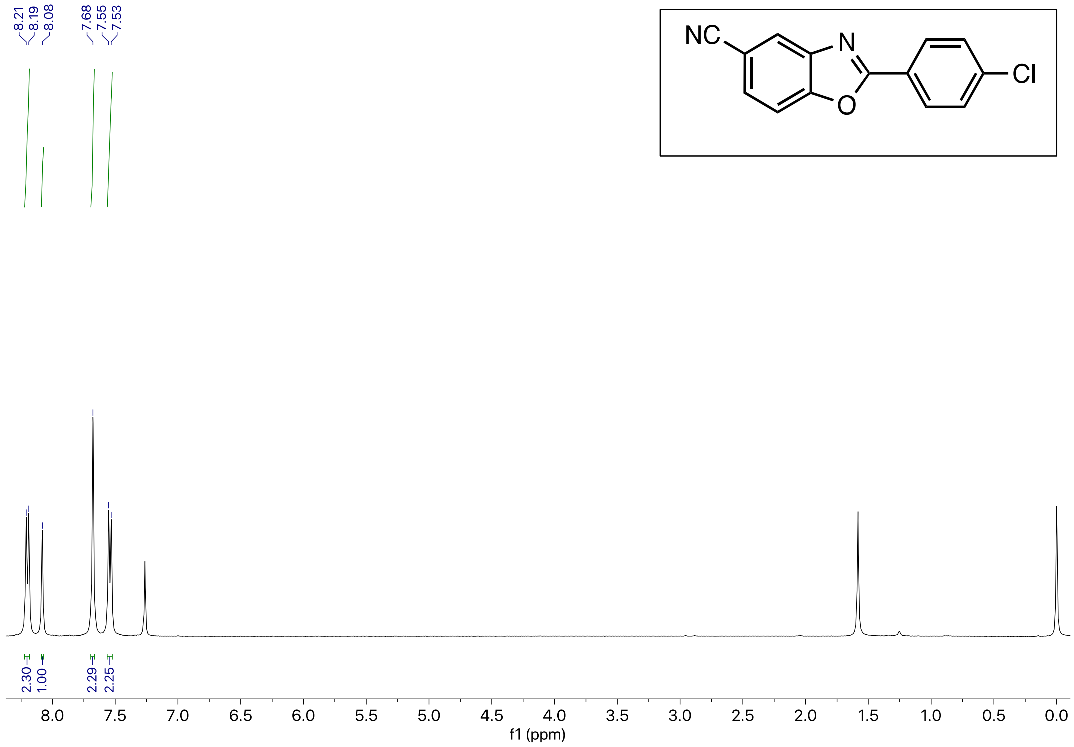

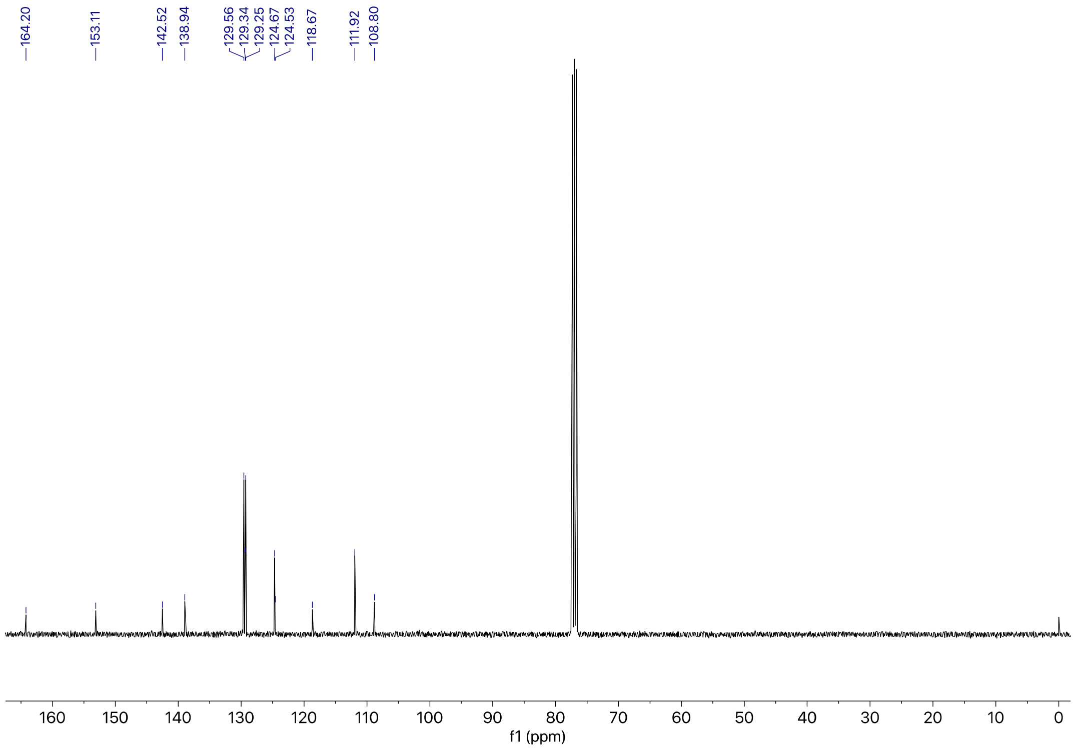

^{1}H and ^{13}C NMR for Methyl 2-phenylbenzo[*d*]oxazole-5-carboxylate (**59**)

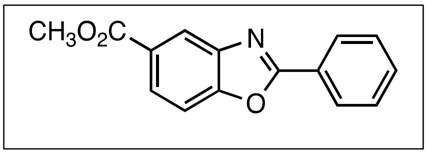

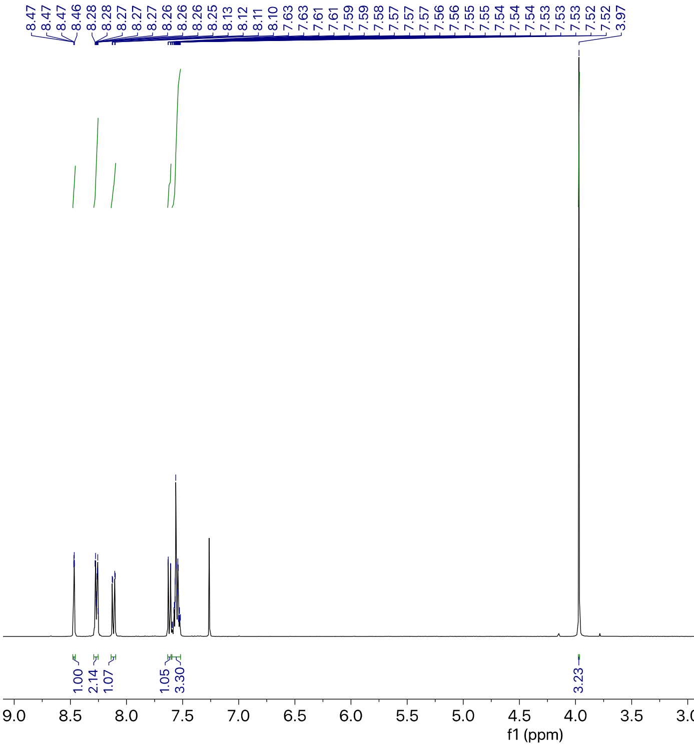

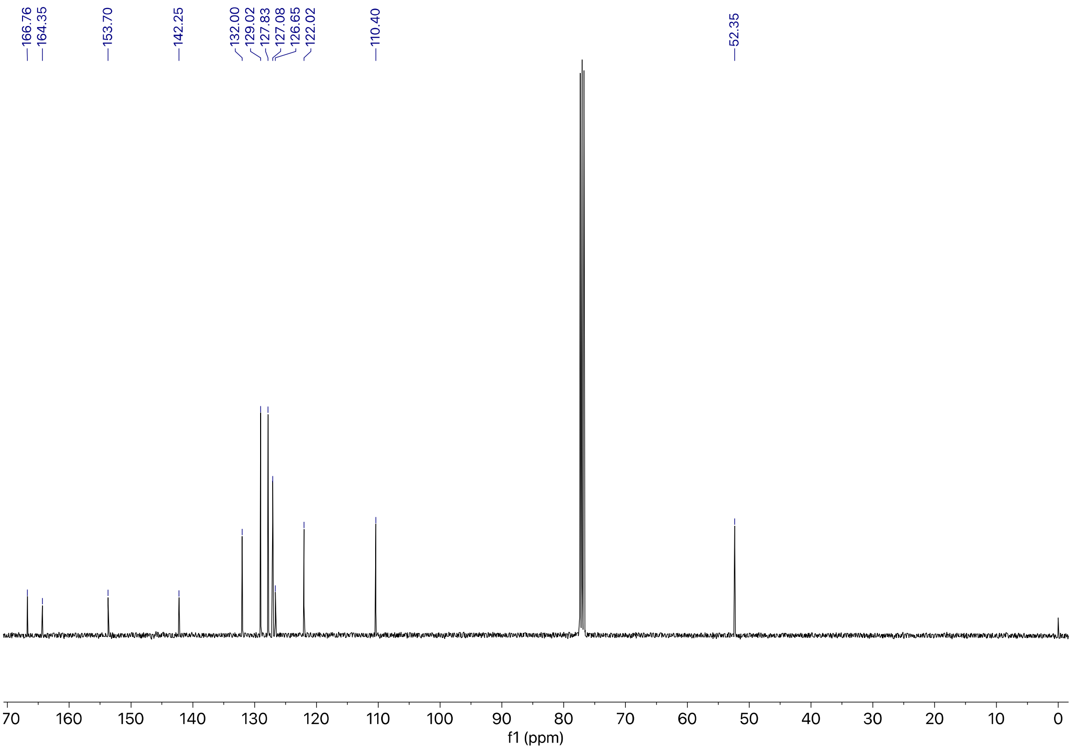

^{1}H and ^{13}C NMR for Methyl 2-(3-methylphenyl)benzo[*d*]oxazole-5-carboxylate (**60**)

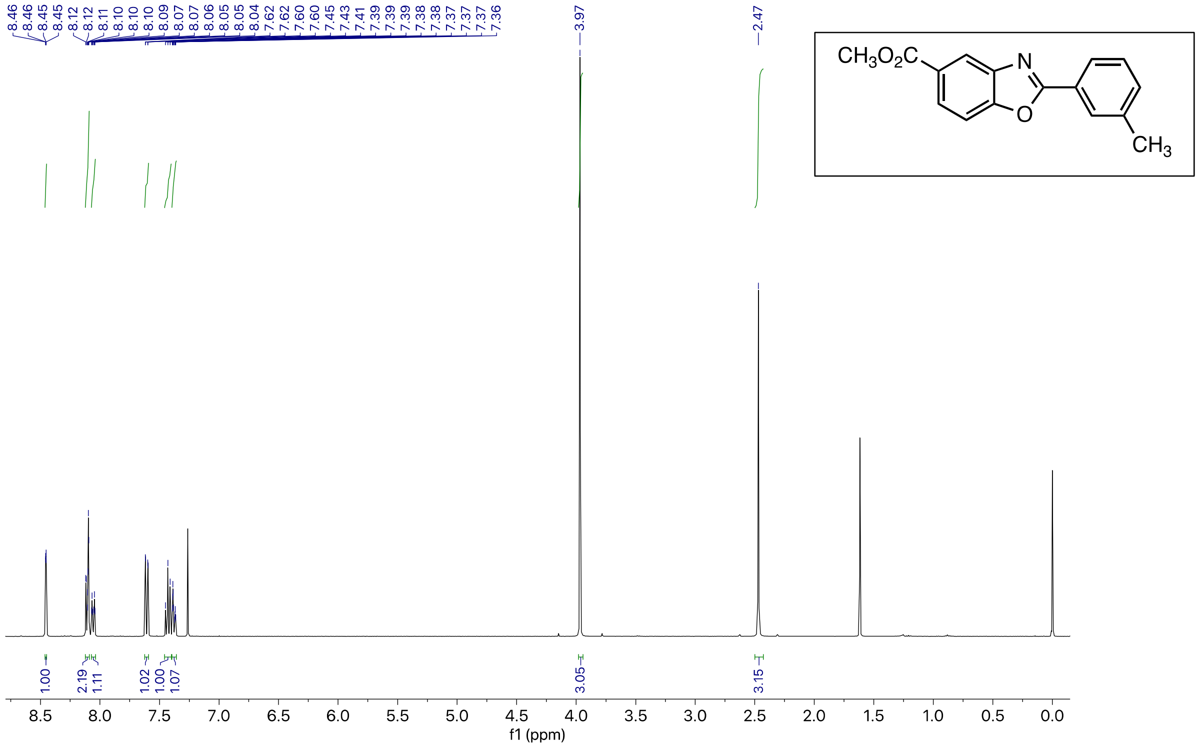

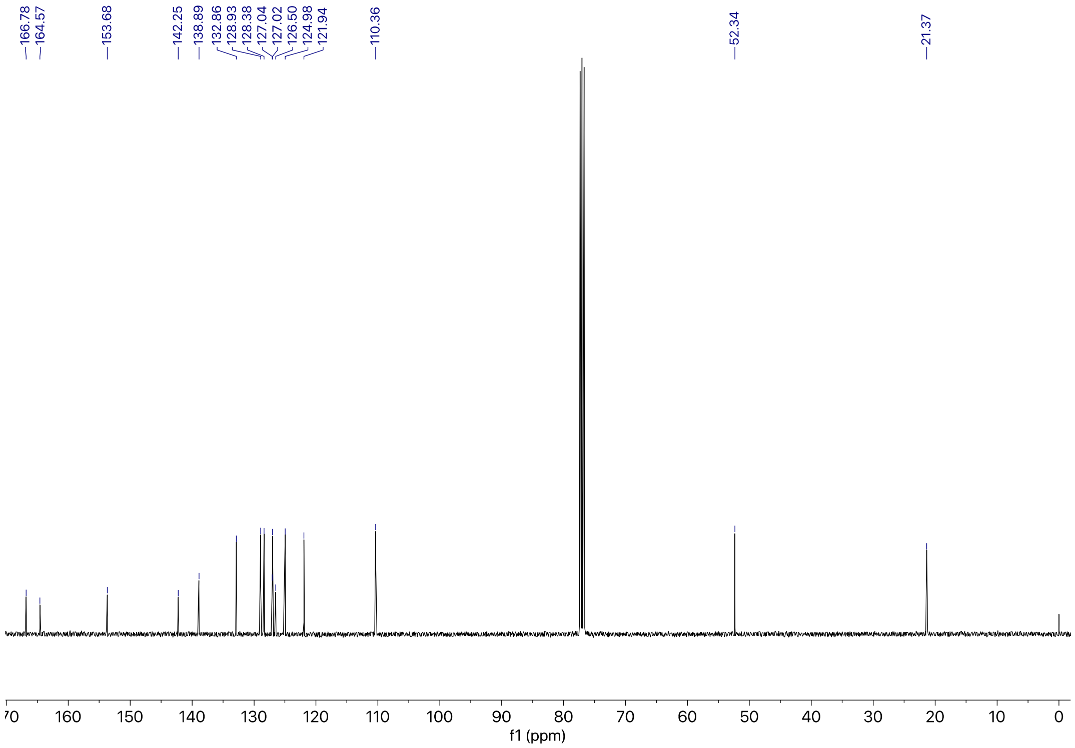

^{1}H and ^{13}C NMR for Methyl 2-(4-methylphenyl)benzo[*d*]oxazole-5-carboxylate (**61**)

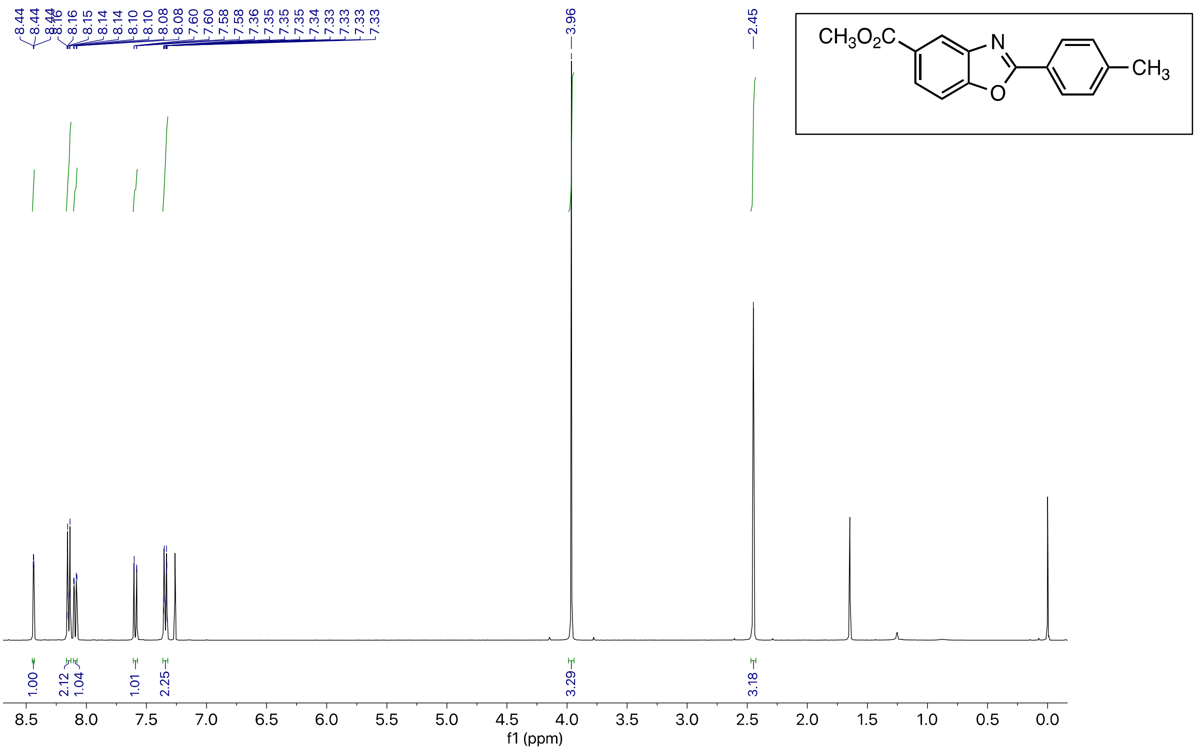

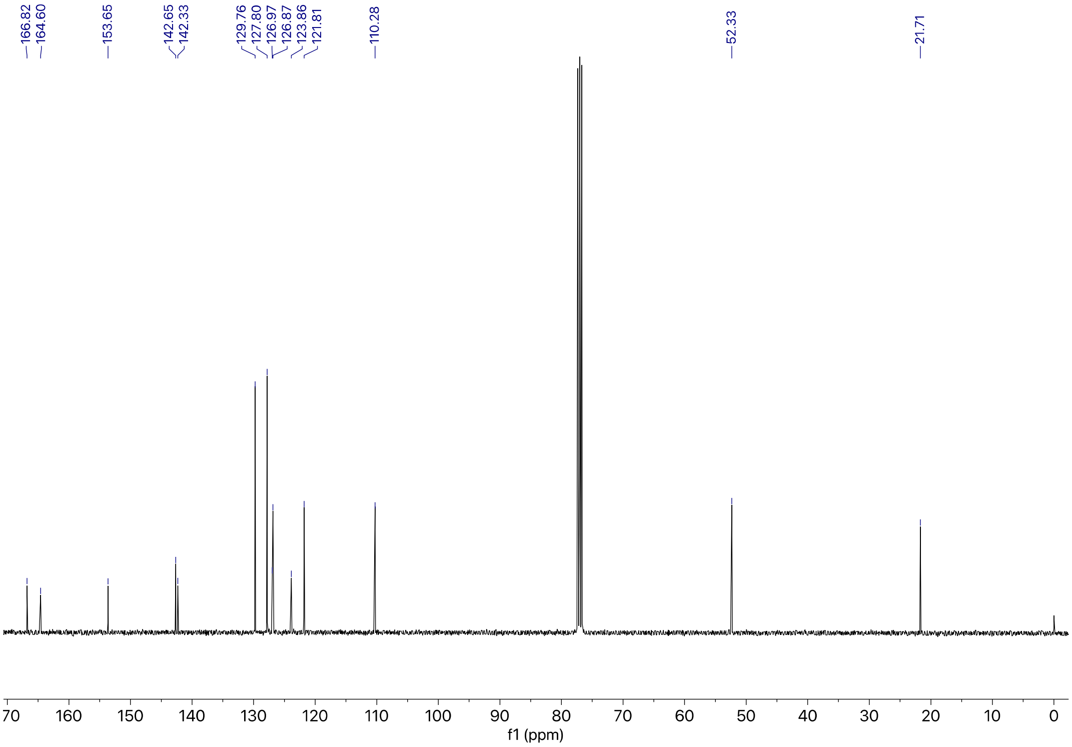

^{1}H and ^{13}C NMR for Methyl 2-(4-methoxyphenyl)benzo[*d*]oxazole-5-carboxylate (**62**)

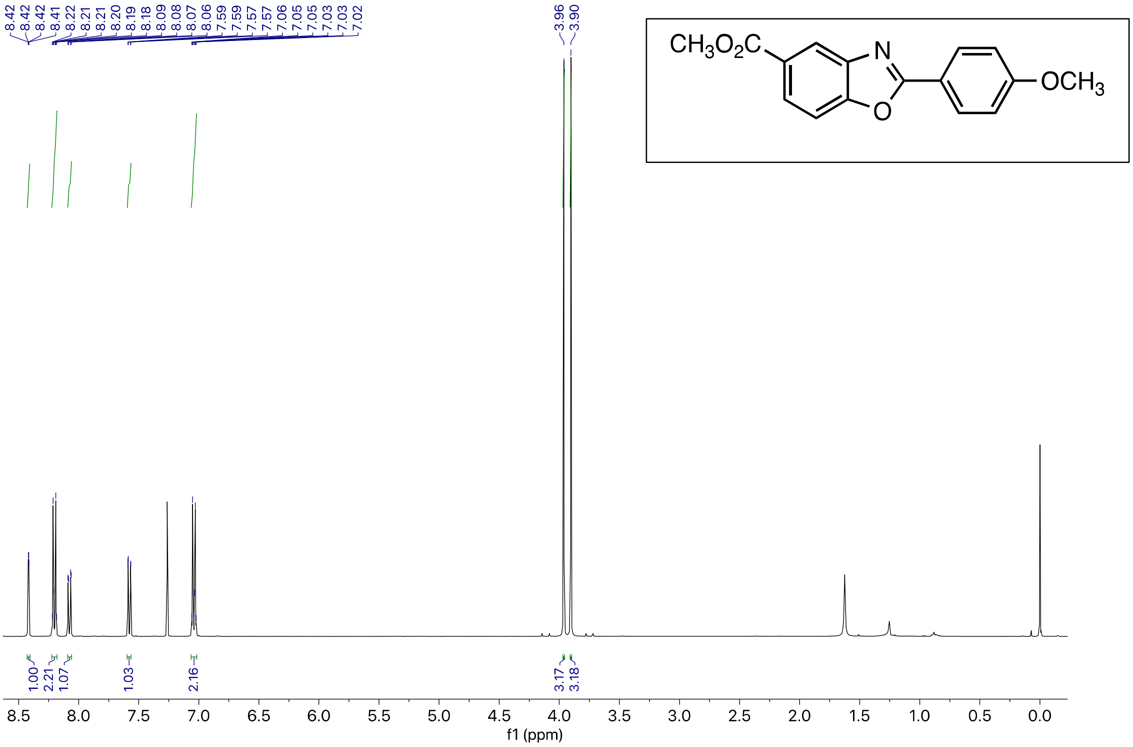

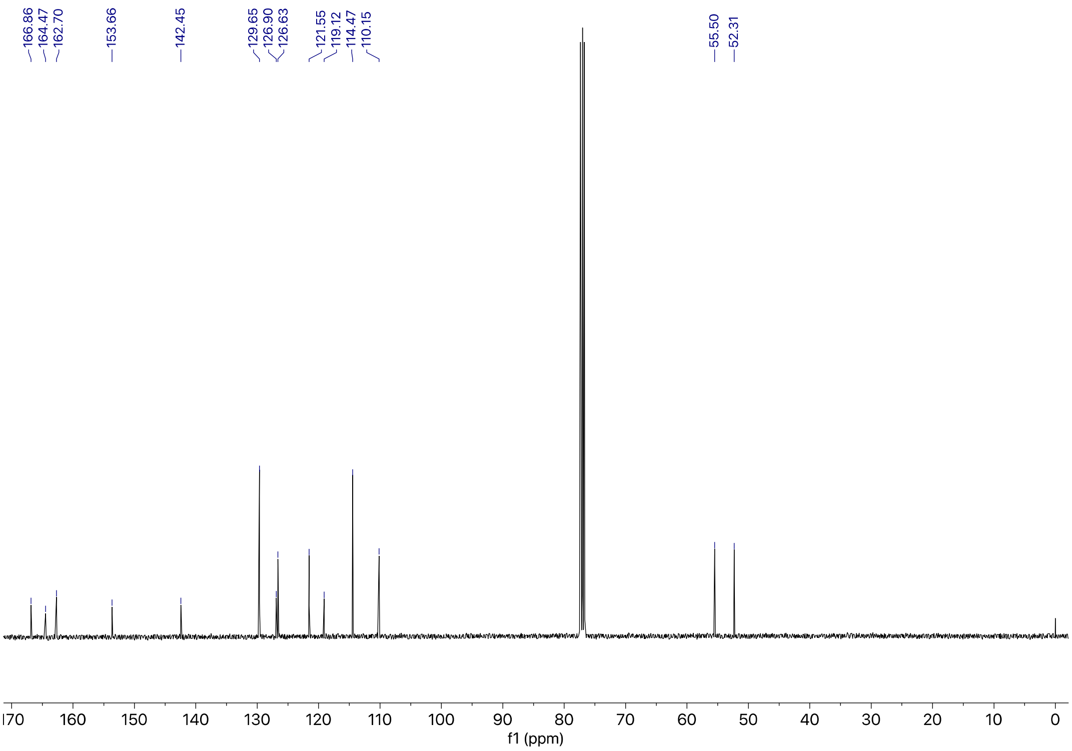

^{1}H and ^{13}C NMR for Methyl 2-(2-fluorophenyl)benzo[*d*]oxazole-5-carboxylate (**63**)

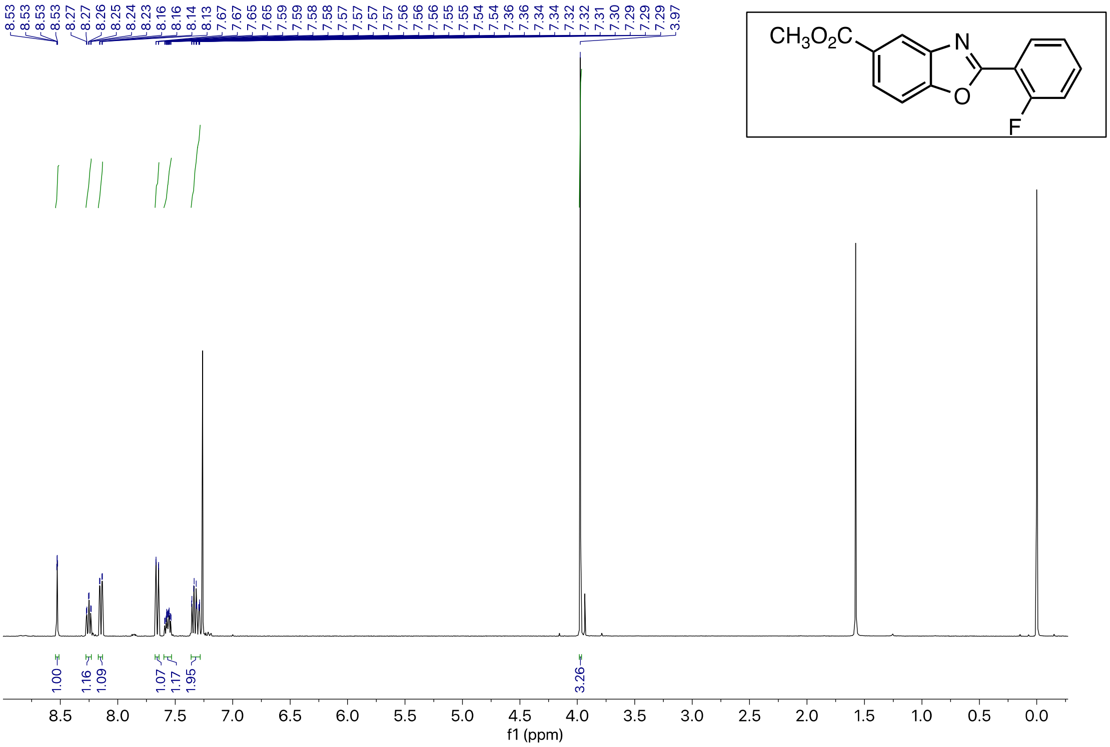

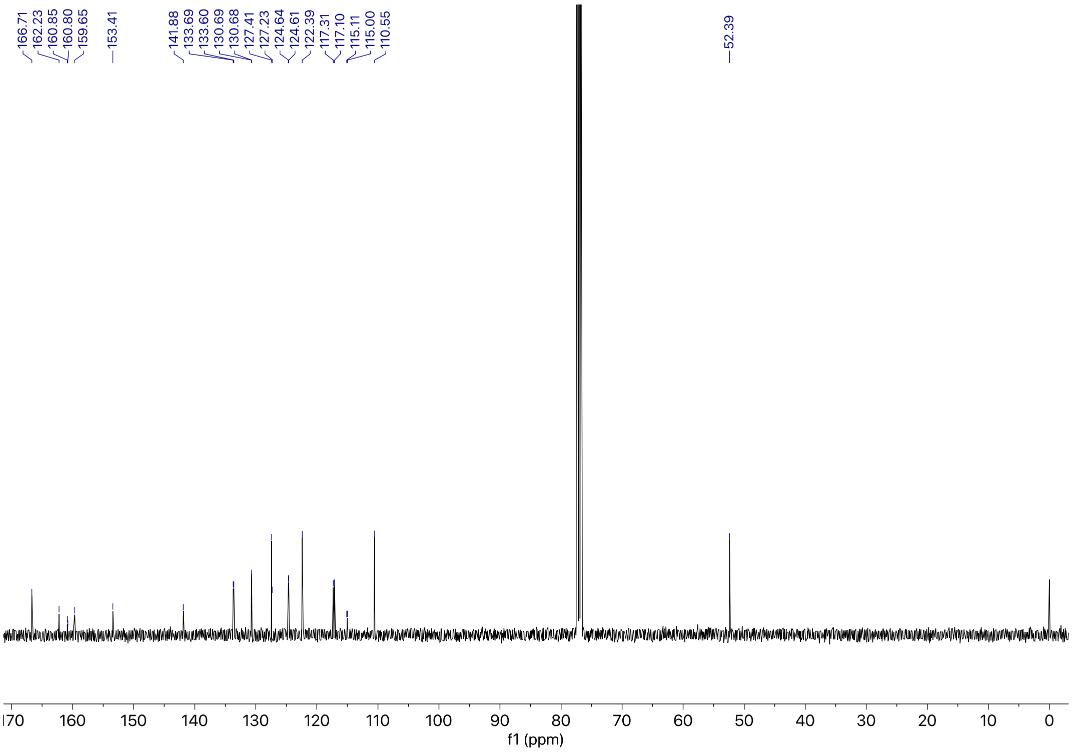

^{1}H and ^{13}C NMR for Methyl 2-(3-chlorophenyl)benzo[*d*]oxazole-5-carboxylate (**64**)

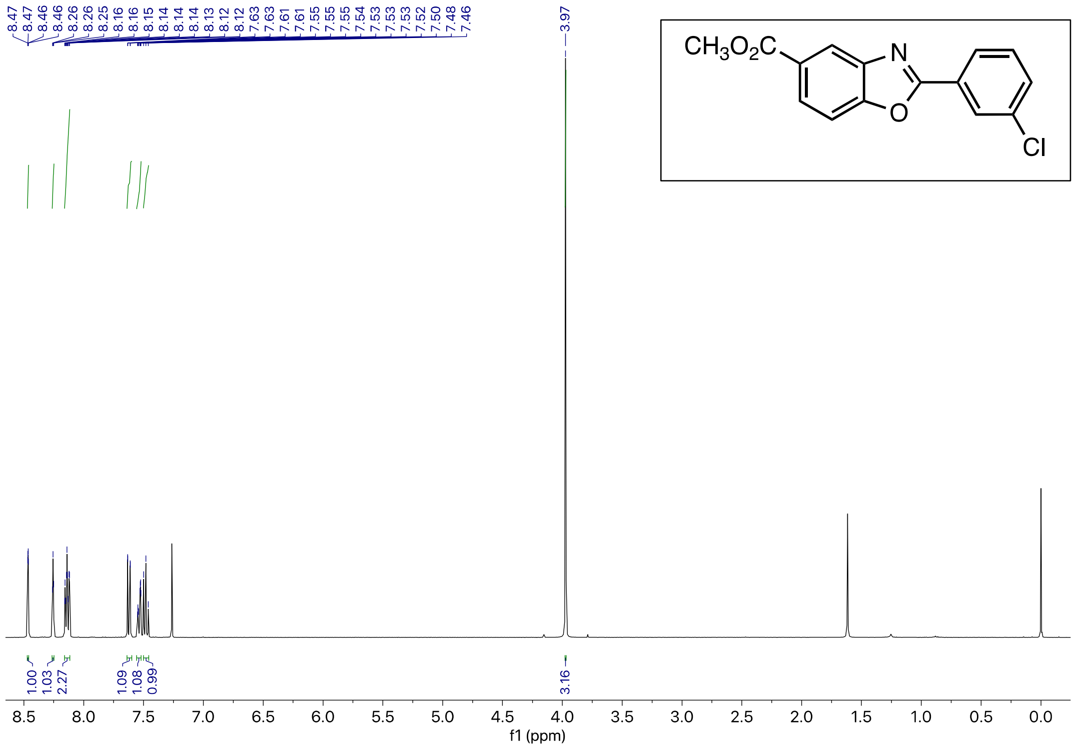

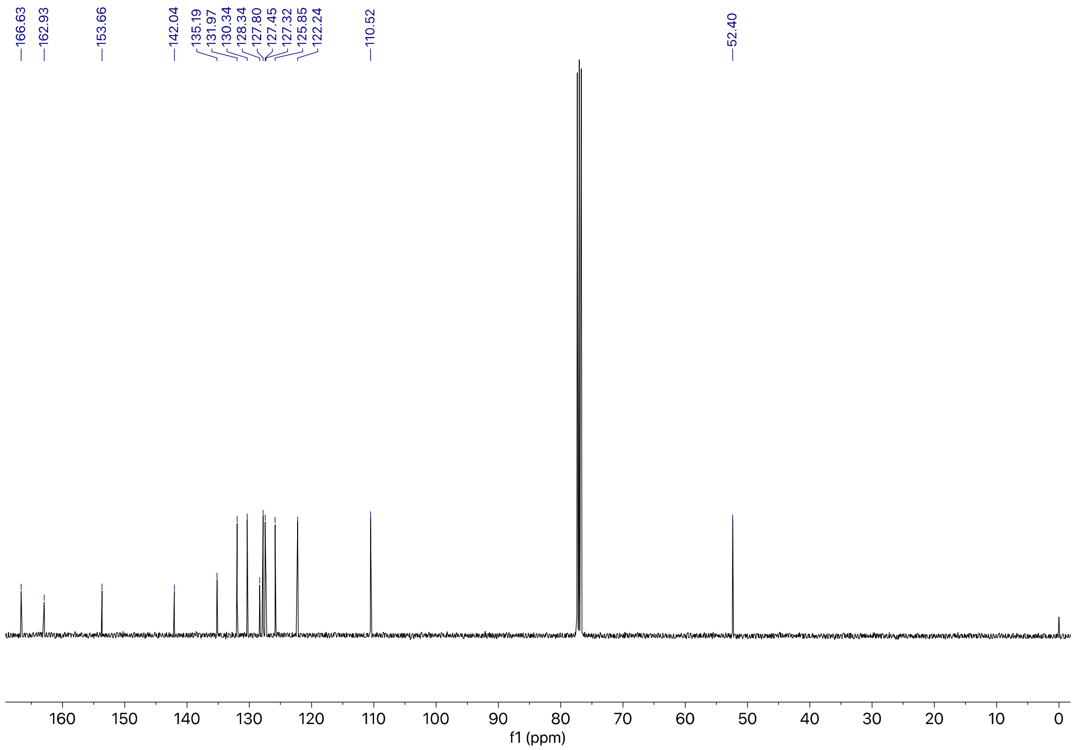

^{1}H and ^{13}C NMR for Methyl 2-(4-chlorophenyl)benzo[*d*]oxazole-5-carboxylate (**65**)

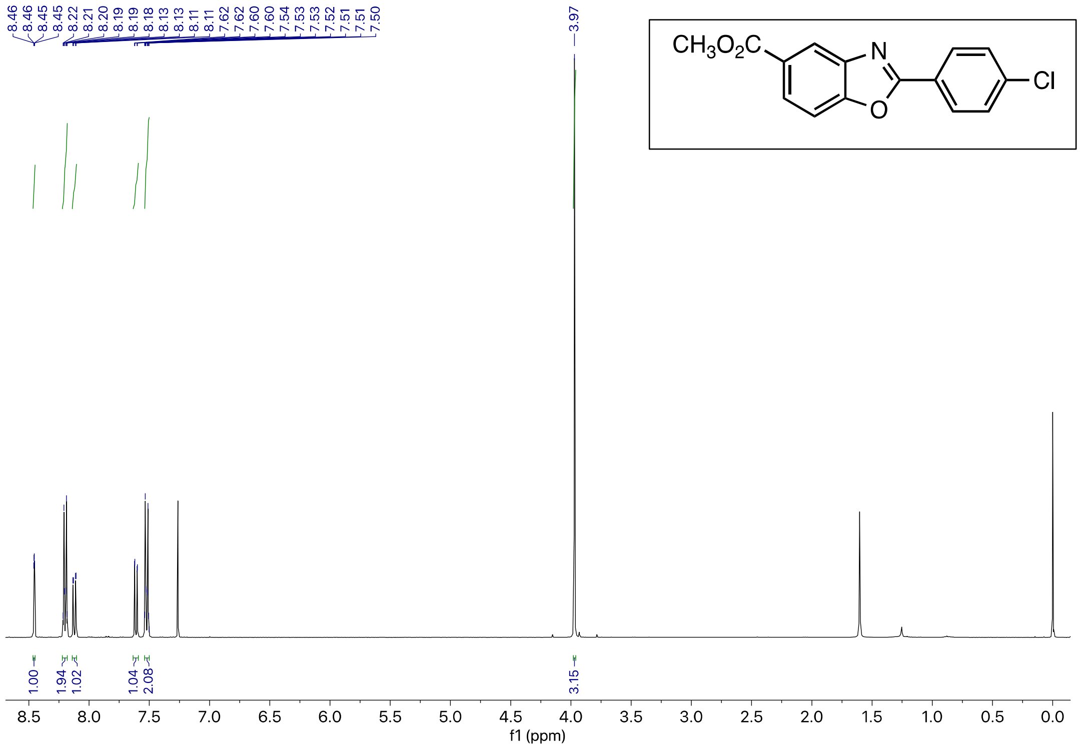

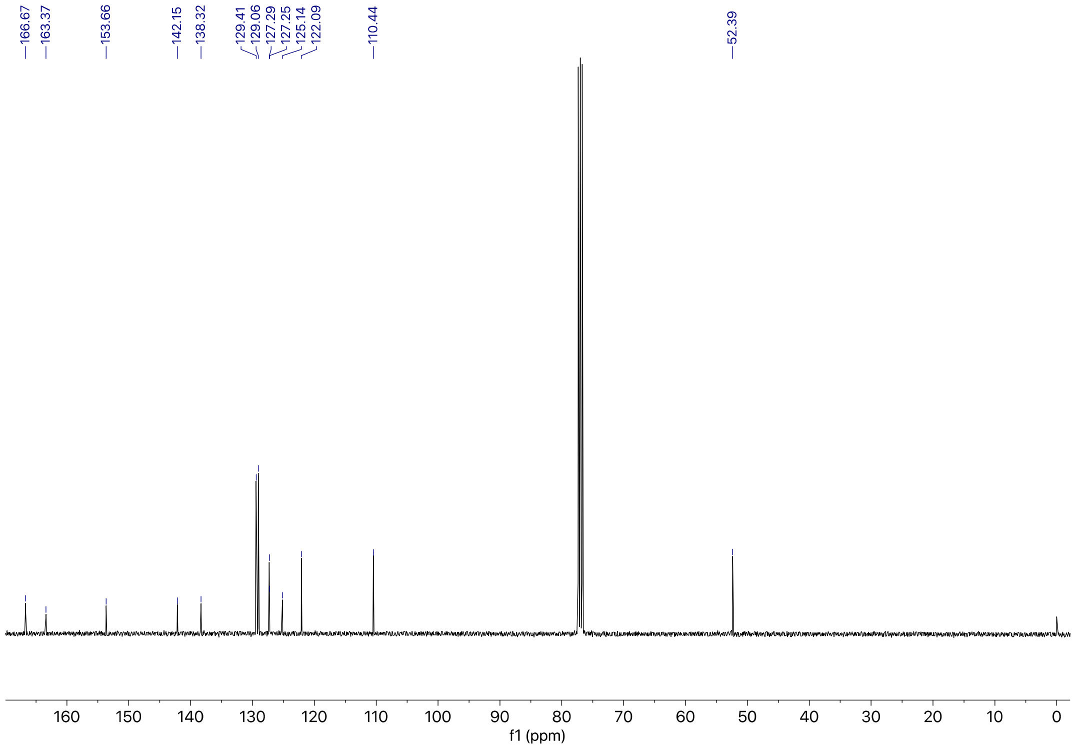

^{1}H and ^{13}C NMR for 2-Phenyl-5-(trifluoromethyl)benzo[*d*]oxazole (**66**)

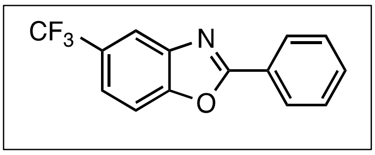

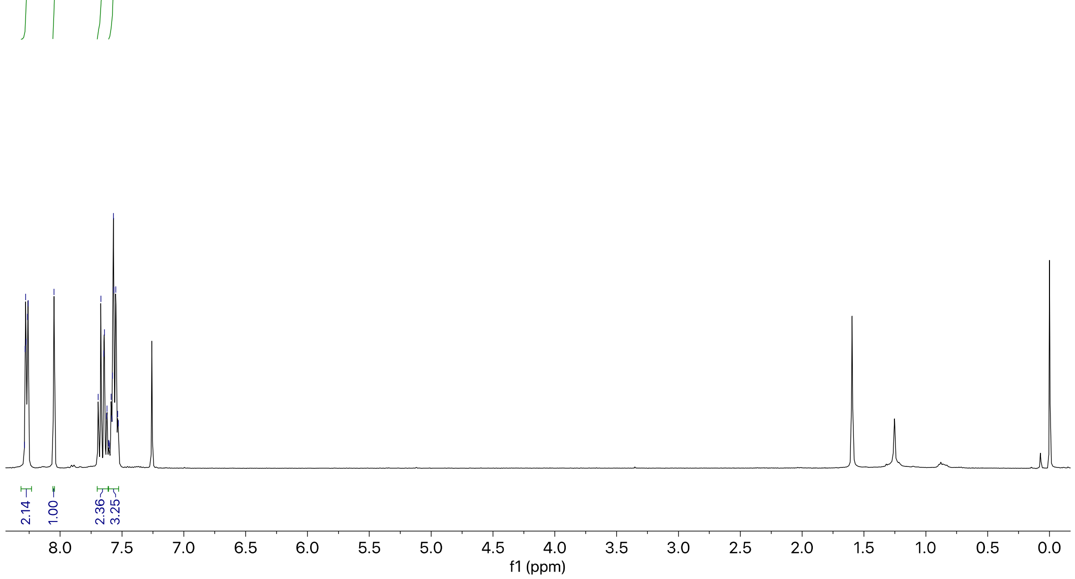

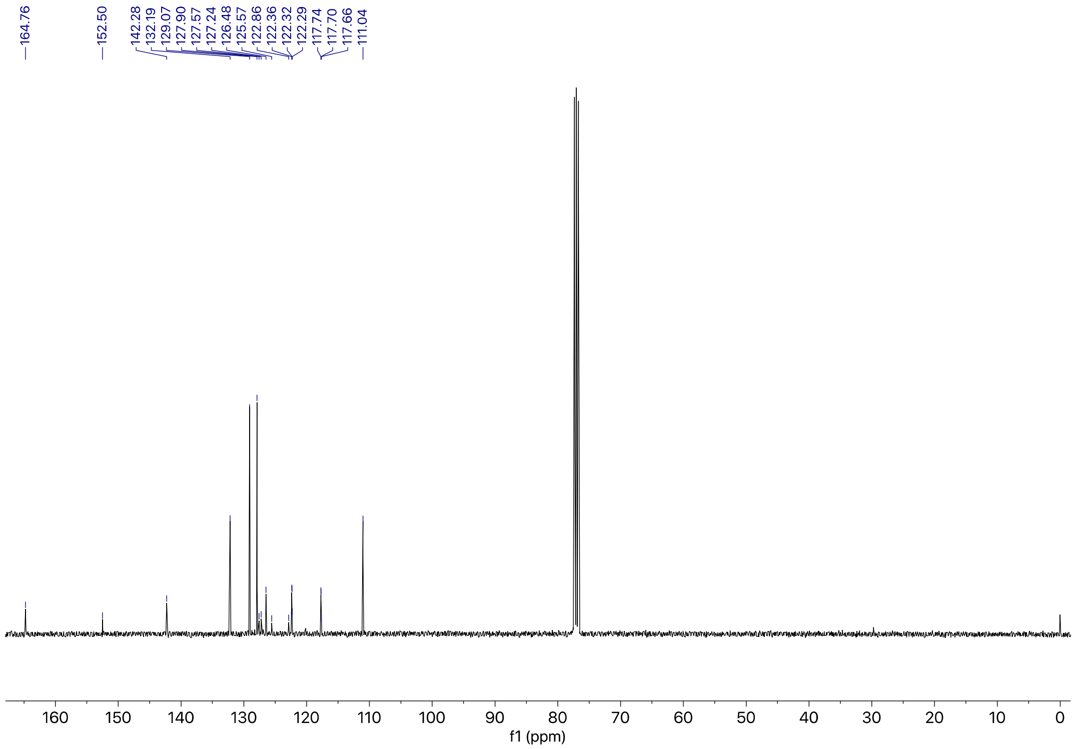

^{19}F NMR for 2-Phenyl-5-(trifluoromethyl)benzo[*d*]oxazole (**66**)

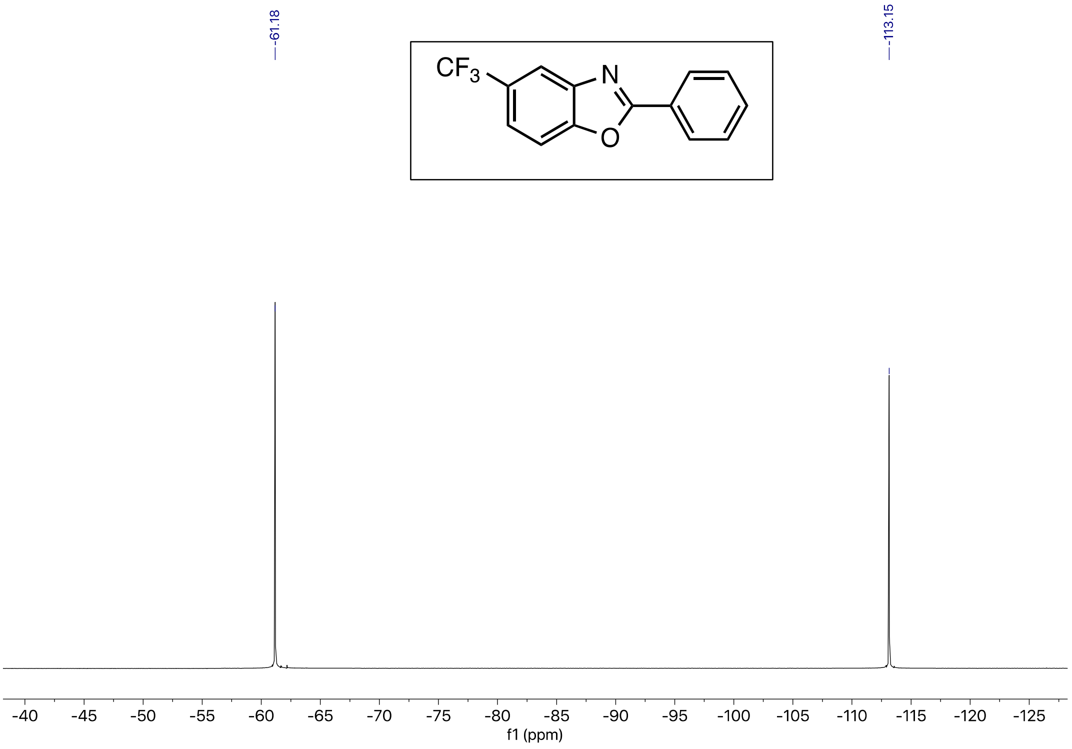

^{1}H and ^{13}C NMR for 2-(3-Methylphenyl)-5-(trifluoromethyl)benzo[*d*]oxazole (**67**)

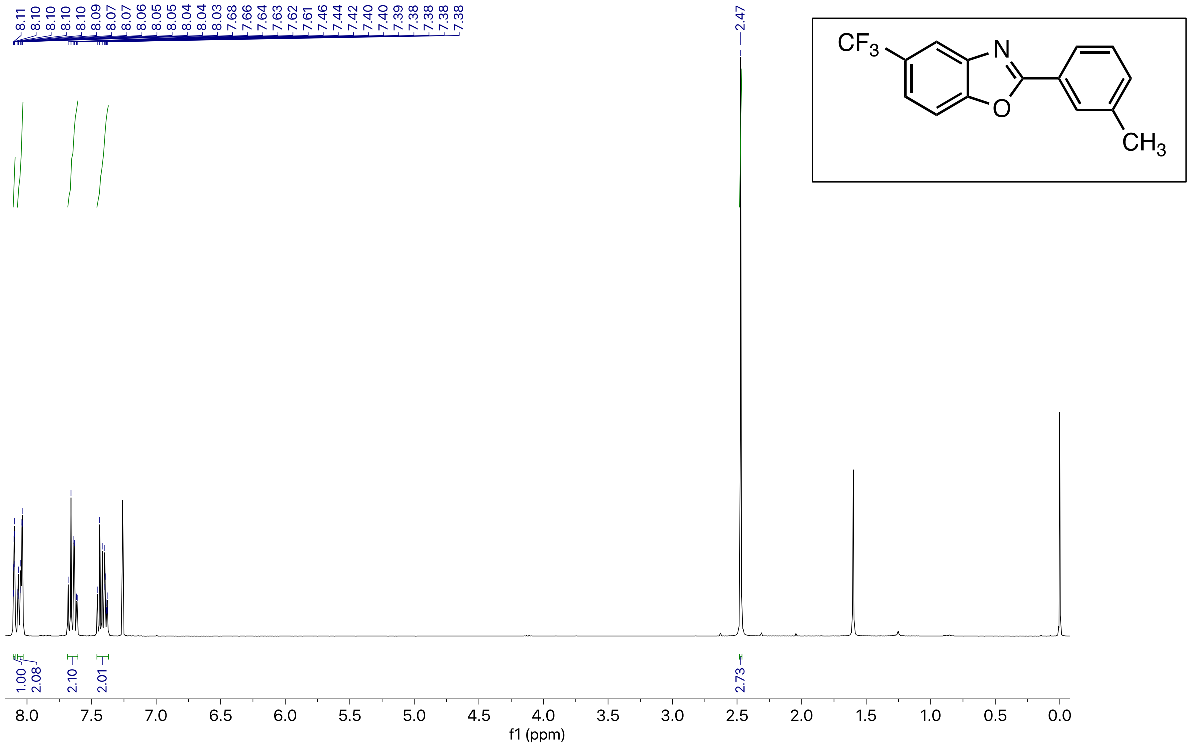

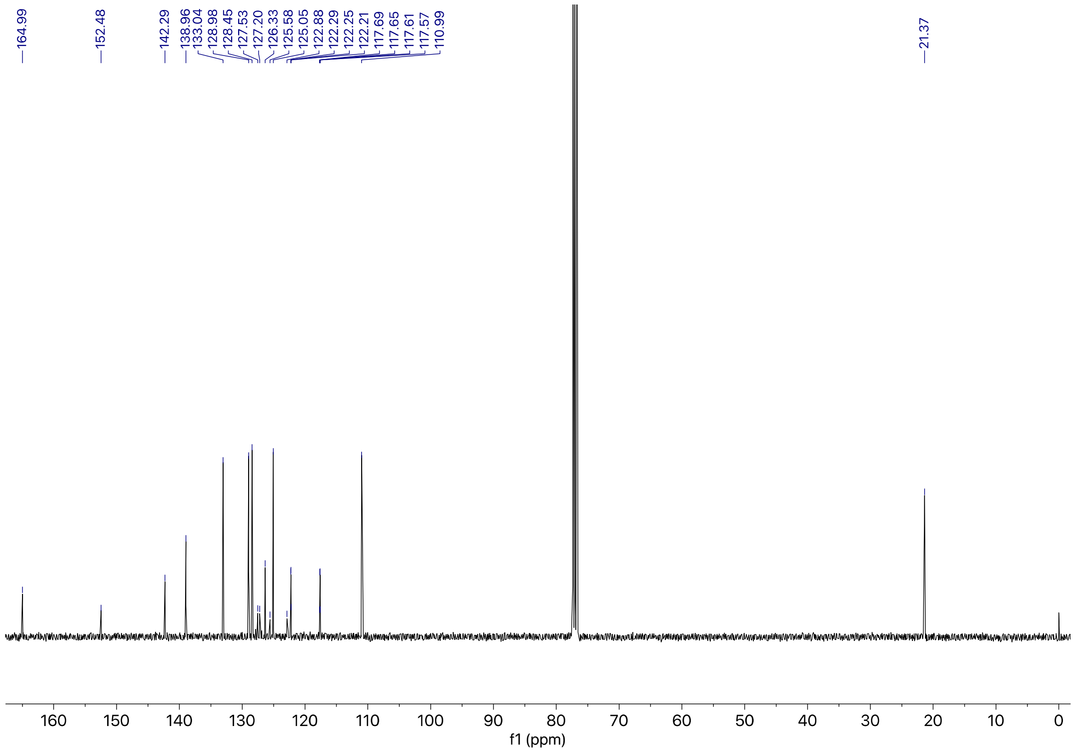

^{19}F NMR for 2-(3-Methylphenyl)-5-(trifluoromethyl)benzo[*d*]oxazole (**67**)

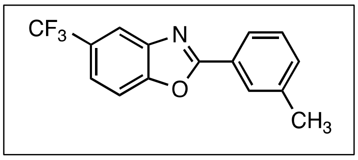

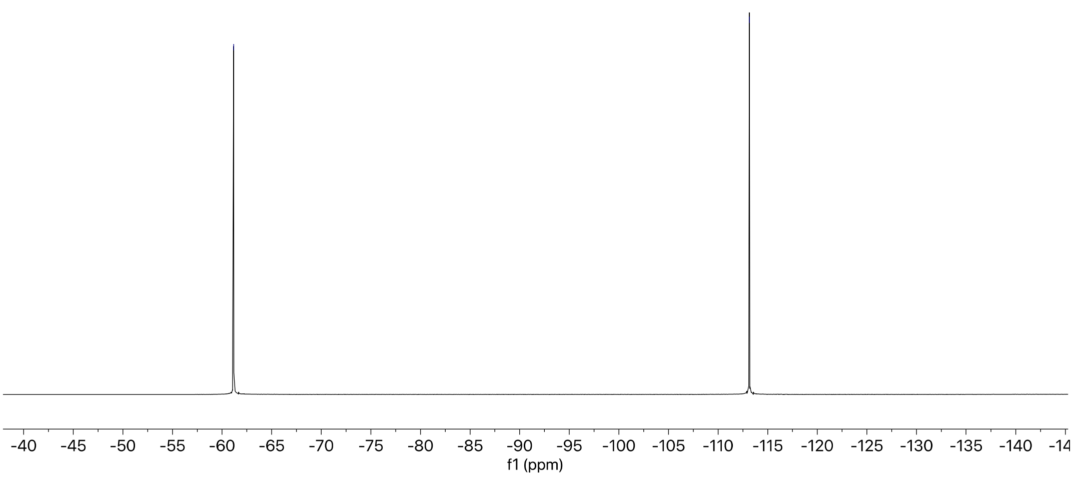

^{1}H and ^{13}C NMR for 2-(4-Methylphenyl)-5-(trifluoromethyl)benzo[*d*]oxazole (**68**)

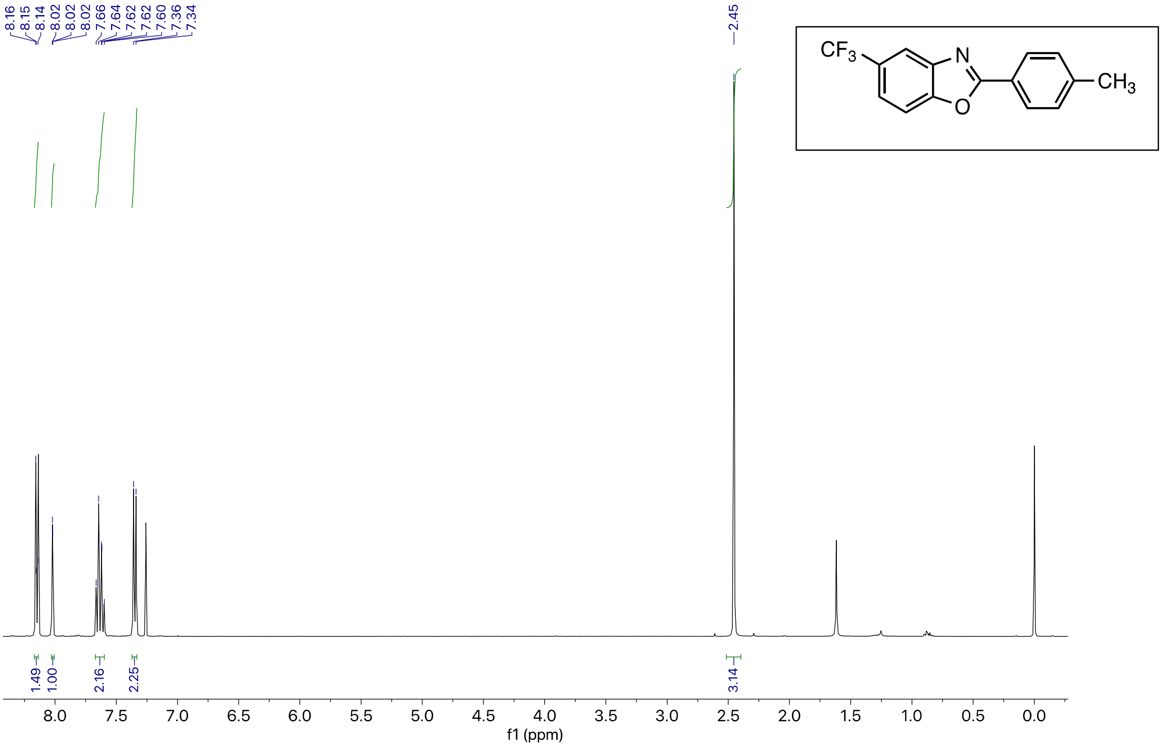

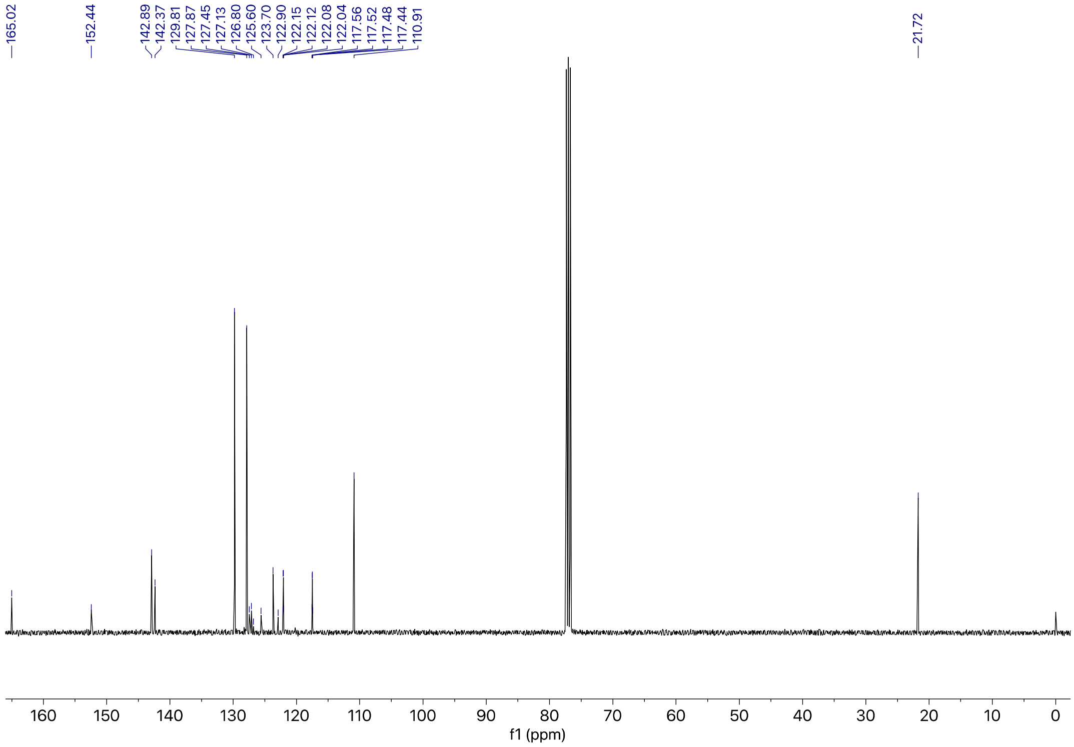

^{19}F NMR for 2-(4-Methylphenyl)-5-(trifluoromethyl)benzo[*d*]oxazole (**68**)

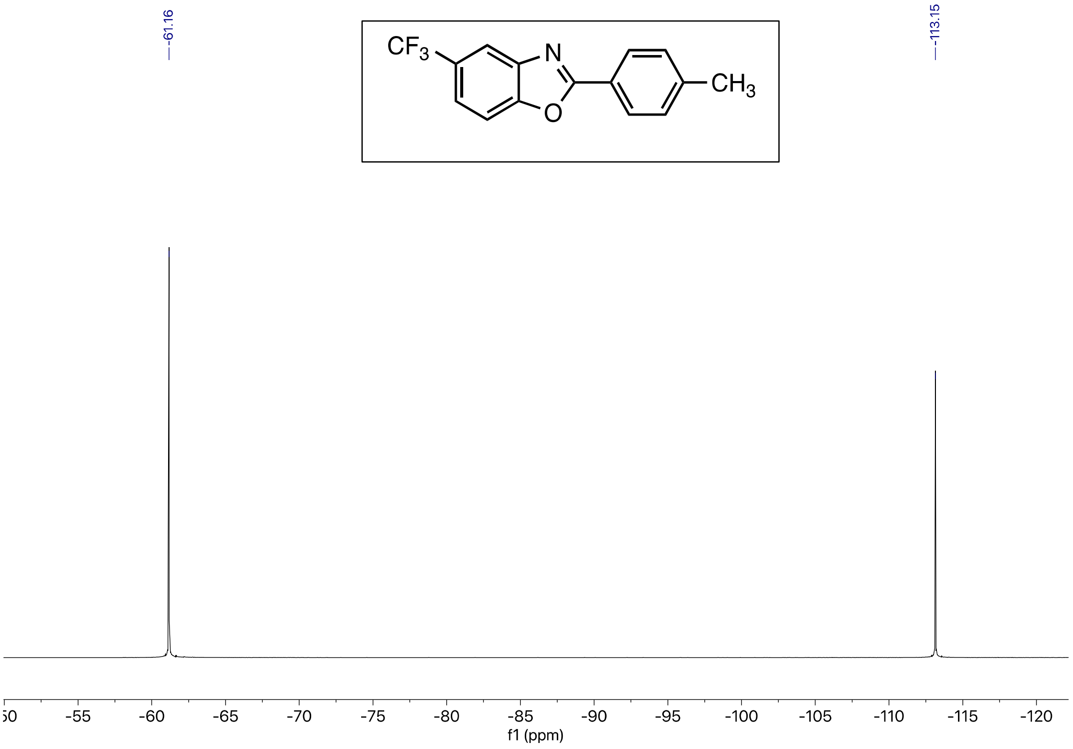

^{1}H and ^{13}C NMR for 2-(4-Methoxyphenyl)-5-(trifluoromethyl)benzo[*d*]oxazole (**69**)

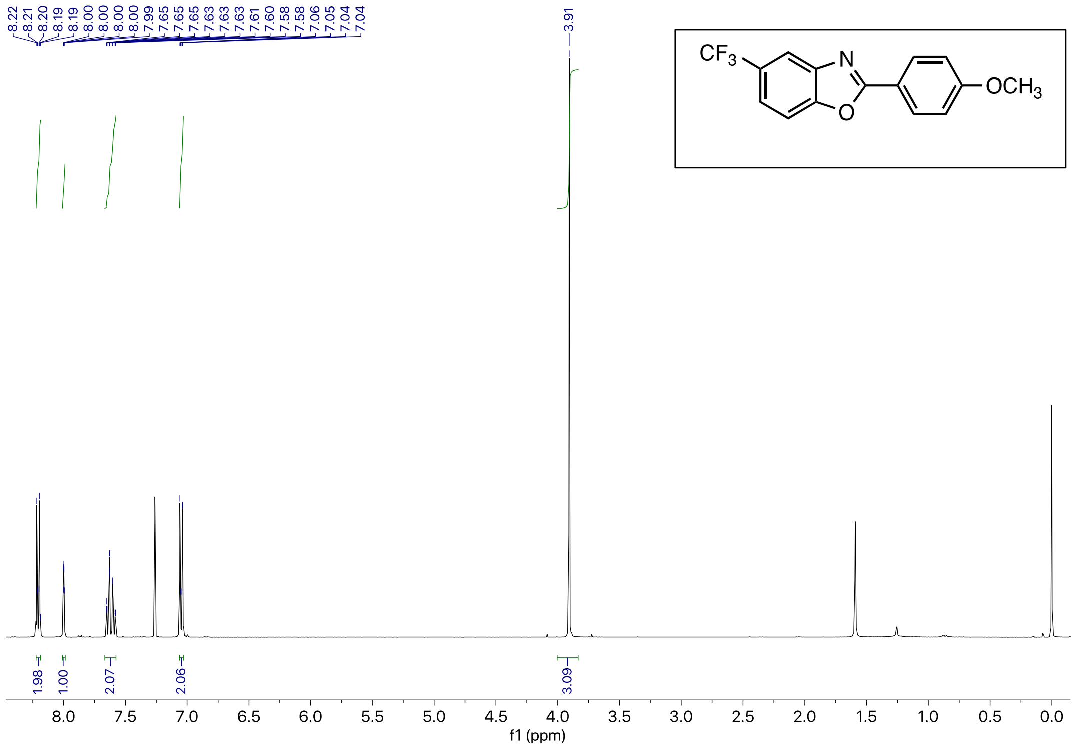

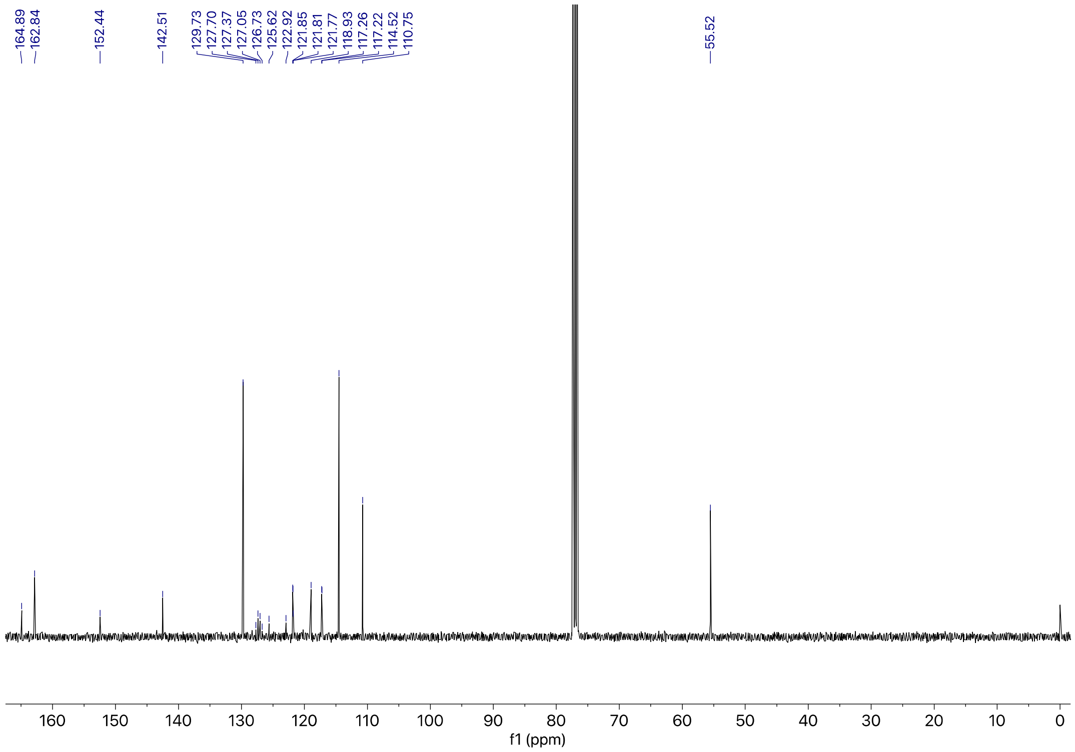

^{19}F NMR for 2-(4-Methoxyphenyl)-5-(trifluoromethyl)benzo[*d*]oxazole (**69**)

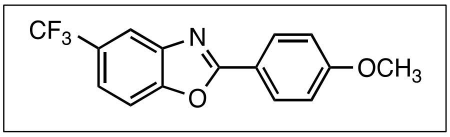

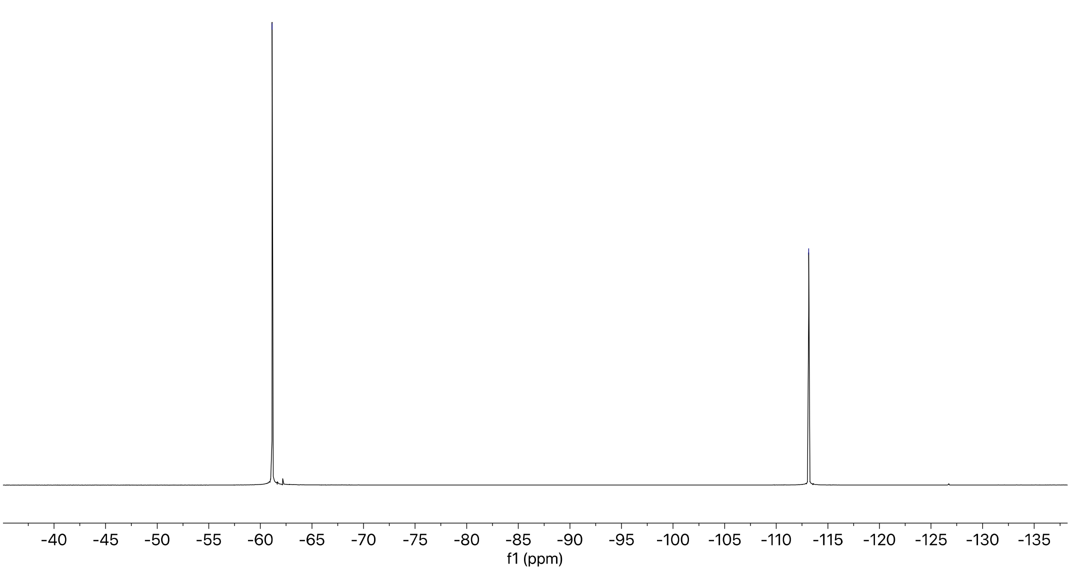

^{1}H and ^{13}C NMR for 2-(2-Fluorophenyl)-5-(trifluoromethyl)benzo[*d*]oxazole (**70**)

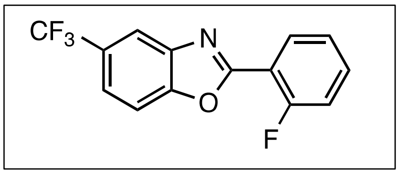

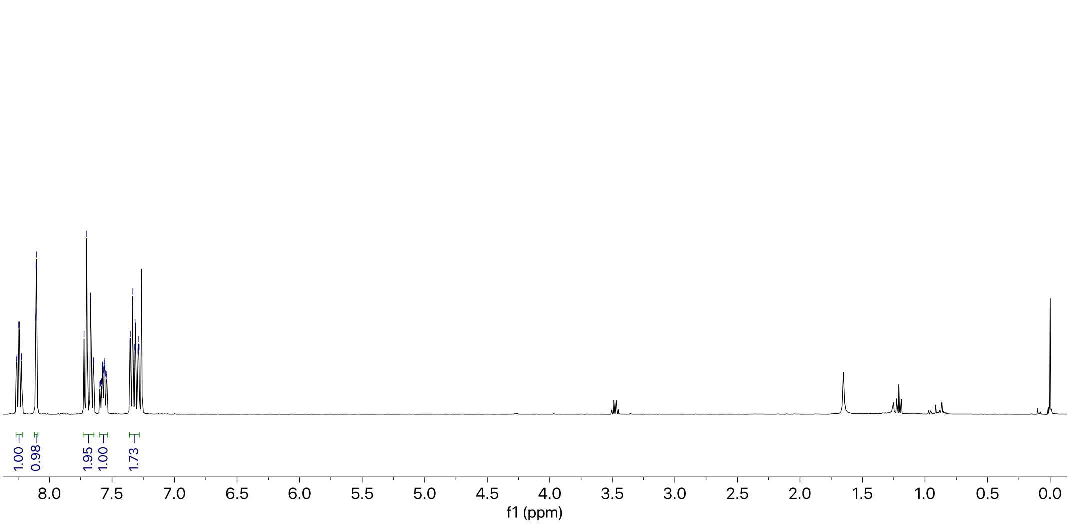

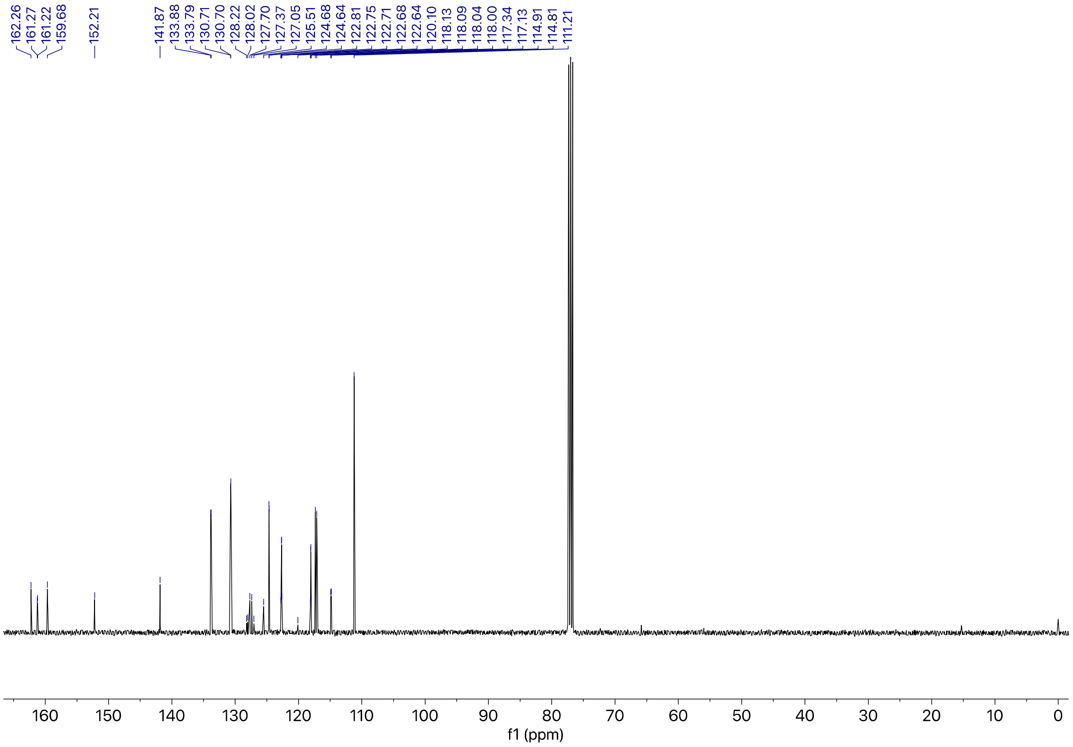

^{19}F NMR for 2-(2-Fluorophenyl)-5-(trifluoromethyl)benzo[*d*]oxazole (**70**)

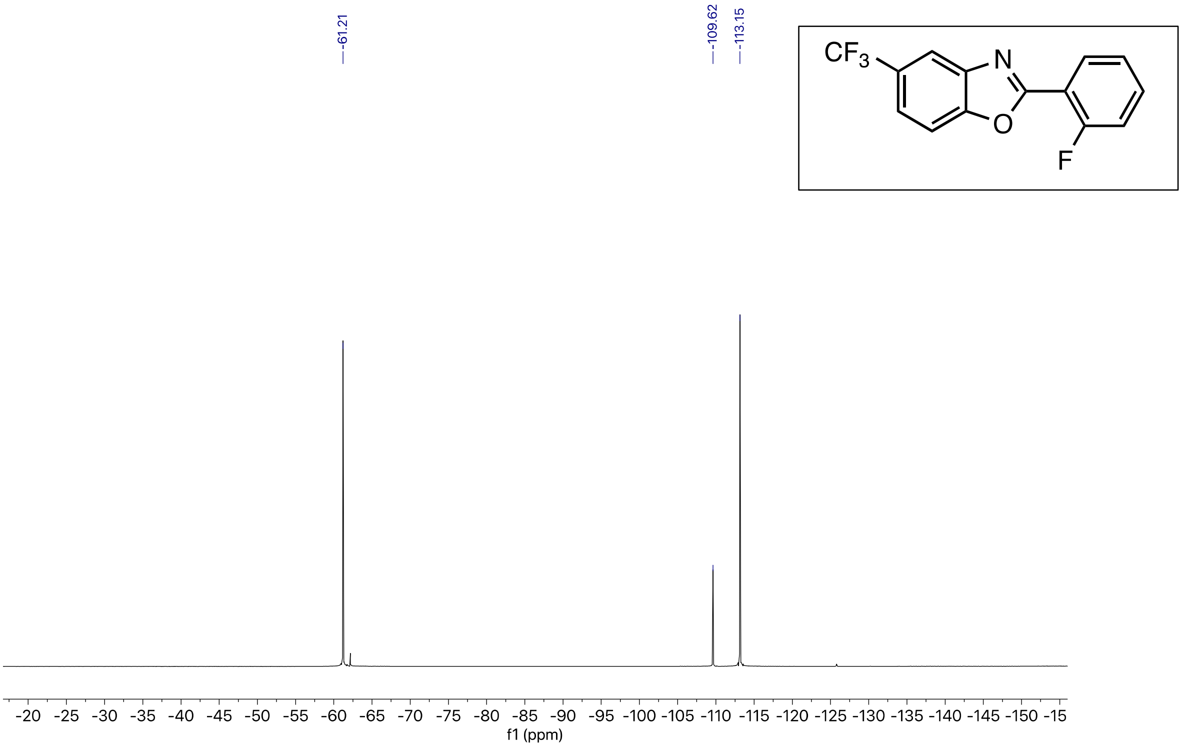

^{1}H and ^{13}C NMR for 2-(3-Chlorophenyl)-5-(trifluoromethyl)benzo[*d*]oxazole (**71**)

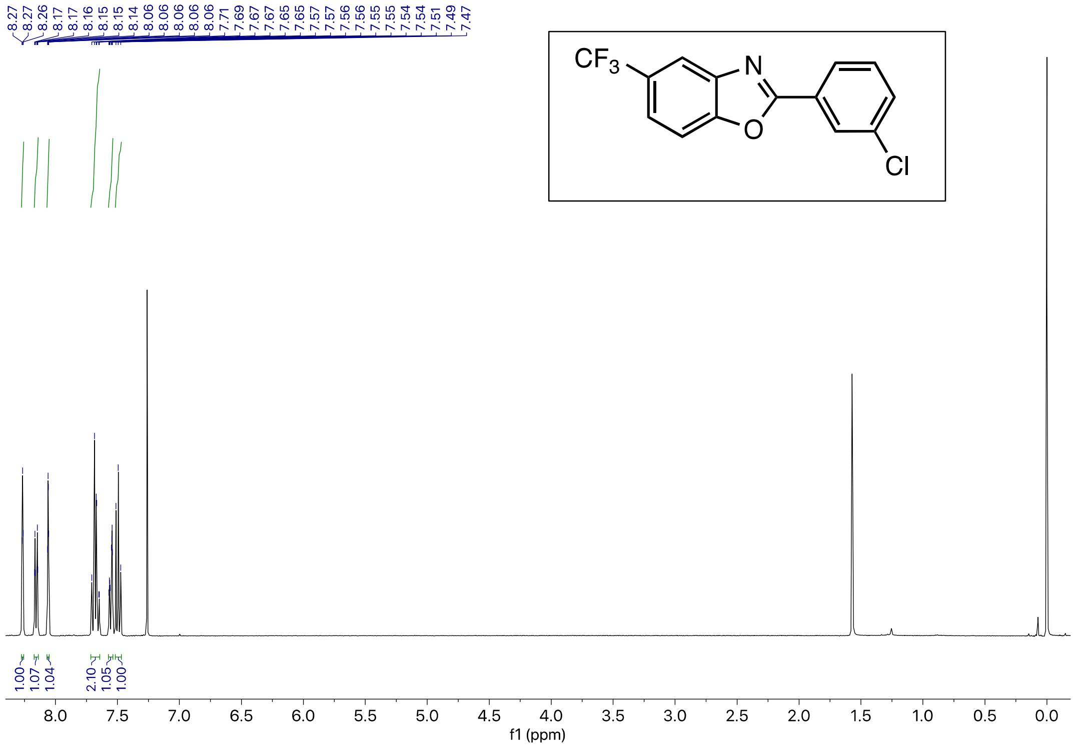

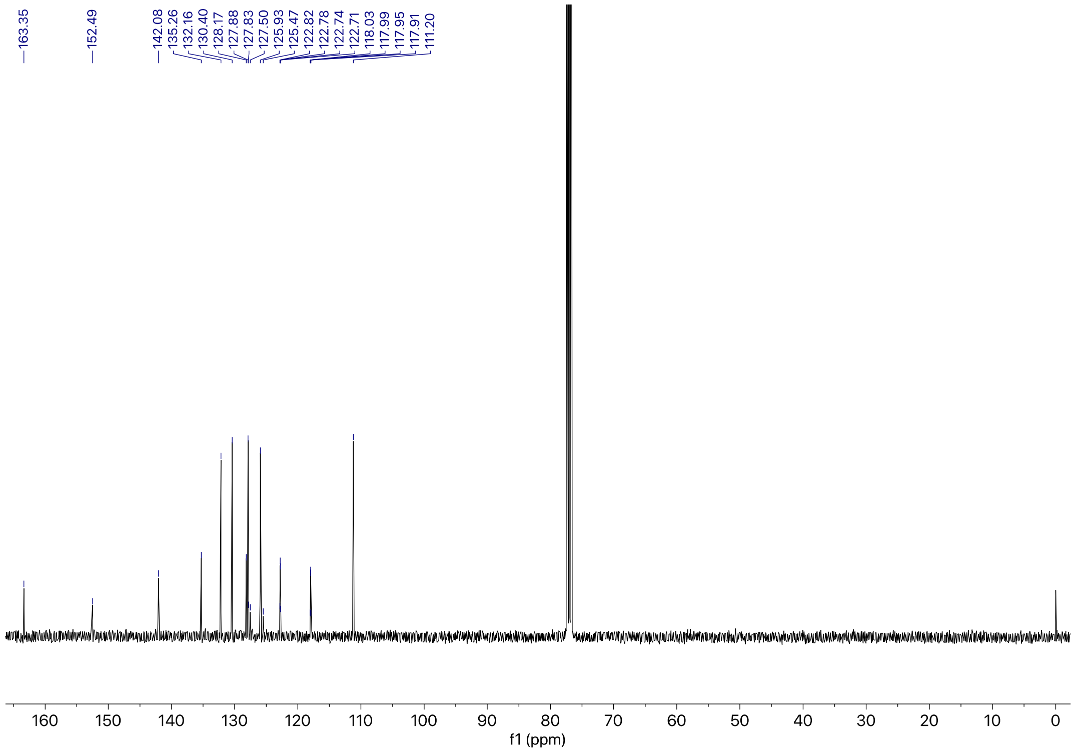

^{19}F NMR for 2-(3-Chlorophenyl)-5-(trifluoromethyl)benzo[*d*]oxazole (**71**)

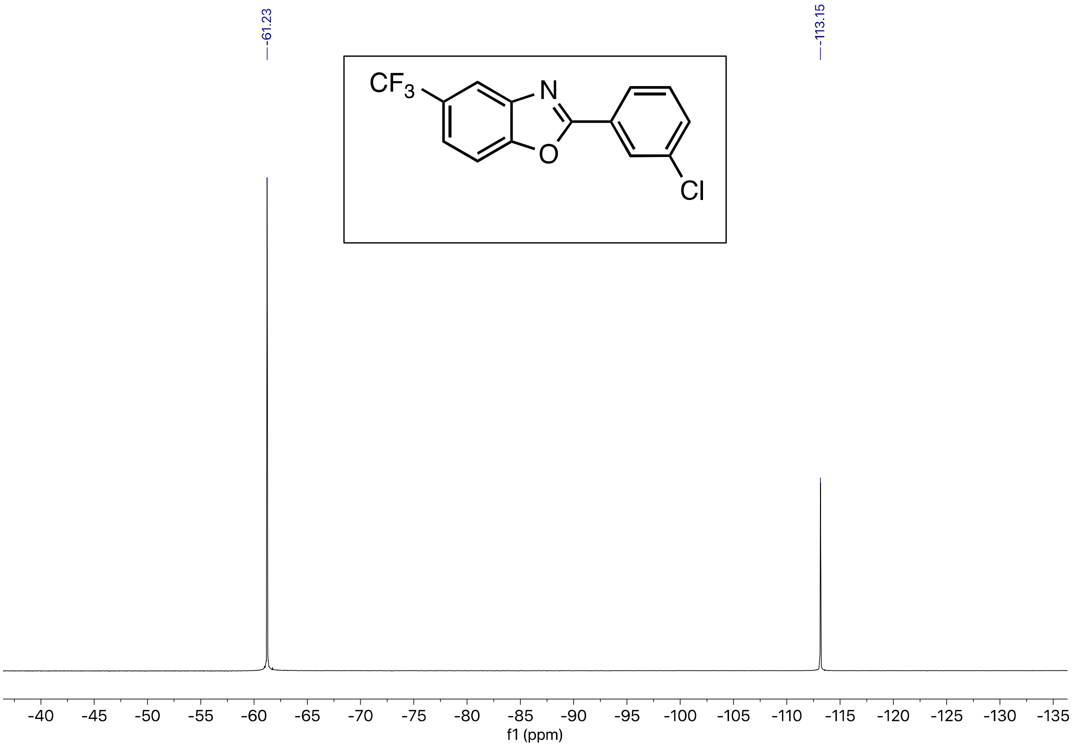

^{1}H and ^{13}C NMR for 2-(4-Chlorophenyl)-5-(trifluoromethyl)benzo[*d*]oxazole (**72**)

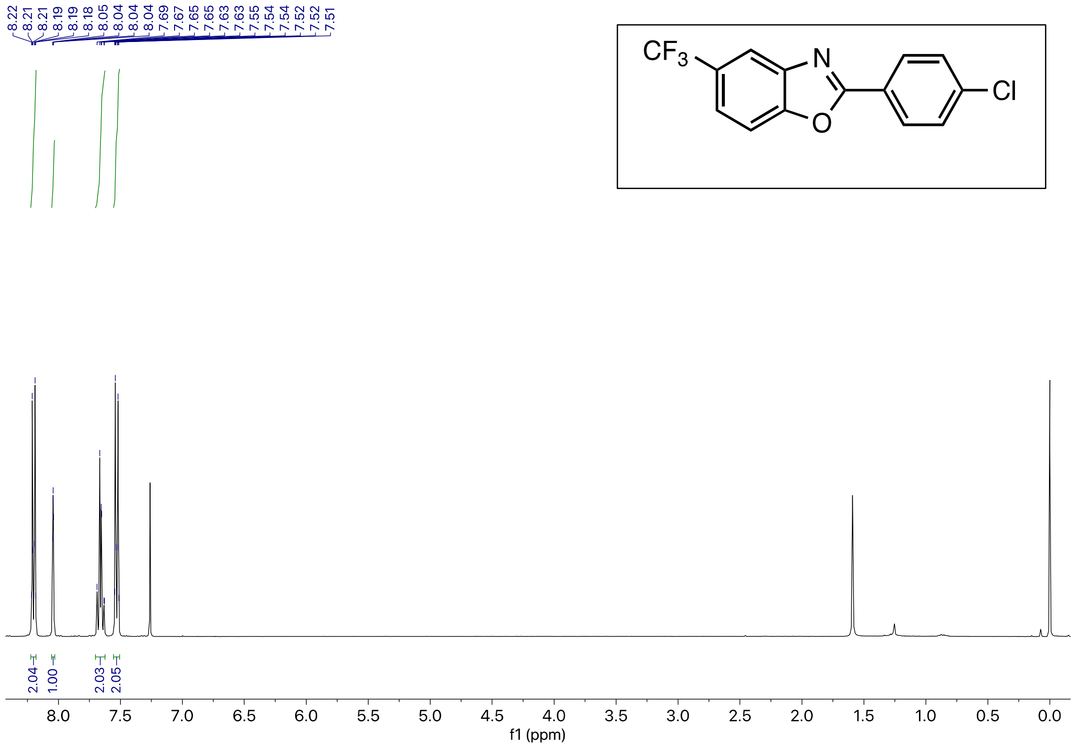

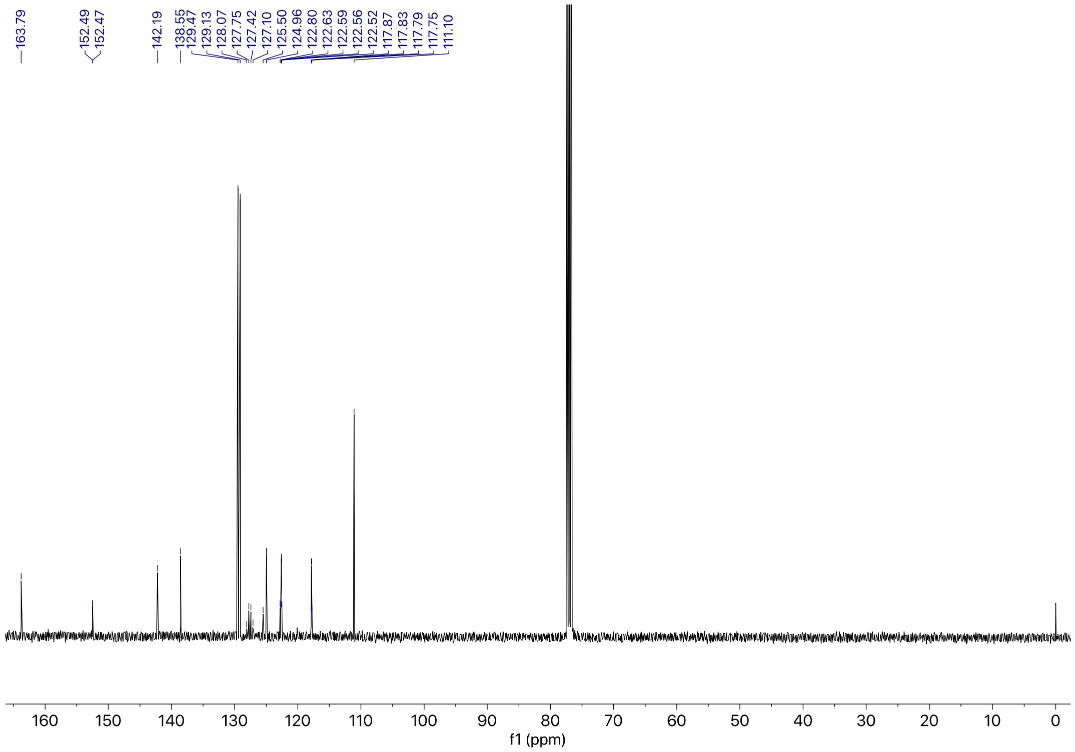

^{19}F NMR for 2-(4-Chlorophenyl)-5-(trifluoromethyl)benzo[*d*]oxazole (**72**)

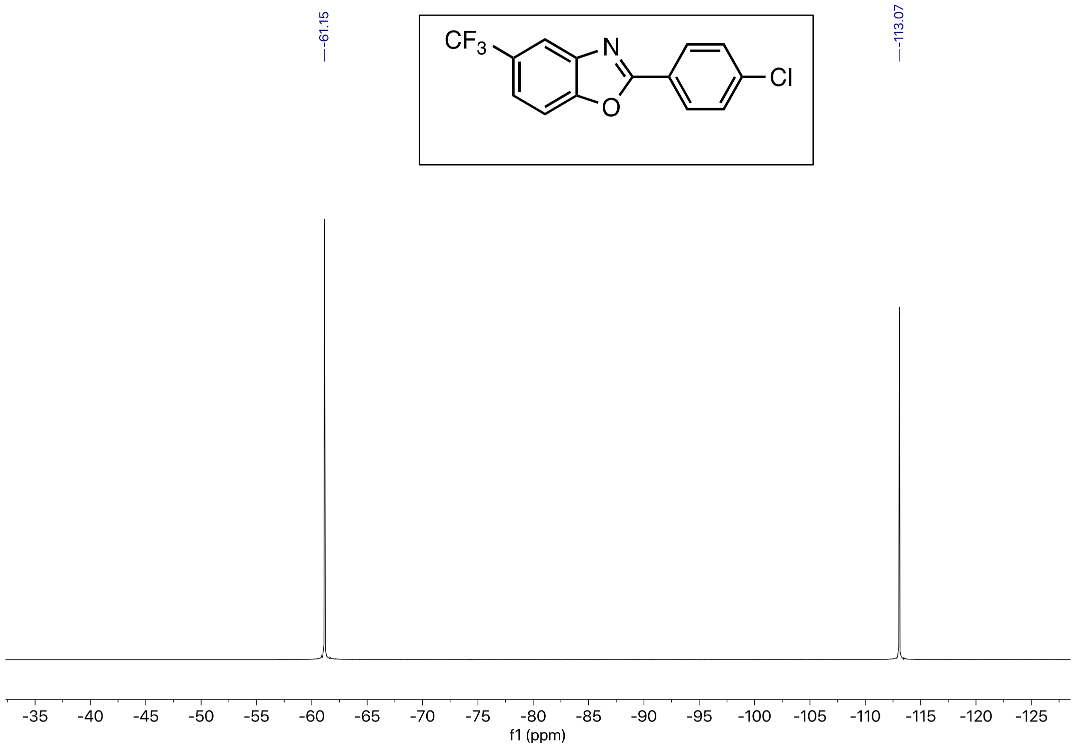

Supplement: Supplementary file 1 [file molecules-29-04322-s001.zip › molecules-3200260-supplementary.pdf]
